# Biomimetic Cationic Cyclopropanation Enables an Efficient Chemoenzymatic Synthesis of 6,8-Cycloeudesmanes

**DOI:** 10.1021/jacs.2c13116

**Published:** 2023-02-28

**Authors:** Phillip S. Grant, Ricardo Meyrelles, Oliver Gajsek, Gerhard Niederacher, Boris Maryasin, Nuno Maulide

**Affiliations:** † Institute of Organic Chemistry, University of Vienna, Vienna 1090, Austria; ‡ Institute of Theoretical Chemistry, University of Vienna, Vienna 1090, Austria; § Institute of Biological Chemistry, University of Vienna, Vienna 1090, Austria; ∥ Vienna Doctoral School in Chemistry, University of Vienna, Vienna 1090, Austria

## Abstract

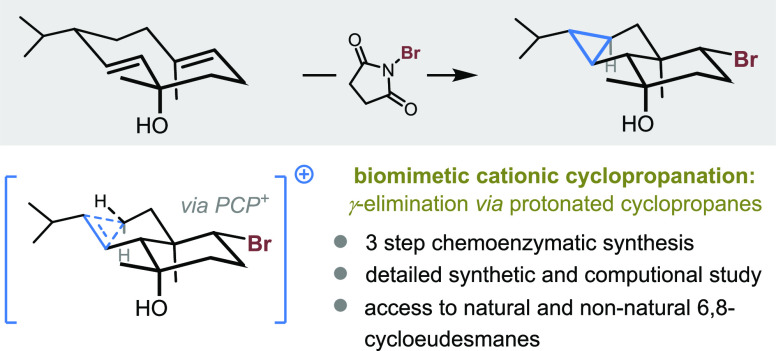

Cationic cyclopropanation involves the γ-elimination at carbocations to form a new σ-C–C bond through proton loss. While exceedingly rare in bulk solution, it is recognized as one of the main biosynthetic cyclopropanation pathways. Despite the rich history of bioinspired synthetic chemistry, cationic cyclopropanation has not been appropriated for the synthetic toolbox, likely due to the preference of carbocations to undergo competing E1 β-elimination pathways. Here, we present an in-depth synthetic and computational study of cationic cyclopropanation, focusing on the 6,8-cycloeudesmanes as a platform for this investigation. We were able to apply biomimetic cationic cyclopropanation to the synthesis of several 6,8-cycloeudesmanes and non-natural analogues—in doing so, we showcase the power of this transformation in the preparation of complex cyclopropanes.

## Introduction

Nature is a continued source of inspiration for synthetic chemistry. The biosynthesis of secondary metabolites has been optimized over millennia by evolution, and the characterization of these pathways has illuminated powerful approaches to organic synthesis. Many biochemical reactions have been appropriated directly for the “synthetic toolbox” (e.g., the Pictet–Spengler reaction^1^), and equally, great effort has been devoted to coaxing biosynthetic mechanisms into enabling “bioinspired” transformations not originally found in nature. Important examples of the latter include enamine, iminium, and *N*-heterocyclic carbene catalysis.^2^

**Figure 1:**
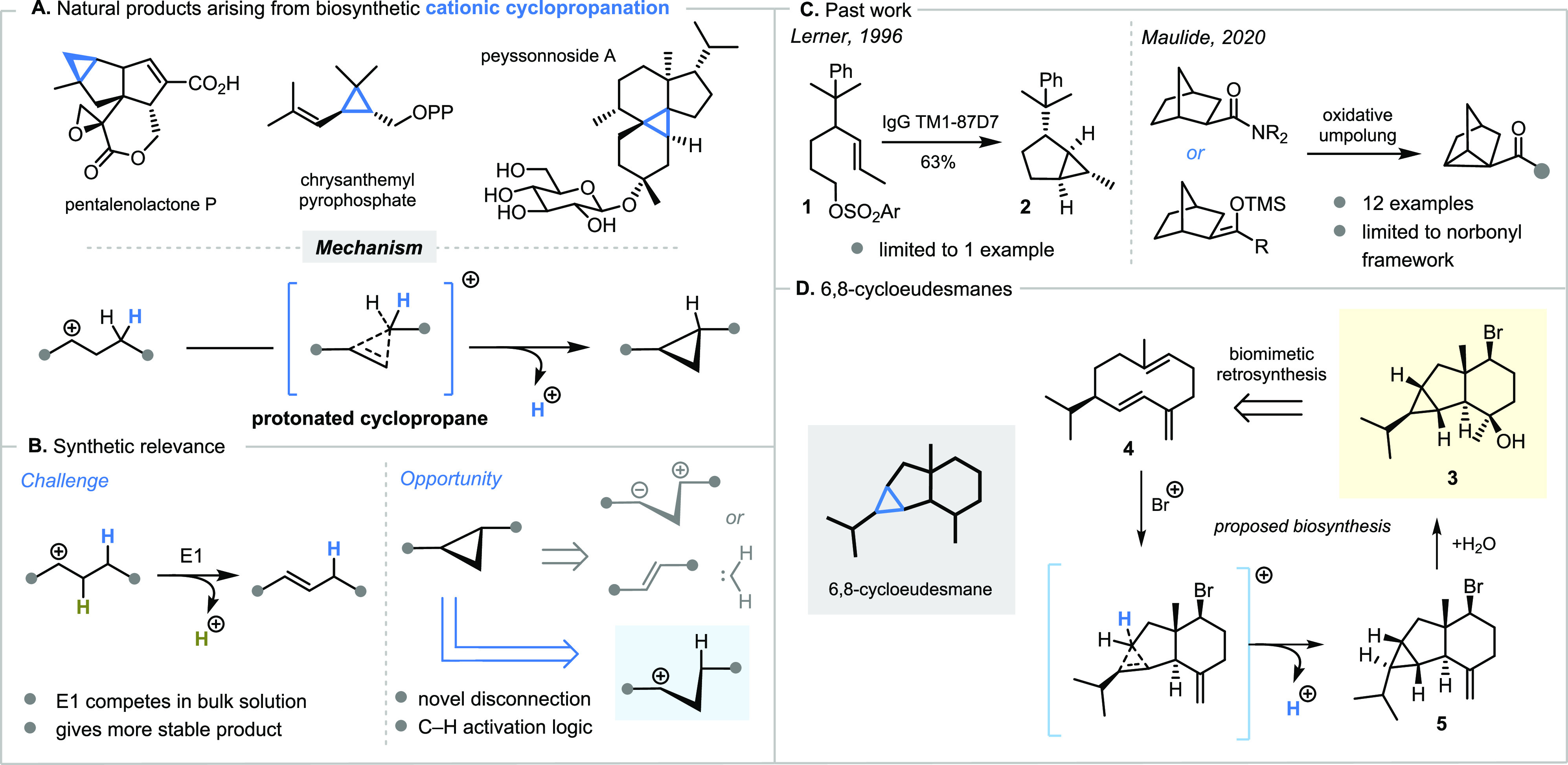
Cationic cyclopropanation in nature (A), organic synthesis (B, C), and the proposed study investigating a biomimetic approach to 6,8-cycloeudesmane **3** and analogues (D). NB: the protonated cyclopropanes depicted in this scheme are shown as corner-protonated structures, but other forms are known to exist, including alkyl-eclipsed structures which fall awkwardly between classical and non-classical designations.^3c^

Cationic cyclopropanation refers to the loss of a proton from a carbocation leading to the formation of a cyclopropane.^3^ While seldomly observed in non-enzymatic contexts, it is considered one of the main pathways in the biosynthesis of cyclopropane-containing metabolites (Figure 1A).^4^ Cationic cyclopropanation can be understood by comparison to the E1 elimination of a carbocation, where instead of producing an alkene by β-elimination, a new C–C σ-bond is formed as an alternative γ-elimination pathway takes over. The mechanism of this remarkable process involves transient formation of a protonated cyclopropane (PCP^+^), which activates the remote γ-C–H bond toward elimination.^3b,3d,5−7^ The paucity of examples of cationic cyclopropanation in synthetic contexts is likely due to an overwhelming preference for E1 elimination in bulk solution, leading to a more stable product while avoiding the intermediacy of a strained PCP^+^.^3d^ Still, the prevalence of cationic cyclopropanation in biochemical pathways is compelling and suggests that the tendency of carbocations to follow E1 pathways should not be insurmountable.

We were intrigued by this prevalence of biosynthetic cationic cyclopropanation in nature, particularly in contrast to its absence from the repertoire of organic synthesis. Cationic cyclopropanation offers a retrosynthetic disconnection for cyclopropanes, which is fundamentally different from canonical approaches involving carbenes/carbenoid reagents or intramolecular nucleophilic substitution (Figure 1B).^8−10^ Such a disconnection is inherently attractive as it would involve the activation of an otherwise inert C–H bond. To the best of our knowledge, efforts to harness synthetic cationic cyclopropanation have generally been limited to the nortricyclane scaffold. Originating from norbornane/ene-derived starting materials, cationic intermediates resulting therefrom classically exhibit a unique geometric propensity toward γ-elimination.^11^ A notable exception was reported by Lerner and co-workers in 1996, where antibody catalysis was applied to the conversion of **1** to **2**. While impressive, this work was confined to a single example and falls short of the generality required for application in organic synthesis (Figure 1C).^12^ A recent example of nortricyclane synthesis by cationic cyclopropanation from our lab, relying on an oxidative α-carbonyl umpolung approach,^11f^ represented an improvement in synthetic practicality compared with Lerner’s approach but was not generalizable beyond the norbornane skeleton. These examples underscore the challenges associated with harnessing cationic cyclopropanation and highlight the need for a deeper understanding of the phenomenon to allow its widespread application in target-oriented synthesis.

With these considerations in mind, we were drawn to investigate 6,8-cycloeudesmane sesquiterpenes, proposed to arise biosynthetically from a cationic cyclopropanation event (Figure 1D).^13−19^ Bromide **3** is an archetypal example of marine origin; in the isolation report, Pietra and co-workers proposed a brominative transannular cyclization terminated by cationic cyclopropanation for establishing the carbon framework, leading to **3** after hydration of the exocyclic double bond.^14^ We chose to investigate a biomimetic total synthesis of bromide **3** and related natural products as a platform for investigating cationic cyclopropanation experimentally and computationally. Herein, we present the results of those studies along with a proof of principle suggesting that cationic cyclopropanation can indeed be harnessed for target-oriented synthesis.

## Results and Discussion

**Scheme 1:**
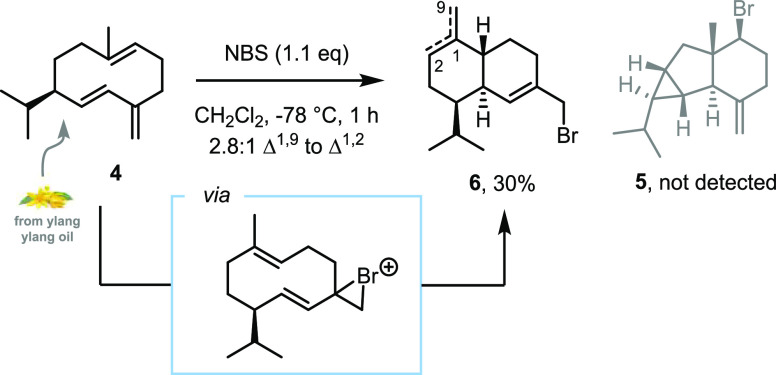
Unsuccessful Attempt at a Biomimetic Synthesis of Cyclopropane **5**

At the outset of our study, we sought to execute the biomimetic bromonium-initiated cyclization of germacrene d (**4**) to access 6,8-cycloeudesmane **3** (Scheme 1). We were encouraged in this pursuit by a report from Dickschat and co-workers, where a diterpene homologue of **3** was obtained in a stereochemical assignment effort.^20^

The most direct way to obtain germacrene d (**4**) was by purification from commercially available ylang ylang oil, and we developed a convenient protocol using Ag-impregnated silica gel (see Supporting Information for details) to separate **3** from several other hydrocarbon components. Unfortunately, treatment of germacrene d (**4**) with NBS led to the formation of **6**, among other minor products, with no detectable formation of cyclopropane **5** (Scheme 1). We surmised that preferential activation of the 1,3-dienyl π-system drove the formation of **6**; therefore, we considered reversing the order of cyclization and hydration events—instead targeting 6,8-cycloeudesmane **3** by cationic cyclization from an already hydrated precursor.

**Scheme 2:**
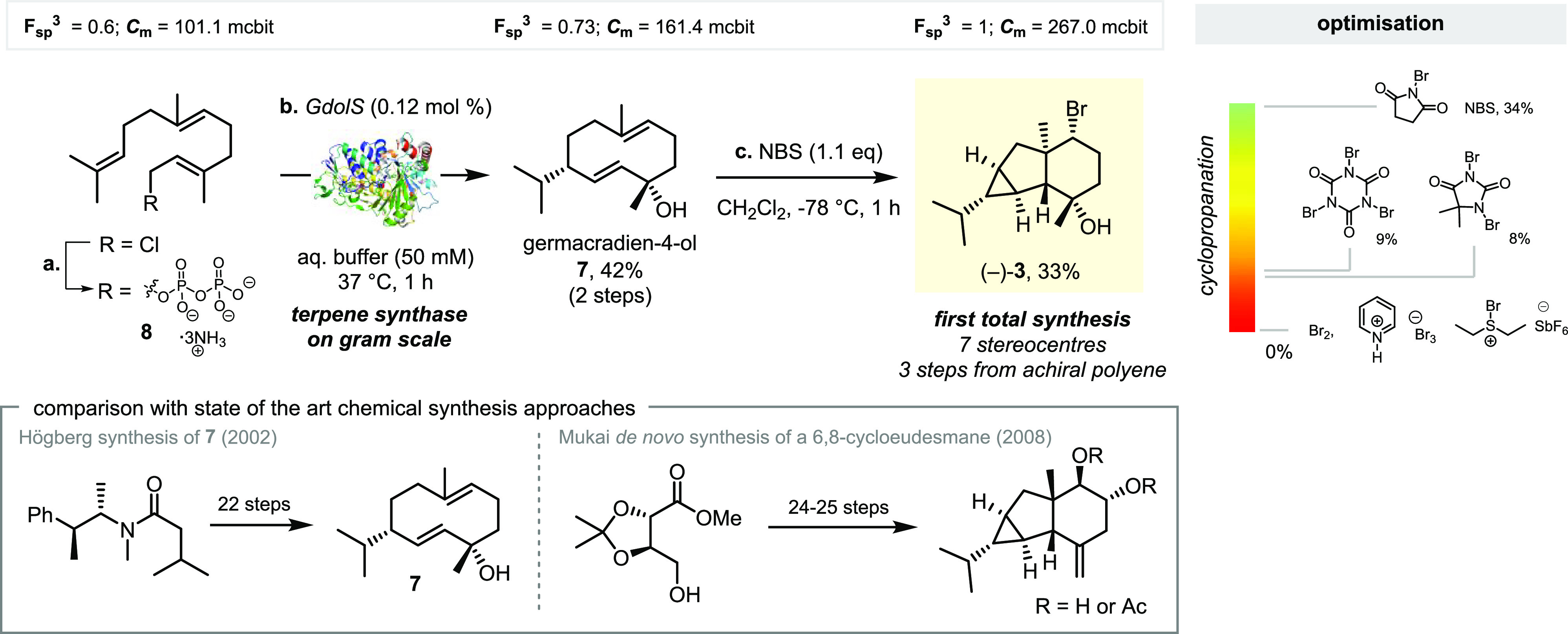
Efficient Chemoenzymatic Synthesis of **3** Optimization yields based on ^1^H NMR against an internal standard (mesitylene); *C*_m_—intrinsic complexity, see ref (27) for detailed definition; *F*_sp3_—fraction of C-atoms that are sp^3^ hybridized.

To target naturally occurring 6,8-cycloeudesmane **3** directly, it was necessary to obtain germacradien-4-ol (**7**) (Scheme 2). This task was complicated by its low abundance in natural sources (compared with germacrene d **4**) and the challenge of synthetic preparation imposed by its 10-membered ring. Indeed, the only reported synthesis of **7** required a total of 22 steps.^21^ Our search for alternative routes led us to pursue a biocatalytic synthesis of **7** using germacradiene-4-ol synthase (*GdolS*) from *Streptomyces citricolor*, which had not been previously implemented as a biocatalyst in preparative-scale synthesis.^22,23^ To our delight, the heterologous expression of *GdolS* in *E. coli* strain BL21(DE3) Rosetta II was extremely high yielding, affording an average of 49 mg of protein per liter cell culture and therefore about 300 mg of total protein in a single 6 L expression. Single-step purification by immobilized metal chelate affinity chromatography (IMAC) yielded *GdolS* in sufficient purity, and the protein stock solution could be stored for months at −80 °C without observable loss of activity.

The substrate for *GdolS*, farnesyl pyrophosphate (FPP, **8**), can be obtained commercially, but the cost is prohibitively high for preparative-scale synthesis (€58/mg at Sigma Aldrich).^24^ We therefore opted to prepare FPP in-house from farnesyl chloride (Scheme 2). Reported protocols for the synthesis of **8** proved laborious on scale due to its instability and the requirement for anion-exchange chromatography, followed by reverse-phase chromatography with two lyophilization steps.^25^ We found that the scalability could be improved by subjecting the pooled anion-exchange column volumes directly to the following *GdolS*-catalyzed cyclization. Using this protocol, we were able to conduct the *GdolS*-catalyzed cyclization on 1.35 g scale (w.r.t to FPP), affording **7** in 42% yield over two steps.

With germacradien-4-ol (**7**) in hand, an investigation of domino cationic polycyclization toward bromide **3** was undertaken. Treatment of **7** with NBS at −78 °C pleasingly led to formation of tricyclic bromide **3** in 33% isolated yield alongside separable olefin-containing side products (Scheme 2). The five newly formed stereocenters of **3** were arranged in the thermodynamically favored orientation, with small groups (H and Me) occupying the (pseudo)axial positions. Interestingly, other electrophilic bromine sources were less proficient in promoting the cascade (Scheme 2 and Supporting Information). Despite the modest yield of this individual step, the strategic implementation of cationic cyclopropanation in concert with biocatalytic preparation of germacradiene-4-ol (**7**) enabled a straightforward and protecting-group-free 3-step synthesis of **3** in 14% overall yield. Furthermore, this concise synthesis highlights the power of cationic cyclopropanation for the synthesis of complex cyclopropanes and provides support to the proposed biosynthesis of **3**.^14^ Viewed from a chemoinformatics perspective, this synthesis charts a very appealing direct path from a simple, achiral, unsaturated starting material (*F*_sp3_ = 0.6, intrinsic complexity *C*_m_ = 101.1 mcbit) to a fully saturated product **3** (containing only sp^3^-hybridized carbons), bearing seven stereocenters with an intrinsic complexity of 267.0 mcbit.^26,27^ To the best of our knowledge, this is the first synthesis of bromide **3**; it compares favorably to the only other reported *de novo* synthesis of a 6,8-cycloeudesmane natural product, which required 24 steps and had an overall yield of 1.7%.^28^ Additionally, the presented enantioselective synthesis of (−)-**3** allowed us to assign the absolute stereochemistry of the natural product to be the antipodal (+)-enantiomer—which was not previously established in the isolation report.^14^

**Scheme 3:**
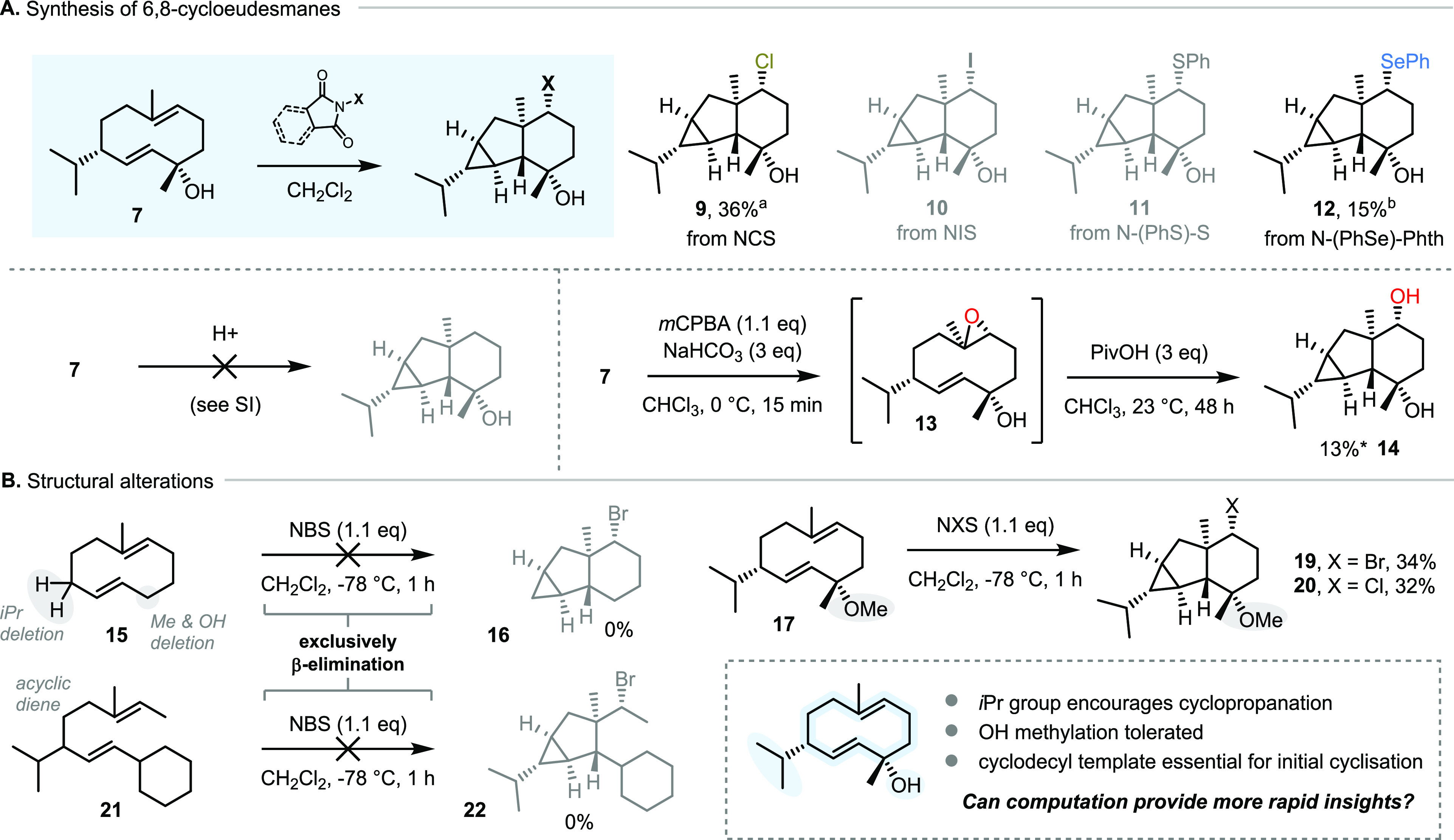
Extending the Scope of the Biomimetic Cyclopropanation (A,B) and Identification of the Factors Which Govern γ-Elimination (B) *Yield by ^1^H NMR. Reaction conditions: ^*a*^NCS (1.1 equiv), CH_2_Cl_2_, −78 °C, 1 h; *^b^N*-(phenylseleno)succinimide (3 equiv), CH_2_Cl_2_, 23 °C, 48 h.

Having demonstrated the power of biomimetic cationic cyclopropanation, we sought to gain a deeper understanding of the reaction through experimental and computational assays. These experiments were designed to extend the scope of the reaction as well as to identify the factors responsible for cationic cyclopropanation by γ-elimination. Initially, the electrophile was altered to examine the role of the halogen atom: NCS was found to perform similarly to NBS, affording **9** in 36% yield—but interestingly, NIS did not lead to the formation of detectable amounts of the desired cyclopropane (structure **10**, Scheme 3A), only β-elimination products. These investigations were extended to sulfur- and selenium-based NXS derivatives (toward **11** and **12**, respectively), whereby cyclopropane formation was observed only in the latter case, giving **12** in 15% yield. Protic acids were not competent cyclization promoters; however, formation of epoxide **13** followed by treatment with pivalic acid afforded naturally occurring 6,8-cycloeudesmane **14** in 13% yield.^19,29^ These observations underscore the decisive role of the heteroatom(X) substituent in promoting γ-elimination.

Following the investigation of electrophilic promoters for the biomimetic cyclopropanation reaction, we sought to examine the influence of structural features of the diene substrate. We found that cyclodecadiene **15**, lacking the isopropyl group and tertiary alcohol moiety of **7**, afforded exclusively β-elimination products upon treatment with NBS (structure **16**, Scheme 3B). This result suggests an important role for the steric bias imposed by the isopropyl group in determining whether loss of either the β-proton or the γ-proton takes place.^30^ Next, O-methylated derivative **17** was investigated. Its exposure to NBS and NCS afforded cyclopropane products in both cases (**19** and **20**), in nearly identical yields to the native substrate **7**—indicating that some degree of structural alteration is tolerated at that position.

Lastly, linear diene **21**, designed as an acyclic analogue of a germacrene, was subjected to the standard conditions, which led to no discernible amount of initial cyclization, showcasing the importance of the cyclodecyl template for promoting the initial cyclization event. Together, these investigations resulted in the synthesis of a range of 6,8-cycloeudesmanes—four non-natural (**9**, **12**, **19**, **20**) and two natural (**3**, **14**)—as well as providing an array of experimental observations. We initiated an in-depth computational study to aid in interpreting these observations.

**Figure 2:**
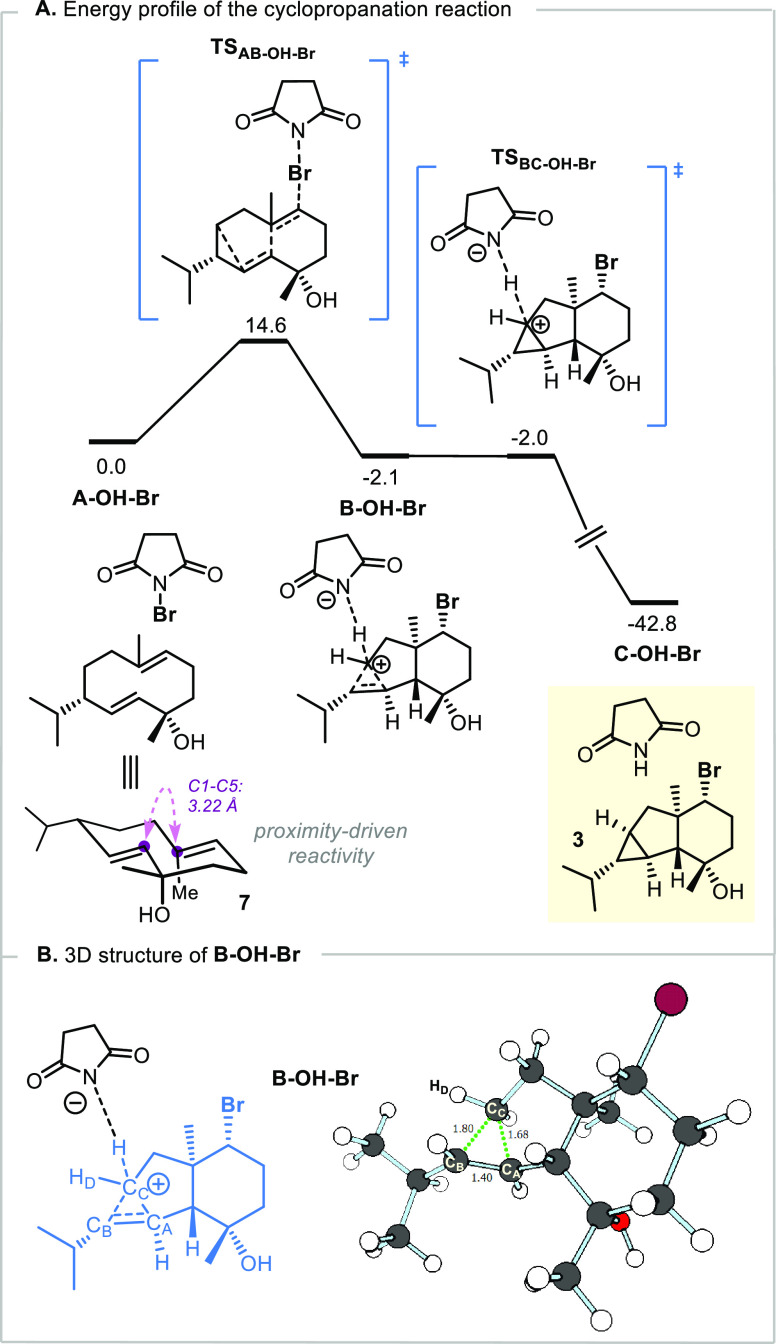
Computed Gibbs free energy profiles of the reaction of **7** with NBS (A) and 3D representation of the structure of the protonated cyclopropane intermediate with selected distances in Å (B). Relative Gibbs free energies are presented in kcal/mol (298 K). The reactant complex (**A-OH-Br**) is used as the reference (0.0 kcal/mol). The explicit solvent molecule (CH_2_Cl_2_) is not represented.

We performed quantum chemical calculations at the density functional theory (DFT) level (PBE0-D3BJ-SMD/def2-TZVP//PBE0-D3BJ-SMD/def2-SVP),^31^ including one explicit solvent molecule (dichloromethane) in the model (see Supporting Information for details). Initially, the mechanistic profile for the reaction of **7** with NBS was obtained (Figure 2A), which identified the pivotal PCP^+^ structure **B-OH-Br** and revealed an intriguing step for its formation. This step involves the following fully concerted bond reorganizations: (a) N–Br bond cleavage, (b) C–Br bond formation, (c) formation of the 6/5 ring bridgehead C–C bond, and (d) formation of the three-center two-electron C–C bond of the PCP^+^. The obtained activation barrier for this event is consistent with the reaction occurring at −78 °C (Δ*G*^‡^(**A-OH-Br → B-OH-Br**) = 14.6 kcal/mol), and the formation of the protonated cyclopropane intermediate is slightly exergonic (Δ*G*(**A-OH-Br → B-OH-Br**) = −2.1 kcal/mol). The “locked” conformation and spatial proximity of the olefin moieties within the 10-membered ring provide kinetic viability, while the alleviation of transannular strain (imposed by the 10-membered ring) through the formation of a 6-membered and a 5-membered ring is a likely thermodynamic driving force.^31^ Indeed, in structure **A-OH-Br**, prior to C–C bond formation, the two proximal sp^2^ carbons lie at a distance of only 3.22 Å. The protonated cyclopropane moiety of structure **B-OH-Br** (Figure 2B) presents three carbon (C_A_, C_B_, C_C_) and one hydrogen (H_D_) atoms in the same plane. A short C–C bond (1.40 Å, consistent with a carbon–carbon double bond) is established between C_A_ and C_B_, and both these carbon atoms present an elongated C–C bond (1.80 and 1.68 Å) to C_C_. The C–H distance between H_D_ and C_C_ (1.11 Å, not shown; covalent C–H bond) and between H_D_ and C_B_ (1.93 Å, not shown) is consistent with the corner-protonated structure obtained by Houk and co-workers for the PCP^+^ involved in the work of Lerner (Figure 1C).^3b,3c,7,12^ Subsequent deprotonation of this species by the succinimidate anion has a negligible barrier and leads to the experimentally observed product **3**. This deprotonation is extremely exergonic ((Δ*G*(**B-OH-Br→C-OH-Br**) = −40.7 kcal/mol), and formation of the cyclopropane product can therefore be considered irreversible.

**Figure 3:**
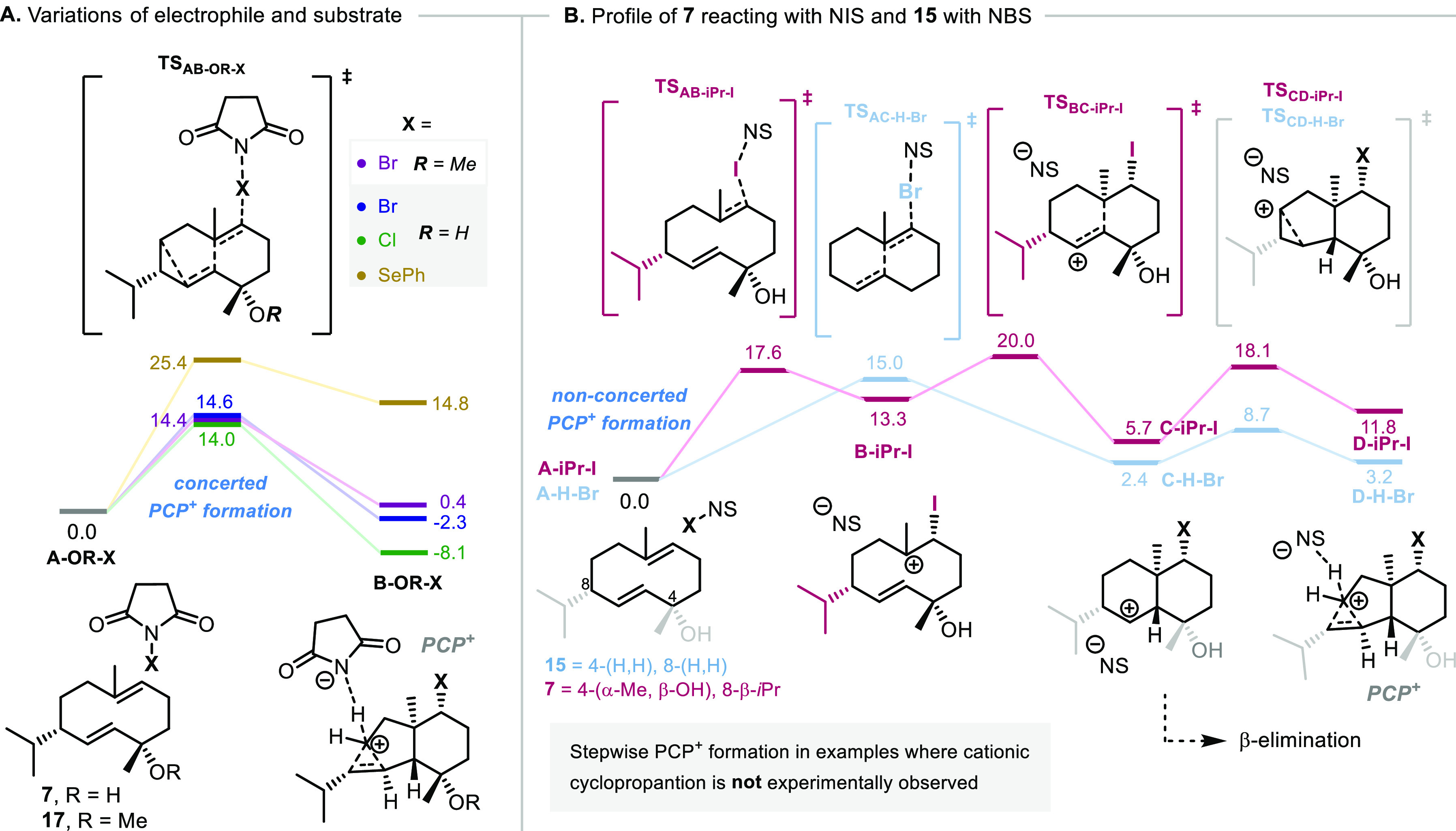
(A and B) Computed Gibbs free energy profiles with different electrophiles reveal concerted PCP^+^ formation as necessary for the cationic cyclopropanation pathway. Relative Gibbs free energies are presented in kcal/mol (298 K). The reactant complex (**A-R-X**) is used as the reference (0.0 kcal/mol) in each case. The explicit solvent molecule (CH_2_Cl_2_) is not represented.

To understand the observations when using different electrophiles, transition states for formation of the pivotal PCP^+^ intermediates in the reaction of germacradien-4-OMe **17** with NBS and the reactions of germacradien-4-ol **7** with NCS, *N*-(phenylseleno)phthalimide, and NIS were computed (Figure 3). The obtained results show a more facile cyclization promoted by NBS and NCS (Figure 3A)—regardless of the substrate (Δ*G*^‡^ = 14.0 and 14.6 kcal/mol)—than by *N*-(phenylseleno)succinimide, in which steric hindrance of the phenyl group destabilizes both the transition state and the PCP^+^ intermediate (Δ*G*^‡^(**A-OH-SePh → B-OH-SePh**) = 25.4 kcal/mol and Δ*G*(**A-OH-SePh → B-OH-SePh**) = 14.8 kcal/mol). In these four cases, the cyclopropane product was observed experimentally, and importantly, the computed profiles (Figure 3A) uniformly show a *concerted* pathway for PCP^+^ formation.

This mechanistic detail is likely crucial to enable γ-elimination since the intermediacy of one or more carbenium ions (in a *stepwise* mechanism) opens further β-elimination and rearrangement pathways. While PCP^+^ itself may also be prone to such undesired pathways, the profile for the native reaction (Figure 2) shows that γ-elimination is kinetically favorable (Δ*G*^‡^ = 0.1 kcal/mol).^3c^

We tested the emerging hypothesis of *concerted* PCP^+^ formation as a key mechanistic feature by computing the profiles of certain reactions which resulted exclusively in β-elimination (Figure 3B), especially the reaction of germacradien-4-ol **7** with NIS and the reaction of simplified cyclodecadiene **15** with NBS (cf. Scheme 3B). In considering the latter case, we hoped to also clarify the role of the isopropyl group of **7** in promoting γ-elimination through steric shielding of the β-C–H bond and its involvement in a potential pseudo-Thorpe–Ingold effect.^30^

These computational studies indeed supported our proposal, and the formation of the PCP^+^ intermediates for the reactions of **7** with NIS and **15** with NBS (Figure 3B) was both shown to follow *stepwise* pathways. The former (pink, Figure 3B) involves individual transition states for the iodination (step **A-iPr-I → B-iPr-I**), cyclization (step **B-iPr-I → C-iPr-I**), and PCP^+^ formation (step **C-iPr-I → D-iPr-I**)—which are all reversible. This stepwise mechanism is likely a consequence of the greater ability of the iodine atom to accommodate a partial positive charge compared to bromine and chlorine, thereby adding stability to the discrete secondary and tertiary carbocations through delocalization (see Supporting Information for calculated partial charges).^32^ The obtained profile for the reaction of **15** with NBS (blue, Figure 3B) presents a single transition state for the bromination and cyclization, resulting in a secondary carbocation structure (**C-H-Br**) which requires a second step to give PCP^+^ (**C-H-Br** → **D-H-Br**). This result shows that the isopropyl group acts to encourage γ-elimination by more than just steric shielding of the β-C–H bond and also through a pseudo-Thorpe–Ingold effect, which operates by stabilization of PCP^+^ relative to the secondary carbenium ion.

The apparent activation barriers for the profiles presented in Figure 3B (Δ*G*^‡^(**A-iPr-I → TS_BC-iPr-I_**) = 20.0 and Δ*G*^‡^(**A-H-Br → TS_AC-H-Br_**) = 15.0 kcal/mol) were within the range of those calculated for successful cationic cyclopropanation examples (Figure 3A), leaving the stepwise nature of these reactions as the key mechanistic difference. Importantly, the formation of PCP^+^ from the secondary carbenium ion has an activation barrier of greater than 6 kcal/mol (Δ*G*^‡^(**C-iPr-I → D-iPr-I**) = 12.4 and Δ*G*^‡^(**C-H-Br → D-H-Br**) = 6.3 kcal/mol), which is unlikely to be competitive with premature β-elimination or rearrangement.^3c^ Taken together, these results overall strongly suggest that *concerted* formation of the PCP^+^ intermediate is crucial for promoting cationic cyclopropanation in these systems.

**Figure 4:**
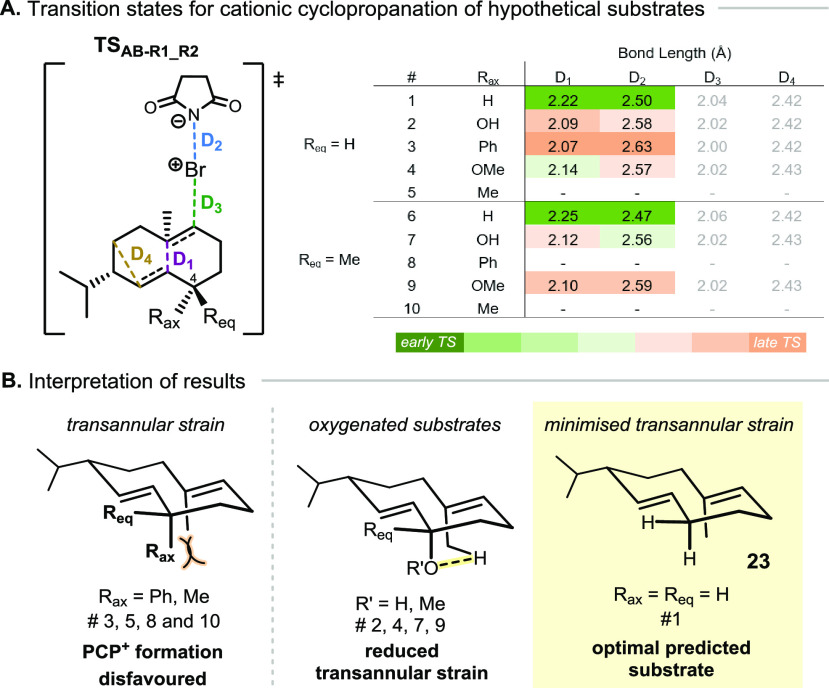
(A) Selected distances for the computed cyclization transition states (TS) with different hypothetical substrates indicating early or late TS character for cationic cyclopropanation [explicit solvent molecule (CH_2_Cl_2_) is not represented]; (B) interpretation of results based on structural features.

To further examine the influence of the substrate structure on this process, the concerted transition state for PCP^+^ formation was computed for a variety of distinct substrates with NBS. Hypothetical substrates were derived from the alteration of the C-4 geminal substituents of **7** (Figure 4), where the pseudoaxial substituent (R_ax_) was either H, OH, Ph, Me, and the pseudoequatorial substituent (R_eq_) was either Me or H. These specific structural alterations were investigated computationally because of their proximity to the initially reactive olefin and aimed to obviate the considerable synthetic efforts that their experimental investigation would entail.

From the obtained transition state structures, **TS_AB-R1_R2_**, four selected bond lengths were directly compared (Figure 4), allowing us to investigate the influence of structural elements on the relative position of the transition state: early or late on the reaction coordinate. Modulation of the steric bulk and electronic properties of the groups at R_ax_ and R_eq_ had little effect on the transition state bond lengths of the Br–C bond (D_3_, green) and the C–C bond of the protonated cyclopropane ring (D_4_, yellow). In contrast, the C–C bond joining the bridgeheads (D_1_, purple) and the N–Br bond (D_2_, blue, undergoing cleavage) show significant difference between substrates. Considering the C–C bond formation and the N–Br bond cleavage more closely, larger D_1_ and smaller D_2_ would be reflective of an early transition state structure, suggesting a more facile process. Through this lens, we were able to judge individual hypothetical substrates on their aptitude for entering the desired cationic cyclopropanation manifold.

Results reflecting a later transition state were obtained for substrates bearing a methyl or phenyl group in pseudoaxial orientation (R_ax_; Figure 4A, entries 3, 5, 8, 10), which is likely a consequence of transannular strain incurred through proximity to the allylic methyl group. In the case of R_ax_ = phenyl, a relatively late transition state for PCP^+^ formation was located when R_eq_ = H (entry 3), but where R_eq_ = Me, no concerted PCP^+^ formation was observed, and instead, a stepwise profile was obtained (entry 8, see Supporting Information for more details). Further evidence of the disruptive effect of transannular strain was encountered through our many attempts to locate a transition state for structures where R_ax_ = Me (entries 5 and 10), all of which were unsuccessful. Interestingly, the effect of transannular strain can be countered if R_ax_ contains a non-bonding electron pair able to establish an interaction with a hydrogen of the allylic methyl group—as with the native substrate **7**—stabilizing the transition state structure (entries 2, 4, 7, 9). Nevertheless, the earliest transition states and most promising results were obtained for substrates where R_ax_ = H, which experience the least transannular strain (entries 1 and 6, D_1_ > 2.22 Å and D_2_ < 2.50 Å).

**Scheme 4:**
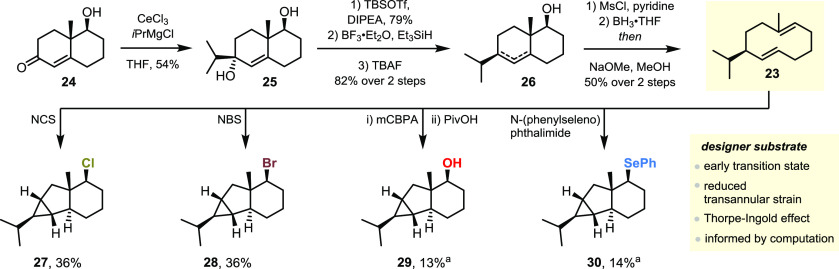
Synthesis of Cyclodecadiene **23** and Subsequent Cationic Cyclopropanations ^1^H NMR (Mesitylene).

Based on the insight gained from our computational investigation, putative cyclodecadiene **23** was predicted to undergo the desired γ-elimination pathway with the best chemoselectivity (Figure 4). To validate this prediction and demonstrate that cationic cyclopropanation can indeed succumb to rational synthetic design, we set out to synthesize **23** from the known derivative of the Wieland–Miescher ketone **24**(33) and test this hypothesis experimentally (Scheme 4). First, the isopropyl group was installed by addition of the respective organocerium reagent to the ketone of **24**, affording diol **25**.^33^ Following addition, **25** was mono-deoxygenated by hydrosilane reduction (to be executed smoothly, this transformation required protection of the secondary alcohol). Following this three-step sequence, secondary alcohol **26** was afforded as an inconsequential mixture of alkene regioisomers. Finally, a Marshall fragmentation was carried out to furnish the decadiene cyclopropanation precursor **23**.^34,35^

Gratifyingly, treatment of **23** with NCS, NBS or *N*-(phenylseleno)succinimide, or epoxidation/acidic epoxide opening all resulted in formation of the respective cyclopropane products (**27**–**30**), in yields comparable to or better than the native substrate germacradien-4-ol **7**.

## Conclusions

In summary, we report an in-depth synthetic and computational study of cationic cyclopropanation—a remarkable bioinspired reaction, which has not previously been appropriated for the repertoire of organic synthesis. Focusing on the 6,8-cycloeudesmane natural products, this study interrogated the mechanistic determinants for γ-elimination combining experiment and computation, resulting in identification of a designer substrate with improved chemoselectivity for γ-elimination. Together, these results demonstrate that cationic cyclopropanation can indeed succumb to rational design. Clearly, the deployment of this disconnection to target-oriented synthesis can result in dramatically simplified routes to complex cyclopropanes.

## Supporting Information Available

Experimental procedures and characterization data for all new compounds and computational details (PDF)
