# Supplementary material for: Biomimetic Cationic Cyclopropanation Enables an Efficient Chemoenzymatic Synthesis of 6,8-Cycloeudesmanes
Source: J Am Chem Soc. 2023 Feb 28;145(10):5855–63. doi: 10.1021/jacs.2c13116 (PMC10021018; doi:10.1021/jacs.2c13116)
Supplement: Supplementary file 1 — ja2c13116_si_001.pdf [file ja2c13116_si_001.pdf]

Biomimetic cationic cyclopropanation enables an efficient chemoenzymatic synthesis of 6,8-cycloeudesmanes

Phillip S. Grant[†a], Ricardo Meyrelles[†a,b,d], Oliver Gajsek[c,d], Gerhard Niederacher[c], Boris Maryasin[a,b], Nuno Maulide*[a]

[†]*These authors contributed equally*

[a]*Institute of Organic Chemistry, University of Vienna, 1090 Vienna, Austria;* [b]*Institute of Theoretical Chemistry, University of Vienna, 1090 Vienna, Austria;* [c]*Institute for Biological Chemistry, University of Vienna, 1090 Vienna, Austria;* [d]*Doctoral School in Chemistry, 1090 Vienna, University of Vienna, Austria*

E-Mail: nuno.maulide@univie.ac.at, Homepage: http://maulide.univie.ac.at

Contents

1. General Information S2
2. Additional synthetic schemes S3
2. Computational studies S6
  2.1. Halide effect S6
  2.2. Thorpe-Ingold effect S7
  2.3. Benchmark study for the protonated cyclopropane structure S8
  2.4. Substituent group replacement S9
  2.5. XYZ Structures S11
3. Isolation of germacrene d S29
4. Expression and purification of germacradien-4-ol synthase S29
5. Experimental Procedures S32
6. NMR spectra S48
7. References S70

1. General Information

Unless otherwise stated, all glassware was flame-dried before use and all reactions were performed under an atmosphere of argon. All solvents were distilled from appropriate drying agents prior to use or directly taken from commercial sealed bottles under an atmosphere of argon. All reagents and commercially available substrates were used as received from commercial suppliers unless otherwise stated. Experiments conducted without external heating or cooling are designated as at “room temperature” (rt), which ranged between 21 °C and 23 °C. Reaction progress was monitored by thin layer chromatography (TLC) performed on aluminium plates coated with silica gel F254 with 0.2 mm thickness. Chromatograms were visualised by fluorescence quenching with UV light at 254 nm or by staining using potassium permanganate. Flash column chromatography was performed using silica gel 60 (230-400 mesh, Merck and co.). Neat infrared spectra were recorded using a Bruker Vertex 70 FT-IR spectrometer. Wavenumbers are reported in cm^{-1}. Mass spectra were obtained using a Bruker maXis UHR-TOF spectrometer, using electrospray ionization (ESI) and by Agilent 7200B GC/Q-TOF spectrometer, using electron impact (EI). All ^{1}H NMR, ^{13}C NMR and ^{19}F NMR spectra were recorded using a Bruker AV III 400, AV NEO 500, AV III 600 or AV III HD 700 spectrometer in $CDCl_3$ or DMSO-d_6. Chemical shifts are given in parts per million (ppm, δ), referenced to the solvent residual peak of $CDCl_3$ or DMSO-d_6, defined at δ = 7.26 ppm (^{1}H NMR) and δ = 77.16 (^{13}C NMR) for $CDCl_3$, and δ = 2.52 ppm (^{1}H NMR) and δ = 39.52 (^{13}C NMR) for DMSO-d_6. Coupling constants are quoted in Hz (J). ^{1}H NMR splitting patterns were designated as singlet (s), doublet (d), triplet (t), quartet (q), pentet (p). Splitting patterns that could not be interpreted or easily visualised were designated as multiplet (m), apparent (app) or broad (br). Enantiomeric excess was measured on a Shimadzu LC-8A preparative HPLC system using Lux Cellulose-1 or Lux Cellulose-3 chiral columns.

2. Additional synthetic schemes

2.1 Investigation of **21**

The synthesis of acyclic diene **21** was achieved over three steps. Subjection of **21** to the brominative cationic cyclopropanation conditions resulted in complex mixtures from which no cyclopropanation products could be identified.

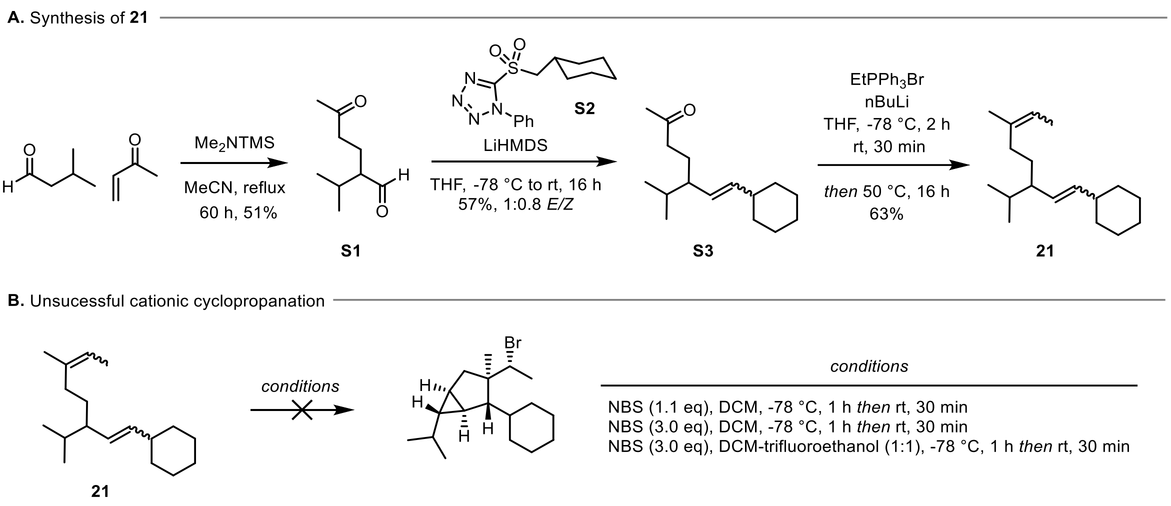

Scheme S1. Synthesis and investigation of **21**

2.2 Optimsation of cationic cyclopropanation to give **3**

Table S1.

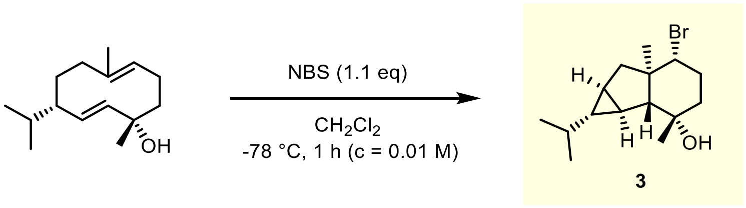

entry	Deviation from the above conditions	result
1	None	34%
2	Br_2 instead of NBS	ND
3	pyridinium tribromide (1.1 eq) instead of NBS	ND
4	1,3-dibromo-5,5-dimethylhydantoin (1.1 eq) instead of NBS	8%
5	dibromoisocyanuric acid (1.1 eq) instead of NBS	9%
6	2,4,4,6-tetrabromo-2,5-cyclohexadienone (1.1 eq) instead of NBS	3%
7	CH_2Cl_2-HFIP (9:1, *v/v*) instead of CH_2Cl_2	6%
8	CH_2Cl_2-HFIP (99:1, *v/v*) instead of CH_2Cl_2	16%
9	PhMe instead of CH_2Cl_2	12%

10	iPrOH instead of CH_2Cl_2	ND
11	THF instead of CH_2Cl_2	10%
12	Acetone instead of CH_2Cl_2	16%
13	EtOAc instead of CH_2Cl_2	6%
14	at 0 °C	27%
15	NBS (10 eq)	24%
16	c = 0.05 M	32%
17	–94 °C	28%

Yields determined by ^{1}H-NMR by comparison to an internal standard (mesitylene); experiment procedure conducted analogously to preparative procedure (see pg S27).

2.3 Selected conditions in the optimisation of epoxide-opening-cationic-cyclopropanation cascade

Table S2.

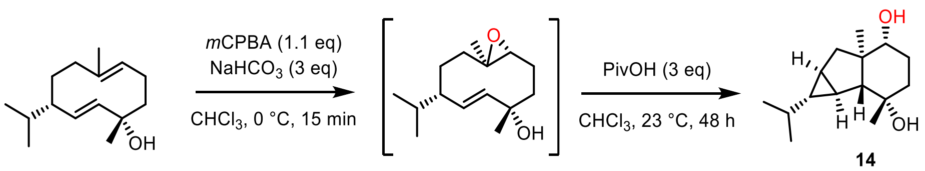

14

entry	Deviation from the above conditions (second step)	result
1	None	14%
2	AcOH (20 eq) instead of PivOH	10%
3	HFIP (10 eq) instead of PivOH	10%
4	$LiBF_4$ (1 eq) instead of PivOH	6%
5	$InBr_3$, $AlCl_3$, $ZnCl_2$, ScOTf, LiCl, or ZnOTf (1 eq) instead of PivOH	-
6	silica instead of PivOH	-
7	PPTS instead of PivOH	3%
8	1M HCl aq (1 mL) instead of PivOH	6%
9	TsOH instead of PivOH	3%
10	benzoic acid instead of PivOH	10%
11	Fluorobenzoic acid instead of PivOH	11%
12	TFA instead of PivOH	1%
13	pentafluorophenol instead of PivOH	9%
14	MeCN, DMF, or Et_2O instead of $CHCl_3$	6%
15	THF, EtOAc, dioxane, or hexane instead of $CHCl_3$	11%
16	hexafluorobenzene instead of $CHCl_3$	10%
17	HFIP instead of $CHCl_3$	2%
18	At -78 °C in CH_2Cl_2 instead of 23 °C in $CHCl_3$	10%

19	-78 °C in CH_2Cl_2 instead of 23 °C in $CHCl_3$; using TFA instead of PivOH	5%
20	-78 °C in CH_2Cl_2 instead of 23 °C in $CHCl_3$; using TsOH instead of PivOH	6%
21	At -78 °C in CH_2Cl_2 instead of 23 °C in $CHCl_3$	10%

2.4 Selected unsuccessful attempts at promoting cationic cyclopropanation

Table S3

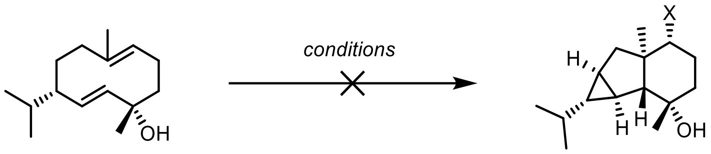

entry	X	Deviation from the above conditions	result
1	H	PPTS (0.1 eq), CH_2Cl_2, –78 °C for 1 h, then 23 °C for 16 h	no desired prd
2	H	TsOH (0.1 eq), CH_2Cl_2, –78 °C for 1 h, then 23 °C for 16 h	no desired prd
3	H	AcOH (0.1 eq), CH_2Cl_2, –78 °C for 1 h, then 23 °C for 16 h	no reaction
4	F	NFSI (1.1 eq), MeCN, 0 °C for 1 h	complex mixture
5	F	SelectFluor (1.1 eq), MeCN, 0 °C for 1 h	no desired prd
6	F	1-fluoro-2,4,6-trimethylpyridinium triflate, MeCN, 0 °C for 1 h	no desired prd.
7	SPh	N-(thiophenol)succinimide (1.1 eq), tetrahydrothiophene (1 eq), CH_2Cl_2, –78 °C for 1 h, then 23 °C for 16 h	no reaction
8	SPh	N-(thiophenol)succinimide (1.1 eq), tetrahydrothiophene (1 eq), MsOH (0.1 eq), CH_2Cl_2, –78 °C for 1 h, then 23 °C for 16 h	no reaction
9	SePh	PhSeCl (1.1 eq), CH_2Cl_2, –78 °C for 30 min, then 23 °C for 10 min	no desired prd.

Yields determined by ^{1}H-NMR by comparison to an internal standard (mesitylene)

2. Computational studies

The conformational space of all molecules has been initially searched using meta-dynamics simulations based on semiempirical tight-binding quantum chemical calculations as implemented in CREST. [1,2]

Structures located with CREST have then been subjected to PBE0-D3BJ/def2-SVP[3–9] single point calculation. Structures within a window of 6 kcal mol^{-1} to the structure with the lowest energy have been subjected to geometry optimization at the same level of theory. The nature of all stationary points (minima and transition states) was verified through the computation of the vibrational frequencies. The thermal corrections to the Gibbs free energies were combined with the single point energies calculated at the PBE0-D3BJ/def2-TZVP level of theory to yield Gibbs free energies ("G_{298}") at 298.15 K. All energies are reported in kcal mol^{-1}. The energy profiles were constructed using the most stable conformation (the global minimum) of each intermediate and transition state.

The DFT calculations have been performed with the Gaussian 16 program package.[10] The polarizable continuum model (PCM) with SMD parameters[11,12] of dichloromethane was applied to consider solvent effects for both geometries and energies. The same method was applied for the computational model benchmarks with B3LYP[13]. The RI-MP2[14,15] calculation was performed using Orca 5.0 program package[16].

The chosen level of theory has been shown to be applicable to systems containing carbocations in previous benchmark studies[17,18] and recent works[19].

Free energies in solution have been corrected to a reference state of 1 mol l^{-1} at 298.15 K through the addition of RTln(24.46) = +7.925 kJ mol^{-1} to the gas phase (1 atm) free energies.

Natural Bond Orbital (NBO) analysis was performed for selected structures at the PBE0-D3BJ-SMD/def2-TZVP//PBE0-D3BJ-SMD/def2-SVP level of theory.

2.1. Halide effect

To better understand the halide effect in the cyclization event, NBO analysis was performed on the transition state structures of the first step of the profiles for the reaction of **7** with NCS, NBS and NIS (**Figure S1**). The obtained natural charges for carbon nuclei C_a and C_b, and the halogen (Cl, Br or I) show that for the case of the reaction of **7** with NCS and NBS, the halogen moiety presents neutral partial charge, and the carbon C_b is partially positive. As a result, the tertiary carbocation that would form would be too unstable, forcing a concerted mechanism for the formation of the two C–C bonds and the Cl–C or Br–C bond. However, the reaction of **7** with NIS presents an initial transition state in which the partial charge of the Iodine is 0.46 (partially positive), and consequently C_b presents a partial charge of 0.14 (significantly lower than 0.39 with NBS and 0.5 with NCS). This strongly suggests that the positive charge is shared between C_b and the halonium moiety, stabilizing an intermediate tertiary

carbocation species. Therefore, the reaction of **7** with NIS presents the obtained stepwise energy profile.

Structure	Natural Charge		
	X	C_a	C_b
$TS_{AB-OH-Cl}$	0.02	-0.32	0.50
$TS_{AB-OH-Br}$	0.09	-0.38	0.39
$TS_{AB-iPr-I}$	0.46	-0.20	0.14

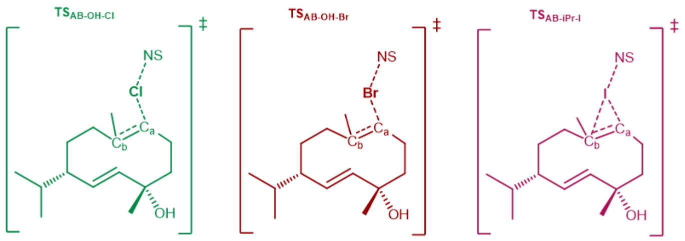

Figure S1. Natural charges of selected nuclei of the transition state structures **$TS_{AB-OH-Cl}$**, **$TS_{AB-OH-Br}$** and **$TS_{AB-iPr-I}$** (first step of the energy profile of the reaction of **7** with NCS, NBS and NIS, respectively).

2.2. Thorpe-Ingold effect

To study the Thorpe-Ingold effect exerted by the isopropyl group, energy profiles leading to the protonated cyclopropane intermediate were obtained by replacing the isopropyl with methyl and hydrogen, as shown in Figure S2. A hydrogen atom at this position provides the ring with enough conformational freedom to result in a two-step process which presents the secondary carbocation intermediate **B-H-Br**. The replacement of the isopropyl group by a methyl still allows the formation of the protonated cyclopropane to occur through a single step, which is a key property for the reaction to occur since elimination reactions from reaction intermediates can yield undesired side products.

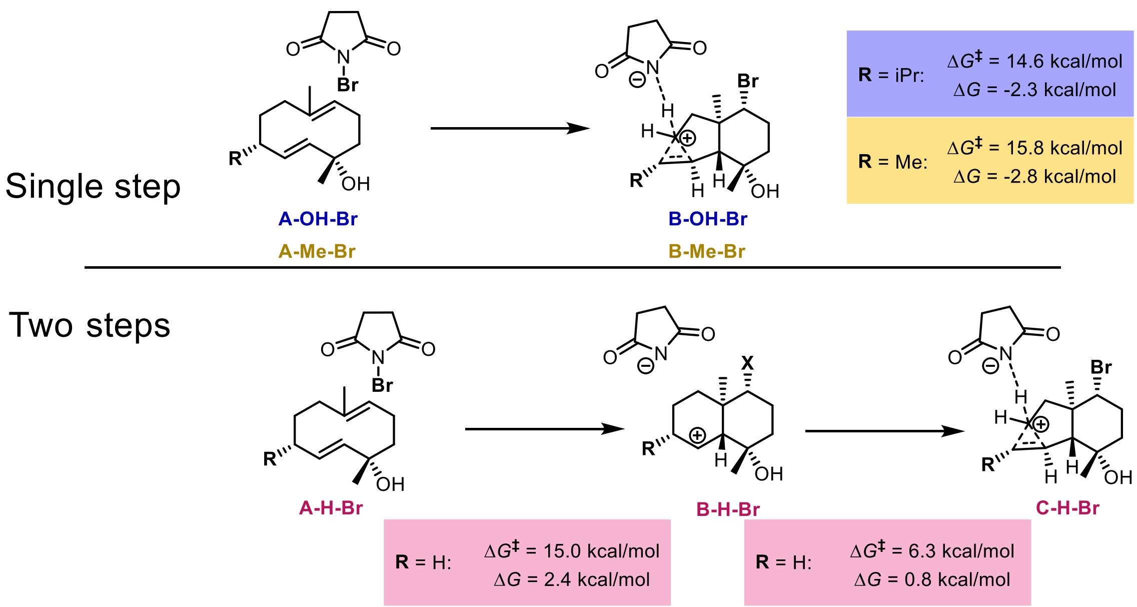

Figure S2. Kinetic ($\Delta G^{\ddagger}$) and thermodynamic (ΔG) properties (Gibbs free energies) of the computed profiles for the cyclization with different substituent groups. The reactant complexes (**A-R-Br**) serve as a reference (0.0 kcal/mol).

2.3. Benchmark study for the protonated cyclopropane structure

The protonated cyclopropane (PCP^+) structure of intermediate **B-OH-Br** was evaluated using different levels of theory to verify the structures optimized at the PBE0-D3BJ/def2-SVP,SMD(DCM) level. For this purpose, the cationic fragment of **B-OH-Br** (neglecting the counterion) was optimized at the following levels of theory: 1) PBE0-D3BJ/def2-SVP,SMD(DCM) as shown in Figure S3A, 2) PBE0-D3BJ/def2-TZVP,SMD(DCM) – Figure S3B, 3) B3LYP-D3BJ/def2-SVP,SMD(DCM) – Figure S3C, and finally, 4) RI-MP2/def2-TZVP,CPCM(DCM) – Figure S3D. Comparing the DFT- and the MP2-optimized geometries, we see that B3LYP leads to the most significant structural deviation, which presents an overly elongated C_B–C_C distance. The PCP^+ structure optimized with PBE0 presents similar distances to that obtained with MP2 and is not altered significantly with the use of a larger basis set, which justifies the use of the chosen model for computational expediency.

Additionally, Wiberg bond index analysis at the PBE0-D3BJ/def2-SVP,SMD(DCM) level of theory of this structure shows no significant interaction between $\mathbf{H_D}$ and $\mathbf{C_B}$, (bond index of 0.05, while the bond index of the covalent bond $\mathbf{C_C}$– $\mathbf{H_D}$ is 0.77). Together, these results show the structure of **B-OH-Br** to be a corner-protonated PCP^+.

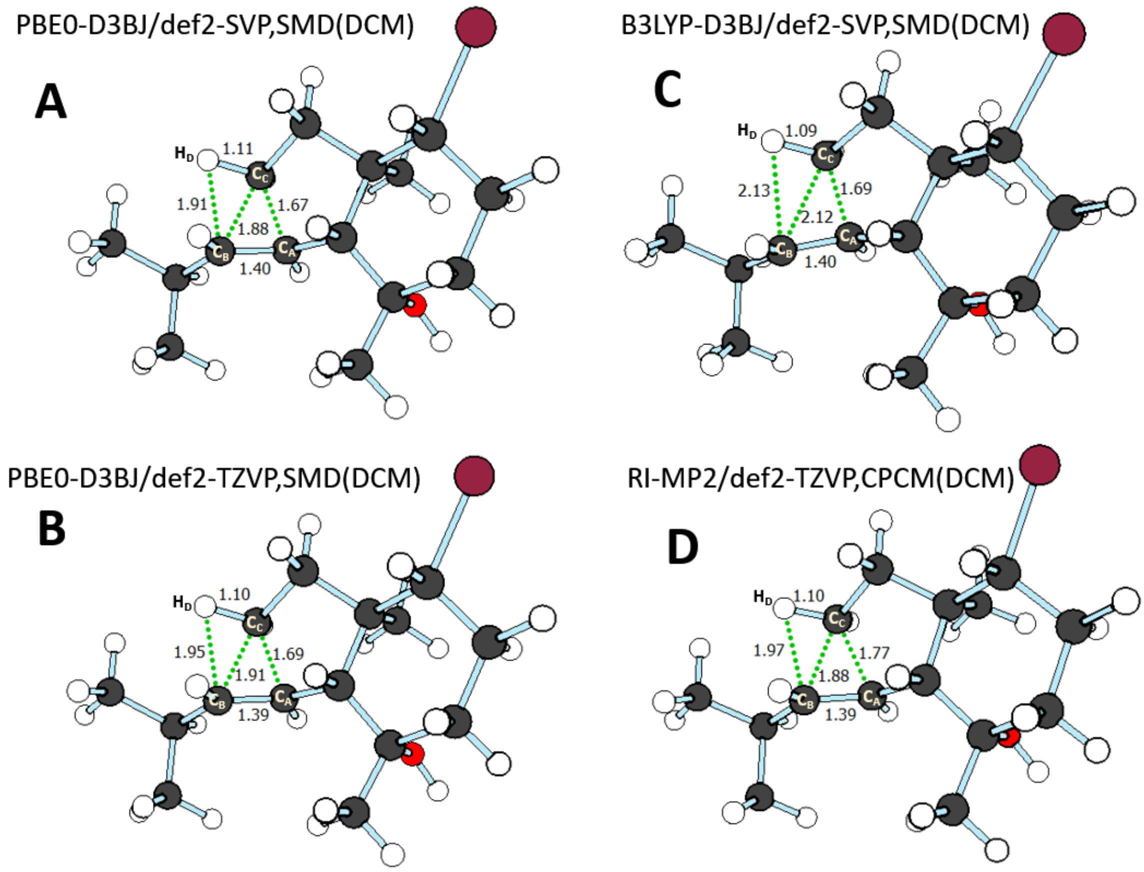

Figure S3. Optimized structures of the cationic fragment of structure **B-OH-Br** at different levels of theory with highlighted distances for comparison.

2.4. Substituent group replacement

The Gibbs free energies of the profiles obtained through substitutent group replacement are present in figure S4. All the computed systems present exergonic reactions with the exception of the substrates containing a Ph group as R_1 (entries 3 and 7), which results from the repulsion between this bulky substituent with the methyl group. The obtained activation barriers of the remaining systems are all very close to each other (approximately 14 kcal/mol), hence the obtained kinetic and thermodynamic data was not considered as a descriptor for future experiments.

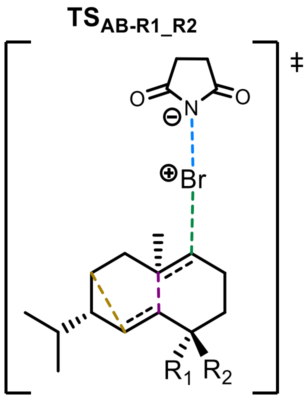

	#	R_1	$\Delta G^{\ddagger}$ (kcal/mol)	ΔG (kcal/mol)
R_2 = H	1	H	14.6	-0.5
	2	OH	14.0	-1.9
	3	Ph	16.0	4.4
	4	OMe	14.8	-2.9
R_2 = Me	5	H	13.8	-5.3
	6	OH	14.6	-2.3
	7	Ph	18.0*	7.5
	8	OMe	14.4	0.4

Figure S4. Kinetic ($\Delta G^{\ddagger}$) and thermodynamic (ΔG) properties (Gibbs free energies) of the obtained profiles for the cyclisation with different susbstituent groups. The reactant complexes (**A-R_1_R_2**) serve as a reference (0.0 kcal/mol). The profile for R_1 = Ph and R_2 = Me (*) presents two transition states, and therefore the global activation barrier is presented.

While for the system with R_1 = Ph and R_2 = H (Figure S4, entry 3) a single transition state is obtained for the cyclisation, the increase of the Thrope-Inghold effect of R_2 being replaced by a methyl group (Figure S4, entry 7), together with the steric clash with the Ph ring results in a profile with two transition states, as shown in Figure S5. The first step forms the C–Br bond, concerted with the cleavage of the Br–N bond, forming a high-energy intermediate, **B-Ph_Me**, which promptly cyclises, leading to the protonated cyclopropane structure, **C-Ph_Me**. The stabilization of a high-energy intermediate clearly shows that the steric repulsion caused by the considered susbstituent groups hinders the cyclisation process.

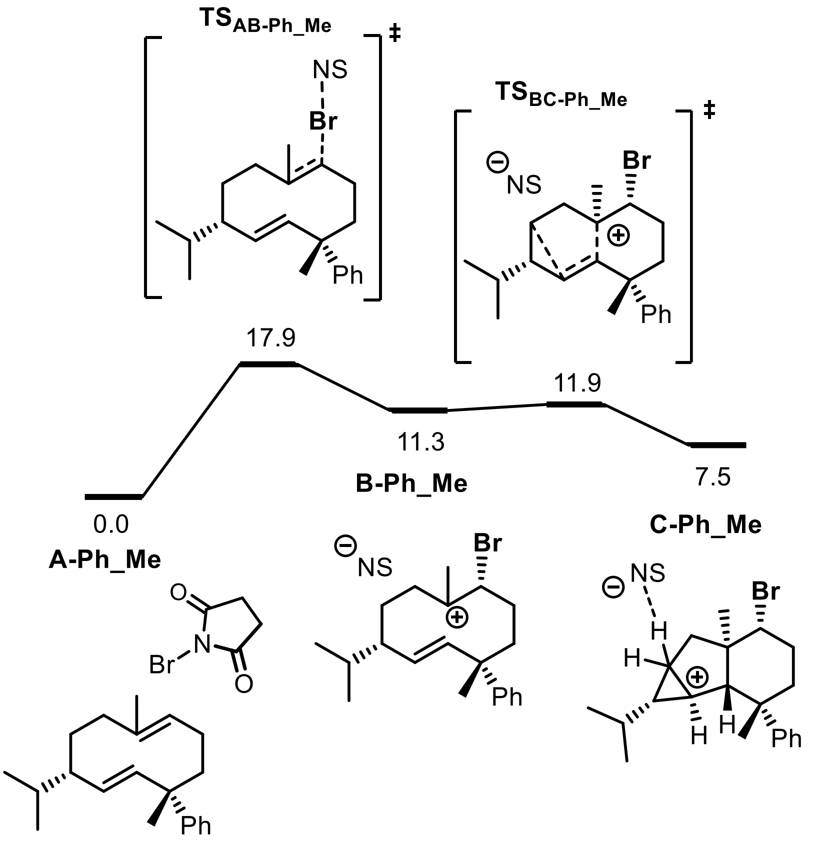

Figure S5. Energy profile for the cyclisation event with the hypothetical susbtrate containing Ph and Me substituents. Relative Gibbs free energies are presented in kcal mol^{-1}. The reactant complex (**A-Ph_Me**) serve as a reference 0.0 kcal/mol.

2.5. XYZ Structures

Cartesian coordinates of the most stable (ΔG_{298}) conformations, computed at the PBE0-D3BJ/def2-TZVP-SMD//PBE0-D3BJ/def2-SVP-SMD level of theory.

```
59
A-OH-Br
C       -3.669623000      0.638295000     -0.513200000
C       -2.385422000     -0.168188000     -0.247045000
C       -3.014032000      2.742817000      0.661075000
C       -4.075029000      1.685471000      0.542240000
H       -2.461575000     -0.657869000      0.737539000
H       -2.342678000     -0.972992000     -1.002178000
H       -3.602549000      1.132770000     -1.498348000
H       -4.484043000     -0.095679000     -0.603625000
H       -2.834309000      3.359629000     -0.228310000
H       -4.155058000      1.168620000      1.517984000
C       -0.388183000      0.852223000      0.809443000
C       -2.203101000      2.889586000      1.709356000
C        0.674403000      1.881162000      1.027508000
H       -0.726617000      0.338611000      1.718385000
C       -0.986259000      3.773761000      1.775114000
H       -2.355979000      2.263838000      2.597165000
C        0.265625000      2.920274000      2.080940000
H        0.932669000      2.388630000      0.088825000
H        1.600419000      1.399149000      1.386425000
H        0.108461000      2.418961000      3.051114000
H        1.111340000      3.614406000      2.227618000
C       -1.114860000      0.638085000     -0.312340000
C       -0.823620000      1.293938000     -1.625802000
H       -1.008914000      0.594561000     -2.456811000
H       -1.493715000      2.156689000     -1.782184000
H        0.209924000      1.652635000     -1.706137000
C       -5.456302000      2.308323000      0.231974000
H       -5.369567000      2.809913000     -0.751209000
C       -6.570062000      1.270150000      0.136219000
H       -6.637136000      0.679171000      1.065924000
H       -7.544608000      1.761791000     -0.012742000
H       -6.431260000      0.568038000     -0.698694000
C       -5.824463000      3.366878000      1.266463000
H       -5.900850000      2.920801000      2.273260000
H       -5.076016000      4.171722000      1.318784000
H       -6.797845000      3.826021000      1.031561000
N        2.125748000     -2.792861000     -0.730561000
C        3.413797000     -2.887866000     -0.219504000
C        4.030283000     -4.137053000     -0.809071000
C        2.962084000     -4.733815000     -1.717074000
C        1.767935000     -3.812217000     -1.603606000
H        4.324127000     -4.798925000      0.019270000
H        4.951024000     -3.850671000     -1.338743000
H        2.646930000     -5.745956000     -1.422894000
H        3.259679000     -4.780842000     -2.775264000
O        0.700533000     -3.912268000     -2.152317000
O        3.914464000     -2.105951000      0.547583000
Br       0.962179000     -1.378238000     -0.288904000
O       -0.847568000      4.434252000      0.529474000
H       -0.123063000      5.068430000      0.621628000
C       -1.165105000      4.814562000      2.881757000
H       -2.034273000      5.451407000      2.657876000
```

H	-1.324782000	4.337847000	3.860644000
H	-0.269879000	5.454525000	2.957599000
C	1.291408000	4.604669000	-1.897671000
H	0.387149000	4.137942000	-1.488845000
H	1.075534000	5.207407000	-2.786729000
Cl	2.438913000	3.333800000	-2.346081000
Cl	1.942247000	5.685792000	-0.643316000

59

AB-OH-Br

C	-3.570321000	0.167128000	-0.104296000
C	-2.156656000	-0.120675000	0.353001000
C	-3.020557000	2.457453000	0.477683000
C	-4.093332000	1.440123000	0.601250000
H	-2.141432000	-0.409019000	1.415844000
H	-1.743363000	-0.977152000	-0.209275000
H	-3.615291000	0.305068000	-1.196193000
H	-4.222977000	-0.683010000	0.138713000
H	-3.027245000	3.122290000	-0.393783000
H	-4.216475000	1.191398000	1.670130000
C	0.080480000	0.923113000	0.937797000
C	-1.962735000	2.553809000	1.341041000
C	0.977114000	2.144787000	0.959242000
H	-0.154770000	0.595992000	1.959846000
C	-1.001207000	3.721858000	1.362548000
H	-2.001955000	1.976062000	2.270424000
C	0.390312000	3.263369000	1.805687000
H	1.177872000	2.487245000	-0.067015000
H	1.949141000	1.851071000	1.380553000
H	0.326481000	2.932877000	2.855490000
H	1.060483000	4.137477000	1.790457000
C	-1.181483000	1.005485000	0.128988000
C	-1.025483000	1.491158000	-1.284305000
H	-1.962237000	1.446504000	-1.851864000
H	-0.636902000	2.514875000	-1.321338000
H	-0.296758000	0.822474000	-1.771044000
C	-5.432165000	1.953189000	0.046207000
H	-5.256854000	2.224968000	-1.011321000
C	-6.514856000	0.883599000	0.094259000
H	-6.667598000	0.523371000	1.125653000
H	-7.473892000	1.292717000	-0.259708000
H	-6.275399000	0.014448000	-0.535776000
C	-5.874764000	3.205396000	0.795597000
H	-6.043604000	2.984468000	1.862951000
H	-5.122141000	4.008662000	0.736980000
H	-6.815356000	3.601522000	0.382427000
N	2.191742000	-2.744534000	-0.816373000
C	3.471786000	-3.194986000	-0.783187000
C	3.572211000	-4.587025000	-1.424810000
C	2.146768000	-4.895115000	-1.839906000
C	1.364573000	-3.650340000	-1.397041000
H	3.989332000	-5.289729000	-0.686173000
H	4.286974000	-4.542547000	-2.261740000
H	1.714618000	-5.781079000	-1.348217000
H	2.011821000	-5.034720000	-2.924191000
O	0.157299000	-3.529444000	-1.563680000
O	4.437803000	-2.605905000	-0.318064000
Br	1.074495000	-0.654401000	0.159340000
O	-0.972661000	4.280692000	0.064597000
H	-0.572056000	5.159992000	0.129334000
C	-1.560788000	4.740829000	2.357232000
H	-2.540546000	5.106250000	2.015148000
H	-1.675926000	4.305796000	3.360442000
H	-0.870226000	5.596202000	2.434395000
C	2.157272000	5.404358000	-0.930358000
H	1.460334000	4.583459000	-1.132702000
H	2.582178000	5.807982000	-1.855908000
Cl	3.473119000	4.798636000	0.079837000
Cl	1.239338000	6.711200000	-0.139951000

59

B-OH-Br

C	-1.741163000	-0.348135000	1.237476000
C	-0.796440000	0.065282000	2.355703000
C	-2.073531000	1.110132000	0.460745000
C	-3.258957000	0.588750000	0.991393000
H	-1.354246000	0.323487000	3.269789000
H	-0.126472000	-0.774304000	2.593234000
H	-1.296150000	-0.849174000	0.327527000
H	-2.497465000	-1.090164000	1.556291000
H	-1.906206000	0.980909000	-0.619710000
H	-3.557315000	0.899221000	2.001151000
C	0.578111000	2.201807000	2.863799000
C	-1.243877000	2.096195000	1.247753000
C	1.044144000	3.514857000	2.254107000
H	-0.159052000	2.392383000	3.657604000
C	-0.903672000	3.415862000	0.557606000
H	-1.842037000	2.383614000	2.128617000
C	-0.109710000	4.254130000	1.575591000
H	1.853090000	3.318837000	1.535141000
H	1.473568000	4.155360000	3.038519000
H	-0.814347000	4.606832000	2.346503000
H	0.277583000	5.152299000	1.068411000
C	-0.044816000	1.288433000	1.813139000
C	0.964062000	0.829105000	0.759717000
H	0.468545000	0.300658000	-0.067183000
H	1.525447000	1.663146000	0.325914000
H	1.675299000	0.128258000	1.221639000
C	-4.293644000	-0.093809000	0.147806000
H	-3.782719000	-0.490825000	-0.744412000
C	-5.023978000	-1.204462000	0.886286000
H	-5.514316000	-0.822395000	1.796477000
H	-5.800996000	-1.642551000	0.242301000
H	-4.343434000	-2.019668000	1.180426000
C	-5.253097000	1.006528000	-0.316340000
H	-5.753273000	1.491164000	0.537725000
H	-4.726086000	1.780963000	-0.893540000
H	-6.031030000	0.570427000	-0.961009000
N	-0.725993000	-1.413887000	-1.378353000
C	0.147653000	-2.407210000	-1.706451000
C	0.191491000	-2.617407000	-3.223807000
C	-0.801553000	-1.600134000	-3.754207000
C	-1.300166000	-0.902276000	-2.489081000
H	-0.069433000	-3.663305000	-3.448813000
H	1.223515000	-2.463205000	-3.575940000
H	-1.661006000	-2.041364000	-4.282994000
H	-0.363752000	-0.848106000	-4.429118000
O	-2.124211000	0.014817000	-2.510403000
O	0.816912000	-3.054822000	-0.914581000
Br	2.059577000	1.331099000	3.825582000
O	-0.133321000	3.129424000	-0.597691000
H	-0.173428000	3.906161000	-1.172769000
C	-2.173493000	4.150558000	0.145706000
H	-2.725408000	3.573265000	-0.612025000
H	-2.837438000	4.324071000	1.005278000
H	-1.917914000	5.130486000	-0.290009000
C	2.560149000	4.895646000	-1.098644000
H	2.118777000	3.965093000	-0.722638000
H	3.354629000	4.705751000	-1.828582000
Cl	3.246807000	5.792885000	0.261648000
Cl	1.264165000	5.797770000	-1.920293000

59

BC-OH-Br

C	-1.773553000	-0.376775000	1.025284000
C	-1.080300000	0.010199000	2.322544000
C	-1.709143000	1.019187000	0.116589000
C	-3.057874000	0.717475000	0.353293000
H	-1.801471000	0.405488000	3.055197000
H	-0.605881000	-0.880914000	2.759511000
H	-1.206142000	-1.031139000	0.295367000
H	-2.694875000	-0.970798000	1.184351000
H	-1.309529000	0.776800000	-0.875065000
H	-3.532169000	1.154697000	1.241373000
C	0.388807000	2.005381000	3.058786000
C	-0.969580000	1.975714000	1.029095000
C	1.123411000	3.231441000	2.538107000
H	-0.492239000	2.315683000	3.639931000
C	-0.310705000	3.207509000	0.409195000
H	-1.729919000	2.392955000	1.709935000
C	0.244487000	4.034382000	1.579701000
H	2.047777000	2.919949000	2.028890000
H	1.422430000	3.868408000	3.383549000
H	-0.605339000	4.468244000	2.131732000
H	0.820863000	4.880495000	1.170530000
C	-0.063918000	1.089704000	1.925025000
C	1.102221000	0.435702000	1.182254000
H	1.560974000	-0.331990000	1.822280000
H	0.759543000	-0.063702000	0.264712000
H	1.868856000	1.160915000	0.890198000
C	-3.952334000	0.102448000	-0.676606000
H	-3.312713000	-0.461338000	-1.376634000
C	-5.021810000	-0.794600000	-0.073543000
H	-5.649728000	-0.241802000	0.644280000
H	-5.678372000	-1.187333000	-0.864187000
H	-4.585035000	-1.659791000	0.450669000
C	-4.564456000	1.276588000	-1.449323000
H	-5.166216000	1.924699000	-0.791673000
H	-3.787520000	1.893377000	-1.925706000
H	-5.225385000	0.890130000	-2.239807000
N	-0.332975000	-1.894494000	-1.099909000
C	0.637677000	-2.853108000	-1.052685000
C	1.001021000	-3.325236000	-2.463555000
C	-0.012470000	-2.620453000	-3.345829000
C	-0.727360000	-1.686925000	-2.371915000
H	0.973728000	-4.424298000	-2.508166000
H	2.039186000	-3.018555000	-2.670325000
H	-0.757745000	-3.300681000	-3.788936000
H	0.420839000	-2.031787000	-4.167604000
O	-1.565418000	-0.852132000	-2.730738000
O	1.168479000	-3.274177000	-0.036107000
Br	1.491956000	1.055200000	4.383764000
C	-1.317013000	4.015744000	-0.399329000
H	-0.846958000	4.939361000	-0.774817000
H	-1.668387000	3.438968000	-1.268274000
H	-2.188370000	4.300912000	0.208777000
O	0.745386000	2.782299000	-0.436318000
H	1.085930000	3.563419000	-0.895168000
C	0.346785000	1.491657000	-3.275708000
H	0.385598000	1.667920000	-2.194953000
H	-0.511618000	0.873182000	-3.561126000

Cl	1.830764000	0.627618000	-3.731924000
Cl	0.214035000	3.082705000	-4.057337000

59

C-OH-Br

C	-1.919464000	-0.107046000	1.090595000
C	-1.344424000	0.225774000	2.467905000
C	-1.602999000	1.062850000	0.178065000
C	-3.026524000	0.726149000	0.511380000
H	-2.106254000	0.690460000	3.116905000
H	-0.968023000	-0.667238000	2.987450000
H	-0.472986000	-1.049609000	-0.974074000
H	-1.836909000	-1.136346000	0.726524000
H	-1.258455000	0.920514000	-0.850094000
H	-3.531969000	1.414390000	1.203779000
C	0.138402000	2.217031000	3.269046000
C	-0.912321000	2.086824000	1.064712000
C	1.060873000	3.316093000	2.762551000
H	-0.787033000	2.655226000	3.671353000
C	-0.078895000	3.206259000	0.449415000
H	-1.712139000	2.631282000	1.597995000
C	0.417930000	4.087234000	1.608883000
H	2.015221000	2.873971000	2.438798000
H	1.294271000	4.012526000	3.581772000
H	-0.442366000	4.660272000	1.992256000
H	1.135152000	4.826243000	1.213621000
C	-0.216310000	1.232362000	2.162753000
C	0.998729000	0.450910000	1.664871000
H	1.387609000	-0.179152000	2.479147000
H	0.720059000	-0.218060000	0.839064000
H	1.802091000	1.099388000	1.299658000
C	-3.925708000	0.138361000	-0.555289000
H	-3.298144000	-0.509892000	-1.193095000
C	-5.025671000	-0.715384000	0.064391000
H	-5.677342000	-0.106886000	0.714232000
H	-5.662456000	-1.173739000	-0.709282000
H	-4.605982000	-1.527334000	0.679396000
C	-4.501521000	1.237385000	-1.441348000
H	-5.129448000	1.929636000	-0.855079000
H	-3.702445000	1.830111000	-1.914630000
H	-5.128525000	0.817808000	-2.244452000
N	-0.164712000	-1.716177000	-1.680910000
C	0.943987000	-2.522655000	-1.510148000
C	1.101169000	-3.341701000	-2.772493000
C	-0.061262000	-2.928788000	-3.668433000
C	-0.788909000	-1.848577000	-2.900765000
H	1.100925000	-4.408329000	-2.504441000
H	2.087559000	-3.116606000	-3.205251000
H	-0.771198000	-3.745545000	-3.869007000
H	0.247661000	-2.519838000	-4.640977000
O	-1.741618000	-1.195508000	-3.263237000
O	1.638491000	-2.536472000	-0.523542000
Br	0.931714000	1.337523000	4.849377000
C	-0.916442000	4.031781000	-0.519856000
H	-0.322633000	4.869543000	-0.921639000
H	-1.248226000	3.415052000	-1.367767000
H	-1.804932000	4.452119000	-0.025539000
O	1.021879000	2.637565000	-0.251106000
H	1.483120000	3.360650000	-0.699047000
C	0.557974000	1.341588000	-3.130174000
H	0.702813000	1.468267000	-2.049770000
H	-0.422137000	0.915647000	-3.372701000
Cl	1.809057000	0.237278000	-3.744831000
Cl	0.664139000	2.950928000	-3.869399000

62

A-OMe-Br

C	-3.826472000	0.607769000	-0.461793000
C	-2.519110000	-0.189785000	-0.303024000
C	-3.165648000	2.646361000	0.818722000
C	-4.205760000	1.571058000	0.679755000
H	-2.542291000	-0.739236000	0.652295000
H	-2.496367000	-0.945980000	-1.107482000
H	-3.806026000	1.171958000	-1.410998000
H	-4.631861000	-0.133320000	-0.572767000
H	-3.025888000	3.311951000	-0.041864000
H	-4.227554000	0.989181000	1.621398000
C	-0.512169000	0.834215000	0.731176000
C	-2.325867000	2.764306000	1.847835000
C	0.526203000	1.886312000	0.959082000
H	-0.801591000	0.268972000	1.626191000
C	-1.125727000	3.675965000	1.914933000
H	-2.432361000	2.092925000	2.708785000
C	0.147801000	2.821100000	2.115835000
H	0.691710000	2.478093000	0.047855000
H	1.491896000	1.416782000	1.216372000
H	0.013371000	2.233838000	3.039688000
H	0.996134000	3.496893000	2.313032000
C	-1.269915000	0.647540000	-0.374748000
C	-1.038487000	1.375748000	-1.662073000
H	-1.284613000	0.732392000	-2.521807000
H	-1.689183000	2.264036000	-1.727104000
H	-0.001781000	1.717867000	-1.772883000
C	-5.615555000	2.174510000	0.475468000
H	-5.592194000	2.731495000	-0.481060000
C	-6.707677000	1.113573000	0.378856000
H	-6.715665000	0.475576000	1.279438000
H	-7.699791000	1.586097000	0.300912000
H	-6.592630000	0.457429000	-0.496098000
C	-5.955316000	3.164099000	1.585201000
H	-5.964207000	2.662122000	2.568288000
H	-5.227551000	3.987259000	1.642348000
H	-6.952426000	3.605252000	1.428184000
N	2.016935000	-2.679084000	-1.061591000
C	3.332238000	-2.750443000	-0.621769000
C	3.949443000	-3.968174000	-1.273597000
C	2.848097000	-4.577503000	-2.132324000
C	1.639917000	-3.687937000	-1.938235000
H	4.310886000	-4.637913000	-0.479059000
H	4.828767000	-3.642471000	-1.849057000
H	2.573352000	-5.601114000	-1.837076000
H	3.088274000	-4.602273000	-3.205687000
O	0.548449000	-3.803596000	-2.434297000
O	3.852973000	-1.972801000	0.136463000
Br	0.838214000	-1.310040000	-0.518917000
O	-1.081630000	4.378002000	0.685764000
C	-1.287699000	4.629791000	3.101290000
H	-2.149067000	5.294874000	2.934276000
H	-1.466975000	4.060631000	4.025390000
H	-0.388855000	5.245495000	3.258781000
C	-0.108234000	5.368752000	0.532441000
H	-0.272730000	6.237463000	1.196224000
H	0.919085000	4.997980000	0.702304000
H	-0.169761000	5.716245000	-0.508009000
C	0.050138000	4.094223000	-3.819198000
H	-0.222759000	3.051756000	-4.016924000
H	0.345123000	4.607278000	-4.741314000
Cl	-1.368614000	4.924525000	-3.157506000
Cl	1.439456000	4.107225000	-2.718180000

59

A-OH-Cl

C	-3.177055000	0.362083000	-0.752415000
C	-2.040491000	-0.604591000	-0.371011000
C	-2.408482000	2.351854000	0.558142000
C	-3.570305000	1.440603000	0.277541000
H	-2.287069000	-1.089601000	0.587969000
H	-2.017446000	-1.403530000	-1.134018000
H	-2.940813000	0.858397000	-1.710151000
H	-4.062460000	-0.259714000	-0.951916000
H	-2.038159000	2.937461000	-0.291661000
H	-3.840644000	0.927059000	1.220432000
C	-0.091595000	0.179015000	0.930401000
C	-1.736963000	2.400722000	1.708719000
C	1.056341000	1.062441000	1.300112000
H	-0.607715000	-0.278914000	1.784274000
C	-0.433320000	3.115834000	1.949953000
H	-2.083994000	1.800198000	2.558410000
C	0.657354000	2.099407000	2.359172000
H	1.455416000	1.581935000	0.417031000
H	1.885361000	0.463758000	1.718083000
H	0.321465000	1.584210000	3.275275000
H	1.555236000	2.675838000	2.643735000
C	-0.680145000	0.035542000	-0.272784000
C	-0.130780000	0.591093000	-1.548806000
H	-0.090899000	-0.197142000	-2.319720000
H	-0.786385000	1.381160000	-1.950408000
H	0.878677000	1.007505000	-1.437846000
C	-4.808412000	2.245086000	-0.186117000
H	-4.528808000	2.746425000	-1.132676000
C	-6.024056000	1.364602000	-0.458630000
H	-6.288510000	0.772100000	0.434253000
H	-6.899070000	1.982971000	-0.714840000
H	-5.867818000	0.664406000	-1.292009000
C	-5.171158000	3.326888000	0.825828000
H	-5.434054000	2.879880000	1.800317000
H	-4.340148000	4.027165000	0.997810000
H	-6.039574000	3.911612000	0.483138000
N	1.854633000	-3.777493000	-0.388820000
C	3.166621000	-4.013867000	0.014421000
C	3.509758000	-5.405264000	-0.460138000
C	2.263686000	-5.926758000	-1.169380000
C	1.242396000	-4.818619000	-1.082991000
H	3.799722000	-6.004958000	0.415376000
H	4.391120000	-5.337467000	-1.114979000
H	1.835631000	-6.825177000	-0.700490000
H	2.428406000	-6.160696000	-2.231599000
O	0.118668000	-4.791401000	-1.509421000
O	3.850442000	-3.231912000	0.619575000
Cl	1.045789000	-2.313257000	-0.057656000
O	-0.060947000	3.777118000	0.752187000
H	0.716153000	4.319308000	0.944548000
C	-0.608989000	4.148327000	3.063514000
H	-1.358580000	4.896641000	2.764698000
H	-0.942664000	3.674598000	3.999022000
H	0.343938000	4.665473000	3.266359000
C	0.046793000	4.127318000	-2.320855000
H	0.724597000	3.652368000	-3.038489000
H	0.038344000	3.620688000	-1.348705000
Cl	0.600654000	5.789625000	-2.045909000
Cl	-1.597702000	4.062208000	-2.993086000

70
A-OH-SePh

C	-3.153526000	0.264807000	0.490944000
C	-1.732821000	-0.278668000	0.727922000
C	-2.679402000	2.686963000	0.867458000
C	-3.561062000	1.535736000	1.261328000
H	-1.585365000	-0.452636000	1.806383000
H	-1.677921000	-1.266749000	0.237610000
H	-3.312112000	0.437477000	-0.588356000
H	-3.846150000	-0.544063000	0.767448000
H	-2.729983000	3.007822000	-0.180479000
H	-3.408367000	1.340616000	2.340430000
C	0.209903000	1.221349000	1.057932000
C	-1.759041000	3.241875000	1.656243000
C	1.114685000	2.377608000	0.772923000
H	0.097206000	0.975577000	2.121465000
C	-0.698545000	4.222621000	1.231594000
H	-1.678825000	2.913012000	2.699599000
C	0.701950000	3.643780000	1.536241000
H	1.152659000	2.592786000	-0.302962000
H	2.148518000	2.136480000	1.077213000
H	0.764622000	3.456460000	2.621849000
H	1.440362000	4.435263000	1.318893000
C	-0.627560000	0.598247000	0.202288000
C	-0.639599000	0.844276000	-1.274968000
H	0.300410000	1.273132000	-1.644762000
H	-0.823372000	-0.094559000	-1.822337000
H	-1.455210000	1.536033000	-1.548253000
C	-5.056268000	1.875582000	1.058537000
H	-5.201650000	2.055914000	-0.024126000
C	-5.986618000	0.741398000	1.477568000
H	-5.816313000	0.463136000	2.531956000
H	-7.039822000	1.050812000	1.384343000
H	-5.860141000	-0.164288000	0.866856000
C	-5.433465000	3.153398000	1.800828000
H	-5.283323000	3.033401000	2.887818000
H	-4.827587000	4.013128000	1.477709000
H	-6.493056000	3.406502000	1.637354000
N	3.300384000	-2.318293000	-0.179579000
C	4.501060000	-2.378112000	0.522727000
C	5.307654000	-3.528579000	-0.036850000
C	4.452473000	-4.119982000	-1.148125000
C	3.174862000	-3.311069000	-1.146091000
H	5.511929000	-4.233632000	0.782942000
H	6.278176000	-3.140288000	-0.379468000
H	4.193652000	-5.178226000	-0.996752000
H	4.908939000	-4.037660000	-2.146162000
O	2.204660000	-3.480382000	-1.843284000
O	4.813654000	-1.636350000	1.420324000
Se	1.966828000	-1.021683000	0.157022000
O	-0.846441000	4.466660000	-0.156279000
H	-0.224909000	5.169654000	-0.390564000
C	-0.874941000	5.535737000	1.996312000
H	-1.855777000	5.975809000	1.760661000
H	-0.815152000	5.379283000	3.083973000
H	-0.088604000	6.255593000	1.712994000
C	0.952371000	4.097314000	-2.850144000
H	0.171115000	3.694734000	-2.194068000
H	0.567012000	4.334423000	-3.847920000
Cl	2.236234000	2.890092000	-3.018027000
Cl	1.540003000	5.606034000	-2.112615000
C	1.098903000	-2.050815000	1.521461000
C	1.245017000	-1.690641000	2.863609000
C	0.316349000	-3.153855000	1.165247000
C	0.592162000	-2.429043000	3.850849000
C	-0.321078000	-3.895905000	2.159204000
C	-0.189943000	-3.530229000	3.499770000
H	1.869347000	-0.836000000	3.133325000
H	0.212509000	-3.427742000	0.112879000
H	0.701921000	-2.145469000	4.900703000
H	-0.931666000	-4.759217000	1.882512000
H	-0.698247000	-4.108318000	4.275710000

62
AB-OMe-Br

C	-3.645504000	0.233054000	-0.088151000
C	-2.243420000	-0.094872000	0.377890000
C	-3.109869000	2.455389000	0.725762000
C	-4.188571000	1.437140000	0.716292000
H	-2.254558000	-0.485595000	1.407596000
H	-1.809470000	-0.888623000	-0.255887000
H	-3.661711000	0.471537000	-1.163357000
H	-4.303571000	-0.634817000	0.058289000
H	-3.086855000	3.205123000	-0.073344000
H	-4.348467000	1.089868000	1.752362000
C	-0.019888000	0.892862000	1.106618000
C	-2.078542000	2.462273000	1.627333000
C	0.864714000	2.113014000	1.273989000
H	-0.271431000	0.461875000	2.085456000
C	-1.125915000	3.628961000	1.797181000
H	-2.145838000	1.795280000	2.493103000
C	0.252383000	3.123338000	2.229892000
H	1.074089000	2.570917000	0.295355000
H	1.832345000	1.780297000	1.676598000
H	0.142950000	2.667722000	3.227536000
H	0.926194000	3.984168000	2.358059000
C	-1.272725000	1.054459000	0.289190000
C	-1.086587000	1.673055000	-1.066865000
H	-2.005835000	1.668401000	-1.663702000
H	-0.722210000	2.702619000	-0.988511000
H	-0.329792000	1.067328000	-1.591194000
C	-5.505533000	2.009857000	0.167473000
H	-5.292913000	2.378839000	-0.853104000
C	-6.592910000	0.947770000	0.078218000
H	-6.782607000	0.494060000	1.065662000
H	-7.537664000	1.394302000	-0.268849000
H	-6.334237000	0.139476000	-0.621402000
C	-5.968397000	3.188952000	1.016529000
H	-6.173395000	2.869812000	2.052238000
H	-5.211461000	3.989226000	1.058609000
H	-6.893250000	3.628474000	0.611814000
N	2.168645000	-2.537971000	-1.043111000
C	3.433482000	-3.028542000	-1.007757000
C	3.575897000	-4.232804000	-1.951282000
C	2.196432000	-4.372733000	-2.564312000
C	1.391443000	-3.245120000	-1.901498000
H	3.898919000	-5.107273000	-1.364336000
H	4.376246000	-4.023916000	-2.678668000
H	1.703078000	-5.335174000	-2.355372000
H	2.172309000	-4.232761000	-3.656774000
O	0.207757000	-3.043819000	-2.144529000
O	4.360457000	-2.601906000	-0.332367000
Br	1.000945000	-0.581058000	0.188018000
O	-1.087265000	4.285775000	0.546591000
C	-1.726047000	4.522464000	2.887033000
H	-2.670653000	4.966375000	2.538272000
H	-1.928760000	3.939679000	3.796920000
H	-1.028435000	5.329467000	3.155969000
C	-0.394785000	5.502360000	0.457756000
H	-0.866967000	6.299368000	1.058184000
H	0.663249000	5.415499000	0.759702000
H	-0.422438000	5.801017000	-0.598466000
C	0.393465000	3.729894000	-3.598279000
H	0.185310000	2.654478000	-3.619667000
H	0.804376000	4.071031000	-4.555207000
Cl	-1.133410000	4.587142000	-3.316404000
Cl	1.602130000	4.041062000	-2.340836000

59
AB-OH-Cl

C	-3.574495000	0.122913000	-0.464411000
C	-2.307822000	-0.611249000	-0.024193000
C	-2.735884000	2.214149000	0.507093000
C	-3.922181000	1.303381000	0.456408000
H	-2.435008000	-1.032796000	0.984002000
H	-2.149694000	-1.462084000	-0.714908000
H	-3.468955000	0.483874000	-1.501038000
H	-4.395114000	-0.608658000	-0.472024000
H	-2.528355000	2.808973000	-0.390066000
H	-4.096781000	0.900349000	1.471153000
C	-0.087892000	0.058955000	0.972820000
C	-1.877302000	2.280949000	1.535428000
C	0.990423000	1.093833000	1.175457000
H	-0.522990000	-0.309215000	1.909133000
C	-0.622870000	3.109860000	1.587481000
H	-2.085949000	1.703556000	2.443862000
C	0.559097000	2.250798000	2.074869000
H	1.346910000	1.471240000	0.206911000
H	1.849105000	0.595724000	1.650087000
H	0.312813000	1.865921000	3.078259000
H	1.425524000	2.920534000	2.206051000
C	-1.043900000	0.171079000	-0.089849000
C	-0.662657000	0.833845000	-1.360175000
H	-1.443271000	0.752221000	-2.125098000
H	-0.427236000	1.892749000	-1.173722000
H	0.258649000	0.359998000	-1.736957000
C	-5.195246000	2.050305000	0.010979000
H	-4.993233000	2.455844000	-0.998633000
C	-6.408398000	1.131760000	-0.080603000
H	-6.601749000	0.637163000	0.886611000
H	-7.309522000	1.707954000	-0.343375000
H	-6.289777000	0.347217000	-0.842261000
C	-5.487086000	3.222944000	0.941078000
H	-5.673454000	2.870039000	1.969820000
H	-4.648458000	3.935226000	0.982020000
H	-6.380896000	3.775350000	0.611052000
N	1.876588000	-3.444693000	-0.405338000
C	3.189452000	-3.701808000	-0.155426000
C	3.582581000	-5.029757000	-0.808406000
C	2.305128000	-5.507514000	-1.477520000
C	1.285272000	-4.411516000	-1.158926000
H	3.962150000	-5.708116000	-0.028621000
H	4.413492000	-4.846555000	-1.507251000
H	1.926637000	-6.465775000	-1.088996000
H	2.382217000	-5.612061000	-2.570881000
O	0.125010000	-4.423057000	-1.534932000
O	3.953617000	-2.999665000	0.484248000
Cl	0.740163000	-1.589638000	0.231553000
O	-0.380189000	3.613636000	0.287102000
H	0.418199000	4.159247000	0.326502000

C	-0.837660000	4.272095000	2.558669000
H	-1.659719000	4.910073000	2.201637000
H	-1.084419000	3.913605000	3.569141000
H	0.078872000	4.881227000	2.626805000
C	2.087568000	3.543107000	-2.040892000
H	2.032325000	2.489956000	-1.745950000
H	1.105390000	3.931106000	-2.331277000
Cl	2.659539000	4.482532000	-0.638405000
Cl	3.177995000	3.679647000	-3.424410000

70
AB-OH-SePh

C	-3.260575000	0.154006000	0.646067000
C	-1.811313000	-0.040380000	1.033962000
C	-2.726545000	2.507821000	0.763981000
C	-3.773577000	1.535349000	1.135764000
H	-1.709376000	-0.145586000	2.125030000
H	-1.444923000	-0.984079000	0.596263000
H	-3.394975000	0.081726000	-0.444577000
H	-3.878669000	-0.633909000	1.098803000
H	-2.826331000	3.057518000	-0.179636000
H	-3.822834000	1.481479000	2.237991000
C	0.486472000	1.037629000	1.198799000
C	-1.548525000	2.648006000	1.464944000
C	1.353215000	2.263017000	0.953446000
H	0.370090000	0.875085000	2.279100000
C	-0.630157000	3.839034000	1.255697000
H	-1.523087000	2.242626000	2.481792000
C	0.801228000	3.489865000	1.656621000
H	1.465315000	2.451364000	-0.125124000
H	2.363158000	2.053729000	1.335606000
H	0.823706000	3.329104000	2.747083000
H	1.438103000	4.366755000	1.454698000
C	-0.871933000	1.039018000	0.543471000
C	-0.862597000	1.255080000	-0.951523000
H	-0.228978000	0.470744000	-1.393841000
H	-1.861489000	1.175837000	-1.397053000
H	-0.443794000	2.231600000	-1.213090000
C	-5.152991000	1.919417000	0.579317000
H	-5.043593000	2.001552000	-0.517972000
C	-6.204280000	0.860159000	0.880753000
H	-6.286484000	0.684461000	1.966683000
H	-7.192320000	1.187986000	0.522100000
H	-5.981716000	-0.102622000	0.397788000
C	-5.582003000	3.278372000	1.122206000
H	-5.690367000	3.245648000	2.219316000
H	-4.849562000	4.067132000	0.883667000
H	-6.550518000	3.585991000	0.698452000
N	2.939504000	-2.679328000	-0.337735000
C	4.293753000	-2.758757000	-0.236266000
C	4.859437000	-3.770583000	-1.241133000
C	3.631933000	-4.258027000	-1.986261000
C	2.482065000	-3.503929000	-1.310434000
H	5.391628000	-4.561063000	-0.688684000
H	5.605472000	-3.266394000	-1.875200000
H	3.449018000	-5.340734000	-1.902372000
H	3.632916000	-4.016819000	-3.061100000
O	1.306469000	-3.657918000	-1.633897000
O	4.994241000	-2.124699000	0.540759000
Se	1.518529000	-0.576276000	0.515719000
O	-0.711808000	4.206752000	-0.106744000
H	-0.122832000	4.963449000	-0.240478000
C	-1.157445000	4.979823000	2.127270000
H	-2.168402000	5.267528000	1.801934000
H	-1.194513000	4.697251000	3.189542000
H	-0.493068000	5.853635000	2.028914000
C	1.159888000	4.388491000	-2.859181000
H	0.124436000	4.158671000	-2.585781000
H	1.218764000	4.837719000	-3.856787000
Cl	2.091206000	2.886103000	-2.867383000
Cl	1.785837000	5.575707000	-1.691482000
C	0.830134000	-1.825741000	1.796795000
C	0.911165000	-1.564452000	3.168189000
C	0.251141000	-3.017140000	1.346034000
C	0.381306000	-2.473962000	4.084198000
C	-0.264264000	-3.927408000	2.268771000
C	-0.210938000	-3.655698000	3.637059000
H	1.392767000	-0.651303000	3.527446000
H	0.240775000	-3.232350000	0.272037000
H	0.444682000	-2.261141000	5.154725000
H	-0.714756000	-4.857970000	1.912574000
H	-0.621894000	-4.369272000	4.355955000

62
B-OMe-Br

C	-2.432763000	-0.390115000	1.649477000
C	-1.450026000	0.185023000	2.645412000
C	-2.551637000	1.566592000	0.628579000
C	-3.543053000	0.680776000	1.141871000
H	-1.986744000	0.550818000	3.536014000
H	-0.774569000	-0.617986000	2.978118000
H	-1.941502000	-0.832750000	0.772208000
H	-3.070477000	-1.161307000	2.106751000
H	-2.249462000	1.460185000	-0.427251000
H	-4.015142000	1.056222000	2.063901000
C	0.155305000	2.127861000	3.078576000
C	-1.738294000	2.366259000	1.521623000
C	0.783111000	3.392709000	2.521354000
H	-0.522135000	2.376573000	3.908476000
C	-1.191164000	3.676284000	0.923580000
H	-2.312041000	2.618178000	2.426190000
C	-0.289424000	4.325825000	1.976499000
H	1.507971000	3.136647000	1.734295000
H	1.342069000	3.901283000	3.320070000
H	-0.933899000	4.670010000	2.801404000
H	0.175878000	5.228645000	1.552221000
C	-0.621381000	1.319046000	2.032222000
C	0.281172000	0.781103000	0.923670000
H	-0.288917000	0.398910000	0.066721000
H	0.954372000	1.549746000	0.531710000
H	0.889006000	-0.041020000	1.328870000
C	-4.541247000	0.089207000	0.148730000
H	-3.950937000	-0.320776000	-0.687198000
C	-5.381400000	-1.015638000	0.772024000
H	-5.923979000	-0.650417000	1.660213000
H	-6.130217000	-1.375611000	0.049999000
H	-4.778429000	-1.885351000	1.073815000
C	-5.423203000	1.212969000	-0.388646000
H	-6.000683000	1.689683000	0.421040000
H	-4.821775000	1.995453000	-0.879972000
H	-6.139494000	0.826421000	-1.129794000
N	0.263919000	-0.056621000	-2.590683000
C	0.876640000	-0.657255000	-3.652096000
C	-0.096042000	-1.553628000	-4.425663000
C	-1.389580000	-1.418393000	-3.646831000
C	-1.018605000	-0.450316000	-2.524760000
H	0.306686000	-2.577785000	-4.460151000
H	-0.157718000	-1.198403000	-5.466201000
H	-1.751671000	-2.359403000	-3.203568000
H	-2.225849000	-0.994543000	-4.224598000
O	-1.839666000	-0.100391000	-1.664654000
O	2.050410000	-0.516198000	-3.967426000
Br	1.539487000	1.021444000	3.934651000
O	-0.493556000	3.298193000	-0.242150000
C	-2.371194000	4.590885000	0.598012000
H	-2.968956000	4.167539000	-0.225549000
H	-3.022503000	4.706635000	1.476239000
H	-2.030538000	5.593263000	0.300444000
C	0.064320000	4.310005000	-1.035875000
H	-0.695396000	5.009750000	-1.425948000
H	0.837492000	4.892043000	-0.504611000
H	0.543061000	3.815895000	-1.891412000
C	2.857018000	1.797529000	-2.146895000
H	1.874837000	1.317472000	-2.016595000
H	3.606115000	1.058037000	-2.444415000
Cl	2.725620000	2.997792000	-3.448595000
Cl	3.380403000	2.538087000	-0.613010000

59
B-OH-Cl

C	-1.810593000	-0.770873000	1.237855000
C	-0.889834000	-0.434610000	2.401237000
C	-1.952868000	0.703419000	0.428176000
C	-3.212721000	0.306040000	0.889796000
H	-1.466905000	-0.112530000	3.282471000
H	-0.316500000	-1.331242000	2.679776000
H	-1.373760000	-1.331219000	0.360351000
H	-2.654433000	-1.425383000	1.527170000
H	-1.732912000	0.541034000	-0.638669000
H	-3.541768000	0.660211000	1.875367000
C	0.654989000	1.560424000	2.971540000
C	-1.078699000	1.615757000	1.254385000
C	1.275471000	2.817965000	2.382543000
H	-0.105137000	1.830592000	3.720266000
C	-0.573403000	2.889303000	0.577892000
H	-1.694614000	1.968880000	2.098582000
C	0.240961000	3.658352000	1.634932000
H	2.099873000	2.529222000	1.713384000
H	1.720960000	3.416218000	3.191430000
H	-0.466948000	4.088843000	2.362207000
H	0.740717000	4.507270000	1.140900000
C	0.002360000	0.704433000	1.890046000
C	1.022908000	0.134351000	0.903941000
H	0.528223000	-0.406720000	0.084339000
H	1.646379000	0.909780000	0.447451000
H	1.670477000	-0.580902000	1.432223000
C	-4.253236000	-0.290478000	-0.010400000
H	-3.727300000	-0.746940000	-0.864750000
C	-5.127698000	-1.318624000	0.690221000
H	-5.635880000	-0.879904000	1.564390000
H	-5.900681000	-1.692508000	0.002429000
H	-4.545395000	-2.188723000	1.033716000
C	-5.075922000	0.886200000	-0.544463000
H	-5.581146000	1.427318000	0.271854000
H	-4.444532000	1.600181000	-1.094278000
H	-5.849288000	0.513679000	-1.232999000
N	-0.778270000	-1.983947000	-1.310066000
C	-0.007596000	-3.075397000	-1.579411000
C	0.144862000	-3.277791000	-3.090754000
C	-0.644513000	-2.127173000	-3.686515000
C	-1.182099000	-1.395212000	-2.457153000

H	-0.241451000	-4.273891000	-3.358100000
H	1.215667000	-3.271299000	-3.347438000
H	-1.489305000	-2.436239000	-4.321666000
H	-0.038688000	-1.424949000	-4.280611000
O	-1.890998000	-0.389224000	-2.534226000
O	0.501744000	-3.809251000	-0.744812000
Cl	1.896408000	0.631215000	3.905993000
O	0.227812000	2.512821000	-0.524777000
H	0.388633000	3.303801000	-1.057632000
C	-1.739067000	3.740727000	0.088807000
H	-2.298716000	3.211046000	-0.697358000
H	-2.432690000	3.990091000	0.905527000
H	-1.362521000	4.684763000	-0.338417000
C	3.245237000	3.385830000	-1.193736000
H	4.278893000	3.728542000	-1.073564000
H	2.817971000	3.036580000	-0.248544000
Cl	2.273060000	4.772894000	-1.743008000
Cl	3.220743000	2.045120000	-2.343342000

70
B-OH-SePh

C	-3.164095000	0.418742000	0.353216000
C	-1.815796000	-0.071612000	0.868086000
C	-2.959147000	2.111860000	0.391039000
C	-4.036269000	1.772384000	1.210722000
H	-1.844476000	-0.197117000	1.961091000
H	-1.600968000	-1.054807000	0.424602000
H	-3.368805000	0.323365000	-0.734644000
H	-3.999945000	-0.124259000	0.834992000
H	-3.207612000	2.486684000	-0.619253000
H	-3.841561000	1.593163000	2.276913000
C	0.522040000	1.007614000	1.257452000
C	-1.565664000	2.263051000	0.937828000
C	1.305254000	2.294508000	0.991750000
H	0.288759000	0.947955000	2.332638000
C	-0.889059000	3.609703000	0.694454000
H	-1.631904000	2.172027000	2.035120000
C	0.497512000	3.538544000	1.352773000
H	1.605673000	2.342410000	-0.066691000
H	2.235693000	2.283851000	1.580146000
H	0.358522000	3.577182000	2.445911000
H	1.062275000	4.443847000	1.073643000
C	-0.792447000	1.002320000	0.469589000
C	-0.561236000	0.916928000	-1.039439000
H	-0.144817000	-0.072940000	-1.279964000
H	-1.495929000	1.024478000	-1.607001000
H	0.138556000	1.677664000	-1.397483000
C	-5.465915000	1.924952000	0.785308000
H	-5.514578000	1.706650000	-0.293848000
C	-6.398812000	1.006362000	1.558205000
H	-6.367725000	1.227365000	2.637580000
H	-7.435917000	1.136838000	1.215441000
H	-6.134428000	-0.054475000	1.420534000
C	-5.823837000	3.404155000	0.964047000
H	-5.648583000	3.744125000	1.997510000
H	-5.241285000	4.037257000	0.278398000
H	-6.890521000	3.552459000	0.737102000
N	-4.165252000	2.577675000	-2.387748000
C	-3.943258000	1.497086000	-3.163566000
C	-4.219731000	1.788689000	-4.638896000
C	-4.617464000	3.253355000	-4.633713000
C	-4.561357000	3.633722000	-3.149424000
H	-5.009296000	1.109176000	-4.996772000
H	-3.315902000	1.564563000	-5.226600000
H	-5.629949000	3.449728000	-5.019413000
H	-3.930117000	3.905504000	-5.195421000
O	-4.840241000	4.748841000	-2.728414000
O	-3.561769000	0.393351000	-2.761987000
Se	1.709959000	-0.527290000	0.912418000
O	-0.784641000	3.807596000	-0.703605000
H	-0.413626000	4.689840000	-0.844759000
C	-1.710672000	4.741823000	1.300575000
H	-2.683855000	4.824320000	0.792799000
H	-1.887431000	4.585799000	2.375336000
H	-1.180980000	5.701010000	1.179182000
C	1.054046000	3.861525000	-3.241259000
H	0.120838000	3.467804000	-2.823529000
H	0.986221000	4.017524000	-4.323440000
Cl	2.345901000	2.691925000	-2.926988000
Cl	1.366854000	5.436443000	-2.478383000
C	0.788713000	-1.869904000	1.920583000
C	0.503796000	-1.688288000	3.279005000
C	0.427361000	-3.065854000	1.291134000
C	-0.164539000	-2.683767000	3.990409000
C	-0.222381000	-4.067504000	2.013694000
C	-0.527967000	-3.875887000	3.360966000
H	0.804402000	-0.766441000	3.783644000
H	0.643165000	-3.207183000	0.229067000
H	-0.391588000	-2.530850000	5.048823000
H	-0.501903000	-4.998641000	1.513777000
H	-1.046396000	-4.656902000	3.922821000

59
A-iPr-I

C	-3.237122000	0.301772000	-0.610331000
C	-2.044480000	-0.590328000	-0.222084000
C	-2.401345000	2.402625000	0.438354000
C	-3.575931000	1.476344000	0.326577000
H	-2.189891000	-0.971608000	0.801606000
H	-2.057586000	-1.463545000	-0.896713000
H	-3.090812000	0.694449000	-1.631511000
H	-4.113261000	-0.360763000	-0.667212000
H	-2.068894000	2.852379000	-0.503394000
H	-3.793223000	1.065638000	1.330995000
C	-0.008331000	0.387458000	0.813575000
C	-1.683963000	2.635664000	1.537724000
C	1.114103000	1.365471000	0.966998000
H	-0.439317000	0.025937000	1.755668000
C	-0.387578000	3.414323000	1.583862000
H	-1.986207000	2.176398000	2.487426000
C	0.773104000	2.479385000	1.964862000
H	1.385775000	1.809388000	-0.000503000
H	2.012548000	0.851010000	1.348450000
H	0.546195000	2.037642000	2.949232000
H	1.669189000	3.107091000	2.099898000
C	-0.701930000	0.087727000	-0.320483000
C	-0.297052000	0.561784000	-1.680410000
H	-0.465729000	-0.226058000	-2.431343000
H	-0.918869000	1.420020000	-1.984607000
H	0.754783000	0.870567000	-1.730026000
C	-4.835103000	2.237746000	-0.153993000
H	-4.601594000	2.637249000	-1.159303000
C	-6.060821000	1.337786000	-0.273301000
H	-6.282641000	0.845196000	0.689105000
H	-6.947397000	1.928443000	-0.553661000
H	-5.941786000	0.552080000	-1.033397000
C	-5.145564000	3.421797000	0.755373000
H	-5.342462000	3.084993000	1.787896000
H	-4.313916000	4.140875000	0.793927000
H	-6.039747000	3.961348000	0.405080000
N	2.236126000	-3.925872000	-0.000788000
C	3.311849000	-4.219362000	0.816613000
C	3.831436000	-5.587967000	0.423723000
C	2.916220000	-6.056086000	-0.699471000
C	1.933398000	-4.921755000	-0.910932000
H	3.812661000	-6.236002000	1.312588000
H	4.884292000	-5.483466000	0.121131000
H	2.345894000	-6.964882000	-0.454849000
H	3.438347000	-6.246933000	-1.648892000
O	1.037062000	-4.874410000	-1.719775000
O	3.743725000	-3.496148000	1.682835000
I	1.129128000	-2.117269000	0.143392000
C	-0.496684000	4.531532000	2.621544000
H	-1.308813000	5.225538000	2.349376000
H	-0.718834000	4.129099000	3.621457000
H	0.446254000	5.097708000	2.672627000
O	-0.060891000	3.971325000	0.323085000
H	-0.717972000	4.654490000	0.128351000
C	-1.232143000	4.400806000	-2.747949000
H	-0.654474000	4.932774000	-3.512059000
H	-0.583992000	3.811050000	-2.087819000
Cl	-2.364711000	3.312421000	-3.564034000
Cl	-2.064233000	5.611267000	-1.744721000

59
AB-iPr-I

C	-3.359038000	0.363444000	-0.730796000
C	-2.206015000	-0.603225000	-0.416667000
C	-2.403531000	2.355135000	0.403585000
C	-3.617835000	1.487277000	0.282834000
H	-2.316323000	-1.000592000	0.604440000
H	-2.303534000	-1.452406000	-1.109917000
H	-3.219132000	0.802866000	-1.732968000
H	-4.260078000	-0.262915000	-0.800045000
H	-2.116360000	2.892690000	-0.506431000
H	-3.817725000	1.023632000	1.267129000
C	-0.029329000	0.267953000	0.558420000
C	-1.633495000	2.483025000	1.485995000
C	1.064504000	1.299226000	0.639604000
H	-0.472890000	-0.026050000	1.515181000
C	-0.349429000	3.275971000	1.543920000
H	-1.902562000	1.953425000	2.408456000
C	0.830974000	2.319870000	1.754157000
H	1.183027000	1.813940000	-0.323375000
H	2.020501000	0.799217000	0.852457000
H	0.693438000	1.797730000	2.714792000
H	1.742287000	2.931480000	1.850970000
C	-0.820105000	-0.026265000	-0.587822000
C	-0.494324000	0.557611000	-1.924487000
H	-0.894784000	-0.068398000	-2.733749000
H	-0.983653000	1.542652000	-2.000960000
H	0.581895000	0.701789000	-2.078098000
C	-4.854707000	2.333132000	-0.103533000
H	-4.630452000	2.797385000	-1.082675000
C	-6.119972000	1.494937000	-0.253532000
H	-6.337030000	0.938972000	0.674702000
H	-6.987074000	2.141572000	-0.461447000
H	-6.055299000	0.766689000	-1.075055000
C	-5.088160000	3.455662000	0.901857000
H	-5.278246000	3.048218000	1.909773000
H	-4.223268000	4.131652000	0.974957000
H	-5.963160000	4.060783000	0.616767000

N	2.648050000	-3.807260000	0.596715000
C	3.624642000	-4.432009000	1.324780000
C	3.975051000	-5.783180000	0.689713000
C	3.059611000	-5.866602000	-0.521439000
C	2.286099000	-4.560477000	-0.448485000
H	3.809737000	-6.580272000	1.431014000
H	5.047652000	-5.793968000	0.441557000
H	2.351417000	-6.709223000	-0.496119000
H	3.588885000	-5.920997000	-1.485354000
O	1.414191000	-4.222180000	-1.261107000
O	4.152323000	-3.997083000	2.333102000
I	0.741229000	-1.848484000	-0.169811000
C	-0.410812000	4.277306000	2.696051000
H	-1.238750000	4.987771000	2.539115000
H	-0.577833000	3.770885000	3.658534000
H	0.530942000	4.844187000	2.755980000
O	-0.098111000	3.960666000	0.330549000
H	-0.773689000	4.647086000	0.235509000
C	-1.310872000	4.562209000	-2.677132000
H	-0.756321000	5.156889000	-3.411577000
H	-0.638657000	3.959219000	-2.054256000
Cl	-2.409930000	3.482321000	-3.549405000
Cl	-2.177603000	5.682769000	-1.601957000

59
B-iPr-I

C	-3.268509000	-0.001690000	-0.215072000
C	-1.972467000	-0.640405000	0.310760000
C	-2.773812000	2.321485000	0.432285000
C	-3.786892000	1.224757000	0.552120000
H	-2.086777000	-0.893022000	1.376426000
H	-1.839978000	-1.587266000	-0.237170000
H	-3.152351000	0.261023000	-1.280746000
H	-4.027976000	-0.796417000	-0.181647000
H	-2.600490000	2.713730000	-0.577121000
H	-3.881903000	0.947989000	1.619261000
C	-0.011754000	0.652138000	1.223177000
C	-1.991335000	2.757770000	1.421033000
C	0.967512000	1.789106000	1.209431000
H	-0.429105000	0.396169000	2.203677000
C	-0.821814000	3.690130000	1.247562000
H	-2.143311000	2.383826000	2.440715000
C	0.428033000	3.053853000	1.886148000
H	1.270292000	2.021482000	0.180749000
H	1.876455000	1.490963000	1.753802000
H	0.212665000	2.847483000	2.947675000
H	1.237097000	3.803978000	1.874558000
C	-0.720436000	0.175103000	0.117967000
C	-0.397160000	0.618075000	-1.270647000
H	0.682020000	0.737207000	-1.430089000
H	-0.795329000	-0.082558000	-2.016830000
H	-0.857222000	1.607070000	-1.431723000
C	-5.178822000	1.674758000	0.056285000
H	-5.075386000	1.919057000	-1.018329000
C	-6.234960000	0.583169000	0.195307000
H	-6.320442000	0.250803000	1.244127000
H	-7.222984000	0.959944000	-0.113203000
H	-6.019331000	-0.301844000	-0.420983000
C	-5.634306000	2.935709000	0.783113000
H	-5.725592000	2.751430000	1.867491000
H	-4.927812000	3.768635000	0.647901000
H	-6.619182000	3.267115000	0.417853000
N	1.542552000	-4.248637000	-0.821575000
C	2.502751000	-4.173154000	0.074223000
C	3.449749000	-5.356906000	0.088796000
C	2.883649000	-6.229352000	-1.017517000
C	1.677179000	-5.432182000	-1.520471000
H	3.435998000	-5.821530000	1.086730000
H	4.477285000	-5.004312000	-0.089138000
H	2.545200000	-7.219629000	-0.676719000
H	3.579208000	-6.395559000	-1.854235000
O	0.941222000	-5.797524000	-2.415108000
O	2.679440000	-3.218856000	0.889314000
I	1.192309000	-1.469939000	0.794912000
O	-0.631944000	3.881614000	-0.139186000
H	0.116811000	4.480993000	-0.263649000
C	-1.112250000	5.029354000	1.926127000
H	-1.990689000	5.500116000	1.459823000
H	-1.313501000	4.901959000	3.000674000
H	-0.250458000	5.708904000	1.819032000
C	2.077857000	3.592116000	-2.415029000
H	2.961179000	3.570692000	-3.062796000
H	2.002643000	2.677165000	-1.817927000
Cl	2.269749000	4.966645000	-1.299651000
Cl	0.638242000	3.728126000	-3.428052000

59
BC-iPr-I

C	-3.178000000	-0.230471000	0.137960000
C	-1.814082000	-0.410367000	0.768014000
C	-2.700074000	2.127139000	0.386595000
C	-3.782895000	1.134105000	0.556469000
H	-1.899317000	-0.528699000	1.859740000
H	-1.353988000	-1.338145000	0.385923000
H	-3.118146000	-0.281423000	-0.960471000
H	-3.850160000	-1.037530000	0.461119000
H	-2.625308000	2.667129000	-0.564627000
H	-4.025349000	1.062343000	1.631532000
C	0.373145000	0.735591000	1.360680000
C	-1.698179000	2.321755000	1.307943000
C	1.258278000	1.964843000	1.280074000
H	0.066500000	0.545838000	2.398666000
C	-0.766969000	3.518204000	1.253165000
H	-1.841069000	1.900922000	2.308708000
C	0.578727000	3.179197000	1.891418000
H	1.541522000	2.162507000	0.235970000
H	2.192273000	1.765975000	1.824668000
H	0.416507000	3.006755000	2.968009000
H	1.232678000	4.062915000	1.809427000
C	-0.828373000	0.692995000	0.460382000
C	-0.557456000	0.944909000	-1.000725000
H	0.206567000	0.219787000	-1.323957000
H	-1.447596000	0.797529000	-1.624013000
H	-0.179267000	1.958070000	-1.173039000
C	-5.047144000	1.519107000	-0.228085000
H	-4.747116000	1.618959000	-1.287695000
C	-6.124797000	0.447953000	-0.131760000
H	-6.397001000	0.256049000	0.919840000
H	-7.036345000	0.773713000	-0.656428000
H	-5.810427000	-0.506043000	-0.579778000
C	-5.577929000	2.866648000	0.249328000
H	-5.875815000	2.817268000	1.310130000
H	-4.823448000	3.664655000	0.152301000
H	-6.460725000	3.172522000	-0.333177000
N	1.314912000	-4.223895000	-0.468175000
C	2.593311000	-4.125058000	-0.090334000
C	3.408661000	-5.377623000	-0.407331000
C	2.388100000	-6.282598000	-1.069126000
C	1.102514000	-5.444277000	-1.044275000
H	3.831842000	-5.777884000	0.527342000
H	4.258645000	-5.102104000	-1.051031000
H	2.211177000	-7.229532000	-0.535615000
H	2.627065000	-6.542375000	-2.112316000
O	0.037442000	-5.842729000	-1.491210000
O	3.121688000	-3.143827000	0.465045000
I	1.594907000	-1.079111000	0.904062000
O	-0.627913000	3.873388000	-0.103996000
H	0.034004000	4.575717000	-0.176590000
C	-1.443620000	4.654193000	2.023179000
H	-2.389314000	4.929699000	1.533148000
H	-1.652890000	4.373459000	3.065887000
H	-0.781470000	5.534803000	2.031314000
C	2.077714000	3.790412000	-2.386786000
H	2.961039000	3.831979000	-3.033673000
H	2.069709000	2.873570000	-1.787321000
Cl	2.161530000	5.181437000	-1.279455000
Cl	0.632541000	3.815198000	-3.402757000

59
C-iPr-I

C	-2.265923000	-0.765433000	1.087413000
C	-0.982343000	-0.380790000	1.780481000
C	-2.364754000	1.380392000	0.258040000
C	-3.283951000	0.464761000	0.857900000
H	-1.168212000	-0.079264000	2.824216000
H	-0.355689000	-1.287692000	1.784739000
H	-2.077788000	-1.262067000	0.121010000
H	-2.853126000	-1.455003000	1.710948000
H	-2.342731000	1.496223000	-0.835060000
H	-3.555644000	0.793766000	1.875650000
C	0.917004000	1.317402000	1.897978000
C	-1.298420000	1.977583000	1.010105000
C	1.543224000	2.566564000	1.308731000
H	0.543848000	1.519994000	2.912097000
C	-0.747098000	3.304841000	0.458543000
H	-1.584383000	2.111500000	2.063125000
C	0.507464000	3.680601000	1.245760000
H	1.946365000	2.352810000	0.307440000
H	2.387133000	2.887819000	1.935590000
H	0.204581000	3.957503000	2.268641000
H	0.945619000	4.585135000	0.793529000
C	-0.221769000	0.730575000	1.056431000
C	0.235005000	0.291386000	-0.331407000
H	1.039986000	-0.447863000	-0.218262000
H	-0.569070000	-0.203600000	-0.893729000
H	0.596612000	1.137016000	-0.925559000
C	-4.501811000	0.083433000	0.015325000
H	-4.122250000	-0.233155000	-0.972189000
C	-5.285109000	-1.070342000	0.624965000
H	-5.598631000	-0.834654000	1.655864000
H	-6.195057000	-1.263727000	0.036682000
H	-4.704497000	-2.003993000	0.647900000
C	-5.388372000	1.310273000	-0.174511000
H	-5.775248000	1.670803000	0.793114000
H	-4.836065000	2.140554000	-0.645211000
H	-6.251543000	1.075555000	-0.815912000
N	-0.190141000	-3.172652000	0.079792000
C	0.900662000	-3.980051000	0.171004000
C	1.329372000	-4.501825000	-1.208225000
C	0.329084000	-3.863221000	-2.151158000
C	-0.568130000	-3.051399000	-1.210198000

```
H        1.304246000     -5.603024000     -1.200177000
H        2.374892000     -4.208236000     -1.392473000
H       -0.289869000     -4.581274000     -2.712043000
H        0.777316000     -3.182307000     -2.892106000
O       -1.509061000     -2.369570000     -1.628444000
O        1.492562000     -4.269993000      1.203637000
I        2.470798000     -0.173155000      2.282023000
O       -0.471889000      3.101757000     -0.908486000
H       -0.113518000      3.923195000     -1.274695000
C       -1.814915000      4.384223000      0.616474000
H       -2.705707000      4.128910000      0.020863000
H       -2.118022000      4.507907000      1.666551000
H       -1.424886000      5.349358000      0.255916000
C        2.848665000      4.609746000     -1.703304000
H        3.716937000      5.161877000     -2.079871000
H        2.897588000      4.486947000     -0.615774000
Cl       1.399691000      5.573473000     -2.086452000
Cl       2.824511000      3.013105000     -2.457106000

59
CD-iPr-I
C       -2.240839000     -0.751232000      1.042522000
C       -0.966895000     -0.383054000      1.761374000
C       -2.362245000      1.347739000      0.242538000
C       -3.296882000      0.474629000      0.865118000
H       -1.169806000     -0.094343000      2.805489000
H       -0.346089000     -1.293708000      1.762833000
H       -2.047485000     -1.218927000      0.062486000
H       -2.837444000     -1.459943000      1.636138000
H       -2.328576000      1.414360000     -0.854284000
H       -3.535188000      0.789532000      1.894024000
C        0.914161000      1.337539000      1.922256000
C       -1.286766000      1.954702000      0.985995000
C        1.534572000      2.592218000      1.337335000
H        0.516797000      1.541381000      2.926984000
C       -0.751592000      3.285898000      0.425554000
H       -1.584930000      2.108918000      2.033172000
C        0.482316000      3.688691000      1.233997000
H        1.968369000      2.375431000      0.349612000
H        2.355721000      2.935106000      1.982839000
H        0.153250000      3.976034000      2.245843000
H        0.917268000      4.593136000      0.778422000
C       -0.199283000      0.738163000      1.057531000
C        0.295761000      0.297843000     -0.317300000
H        1.094462000     -0.444513000     -0.181870000
H       -0.493889000     -0.193195000     -0.902519000
H        0.676769000      1.141496000     -0.901605000
C       -4.529001000      0.079882000      0.053682000
H       -4.170942000     -0.251755000     -0.936763000
C       -5.302170000     -1.061708000      0.698073000
H       -5.597436000     -0.806678000      1.729641000
H       -6.222216000     -1.264681000      0.129172000
H       -4.722882000     -1.996178000      0.727605000
C       -5.416416000      1.306744000     -0.135668000
H       -5.778144000      1.685733000      0.834528000
H       -4.873558000      2.125790000     -0.635491000
H       -6.296018000      1.061808000     -0.750333000
N       -0.191585000     -3.160540000      0.042910000
C        0.878285000     -3.995041000      0.138297000
C        1.292627000     -4.535259000     -1.238214000
C        0.312326000     -3.871575000     -2.184933000
C       -0.563718000     -3.032874000     -1.247876000
H        1.233927000     -5.635149000     -1.225107000
H        2.346676000     -4.274566000     -1.423085000
H       -0.325139000     -4.573346000     -2.745669000
H        0.781011000     -3.205120000     -2.926369000
O       -1.484863000     -2.326206000     -1.669529000
O        1.462617000     -4.294152000      1.172528000
I        2.475896000     -0.131911000      2.349211000
O       -0.448776000      3.074620000     -0.934101000
H       -0.097560000      3.898891000     -1.300723000
C       -1.837731000      4.350891000      0.553534000
H       -2.712294000      4.079122000     -0.058637000
H       -2.164462000      4.477749000      1.596131000
H       -1.454509000      5.319126000      0.193913000
C        2.864707000      4.625116000     -1.675459000
H        3.732984000      5.183070000     -2.043258000
H        2.900061000      4.508173000     -0.586748000
Cl       1.412320000      5.573871000     -2.082825000
Cl       2.865259000      3.024745000     -2.421484000

59
D-iPr-I
C       -1.661851000     -0.641184000      1.536301000
C       -0.689391000     -0.034989000      2.536987000
C       -1.917562000      0.623006000      0.445730000
C       -3.131236000      0.288298000      1.055828000
H       -1.226686000      0.445335000      3.369788000
H       -0.058345000     -0.833690000      2.955135000
H       -1.256917000     -1.365079000      0.772632000
H       -2.458366000     -1.237508000      2.020932000
H       -1.746126000      0.244792000     -0.574631000
H       -3.423832000      0.832854000      1.963038000
C        0.787387000      2.092351000      2.569637000
C       -1.049136000      1.721641000      1.009940000
C        1.303546000      3.225689000      1.696818000
H        0.074696000      2.476621000      3.313828000
C       -0.652747000      2.851994000      0.061077000
H       -1.635159000      2.211354000      1.805043000
C        0.162053000      3.854134000      0.896024000
H        2.079955000      2.846839000      1.015520000
H        1.775039000      3.996793000      2.323763000
H       -0.527505000      4.367254000      1.586288000
H        0.563838000      4.629688000      0.223300000
C        0.117876000      1.000586000      1.743071000
C        1.090558000      0.273713000      0.813395000
H        1.778103000     -0.338986000      1.416371000
H        0.560079000     -0.406224000      0.131446000
H        1.671867000      0.965133000      0.195116000
C       -4.188076000     -0.524887000      0.370401000
H       -3.687032000     -1.143160000     -0.391913000
C       -4.980612000     -1.397607000      1.330965000
H       -5.458581000     -0.791471000      2.117668000
H       -5.773069000     -1.936403000      0.790566000
H       -4.344132000     -2.153084000      1.819064000
C       -5.086287000      0.477996000     -0.360333000
H       -5.570373000      1.172954000      0.344759000
H       -4.515219000      1.068948000     -1.091860000
H       -5.878480000     -0.061604000     -0.900908000
N       -0.753915000     -2.346754000     -0.775941000
C        0.009417000     -3.472371000     -0.862959000
C        0.061377000     -3.988656000     -2.305033000
C       -0.803778000     -3.003187000     -3.068158000
C       -1.248875000     -2.016528000     -1.988877000
H       -0.306891000     -5.026169000     -2.328499000
H        1.111134000     -4.015000000     -2.636980000
H       -1.695265000     -3.451342000     -3.534422000
H       -0.272911000     -2.451886000     -3.859951000
O       -1.970959000     -1.045726000     -2.227170000
O        0.584393000     -4.015008000      0.069553000
I        2.388595000      1.327901000      3.851447000
O        0.118857000      2.296248000     -0.981824000
H        0.387568000      3.013397000     -1.572413000
C       -1.888797000      3.529930000     -0.520539000
H       -2.449383000      2.825160000     -1.153452000
H       -2.559299000      3.904731000      0.267476000
H       -1.588855000      4.384625000     -1.148398000
C        3.159955000      4.365744000     -1.521505000
H        3.986288000      5.014647000     -1.832150000
H        2.828807000      4.605204000     -0.505233000
Cl       1.800703000      4.664817000     -2.632137000
Cl       3.717643000      2.690419000     -1.556871000

46
A-H-Br
C       -3.657475000      0.790453000     -0.452391000
C       -2.399922000     -0.066829000     -0.225332000
C       -2.914147000      2.882037000      0.758628000
C       -3.978496000      1.836992000      0.627178000
H       -2.474876000     -0.573255000      0.751023000
H       -2.391120000     -0.857121000     -0.996859000
H       -3.589278000      1.290805000     -1.433714000
H       -4.517673000      0.105086000     -0.528598000
H       -2.708975000      3.482687000     -0.139136000
H       -4.118336000      1.331430000      1.598204000
C       -0.364376000      0.891823000      0.823623000
C       -2.101358000      3.006912000      1.811768000
C        0.740978000      1.877069000      1.040908000
H       -0.707284000      0.377497000      1.730700000
C       -0.832569000      3.798315000      1.854876000
H       -2.297489000      2.377386000      2.691873000
C        0.395447000      2.914326000      2.121923000
H        1.004186000      2.390261000      0.104569000
H        1.653408000      1.347872000      1.368303000
H        0.250954000      2.391456000      3.084499000
H        1.272888000      3.567068000      2.258380000
C       -1.107846000      0.708504000     -0.292812000
C       -0.812614000      1.375314000     -1.600895000
H       -1.035561000      0.697869000     -2.440711000
H       -1.453697000      2.264175000     -1.734800000
H        0.231721000      1.698908000     -1.692348000
H       -4.947764000      2.298282000      0.365659000
N        2.019734000     -2.818475000     -0.776838000
C        3.387550000     -2.810380000     -0.533586000
C        3.930903000     -4.130658000     -1.032958000
C        2.728946000     -4.889099000     -1.582448000
C        1.541227000     -3.974268000     -1.380899000
H        4.426896000     -4.638874000     -0.192701000
H        4.703232000     -3.921877000     -1.788124000
H        2.527051000     -5.834972000     -1.057988000
H        2.806591000     -5.122051000     -2.654862000
O        0.391331000     -4.177259000     -1.675472000
O        3.994470000     -1.906016000     -0.019836000
Br       0.903602000     -1.372923000     -0.320697000
H       -0.699281000      4.352644000      0.910959000
H       -0.877050000      4.553806000      2.660227000
C        1.100855000      4.802345000     -1.695601000
H        0.190357000      4.259344000     -1.417372000
H        0.917058000      5.481892000     -2.535250000
Cl       2.321215000      3.618243000     -2.199158000
Cl       1.626410000      5.765956000     -0.305338000
```

```
46
AC-H-Br
C       -3.203431000     0.609941000    -0.560082000
C       -1.946649000     0.082616000     0.101629000
C       -2.603521000     2.707876000     0.508810000
C       -3.736852000     1.789186000     0.277047000
H       -2.182428000    -0.381375000     1.072453000
H       -1.492463000    -0.708649000    -0.522003000
H       -2.997635000     0.939745000    -1.589744000
H       -3.966243000    -0.179827000    -0.623869000
H       -2.372928000     3.451329000    -0.263180000
H       -4.125834000     1.408094000     1.233217000
C        0.169106000     0.757267000     1.332241000
C       -1.751057000     2.598087000     1.574890000
C        1.167046000     1.826735000     1.736839000
H       -0.324454000     0.328132000     2.214901000
C       -0.669483000     3.576900000     1.899389000
H       -2.037536000     1.905118000     2.376097000
C        0.529316000     2.926886000     2.575790000
H        1.651721000     2.250072000     0.843019000
H        1.963862000     1.340557000     2.318723000
H        0.208620000     2.503028000     3.543142000
H        1.281290000     3.697696000     2.804781000
C       -0.855595000     1.102313000     0.292416000
C       -0.337583000     1.786016000    -0.938031000
H       -1.114397000     1.964264000    -1.689940000
H        0.167360000     2.731021000    -0.706337000
H        0.418871000     1.114494000    -1.377496000
H       -4.547489000     2.300521000    -0.262877000
N        2.197443000    -2.885888000    -0.564538000
C        3.350254000    -3.573266000    -0.359568000
C        3.417847000    -4.801306000    -1.279810000
C        2.124610000    -4.732022000    -2.068285000
C        1.433072000    -3.477125000    -1.517482000
H        3.518604000    -5.705108000    -0.658224000
H        4.328066000    -4.735174000    -1.896494000
H        1.459771000    -5.598396000    -1.924649000
H        2.264520000    -4.613952000    -3.154542000
O        0.338790000    -3.093762000    -1.912429000
O        4.234921000    -3.288792000     0.435716000
Br       1.145453000    -0.825339000     0.528774000
H       -0.372745000     4.128780000     0.993841000
H       -1.113729000     4.321635000     2.583300000
C        0.502093000     5.180889000    -2.262298000
H        0.485000000     4.192899000    -2.736050000
H        0.835884000     5.951418000    -2.966486000
Cl       1.649596000     5.135164000    -0.911089000
Cl      -1.145473000     5.584730000    -1.746374000

46
C-H-Br
C       -1.117419000    -0.797058000     0.066769000
C       -0.109471000    -0.295513000     1.083827000
C       -2.154243000     1.338437000     0.199026000
C       -2.492454000    -0.081477000     0.228165000
H       -0.439456000    -0.569477000     2.100491000
H        0.859138000    -0.790203000     0.916701000
H       -0.751030000    -0.653810000    -0.960080000
H       -1.302832000    -1.873944000     0.193405000
H       -2.172223000     1.875347000    -0.791822000
H       -2.918513000    -0.380578000     1.196907000
C        0.929314000     1.722639000     2.218233000
C       -1.346422000     1.908522000     1.273019000
C        1.052911000     3.231905000     2.315929000
H        0.488131000     1.317937000     3.140838000
C       -1.255304000     3.429863000     1.310982000
H       -1.672874000     1.510794000     2.250803000
C       -0.324467000     3.876057000     2.426020000
H        1.586639000     3.618488000     1.433501000
H        1.662872000     3.486762000     3.195033000
H       -0.772501000     3.605242000     3.398049000
H       -0.220728000     4.972027000     2.421318000
C        0.092806000     1.223520000     1.024609000
C        0.695085000     1.647716000    -0.314779000
H        0.091682000     1.307339000    -1.167144000
H        0.790248000     2.736952000    -0.409098000
H        1.698801000     1.210763000    -0.418405000
H       -3.158480000    -0.347987000    -0.602921000
N       -2.370429000     2.698016000    -2.383377000
C       -3.196285000     2.049031000    -3.241632000
C       -3.457268000     2.889704000    -4.495498000
C       -2.654897000     4.154733000    -4.246791000
C       -2.016564000     3.911652000    -2.874779000
H       -4.541243000     3.056567000    -4.596692000
H       -3.138480000     2.320723000    -5.382976000
H       -3.262553000     5.072249000    -4.202455000
H       -1.858751000     4.335777000    -4.985979000
O       -1.287355000     4.724103000    -2.315791000
O       -3.682877000     0.938328000    -3.055119000
Br       2.737682000     0.932658000     2.198460000
H       -0.932010000     3.814530000     0.330443000
H       -2.261065000     3.843578000     1.478030000
C       -4.740278000     2.337257000     2.179612000
H       -3.863995000     2.717156000     2.717760000
H       -5.611650000     2.985281000     2.330916000
Cl      -5.115104000     0.713862000     2.747581000
Cl      -4.374435000     2.392358000     0.430712000

46
CD-H-Br
C       -1.674050000    -1.297765000     0.769076000
C       -1.421148000    -0.366109000     1.934392000
C       -1.318121000     0.134269000    -0.674354000
C       -2.500046000    -0.605754000    -0.469498000
H       -2.370579000     0.075050000     2.274868000
H       -1.011067000    -0.957543000     2.767849000
H       -0.753150000    -1.785981000     0.415602000
H       -2.378400000    -2.117042000     0.990520000
H       -0.545368000    -0.269904000    -1.342238000
H       -3.353455000    -0.070264000    -0.037788000
C       -0.481440000     1.972046000     2.442776000
C       -1.024769000     1.293271000     0.161523000
C        0.352935000     3.131524000     1.921835000
H       -1.530693000     2.274342000     2.569227000
C       -0.134241000     2.364126000    -0.455866000
H       -1.993738000     1.740833000     0.435720000
C       -0.055834000     3.543274000     0.505957000
H        1.416867000     2.849866000     1.926874000
H        0.242487000     3.988311000     2.603399000
H       -1.036561000     4.048200000     0.535167000
H        0.687613000     4.260829000     0.124556000
C       -0.434405000     0.737959000     1.531430000
C        0.981665000     0.188384000     1.384999000
H        1.022111000    -0.678119000     0.709747000
H        1.701184000     0.926356000     1.002715000
H        1.338968000    -0.146659000     2.370270000
H       -2.752179000    -1.383632000    -1.195908000
N        3.389370000     2.416412000    -0.115494000
C        4.424069000     1.539391000    -0.165065000
C        5.692047000     2.195066000    -0.732534000
C        5.254879000     3.620107000    -1.008697000
C        3.779090000     3.626824000    -0.584978000
H        6.012450000     1.640081000    -1.629139000
H        6.506117000     2.105049000     0.004751000
H        5.327408000     3.920503000    -2.066300000
H        5.798270000     4.380281000    -0.424660000
O        3.074915000     4.630159000    -0.677405000
O        4.386044000     0.364662000     0.187701000
Br       0.066556000     1.526102000     4.281803000
H        0.872824000     1.966738000    -0.655986000
H       -0.562627000     2.688594000    -1.416351000
C       -4.072772000     3.650557000     1.505918000
H       -4.824263000     4.399770000     1.778795000
H       -3.076030000     3.942651000     1.856729000
Cl      -4.029344000     3.523820000    -0.261112000
Cl      -4.515613000     2.119455000     2.285865000

46
D-H-Br
C       -1.448171000    -0.892819000     1.235912000
C       -0.635413000    -0.413277000     2.425575000
C       -1.853738000     0.616157000     0.414927000
C       -2.915469000    -0.108792000     0.966325000
H       -1.282595000    -0.228259000     3.297749000
H        0.093851000    -1.188511000     2.704093000
H       -0.857572000    -1.146689000     0.298966000
H       -1.975758000    -1.855111000     1.350756000
H       -1.601136000     0.529765000    -0.658245000
H       -3.330141000     0.177821000     1.937260000
C        0.482906000     1.838408000     3.074604000
C       -1.128034000     1.625051000     1.261086000
C        0.960663000     3.175756000     2.520762000
H       -0.358980000     1.990758000     3.766468000
C       -0.677959000     2.901384000     0.570194000
H       -1.823102000     1.912093000     2.070008000
C       -0.100786000     3.834988000     1.632404000
H        1.889374000     3.021657000     1.950066000
H        1.212201000     3.846288000     3.356508000
H       -0.926955000     4.191303000     2.272107000
H        0.333902000     4.727918000     1.154912000
C        0.046762000     0.882228000     1.963729000
C        1.196230000     0.547864000     1.014559000
H        0.864464000    -0.094682000     0.187061000
H        1.657561000     1.438086000     0.569914000
H        1.977812000     0.003570000     1.565045000
H       -3.542549000    -0.729177000     0.321879000
N       -0.130683000    -1.542236000    -1.405632000
C        0.726053000    -2.583330000    -1.634561000
C        1.497996000    -2.377809000    -2.941765000
C        1.038785000    -1.010258000    -3.414232000
C       -0.019695000    -0.626187000    -2.387777000
H        1.243358000    -3.194644000    -3.636227000
H        2.577364000    -2.451999000    -2.740652000
H        0.601392000    -0.990876000    -4.424315000
H        1.832281000    -0.245873000    -3.390392000
O       -0.672974000     0.423983000    -2.454655000
O        0.867517000    -3.553670000    -0.906054000
Br       1.870575000     1.044629000     4.225477000
H        0.064963000     2.674742000    -0.208763000
```

H	-1.526468000	3.387100000	0.064247000
C	0.991574000	2.917711000	-3.189925000
H	0.290281000	2.080742000	-3.047435000
H	1.323883000	3.009014000	-4.230425000
Cl	2.426676000	2.596687000	-2.189425000
Cl	0.179573000	4.429715000	-2.736647000

55
A-H_H

C	-3.402896000	0.429710000	-0.402882000
C	-2.115750000	-0.098945000	0.195917000
C	-2.645042000	2.566496000	0.436704000
C	-3.845651000	1.694771000	0.362609000
H	-2.285022000	-0.459749000	1.222653000
H	-1.758860000	-0.969192000	-0.384662000
H	-3.274372000	0.669563000	-1.470736000
H	-4.183452000	-0.341747000	-0.340453000
H	-2.452389000	3.218219000	-0.423145000
H	-4.125398000	1.384334000	1.384928000
C	0.117861000	0.587258000	1.179663000
C	-1.727785000	2.540660000	1.446321000
C	1.176163000	1.649167000	1.412172000
H	-0.311330000	0.239776000	2.129128000
C	-0.578215000	3.485341000	1.579857000
H	-1.962810000	1.931400000	2.328161000
C	0.645584000	2.833906000	2.210052000
H	1.592771000	1.984010000	0.448889000
H	2.007002000	1.183107000	1.962245000
H	0.385352000	2.496028000	3.228214000
H	1.442110000	3.585312000	2.324234000
C	-0.964288000	0.867387000	0.196977000
C	-0.545405000	1.444014000	-1.120693000
H	-1.378829000	1.537897000	-1.825597000
H	-0.049183000	2.417447000	-1.019124000
H	0.193660000	0.747486000	-1.551208000
C	-5.034537000	2.426607000	-0.284238000
H	-4.696406000	2.762374000	-1.282383000
H	-0.341691000	3.934196000	0.601774000
H	-0.921324000	4.314936000	2.223377000
N	1.996203000	-3.191094000	-0.506821000
C	3.264732000	-3.673322000	-0.447646000
C	3.384194000	-4.965036000	-1.268388000
C	1.991167000	-5.164988000	-1.834101000
C	1.199175000	-3.974091000	-1.278955000
H	3.721705000	-5.776907000	-0.604946000
H	4.166609000	-4.830437000	-2.031770000
H	1.502884000	-6.098740000	-1.513452000
H	1.943922000	-5.141512000	-2.934323000
O	0.014464000	-3.786954000	-1.520859000
O	4.202047000	-3.178797000	0.161300000
Br	0.998173000	-1.097997000	0.428264000
C	-6.238662000	1.512878000	-0.468395000
H	-6.031184000	0.676295000	-1.151676000
H	-6.563980000	1.089612000	0.496977000
H	-7.087600000	2.076128000	-0.886408000
C	-5.411694000	3.659680000	0.530224000
H	-6.237278000	4.210396000	0.052782000
H	-5.742612000	3.374103000	1.542994000
H	-4.565703000	4.356446000	0.642188000
C	-3.275300000	5.899217000	-1.848132000
H	-4.291854000	5.537183000	-1.658632000
H	-3.284967000	6.809220000	-2.458725000
Cl	-2.520206000	6.285617000	-0.291667000
Cl	-2.393739000	4.646946000	-2.743783000

58
A-Me_H

C	0.143530000	0.608267000	1.325610000
Br	1.116076000	-0.997742000	0.489690000
N	2.204734000	-2.966078000	-0.535397000
C	-1.771510000	2.494430000	1.667784000
C	1.150571000	1.685737000	1.675919000
C	-3.304886000	0.467856000	-0.399486000
C	-2.026773000	-0.073584000	0.208909000
C	-2.642144000	2.564993000	0.622760000
C	-3.810214000	1.664953000	0.432415000
H	-2.223152000	-0.510029000	1.200639000
H	-1.629653000	-0.893079000	-0.418286000
H	-3.144385000	0.783126000	-1.443158000
H	-4.065577000	-0.325418000	-0.420936000
H	-2.437085000	3.292351000	-0.171945000
H	-4.121959000	1.275693000	1.418004000
H	-0.309445000	0.176081000	2.227888000
C	-0.666158000	3.476138000	1.923870000
H	-2.018187000	1.815239000	2.494222000
C	0.550765000	2.789187000	2.538832000
H	1.590879000	2.107106000	0.758567000
H	1.977710000	1.210059000	2.223342000
H	0.259537000	2.369360000	3.518499000
H	1.322531000	3.548669000	2.742650000
C	-0.897469000	0.910480000	0.316669000
C	-0.453743000	1.593956000	-0.938649000
H	-1.271267000	1.743946000	-1.652327000
H	0.042369000	2.552628000	-0.744354000
H	0.295154000	0.933966000	-1.408618000

C	-4.994843000	2.407944000	-0.209485000
H	-4.632961000	2.813024000	-1.173280000
C	-6.169232000	1.479979000	-0.490489000
H	-6.517003000	0.992606000	0.436053000
H	-7.017009000	2.047465000	-0.905088000
H	-5.917656000	0.690174000	-1.213569000
C	-5.429207000	3.580225000	0.663664000
H	-5.776184000	3.228239000	1.649793000
H	-4.606499000	4.292876000	0.835404000
H	-6.257234000	4.135248000	0.195794000
C	3.476662000	-3.434937000	-0.440133000
C	3.656467000	-4.668462000	-1.334961000
C	2.297670000	-4.847480000	-1.985236000
C	1.459356000	-3.705709000	-1.397611000
H	3.970762000	-5.517755000	-0.708050000
H	4.475412000	-4.475642000	-2.045633000
H	1.807898000	-5.805925000	-1.751750000
H	2.307440000	-4.752687000	-3.082535000
O	0.286675000	-3.515291000	-1.688283000
O	4.372887000	-2.968920000	0.247000000
C	-1.193219000	4.585851000	2.834541000
H	-2.029671000	5.124273000	2.363714000
H	-1.545714000	4.178839000	3.796090000
H	-0.393937000	5.313007000	3.047240000
C	-0.564041000	5.196297000	-2.236025000
H	-0.739732000	4.166756000	-2.567101000
H	-0.433072000	5.869580000	-3.090728000
Cl	0.924881000	5.230134000	-1.274979000
Cl	-1.981992000	5.739346000	-1.319035000
H	-0.378670000	3.935835000	0.963546000

56
A-OH_H

C	0.105808000	0.808212000	1.133403000
Br	1.095268000	-0.789121000	0.403059000
N	2.176107000	-2.908473000	-0.591888000
C	-1.927871000	2.473223000	1.405942000
C	1.032795000	2.006092000	1.210170000
C	-3.492453000	0.131650000	-0.139731000
C	-2.118640000	-0.183229000	0.411394000
C	-2.932814000	2.407105000	0.476045000
C	-4.035787000	1.418140000	0.526997000
H	-2.180560000	-0.471287000	1.472745000
H	-1.684187000	-1.046842000	-0.122844000
H	-3.461858000	0.267679000	-1.232377000
H	-4.178469000	-0.703134000	0.060807000
H	-2.866151000	3.069616000	-0.394416000
H	-4.237832000	1.174641000	1.585044000
H	-0.195816000	0.487152000	2.140114000
C	-0.937100000	3.601755000	1.481699000
H	-2.047480000	1.903669000	2.333654000
C	0.415104000	3.141191000	2.011664000
H	1.310457000	2.344209000	0.200717000
H	1.965491000	1.687107000	1.697441000
H	0.275317000	2.823139000	3.058301000
H	1.096174000	4.007194000	2.037158000
C	-1.111719000	0.928154000	0.255440000
C	-0.852572000	1.408652000	-1.145745000
H	-1.750427000	1.386826000	-1.774116000
H	-0.437185000	2.421710000	-1.156173000
H	-0.108130000	0.725574000	-1.585382000
C	-5.319448000	1.961095000	-0.121355000
H	-5.063578000	2.225575000	-1.164185000
C	-6.428024000	0.917703000	-0.148108000
H	-6.661756000	0.564835000	0.870562000
H	-7.349557000	1.348332000	-0.569698000
H	-6.165396000	0.040960000	-0.758106000
C	-5.782480000	3.225789000	0.594025000
H	-6.034394000	3.011071000	1.646156000
H	-5.006268000	4.008709000	0.590925000
H	-6.679672000	3.645836000	0.113478000
C	3.447016000	-3.384249000	-0.610284000
C	3.520892000	-4.704269000	-1.392904000
C	2.090731000	-4.937191000	-1.839096000
C	1.334131000	-3.727642000	-1.270861000
H	3.921115000	-5.488495000	-0.730937000
H	4.239023000	-4.588028000	-2.220046000
H	1.638144000	-5.859557000	-1.441733000
H	1.956260000	-4.961399000	-2.932145000
O	0.130941000	-3.562156000	-1.432140000
O	4.425036000	-2.865136000	-0.089777000
H	-1.368863000	4.293349000	2.235280000
C	1.291274000	4.983351000	-2.218181000
H	0.223611000	4.741204000	-2.179183000
H	1.530774000	5.584098000	-3.102560000
Cl	2.213761000	3.478397000	-2.297825000
Cl	1.689979000	5.955936000	-0.781438000
O	-0.864197000	4.231484000	0.229268000
H	-0.219993000	4.951160000	0.292327000

59
A-OMe_H

C	0.261834000	0.720386000	1.128764000
Br	1.213553000	-0.927462000	0.446104000
N	2.246070000	-3.086973000	-0.480905000

```
C       -1.702332000     2.499291000     1.333109000
C        1.240712000     1.870875000     1.270006000
C       -3.282956000     0.249535000    -0.359464000
C       -1.960967000    -0.151774000     0.259478000
C       -2.648538000     2.481656000     0.346815000
C       -3.798523000     1.545953000     0.307551000
H       -2.096135000    -0.452556000     1.310448000
H       -1.548027000    -1.031050000    -0.267329000
H       -3.180926000     0.409022000    -1.444663000
H       -4.019329000    -0.554651000    -0.222174000
H       -2.498510000     3.162512000    -0.498053000
H       -4.080446000     1.289252000     1.344061000
H       -0.115496000     0.406550000     2.111620000
C       -0.648633000     3.568519000     1.446083000
H       -1.882755000     1.919383000     2.244302000
C        0.643914000     3.032202000     2.051586000
H        1.583453000     2.202837000     0.278065000
H        2.130574000     1.500133000     1.798903000
H        0.426004000     2.713091000     3.084363000
H        1.372142000     3.854280000     2.126296000
C       -0.883201000     0.895779000     0.175057000
C       -0.527218000     1.395255000    -1.195188000
H       -1.381573000     1.374010000    -1.881514000
H       -0.118241000     2.411470000    -1.168533000
H        0.246232000     0.716736000    -1.590889000
C       -5.006782000     2.174235000    -0.406578000
H       -4.662995000     2.465320000    -1.416850000
C       -6.160000000     1.191530000    -0.555074000
H       -6.484571000     0.812763000     0.428822000
H       -7.026338000     1.684700000    -1.022783000
H       -5.896668000     0.325964000    -1.180634000
C       -5.458367000     3.435695000     0.321190000
H       -5.809178000     3.196745000     1.339236000
H       -4.643257000     4.171364000     0.412954000
H       -6.287314000     3.924915000    -0.213551000
C        3.497645000    -3.611765000    -0.445232000
C        3.562685000    -4.912301000    -1.260129000
C        2.153091000    -5.072184000    -1.795093000
C        1.412201000    -3.851814000    -1.230388000
H        3.889674000    -5.730177000    -0.598661000
H        4.333014000    -4.805622000    -2.040190000
H        1.642210000    -5.987860000    -1.457634000
H        2.082758000    -5.054486000    -2.894274000
O        0.227258000    -3.633693000    -1.451288000
O        4.464830000    -3.146654000     0.141856000
H       -1.070365000     4.315338000     2.151304000
C       -2.281041000     4.189796000    -3.272688000
H       -2.439064000     3.183281000    -2.870355000
H       -2.768337000     4.305164000    -4.247225000
Cl      -0.542078000     4.436957000    -3.491368000
Cl      -3.011028000     5.358187000    -2.154917000
O       -0.467920000     4.155299000     0.186489000
C       -0.037433000     5.488609000     0.202213000
H       -0.756031000     6.138762000     0.735444000
H        0.955050000     5.605187000     0.673882000
H        0.030074000     5.821174000    -0.841928000
```

65
A-Ph_H

```
C        0.243221000     0.918603000     0.970302000
Br       1.221604000    -0.738219000     0.412926000
N        2.165557000    -3.047358000    -0.428798000
C       -1.806002000     2.543691000     1.147556000
C        1.135783000     2.133276000     0.824403000
C       -3.384040000    -0.047928000     0.018575000
C       -1.962832000    -0.240622000     0.498465000
C       -2.869795000     2.292779000     0.315557000
C       -3.920449000     1.282607000     0.590389000
H       -1.942275000    -0.409819000     1.586814000
H       -1.514878000    -1.136159000     0.032301000
H       -3.437757000    -0.028123000    -1.081538000
H       -4.016529000    -0.880054000     0.357906000
H       -2.919031000     2.816952000    -0.646694000
H       -4.010418000     1.155650000     1.683438000
H        0.004792000     0.738269000     2.027515000
C       -0.879177000     3.740106000     1.073983000
H       -1.835695000     2.072342000     2.136821000
C        0.542332000     3.360364000     1.504701000
H        1.353387000     2.308665000    -0.239494000
H        2.100590000     1.903767000     1.299746000
H        0.514790000     3.162144000     2.588655000
H        1.204674000     4.228262000     1.367305000
C       -1.031608000     0.897021000     0.155336000
C       -0.861657000     1.183912000    -1.311851000
H       -1.786755000     1.026819000    -1.877249000
H       -0.497011000     2.198688000    -1.507378000
H       -0.107946000     0.475114000    -1.690891000
C       -5.280274000     1.715100000     0.016883000
H       -5.131628000     1.898153000    -1.062641000
C       -6.337095000     0.631971000     0.182260000
H       -6.463307000     0.360196000     1.243891000
H       -7.310917000     0.989684000    -0.186855000
H       -6.090396000    -0.283760000    -0.374869000
C       -5.733857000     3.020106000     0.661320000
H       -5.876178000     2.892113000     1.747643000
H       -4.999618000     3.828251000     0.512675000
H       -6.690509000     3.359603000     0.235266000
C        3.378522000    -3.650374000    -0.510021000
C        3.277095000    -4.984912000    -1.265591000
C        1.808604000    -5.078633000    -1.628818000
C        1.211049000    -3.791312000    -1.041027000
H        3.632507000    -5.794258000    -0.608213000
H        3.956771000    -4.956383000    -2.132009000
H        1.288622000    -5.946208000    -1.192441000
H        1.611434000    -5.100939000    -2.712456000
O        0.020097000    -3.514325000    -1.137896000
O        4.433646000    -3.226006000    -0.056921000
H       -1.274760000     4.371084000     1.891421000
C       -4.247593000     4.243837000    -3.922121000
H       -4.762045000     4.020260000    -4.863420000
H       -3.617788000     5.135388000    -4.012815000
Cl      -3.197998000     2.869780000    -3.531902000
Cl      -5.471688000     4.550965000    -2.680825000
C       -1.012016000     4.588304000    -0.177241000
C       -0.004178000     4.707792000    -1.140475000
C       -2.190917000     5.326625000    -0.360895000
C       -0.179005000     5.519390000    -2.263280000
C       -2.369044000     6.138722000    -1.477700000
C       -1.361645000     6.234587000    -2.439187000
H        0.937774000     4.170041000    -1.022993000
H       -2.982609000     5.266393000     0.392365000
H        0.622432000     5.593628000    -3.002823000
H       -3.299827000     6.698245000    -1.597197000
H       -1.495641000     6.871670000    -3.317192000
```

55
AB-H_H

```
C       -3.380531000     0.786990000    -0.683009000
C       -2.191068000    -0.135701000    -0.362157000
C       -2.598257000     2.781959000     0.587414000
C       -3.754916000     1.850324000     0.368605000
H       -2.363937000    -0.630341000     0.607653000
H       -2.179319000    -0.931258000    -1.128198000
H       -3.208333000     1.291965000    -1.649759000
H       -4.250739000     0.132416000    -0.840374000
H       -2.289512000     3.366957000    -0.289277000
H       -3.962658000     1.330192000     1.323589000
C       -0.159567000     0.663328000     0.814768000
C       -1.855706000     2.850650000     1.695376000
C        0.984120000     1.580877000     1.111524000
H       -0.590107000     0.165227000     1.692941000
C       -0.546300000     3.566384000     1.825223000
H       -2.157632000     2.246557000     2.563184000
C        0.612203000     2.622210000     2.179373000
H        1.323873000     2.093790000     0.198771000
H        1.846056000     1.003339000     1.489657000
H        0.366033000     2.096793000     3.119743000
H        1.507860000     3.227380000     2.396482000
C       -0.848511000     0.548422000    -0.344821000
C       -0.428803000     1.208974000    -1.621551000
H       -0.670651000     0.571598000    -2.486948000
H       -0.973832000     2.158287000    -1.764183000
H        0.645166000     1.434536000    -1.651724000
C       -5.035676000     2.618093000    -0.034766000
H       -4.833343000     3.079778000    -1.020374000
H       -0.321891000     4.106597000     0.889773000
H       -0.609279000     4.328980000     2.622758000
N        1.878869000    -3.296941000    -0.673697000
C        3.190405000    -3.517240000    -0.273245000
C        3.579039000    -4.890436000    -0.775247000
C        2.347223000    -5.430953000    -1.490924000
C        1.303645000    -4.340851000    -1.387068000
H        3.891171000    -5.497542000     0.087446000
H        4.456140000    -4.781286000    -1.430461000
H        1.936542000    -6.342860000    -1.032236000
H        2.514463000    -5.649703000    -2.555999000
O        0.181699000    -4.345291000    -1.824940000
O        3.863645000    -2.740489000     0.354350000
Br       0.940315000    -1.714310000    -0.267777000
C       -6.250401000     1.707291000    -0.180782000
H       -6.130665000     0.958734000    -0.977500000
H       -6.451067000     1.167559000     0.760709000
H       -7.149875000     2.296080000    -0.421621000
C       -5.343219000     3.739124000     0.952546000
H       -6.249251000     4.289832000     0.653122000
H       -5.519101000     3.334313000     1.964231000
H       -4.517510000     4.462318000     1.027414000
C       -3.515116000     5.863746000    -1.765628000
H       -4.368443000     5.362208000    -1.296429000
H       -3.840359000     6.718702000    -2.368891000
Cl      -2.447685000     6.463537000    -0.485608000
Cl      -2.712795000     4.706491000    -2.844887000
```

58
AB-Me_H

```
C       -0.283090000     0.772313000     0.905065000
Br       1.006086000    -1.468396000    -0.256933000
N        2.106202000    -2.923898000    -0.728365000
C       -2.070652000     2.838089000     1.850218000
C        0.805872000     1.750145000     1.210442000
C       -3.518505000     0.734172000    -0.545803000
```

C	-2.270117000	-0.125367000	-0.275904000
C	-2.834682000	2.743540000	0.759047000
C	-3.931194000	1.739339000	0.546994000
H	-2.397889000	-0.661393000	0.678750000
H	-2.224036000	-0.892057000	-1.069543000
H	-3.398152000	1.277096000	-1.500127000
H	-4.350554000	0.032508000	-0.705630000
H	-2.584094000	3.364181000	-0.112763000
H	-4.070531000	1.178398000	1.491253000
H	-0.674616000	0.228859000	1.774559000
C	-0.817411000	3.656534000	1.978971000
H	-2.310372000	2.201683000	2.715149000
C	0.391545000	2.757332000	2.294861000
H	1.110576000	2.295590000	0.305537000
H	1.700520000	1.216241000	1.576312000
H	0.185939000	2.214397000	3.235995000
H	1.258980000	3.406307000	2.503655000
C	-0.971797000	0.638229000	-0.252598000
C	-0.603436000	1.347330000	-1.519088000
H	-1.226032000	2.248875000	-1.651601000
H	0.448559000	1.659845000	-1.540718000
H	-0.788721000	0.702156000	-2.392677000
C	-5.274485000	2.429864000	0.213231000
H	-5.126772000	2.978471000	-0.737125000
C	-6.418831000	1.440624000	0.014602000
H	-6.548377000	0.805583000	0.907944000
H	-7.367889000	1.975286000	-0.150028000
H	-6.267194000	0.777411000	-0.849404000
C	-5.650026000	3.445807000	1.286800000
H	-5.788954000	2.950379000	2.263367000
H	-4.874856000	4.215965000	1.415591000
H	-6.593830000	3.955069000	1.035025000
C	3.213285000	-3.298266000	0.022051000
C	3.832030000	-4.491664000	-0.671569000
C	2.962326000	-4.752895000	-1.895138000
C	1.875353000	-3.701494000	-1.855682000
H	3.854655000	-5.330173000	0.040412000
H	4.875829000	-4.244805000	-0.916305000
H	2.483981000	-5.743633000	-1.893406000
H	3.501886000	-4.654055000	-2.848857000
O	0.971014000	-3.541211000	-2.634888000
O	3.583331000	-2.753462000	1.030351000
C	-0.966932000	4.730437000	3.055452000
H	-1.807444000	5.404951000	2.828693000
H	-1.158456000	4.276851000	4.042590000
H	-0.053074000	5.340875000	3.139284000
C	0.434189000	4.860295000	-2.377421000
H	0.433339000	3.850213000	-2.800898000
H	0.761931000	5.600181000	-3.116337000
Cl	1.572350000	4.895943000	-1.020038000
Cl	-1.223335000	5.264487000	-1.895658000
H	-0.631429000	4.156446000	1.012512000

56
AB-OH_H

C	-0.350140000	0.759831000	0.744227000
Br	0.923917000	-1.526695000	-0.322417000
N	2.042482000	-2.983218000	-0.741268000
C	-2.127303000	2.854474000	1.609716000
C	0.738295000	1.772739000	0.905531000
C	-3.674808000	0.567738000	-0.502155000
C	-2.396252000	-0.246409000	-0.231609000
C	-2.972457000	2.700712000	0.589106000
C	-4.049726000	1.655364000	0.523551000
H	-2.459446000	-0.700511000	0.770782000
H	-2.380047000	-1.078076000	-0.958220000
H	-3.620051000	1.026834000	-1.505030000
H	-4.499491000	-0.158571000	-0.552467000
H	-2.809980000	3.295247000	-0.318741000
H	-4.119231000	1.172200000	1.517226000
H	-0.672540000	0.276279000	1.675224000
C	-0.888809000	3.697779000	1.609411000
H	-2.264862000	2.249835000	2.514562000
C	0.362188000	2.858616000	1.924673000
H	0.991488000	2.238241000	-0.056403000
H	1.660934000	1.286308000	1.266784000
H	0.217800000	2.400052000	2.918145000
H	1.216019000	3.549868000	2.029469000
C	-1.113985000	0.535223000	-0.350675000
C	-0.848889000	1.156306000	-1.686250000
H	-1.075462000	0.444754000	-2.496383000
H	-1.504638000	2.030599000	-1.839716000
H	0.189169000	1.490351000	-1.805879000
C	-5.427887000	2.285075000	0.215177000
H	-5.350264000	2.755342000	-0.784136000
C	-6.555741000	1.258867000	0.167989000
H	-6.614988000	0.696150000	1.115610000
H	-7.526503000	1.758313000	0.020228000
H	-6.439334000	0.530872000	-0.648002000
C	-5.766245000	3.379229000	1.222314000
H	-5.835630000	2.964217000	2.242800000
H	-5.004756000	4.173333000	1.240609000
H	-6.735601000	3.846520000	0.986806000
C	3.409935000	-2.968458000	-0.497341000
C	3.955009000	-4.299293000	-0.966148000
C	2.754334000	-5.070684000	-1.500144000
C	1.565612000	-4.152723000	-1.319348000
H	4.449974000	-4.788177000	-0.113877000
H	4.728415000	-4.107049000	-1.724584000
H	2.553006000	-6.005439000	-0.955916000
H	2.833060000	-5.326243000	-2.567318000
O	0.416063000	-4.363686000	-1.609880000
O	4.015921000	-2.052224000	-0.003746000
H	-0.978970000	4.433533000	2.436386000
C	1.351940000	4.430981000	-2.063612000
H	0.436144000	4.001329000	-1.640065000
H	1.148626000	5.025579000	-2.961106000
Cl	2.454232000	3.116501000	-2.498262000
Cl	2.046544000	5.511719000	-0.832246000
O	-0.779228000	4.379279000	0.380842000
H	-0.025837000	4.981049000	0.447321000

59
AB-OMe_H

C	-0.150546000	0.683487000	0.845234000
Br	1.068604000	-1.618523000	-0.223592000
N	2.123987000	-3.122659000	-0.648407000
C	-1.919124000	2.865830000	1.526306000
C	0.961863000	1.651861000	1.096094000
C	-3.390006000	0.662916000	-0.683136000
C	-2.163586000	-0.192755000	-0.318677000
C	-2.725230000	2.757854000	0.469316000
C	-3.824907000	1.747458000	0.322408000
H	-2.328156000	-0.670407000	0.661073000
H	-2.105156000	-1.006406000	-1.063635000
H	-3.231531000	1.138992000	-1.667261000
H	-4.226450000	-0.037585000	-0.824911000
H	-2.491142000	3.359680000	-0.416349000
H	-3.984656000	1.259641000	1.303244000
H	-0.547430000	0.179373000	1.735533000
C	-0.639845000	3.651271000	1.557223000
H	-2.116678000	2.265204000	2.422284000
C	0.533188000	2.781557000	2.044883000
H	1.319116000	2.087386000	0.151863000
H	1.822405000	1.136508000	1.556158000
H	0.250227000	2.362536000	3.025435000
H	1.403955000	3.429827000	2.235225000
C	-0.851402000	0.546000000	-0.305190000
C	-0.462138000	1.245727000	-1.571759000
H	-0.908064000	0.760794000	-2.452882000
H	-0.808253000	2.291855000	-1.542687000
H	0.627284000	1.275948000	-1.713634000
C	-5.156163000	2.411648000	-0.096468000
H	-4.995872000	2.847184000	-1.101250000
C	-6.311802000	1.420313000	-0.185758000
H	-6.464352000	0.907355000	0.779464000
H	-7.250296000	1.940948000	-0.434421000
H	-6.156588000	0.649086000	-0.954341000
C	-5.521439000	3.551899000	0.847841000
H	-5.655661000	3.181290000	1.878850000
H	-4.743042000	4.329241000	0.874593000
H	-6.464543000	4.031323000	0.540690000
C	3.497773000	-3.153252000	-0.447084000
C	3.980424000	-4.511200000	-0.907050000
C	2.739259000	-5.242213000	-1.403863000
C	1.589070000	-4.281839000	-1.195393000
H	4.472228000	-5.006782000	-0.056697000
H	4.745771000	-4.361525000	-1.682953000
H	2.520612000	-6.168421000	-0.851954000
H	2.779805000	-5.502207000	-2.472209000
O	0.424160000	-4.456407000	-1.446769000
O	4.151535000	-2.251560000	0.011402000
H	-0.742735000	4.481227000	2.290821000
C	-2.589267000	3.988211000	-3.391392000
H	-2.623115000	3.072696000	-2.790685000
H	-3.048281000	3.836606000	-4.374760000
Cl	-0.890889000	4.430329000	-3.635758000
Cl	-3.506648000	5.243911000	-2.540207000
O	-0.422066000	4.193604000	0.279319000
C	0.536517000	5.205774000	0.207124000
H	0.330134000	6.021267000	0.927689000
H	1.564853000	4.841330000	0.389793000
H	0.496697000	5.621485000	-0.810279000

65
AB-Ph_H

C	-0.275124000	0.698442000	0.593216000
Br	0.962901000	-1.701261000	-0.254250000
N	2.057553000	-3.203933000	-0.560294000
C	-2.036103000	2.868110000	1.233133000
C	0.797920000	1.735281000	0.510124000
C	-3.667125000	0.257253000	-0.367861000
C	-2.346989000	-0.483109000	-0.093885000
C	-2.982815000	2.549258000	0.347511000
C	-3.996149000	1.455533000	0.542781000
H	-2.323984000	-0.810158000	0.958483000
H	-2.348649000	-1.397769000	-0.712994000
H	-3.699851000	0.589796000	-1.419995000
H	-4.469302000	-0.488575000	-0.266120000
H	-2.990728000	3.034892000	-0.636306000
H	-3.931681000	1.110200000	1.592705000

H	-0.509383000	0.340045000	1.603406000
C	-0.846992000	3.778787000	1.043269000
H	-2.045295000	2.352219000	2.202536000
C	0.467823000	3.010135000	1.311544000
H	1.016215000	1.971721000	-0.539196000
H	1.735180000	1.334367000	0.933601000
H	0.449037000	2.732223000	2.379044000
H	1.309806000	3.713708000	1.202420000
C	-1.107392000	0.312928000	-0.402418000
C	-0.965199000	0.778866000	-1.817971000
H	-1.315942000	0.008483000	-2.522331000
H	-1.586814000	1.675436000	-1.985227000
H	0.067458000	1.041766000	-2.081916000
C	-5.433882000	1.974236000	0.307356000
H	-5.496450000	2.277540000	-0.754437000
C	-6.498375000	0.912124000	0.563691000
H	-6.424185000	0.520741000	1.593029000
H	-7.506860000	1.339629000	0.445555000
H	-6.427388000	0.058245000	-0.125712000
C	-5.720196000	3.207260000	1.157417000
H	-5.646232000	2.969367000	2.232863000
H	-5.013939000	4.024518000	0.949950000
H	-6.737140000	3.587106000	0.969573000
C	3.412058000	-3.218707000	-0.253083000
C	3.935393000	-4.585155000	-0.636766000
C	2.736319000	-5.344095000	-1.191652000
C	1.570170000	-4.384675000	-1.105908000
H	4.375658000	-5.049230000	0.258326000
H	4.747978000	-4.450369000	-1.366102000
H	2.481946000	-6.247683000	-0.617848000
H	2.854743000	-5.648951000	-2.242115000
O	0.428221000	-4.575120000	-1.437798000
O	4.023872000	-2.299278000	0.227357000
H	-0.894243000	4.515116000	1.868215000
C	-4.416510000	4.629409000	-3.607581000
H	-5.075425000	4.593167000	-4.482306000
H	-3.661450000	5.416623000	-3.708476000
Cl	-3.573513000	3.074369000	-3.491416000
Cl	-5.405135000	4.989676000	-2.183747000
C	-0.915170000	4.604106000	-0.226553000
C	-0.013948000	4.484884000	-1.289947000
C	-1.933391000	5.565226000	-0.337917000
C	-0.133487000	5.283780000	-2.430465000
C	-2.055309000	6.366538000	-1.469140000
C	-1.154342000	6.225964000	-2.527672000
H	0.804427000	3.766869000	-1.239474000
H	-2.644133000	5.684030000	0.485072000
H	0.584704000	5.166674000	-3.246416000
H	-2.860332000	7.103657000	-1.526595000
H	-1.245517000	6.853141000	-3.418394000

55

B-H_H

C	-2.124155000	0.069047000	0.523742000
C	-1.093191000	-0.144543000	1.621640000
C	-2.157994000	1.747885000	0.363938000
C	-3.438967000	1.306651000	0.709903000
H	-1.570900000	-0.187468000	2.612651000
H	-0.595090000	-1.105676000	1.427849000
H	-1.817015000	-0.192892000	-0.519859000
H	-3.023637000	-0.561375000	0.670597000
H	-1.974595000	2.006325000	-0.683585000
H	-3.701785000	1.278974000	1.774927000
C	0.646968000	1.362036000	2.801744000
C	-1.170772000	2.213577000	1.406904000
C	1.356119000	2.707005000	2.725280000
H	-0.039611000	1.345356000	3.661091000
C	-0.520286000	3.570871000	1.211841000
H	-1.714408000	2.244368000	2.367443000
C	0.373252000	3.843066000	2.422168000
H	2.138293000	2.664966000	1.951409000
H	1.867416000	2.904318000	3.679384000
H	-0.272706000	3.997171000	3.303882000
H	0.937020000	4.778399000	2.280908000
C	-0.144962000	1.056965000	1.533723000
C	0.747289000	0.935078000	0.298078000
H	0.152705000	0.787802000	-0.614993000
H	1.378490000	1.820578000	0.146403000
H	1.408463000	0.062983000	0.401470000
C	-4.553444000	1.158956000	-0.281519000
H	-4.102058000	0.923110000	-1.259474000
H	0.059664000	3.589621000	0.275263000
H	-1.286903000	4.356885000	1.126404000
N	-0.280353000	-2.297755000	-1.064552000
C	0.516375000	-3.363648000	-1.364276000
C	0.857516000	-3.401648000	-2.860665000
C	0.143710000	-2.184554000	-3.415413000
C	-0.528378000	-1.589704000	-2.178892000
H	0.512811000	-4.358410000	-3.283353000
H	1.952078000	-3.375833000	-2.979480000
H	-0.625081000	-2.412416000	-4.170481000
H	0.811702000	-1.429201000	-3.858623000
O	-1.217699000	-0.560241000	-2.237566000
O	0.922690000	-4.196919000	-0.567143000
Br	1.935585000	-0.065669000	3.228333000
C	-5.541236000	0.070446000	0.109021000
H	-5.061817000	-0.920243000	0.157583000
H	-5.993224000	0.281024000	1.091619000
H	-6.352359000	0.008982000	-0.631410000
C	-5.226836000	2.530559000	-0.391250000
H	-6.054033000	2.475754000	-1.114878000
H	-5.644860000	2.850046000	0.576717000
H	-4.519408000	3.299423000	-0.735549000
C	-2.590232000	3.685606000	-3.570297000
H	-3.668883000	3.661130000	-3.762132000
H	-2.049631000	4.117521000	-4.420372000
Cl	-2.310754000	4.734718000	-2.160520000
Cl	-2.019022000	2.032596000	-3.311679000

58

B-Me_H

C	0.401635000	1.748582000	3.365349000
Br	1.686770000	0.614154000	4.338012000
N	-0.789408000	-1.017542000	-1.570181000
C	-1.239848000	2.023138000	1.579248000
C	1.043862000	3.077677000	2.997315000
C	-1.927323000	-0.371379000	1.208841000
C	-1.059423000	-0.176550000	2.442525000
C	-2.043223000	1.215900000	0.589126000
C	-3.316769000	0.737746000	0.903474000
H	-1.682558000	0.004635000	3.332303000
H	-0.475966000	-1.092444000	2.619576000
H	-1.458185000	-0.793499000	0.285325000
H	-2.774319000	-1.057627000	1.394839000
H	-1.718272000	1.195656000	-0.466561000
H	-3.723030000	0.964688000	1.897774000
H	-0.400196000	1.905495000	4.101631000
C	-0.655869000	3.339446000	1.081743000
H	-1.913966000	2.267641000	2.420461000
C	0.054665000	3.993192000	2.271312000
H	1.926667000	2.895316000	2.365378000
H	1.406129000	3.574069000	3.910015000
H	-0.716794000	4.328584000	2.987766000
H	0.583622000	4.899441000	1.934919000
C	-0.179906000	1.042303000	2.144999000
C	0.887370000	0.670766000	1.117075000
H	0.441560000	0.213516000	0.222093000
H	1.476699000	1.533795000	0.783919000
H	1.581379000	-0.062710000	1.553585000
C	-4.277048000	0.225723000	-0.130671000
H	-3.684498000	-0.224139000	-0.944216000
C	-5.245141000	-0.804922000	0.429809000
H	-5.840169000	-0.380672000	1.254681000
H	-5.941606000	-1.138985000	-0.353397000
H	-4.722800000	-1.697865000	0.809216000
C	-5.008055000	1.448436000	-0.691904000
H	-5.568402000	1.976119000	0.096566000
H	-4.305377000	2.159000000	-1.152118000
H	-5.726828000	1.129112000	-1.461421000
C	-0.200466000	-2.178768000	-1.983440000
C	0.521614000	-1.982726000	-3.320008000
C	0.350780000	-0.501261000	-3.600072000
C	-0.531111000	-0.029586000	-2.448139000
H	0.052124000	-2.633401000	-4.074711000
H	1.567581000	-2.310732000	-3.220468000
H	-0.134875000	-0.264676000	-4.559091000
H	1.295656000	0.064183000	-3.567484000
O	-0.932664000	1.137376000	-2.344143000
O	-0.237306000	-3.241218000	-1.382137000
C	-1.721254000	4.254660000	0.498044000
H	-2.178156000	3.819751000	-0.404752000
H	-2.525859000	4.442463000	1.228938000
H	-1.291279000	5.226953000	0.212927000
C	1.353863000	3.177111000	-2.800768000
H	0.438469000	2.564912000	-2.774434000
H	1.706323000	3.355999000	-3.822788000
Cl	2.624702000	2.287054000	-1.934697000
Cl	1.010004000	4.751960000	-2.056968000
H	0.084391000	3.126812000	0.292943000

56

B-OH_H

C	0.397999000	2.362364000	2.655331000
Br	1.899954000	1.581057000	3.661349000
N	-0.715559000	-1.458577000	-1.452199000
C	-1.403047000	2.123637000	1.024357000
C	0.823947000	3.668738000	1.999505000
C	-1.808130000	-0.336501000	1.106794000
C	-0.900033000	0.158191000	2.222449000
C	-2.184215000	1.078691000	0.264892000
C	-3.352943000	0.528229000	0.803673000
H	-1.483386000	0.434029000	3.115188000
H	-0.204361000	-0.645206000	2.506360000
H	-1.326924000	-0.849953000	0.221771000
H	-2.534331000	-1.101471000	1.439988000
H	-1.996851000	0.913090000	-0.807468000
H	-3.678675000	0.866683000	1.795791000
H	-0.352070000	2.558779000	3.435726000
C	-1.089307000	3.419951000	0.299124000
H	-2.026469000	2.424434000	1.883067000
C	-0.349002000	4.335852000	1.278070000

H	1.645966000	3.481263000	1.292754000
H	1.217783000	4.352639000	2.765883000
H	-1.082038000	4.691321000	2.021321000
H	0.008597000	5.228173000	0.739329000
C	-0.184062000	1.383555000	1.639465000
C	0.853047000	0.918477000	0.616706000
H	0.386049000	0.373466000	-0.215700000
H	1.423797000	1.750910000	0.193915000
H	1.561429000	0.232247000	1.104151000
C	-4.348057000	-0.229078000	-0.024980000
H	-3.810350000	-0.633941000	-0.897771000
C	-5.034037000	-1.346254000	0.745820000
H	-5.549270000	-0.956240000	1.638741000
H	-5.784650000	-1.840810000	0.111519000
H	-4.320211000	-2.119295000	1.072839000
C	-5.353156000	0.807471000	-0.535843000
H	-5.886425000	1.294666000	0.296477000
H	-4.857429000	1.587431000	-1.133087000
H	-6.101522000	0.313317000	-1.173616000
C	0.195391000	-2.434790000	-1.724650000
C	0.279218000	-2.699001000	-3.231851000
C	-0.734347000	-1.735180000	-3.820254000
C	-1.282186000	-1.007685000	-2.592739000
H	0.057232000	-3.760478000	-3.423105000
H	1.313188000	-2.523650000	-3.568046000
H	-1.567651000	-2.223735000	-4.349300000
H	-0.306636000	-0.994753000	-4.514198000
O	-2.134778000	-0.119851000	-2.665108000
O	0.866370000	-3.031219000	-0.895029000
H	-2.049152000	3.905002000	0.036411000
C	2.584677000	3.414201000	-2.030340000
H	1.739803000	2.747760000	-1.824727000
H	3.074140000	3.175723000	-2.981003000
Cl	3.771542000	3.231193000	-0.729805000
Cl	1.948871000	5.070959000	-2.138949000
O	-0.362145000	3.148122000	-0.875346000
H	-0.237190000	3.984506000	-1.343659000

59

B-OMe_H

C	0.653306000	1.628337000	2.717025000
Br	2.065506000	0.359867000	3.239110000
N	-0.182901000	-2.234928000	-1.153270000
C	-1.250613000	2.203784000	1.303198000
C	1.241532000	3.014524000	2.493375000
C	-2.060664000	-0.058707000	0.623936000
C	-1.000688000	-0.099258000	1.718836000
C	-2.198076000	1.586449000	0.306764000
C	-3.461253000	1.115907000	0.679783000
H	-1.467134000	-0.078172000	2.715694000
H	-0.441230000	-1.040636000	1.616956000
H	-1.782638000	-0.413058000	-0.391878000
H	-2.928043000	-0.700078000	0.882370000
H	-2.007615000	1.764567000	-0.756409000
H	-3.740851000	1.170867000	1.739578000
H	-0.012462000	1.638312000	3.592820000
C	-0.771096000	3.617119000	1.018224000
H	-1.783736000	2.263037000	2.266562000
C	0.150508000	4.037499000	2.168204000
H	1.979357000	2.973598000	1.677852000
H	1.784144000	3.332848000	3.395737000
H	-0.479915000	4.196892000	3.058612000
H	0.612707000	5.009751000	1.937406000
C	-0.132469000	1.147036000	1.501236000
C	0.753417000	0.944549000	0.270809000
H	0.153135000	0.708914000	-0.617702000
H	1.339437000	1.837693000	0.030061000
H	1.435392000	0.100485000	0.446716000
C	-4.544164000	0.812443000	-0.308430000
H	-4.061157000	0.547977000	-1.263562000
C	-5.457249000	-0.317791000	0.141159000
H	-5.930060000	-0.083872000	1.108488000
H	-6.256473000	-0.475431000	-0.597913000
H	-4.910967000	-1.268640000	0.245859000
C	-5.315548000	2.121865000	-0.507681000
H	-5.784312000	2.455755000	0.431619000
H	-4.660405000	2.925737000	-0.874164000
H	-6.114128000	1.965737000	-1.248225000
C	0.870748000	-3.054246000	-1.447435000
C	1.351557000	-2.846078000	-2.890078000
C	0.429922000	-1.762261000	-3.415012000
C	-0.475444000	-1.480888000	-2.220291000
H	1.280569000	-3.800828000	-3.434138000
H	2.415807000	-2.563806000	-2.876065000
H	-0.185683000	-2.065700000	-4.276425000
H	0.942201000	-0.829550000	-3.699515000
O	-1.374020000	-0.619741000	-2.257565000
O	1.383572000	-3.854459000	-0.680317000
H	-1.649216000	4.293192000	1.008358000
C	-1.158364000	1.874819000	-4.001557000
H	-1.321447000	0.894613000	-3.523654000
H	-1.357964000	1.864726000	-5.078954000
Cl	0.545258000	2.313852000	-3.763249000
Cl	-2.275243000	3.042524000	-3.257455000
O	-0.153458000	3.649589000	-0.241451000
C	-0.099711000	4.910597000	-0.841291000

H	-1.107517000	5.356459000	-0.946194000
H	0.533325000	5.622884000	-0.279429000
H	0.327743000	4.781090000	-1.844901000

65

B-Ph_H

C	0.440127000	2.284345000	2.805122000
Br	2.022527000	1.221923000	3.299372000
N	1.137134000	2.186799000	-3.418539000
C	-1.490353000	2.642339000	1.346725000
C	0.805649000	3.753360000	2.689963000
C	-1.958128000	0.287058000	0.627393000
C	-0.892851000	0.387229000	1.711657000
C	-2.337268000	1.892110000	0.341483000
C	-3.539580000	1.274262000	0.712281000
H	-1.361453000	0.307826000	2.704226000
H	-0.188263000	-0.449726000	1.600758000
H	-1.644213000	-0.038263000	-0.371683000
H	-2.736489000	-0.450032000	0.917370000
H	-2.187211000	2.088025000	-0.723827000
H	-3.790302000	1.262437000	1.781745000
H	-0.241822000	2.131763000	3.655038000
C	-1.251191000	4.151851000	1.153022000
H	-2.034661000	2.575686000	2.304208000
C	-0.425441000	4.606965000	2.368243000
H	1.586854000	3.872858000	1.925330000
H	1.241265000	4.096314000	3.640213000
H	-1.093770000	4.581684000	3.244638000
H	-0.120220000	5.657170000	2.245216000
C	-0.230796000	1.758685000	1.540330000
C	0.695551000	1.740438000	0.328268000
H	0.153351000	1.532293000	-0.604997000
H	1.226101000	2.685421000	0.174840000
H	1.455446000	0.955740000	0.448289000
C	-4.601158000	0.846427000	-0.241104000
H	-4.134993000	0.670223000	-1.222976000
C	-5.347944000	-0.396152000	0.217110000
H	-5.802590000	-0.244536000	1.209099000
H	-6.153002000	-0.634047000	-0.493468000
H	-4.683217000	-1.272674000	0.271939000
C	-5.531429000	2.060497000	-0.371647000
H	-6.015259000	2.300033000	0.588304000
H	-4.989917000	2.951664000	-0.721450000
H	-6.319580000	1.828620000	-1.103228000
C	0.872194000	2.635785000	-4.662008000
C	2.048969000	3.407888000	-5.264660000
C	3.085561000	3.368713000	-4.161000000
C	2.394313000	2.559019000	-3.055606000
H	1.711327000	4.420217000	-5.536734000
H	2.362405000	2.914665000	-6.198336000
H	3.352953000	4.359039000	-3.759553000
H	4.028157000	2.871705000	-4.439201000
O	2.942857000	2.305714000	-1.988491000
O	-0.180008000	2.470856000	-5.287058000
H	-2.244136000	4.620473000	1.256824000
C	-2.577523000	0.969524000	-4.238398000
H	-3.077215000	0.526428000	-5.107241000
H	-1.631298000	1.475674000	-4.512161000
Cl	-2.217980000	-0.351554000	-3.102420000
Cl	-3.676376000	2.184590000	-3.547701000
C	-0.741008000	4.610975000	-0.202630000
C	0.535560000	5.153446000	-0.398051000
C	-1.603316000	4.566406000	-1.309225000
C	0.943828000	5.608003000	-1.652362000
C	-1.196694000	5.005522000	-2.567348000
C	0.084424000	5.526740000	-2.745752000
H	1.235022000	5.231340000	0.435263000
H	-2.625338000	4.199724000	-1.183468000
H	1.947866000	6.023008000	-1.772450000
H	-1.884485000	4.933469000	-3.413153000
H	0.408219000	5.871387000	-3.730830000

68

A-Ph_Me

C	-0.431754000	0.749868000	0.524195000
Br	0.797189000	-1.628457000	-0.409743000
N	1.893011000	-3.126761000	-0.733589000
C	-2.082852000	2.982507000	1.347839000
C	0.671945000	1.755313000	0.576219000
C	-3.766504000	0.498387000	-0.522105000
C	-2.486126000	-0.332798000	-0.333958000
C	-2.956624000	2.673672000	0.387568000
C	-4.021211000	1.617650000	0.504415000
H	-2.489458000	-0.776298000	0.675078000
H	-2.524291000	-1.170006000	-1.052836000
H	-3.783845000	0.938274000	-1.534865000
H	-4.607604000	-0.209603000	-0.488181000
H	-2.872544000	3.144790000	-0.599125000
H	-3.960546000	1.176753000	1.518092000
H	-0.730526000	0.331817000	1.493872000
C	-0.838076000	3.846816000	1.284625000
H	-2.192544000	2.473155000	2.315558000
C	0.382560000	2.921966000	1.536480000
H	0.899811000	2.125412000	-0.431815000
H	1.593759000	1.271046000	0.943909000
H	0.250334000	2.502829000	2.548624000

```
H        1.285641000      3.551518000      1.598003000
C       -1.211886000      0.442075000     -0.537963000
C       -0.985142000      0.982683000     -1.915448000
H       -1.652896000      1.840999000     -2.103362000
H        0.045533000      1.322626000     -2.079619000
H       -1.224044000      0.223079000     -2.676717000
C       -5.437693000      2.222289000      0.348315000
H       -5.508192000      2.618116000     -0.681655000
C       -6.544678000      1.189417000      0.536100000
H       -6.470549000      0.710180000      1.527495000
H       -7.534570000      1.669026000      0.474856000
H       -6.523504000      0.394141000     -0.223121000
C       -5.654834000      3.388714000      1.305465000
H       -5.574069000      3.056335000      2.355024000
H       -4.916793000      4.189907000      1.155199000
H       -6.656986000      3.825268000      1.169698000
C        3.153886000     -3.264841000     -0.167701000
C        3.716321000     -4.579549000     -0.660894000
C        2.636377000     -5.180233000     -1.552253000
C        1.502389000     -4.178891000     -1.551795000
H        3.963818000     -5.199515000      0.213505000
H        4.658655000     -4.372509000     -1.189875000
H        2.245907000     -6.140705000     -1.184014000
H        2.958176000     -5.337446000     -2.592416000
O        0.450943000     -4.250429000     -2.134727000
O        3.672775000     -2.467936000      0.571374000
C       -0.884765000      4.834373000      2.468238000
H       -1.745596000      5.514448000      2.385074000
H       -0.972374000      4.288885000      3.421633000
H        0.032187000      5.443562000      2.504625000
C       -5.268530000      3.965565000     -3.343357000
H       -5.846473000      3.092420000     -3.020955000
H       -5.641007000      4.358088000     -4.296246000
Cl      -3.586243000      3.459976000     -3.578275000
Cl      -5.446257000      5.232593000     -2.116892000
C       -0.733317000      4.671640000      0.003631000
C        0.434415000      4.740165000     -0.767128000
C       -1.818159000      5.469976000     -0.395971000
C        0.506115000      5.544800000     -1.906976000
C       -1.753653000      6.271279000     -1.532835000
C       -0.589511000      6.308197000     -2.301583000
H        1.317414000      4.167845000     -0.481998000
H       -2.737509000      5.459685000      0.193301000
H        1.431811000      5.570694000     -2.488226000
H       -2.624529000      6.863111000     -1.825034000
H       -0.537111000      6.931969000     -3.197689000


68
AB-Ph_Me
C        0.204984000      0.990154000      1.113808000
Br       1.191768000     -0.661911000      0.559164000
N        1.922946000     -3.011618000     -0.460227000
C       -1.821408000      2.742105000      1.303805000
C        1.112548000      2.201530000      1.075198000
C       -3.365648000      0.109320000     -0.042172000
C       -1.955654000     -0.155747000      0.440846000
C       -2.786037000      2.411943000      0.396302000
C       -3.853993000      1.401029000      0.632899000
H       -1.959802000     -0.404709000      1.513656000
H       -1.512858000     -1.025036000     -0.083877000
H       -3.401054000      0.221633000     -1.137912000
H       -4.018228000     -0.736141000      0.217006000
H       -2.794313000      2.905028000     -0.581137000
H       -3.925533000      1.207517000      1.717589000
H       -0.111197000      0.770375000      2.142178000
C       -0.853384000      3.909112000      1.288719000
H       -1.884585000      2.252174000      2.283356000
C        0.511207000      3.403033000      1.792696000
H        1.387703000      2.429468000      0.035393000
H        2.048039000      1.933888000      1.587755000
H        0.379148000      3.128644000      2.852335000
H        1.224645000      4.241067000      1.786704000
C       -0.990981000      0.972735000      0.198293000
C       -0.746644000      1.358842000     -1.228399000
H       -1.638217000      1.235359000     -1.852791000
H       -0.370028000      2.383181000     -1.334396000
H        0.030885000      0.674423000     -1.607492000
C       -5.215093000      1.910351000      0.127974000
H       -5.089434000      2.156092000     -0.941801000
C       -6.300437000      0.849821000      0.252346000
H       -6.410614000      0.516491000      1.298083000
H       -7.271482000      1.256644000     -0.070640000
H       -6.093186000     -0.036564000     -0.365102000
C       -5.614739000      3.185213000      0.861746000
H       -5.735554000      2.994141000      1.941424000
H       -4.860025000      3.979598000      0.746412000
H       -6.569656000      3.577556000      0.480033000
C        3.060147000     -3.706240000     -0.719276000
C        2.802118000     -4.822465000     -1.743815000
C        1.331471000     -4.668273000     -2.075132000
C        0.889157000     -3.488971000     -1.196017000
H        3.062993000     -5.790911000     -1.288202000
H        3.477387000     -4.681880000     -2.602636000
H        0.716466000     -5.548785000     -1.830443000
H        1.129612000     -4.421810000     -3.129762000
O       -0.265090000     -3.069131000     -1.197154000
O        4.161696000     -3.506318000     -0.223786000
C       -1.395976000      4.877470000      2.365409000
H       -2.388216000      5.268276000      2.100312000
H       -1.472295000      4.372056000      3.340321000
H       -0.704214000      5.727277000      2.470071000
C       -4.960577000      3.538771000     -3.768105000
H       -5.639825000      2.680308000     -3.722466000
H       -5.007283000      4.028257000     -4.747364000
Cl      -3.305092000      2.952000000     -3.528932000
Cl      -5.464099000      4.712242000     -2.539715000
C       -0.790616000      4.666096000     -0.038936000
C        0.411339000      4.886915000     -0.724134000
C       -1.957418000      5.231056000     -0.579296000
C        0.442124000      5.612938000     -1.916097000
C       -1.932616000      5.949396000     -1.772025000
C       -0.730057000      6.141010000     -2.451352000
H        1.351152000      4.495899000     -0.334745000
H       -2.912143000      5.111843000     -0.062428000
H        1.396675000      5.763855000     -2.426899000
H       -2.864144000      6.353736000     -2.174147000
H       -0.707178000      6.702946000     -3.388449000


68
B-Ph_Me
C        0.608307000      1.315641000      1.102547000
Br       1.865776000     -0.179016000      0.748293000
N        2.168031000      2.758088000     -3.003421000
C       -1.619057000      2.756345000      1.308640000
C        1.329081000      2.627931000      0.868785000
C       -2.845826000     -0.177079000      0.253301000
C       -1.390101000     -0.196253000      0.667028000
C       -2.551451000      2.211810000      0.469004000
C       -3.469094000      1.097840000      0.840366000
H       -1.301317000     -0.336488000      1.755714000
H       -0.869846000     -1.044446000      0.189288000
H       -2.949341000     -0.176810000     -0.843492000
H       -3.359848000     -1.072373000      0.629411000
H       -2.665912000      2.628328000     -0.537882000
H       -3.471794000      0.995590000      1.939692000
H        0.368836000      1.175618000      2.164348000
C       -0.851721000      4.050520000      1.148922000
H       -1.567050000      2.338382000      2.321708000
C        0.616485000      3.795179000      1.539556000
H        1.490053000      2.778340000     -0.208803000
H        2.327065000      2.549017000      1.322586000
H        0.632832000      3.613781000      2.626595000
H        1.190824000      4.719507000      1.377471000
C       -0.618621000      1.026749000      0.250716000
C       -0.489830000      1.300642000     -1.205813000
H       -1.353779000      0.930118000     -1.769176000
H       -0.346454000      2.365524000     -1.426190000
H        0.423113000      0.801369000     -1.599191000
C       -4.904780000      1.381371000      0.363298000
H       -4.857286000      1.543040000     -0.728397000
C       -5.830958000      0.203784000      0.635951000
H       -5.850888000     -0.044444000      1.710619000
H       -6.860435000      0.450455000      0.332831000
H       -5.533672000     -0.700373000      0.084618000
C       -5.442258000      2.653405000      1.008517000
H       -5.483855000      2.549686000      2.105955000
H       -4.814172000      3.528667000      0.779659000
H       -6.460576000      2.874945000      0.653739000
C        2.431032000      1.513980000     -3.467310000
C        3.484685000      1.533731000     -4.582082000
C        3.811945000      3.007007000     -4.724048000
C        2.906382000      3.671910000     -3.678656000
H        3.058188000      1.075741000     -5.488520000
H        4.338409000      0.906613000     -4.280080000
H        3.584129000      3.427851000     -5.716100000
H        4.861049000      3.260483000     -4.503821000
O        2.880852000      4.889433000     -3.509821000
O        1.910967000      0.469323000     -3.077098000
C       -1.440609000      4.983910000      2.233081000
H       -2.504292000      5.196411000      2.059548000
H       -1.336619000      4.534045000      3.232204000
H       -0.889983000      5.936899000      2.223669000
C       -4.487189000      3.539457000     -3.812230000
H       -5.057385000      3.135223000     -4.656009000
H       -3.973968000      4.467437000     -4.086940000
Cl      -3.258198000      2.346859000     -3.359070000
Cl      -5.621520000      3.896743000     -2.499218000
C       -1.032058000      4.720110000     -0.213857000
C        0.042236000      4.988961000     -1.067880000
C       -2.308394000      5.137550000     -0.626161000
C       -0.143923000      5.618086000     -2.300285000
C       -2.500212000      5.779763000     -1.846757000
C       -1.417752000      6.017309000     -2.695648000
H        1.057212000      4.698936000     -0.799891000
H       -3.175520000      4.962198000      0.014818000
H        0.732926000      5.761278000     -2.938880000
H       -3.507591000      6.088531000     -2.136538000
H       -1.570350000      6.511044000     -3.659047000


68
BC-Ph_Me
```

C	1.043128000	1.996030000	1.375652000
Br	2.670291000	0.904827000	1.049533000
N	-0.909524000	-3.131358000	-0.614497000
C	-1.505703000	2.926455000	1.357212000
C	1.435613000	3.459763000	1.319139000
C	-1.901680000	-0.112698000	0.074210000
C	-0.520166000	0.134888000	0.635370000
C	-2.241614000	2.246028000	0.431686000
C	-2.867220000	0.922586000	0.675453000
H	-0.484225000	-0.093329000	1.711950000
H	0.179063000	-0.573010000	0.107074000
H	-1.903022000	-0.027078000	-1.024428000
H	-2.204030000	-1.142709000	0.299102000
H	-2.368822000	2.666711000	-0.571563000
H	-2.920935000	0.745108000	1.763791000
H	0.748150000	1.699404000	2.390301000
C	-1.017737000	4.360297000	1.324557000
H	-1.448212000	2.478869000	2.357608000
C	0.393319000	4.382363000	1.936632000
H	1.679661000	3.735896000	0.283721000
H	2.363106000	3.576345000	1.897371000
H	0.288045000	4.107198000	2.998878000
H	0.773027000	5.415147000	1.930692000
C	0.020867000	1.487211000	0.377792000
C	0.203277000	1.904773000	-1.044026000
H	-0.540784000	1.447356000	-1.704938000
H	0.193516000	2.993238000	-1.178465000
H	1.197637000	1.539407000	-1.354851000
C	-4.287765000	0.852601000	0.085806000
H	-4.200182000	1.089961000	-0.989601000
C	-4.889820000	-0.539628000	0.223047000
H	-4.928578000	-0.850403000	1.280748000
H	-5.920988000	-0.549426000	-0.163611000
H	-4.322145000	-1.302234000	-0.330175000
C	-5.187631000	1.900357000	0.730687000
H	-5.292710000	1.713502000	1.812765000
H	-4.786161000	2.918842000	0.605837000
H	-6.194906000	1.883541000	0.286943000
C	-1.428086000	-4.307939000	-1.058030000
C	-0.511289000	-4.967945000	-2.098336000
C	0.631791000	-3.981521000	-2.233447000
C	0.250385000	-2.871745000	-1.248452000
H	-1.079859000	-5.131978000	-3.027253000
H	-0.208288000	-5.961010000	-1.730340000
H	0.739715000	-3.547800000	-3.240228000
H	1.617172000	-4.386873000	-1.954227000
O	0.957622000	-1.870452000	-1.091224000
O	-2.488851000	-4.802715000	-0.697686000
C	-1.941945000	5.116627000	2.306547000
H	-2.984061000	5.125235000	1.958607000
H	-1.914587000	4.650907000	3.303694000
H	-1.598351000	6.158112000	2.401992000
C	-4.414986000	2.402740000	-3.857949000
H	-4.793689000	1.375047000	-3.831108000
H	-4.548400000	2.847750000	-4.850450000
Cl	-2.678241000	2.365487000	-3.504948000
Cl	-5.339064000	3.365321000	-2.692308000
C	-1.120810000	5.039393000	-0.041741000
C	-0.023904000	5.649832000	-0.664076000
C	-2.364435000	5.129810000	-0.688311000
C	-0.154843000	6.291488000	-1.896953000
C	-2.498231000	5.762105000	-1.921894000
C	-1.390233000	6.343172000	-2.537496000
H	0.958093000	5.639247000	-0.191611000
H	-3.254965000	4.699509000	-0.225180000
H	0.722634000	6.754209000	-2.356057000
H	-3.477069000	5.791170000	-2.405567000
H	-1.491696000	6.838252000	-3.506540000

68

C-Ph_Me

C	1.017633000	1.521229000	0.578426000
Br	2.267674000	0.212176000	-0.197899000
N	1.625622000	3.492568000	-3.839853000
C	-1.267046000	2.374085000	0.801664000
C	1.547674000	2.934945000	0.436929000
C	-2.581280000	0.316531000	0.213898000
C	-1.082909000	0.068190000	0.360073000
C	-2.680157000	1.970052000	0.448148000
C	-3.562687000	1.411160000	1.379185000
H	-0.852120000	-0.215365000	1.397856000
H	-0.795442000	-0.769155000	-0.292160000
H	-3.025107000	0.201497000	-0.782274000
H	-3.147909000	-0.390254000	0.856431000
H	-3.123692000	2.393484000	-0.456085000
H	-3.168299000	1.170291000	2.375593000
H	0.988139000	1.235139000	1.640511000
C	-0.892471000	3.876068000	0.761829000
H	-1.122965000	2.088535000	1.858253000
C	0.609706000	3.927295000	1.131227000
H	1.717463000	3.189681000	-0.620460000
H	2.534245000	2.999244000	0.920942000
H	0.666513000	3.731861000	2.215069000
H	0.986837000	4.951789000	0.989786000
C	-0.383319000	1.383761000	-0.001993000
C	-0.427014000	1.595915000	-1.512601000
H	-1.455081000	1.706784000	-1.881853000
H	0.130031000	2.478866000	-1.846537000
H	0.014634000	0.727199000	-2.022623000
C	-5.037377000	1.332790000	1.181136000
H	-5.237084000	1.299113000	0.097350000
C	-5.666891000	0.132800000	1.870352000
H	-5.470414000	0.152206000	2.954088000
H	-6.756558000	0.140753000	1.721894000
H	-5.280933000	-0.816440000	1.467098000
C	-5.584456000	2.662468000	1.721615000
H	-5.355310000	2.785245000	2.791692000
H	-5.169150000	3.521324000	1.173914000
H	-6.678192000	2.673622000	1.604148000
C	1.271126000	3.712465000	-5.130917000
C	1.953764000	4.967226000	-5.696234000
C	2.826926000	5.436106000	-4.550022000
C	2.470552000	4.460261000	-3.420880000
H	1.172465000	5.690008000	-5.982453000
H	2.499465000	4.705978000	-6.616320000
H	2.634187000	6.467270000	-4.215080000
H	3.907466000	5.363803000	-4.754157000
O	2.915834000	4.588343000	-2.279140000
O	0.494109000	3.032921000	-5.794768000
C	-1.655688000	4.583838000	1.894122000
H	-2.745414000	4.545526000	1.751357000
H	-1.427695000	4.112635000	2.863166000
H	-1.361485000	5.643241000	1.950770000
C	-3.526332000	1.154949000	-4.178981000
H	-3.225663000	0.185436000	-3.765894000
H	-4.212707000	1.024183000	-5.023365000
Cl	-2.088899000	2.012233000	-4.732372000
Cl	-4.423154000	2.028635000	-2.905139000
C	-1.233339000	4.584553000	-0.553831000
C	-0.253235000	5.097697000	-1.412386000
C	-2.576626000	4.801278000	-0.910779000
C	-0.599710000	5.769878000	-2.586010000
C	-2.926000000	5.456541000	-2.089129000
C	-1.934590000	5.944189000	-2.939636000
H	0.809243000	4.964951000	-1.203211000
H	-3.381600000	4.467268000	-0.253552000
H	0.192176000	6.151472000	-3.234535000
H	-3.981785000	5.589543000	-2.338738000
H	-2.200909000	6.460381000	-3.865514000

53

A-Me-Br

C	-3.672395000	0.721765000	-0.491614000
C	-2.400911000	-0.115934000	-0.272261000
C	-2.980572000	2.799784000	0.739167000
C	-4.038674000	1.742093000	0.603042000
H	-2.472085000	-0.639745000	0.694951000
H	-2.375337000	-0.892712000	-1.056764000
H	-3.610874000	1.246608000	-1.461303000
H	-4.517693000	0.019577000	-0.587898000
H	-2.803699000	3.435454000	-0.137479000
H	-4.099043000	1.196219000	1.562167000
C	-0.399532000	0.874806000	0.806249000
C	-2.167879000	2.921596000	1.789308000
C	0.676191000	1.886709000	1.042216000
H	-0.740883000	0.345313000	1.704915000
C	-0.939395000	3.787571000	1.866304000
H	-2.318775000	2.273555000	2.661434000
C	0.299268000	2.900994000	2.131213000
H	0.928819000	2.417863000	0.115274000
H	1.601000000	1.382638000	1.373221000
H	0.144494000	2.374232000	3.088175000
H	1.159927000	3.574371000	2.287158000
C	-1.124565000	0.683788000	-0.320881000
C	-0.829971000	1.366631000	-1.620226000
H	-1.020559000	0.687326000	-2.466500000
H	-1.494115000	2.237398000	-1.757414000
H	0.205805000	1.720362000	-1.693917000
C	-5.404466000	2.357564000	0.306816000
H	-5.384221000	2.919113000	-0.642320000
H	-6.182971000	1.582482000	0.219944000
H	-5.706744000	3.056924000	1.102012000
N	2.068870000	-2.784805000	-0.776946000
C	3.432614000	-2.759828000	-0.513637000
C	4.001085000	-4.070001000	-1.011929000
C	2.817967000	-4.840542000	-1.584867000
C	1.615003000	-3.943370000	-1.394373000
H	4.489871000	-4.576765000	-0.166559000
H	4.782881000	-3.846948000	-1.753127000
H	2.621335000	-5.793190000	-1.070799000
H	2.914626000	-5.063917000	-2.657794000
O	0.472253000	-4.160653000	-1.706265000
O	4.019877000	-1.850431000	0.014028000
Br	0.925303000	-1.357068000	-0.328821000
O	-0.803768000	4.478923000	0.637394000
H	-0.066975000	5.097596000	0.737298000
C	-1.084721000	4.799275000	3.003848000
H	-1.945331000	5.457281000	2.810465000
H	-1.239411000	4.297609000	3.971006000
H	-0.177266000	5.421002000	3.084999000
C	1.311386000	4.682214000	-1.813017000
H	0.404974000	4.216737000	-1.407504000

H	1.093862000	5.314321000	-2.680984000
Cl	2.434264000	3.408790000	-2.313576000
Cl	1.992947000	5.716203000	-0.535454000

53
AB-Me-Br

C	-3.562735000	0.180434000	-0.107088000
C	-2.153106000	-0.111683000	0.357279000
C	-3.023842000	2.474468000	0.474863000
C	-4.091314000	1.453469000	0.597089000
H	-2.141908000	-0.401189000	1.419851000
H	-1.737584000	-0.967031000	-0.204964000
H	-3.599487000	0.323310000	-1.198396000
H	-4.231872000	-0.661075000	0.127463000
H	-3.036500000	3.149554000	-0.388846000
H	-4.208596000	1.203259000	1.664368000
C	0.085916000	0.927135000	0.945279000
C	-1.955612000	2.552680000	1.330004000
C	0.984397000	2.147237000	0.968710000
H	-0.150920000	0.600276000	1.967109000
C	-0.995967000	3.723461000	1.360561000
H	-1.997881000	1.973315000	2.258317000
C	0.393753000	3.266970000	1.810774000
H	1.189904000	2.487998000	-0.057172000
H	1.954223000	1.853124000	1.394791000
H	0.325353000	2.937882000	2.860735000
H	1.063301000	4.141574000	1.797363000
C	-1.177345000	1.016557000	0.137187000
C	-1.015937000	1.498598000	-1.277622000
H	-1.952653000	1.462429000	-1.845877000
H	-0.619079000	2.519033000	-1.315744000
H	-0.293218000	0.823034000	-1.763523000
C	-5.419597000	1.917499000	0.016863000
H	-5.312862000	2.178220000	-1.047951000
H	-6.169683000	1.116812000	0.098218000
H	-5.804964000	2.799821000	0.549235000
N	2.187189000	-2.745514000	-0.811823000
C	3.465621000	-3.200776000	-0.783385000
C	3.559018000	-4.591962000	-1.427928000
C	2.131360000	-4.893606000	-1.840186000
C	1.355077000	-3.646944000	-1.392144000
H	3.975113000	-5.297747000	-0.691720000
H	4.271958000	-4.548680000	-2.266548000
H	1.697072000	-5.779238000	-1.349787000
H	1.993159000	-5.029784000	-2.924508000
O	0.147760000	-3.521237000	-1.555122000
O	4.435263000	-2.616232000	-0.320045000
Br	1.076402000	-0.650649000	0.166473000
O	-0.961376000	4.285237000	0.063925000
H	-0.563315000	5.165380000	0.132926000
C	-1.562840000	4.739563000	2.353762000
H	-2.541821000	5.102819000	2.006967000
H	-1.682089000	4.302820000	3.355774000
H	-0.874712000	5.596453000	2.435664000
C	2.167040000	5.404323000	-0.924716000
H	1.468023000	4.584916000	-1.125944000
H	2.591915000	5.806682000	-1.850827000
Cl	3.482571000	4.796005000	0.084353000
Cl	1.252833000	6.713436000	-0.133790000

53
B-Me-Br

C	-1.329718000	-0.133968000	-0.033891000
C	-0.167698000	0.148719000	0.915468000
C	-2.037172000	1.405699000	-0.180844000
C	-3.053888000	0.548457000	0.249066000
H	-0.480403000	-0.038372000	1.953905000
H	0.657286000	-0.540312000	0.680152000
H	-1.087414000	-0.349174000	-1.081239000
H	-1.903486000	-1.016566000	0.309050000
H	-2.032021000	1.692435000	-1.236779000
H	-3.192332000	0.430999000	1.331083000
C	0.947885000	2.270507000	1.881700000
C	-1.224318000	2.246335000	0.767607000
C	1.000391000	3.783539000	1.741755000
H	0.450024000	1.997873000	2.824079000
C	-1.293826000	3.764879000	0.569344000
H	-1.606663000	2.050425000	1.783803000
C	-0.409101000	4.370678000	1.679102000
H	1.563405000	4.057941000	0.837313000
H	1.541963000	4.211862000	2.598401000
H	-0.910975000	4.217616000	2.649385000
H	-0.344844000	5.457340000	1.516306000
C	0.197781000	1.624807000	0.721431000
C	0.928607000	1.794548000	-0.612052000
H	0.328660000	1.417667000	-1.452455000
H	1.142196000	2.845070000	-0.831174000
H	1.867242000	1.221165000	-0.585376000
C	-4.070022000	-0.041779000	-0.646262000
H	-3.757410000	-0.026008000	-1.698326000
H	-4.360108000	-1.053880000	-0.333357000
H	-4.961125000	0.603699000	-0.548190000
N	-0.296015000	6.650966000	-0.909762000
C	-1.121117000	7.715757000	-0.740398000
C	-0.401822000	9.024415000	-1.078099000
C	1.000187000	8.576231000	-1.446981000
C	0.942727000	7.052896000	-1.301748000
H	-0.447506000	9.694268000	-0.205326000
H	-0.942276000	9.527762000	-1.895175000
H	1.786556000	8.968315000	-0.783034000
H	1.298315000	8.826574000	-2.477131000
O	1.898812000	6.317919000	-1.511904000
O	-2.286254000	7.666991000	-0.362366000
Br	2.760223000	1.526988000	2.106482000
O	-0.814601000	4.064042000	-0.705504000
H	-0.652505000	5.064617000	-0.776624000
C	-2.730844000	4.267127000	0.718633000
H	-3.370085000	3.819835000	-0.058843000
H	-3.158693000	4.029665000	1.705803000
H	-2.749173000	5.359360000	0.571799000
C	-3.473044000	3.043071000	-4.402908000
H	-2.861793000	2.536324000	-5.158330000
H	-3.954227000	3.934187000	-4.822277000
Cl	-2.430910000	3.536120000	-3.061426000
Cl	-4.755195000	1.919869000	-3.893211000

43
cation-B-OH-Br-PBE0/Def2-SVP

C	1.733606000	-0.377895000	1.337477000
C	0.710022000	0.057236000	2.387398000
C	2.045507000	1.048375000	0.525256000
C	3.298591000	0.600901000	0.966859000
H	1.223401000	0.318435000	3.324844000
H	0.031626000	-0.783360000	2.594368000
H	1.394299000	-1.041519000	0.533536000
H	2.558120000	-0.941667000	1.824425000
H	1.810599000	0.915821000	-0.535203000
H	3.637042000	0.929140000	1.958916000
C	-0.659652000	2.208790000	2.820921000
C	1.238253000	2.067816000	1.292551000
C	-1.065900000	3.531970000	2.189689000
H	0.042922000	2.386860000	3.648485000
C	0.962728000	3.402906000	0.602758000
H	1.818985000	2.324403000	2.193982000
C	0.144959000	4.248838000	1.593218000
H	-1.825514000	3.355085000	1.413539000
H	-1.531907000	4.174900000	2.950728000
H	0.816561000	4.567473000	2.407233000
H	-0.184544000	5.167308000	1.080093000
C	0.000546000	1.284472000	1.802220000
C	-0.965132000	0.833566000	0.705365000
H	-0.453007000	0.239734000	-0.064905000
H	-1.436086000	1.678679000	0.193364000
H	-1.746270000	0.199041000	1.148949000
C	4.286090000	-0.101575000	0.102607000
H	3.741528000	-0.573636000	-0.731668000
C	5.108901000	-1.137711000	0.851941000
H	5.644167000	-0.680254000	1.699044000
H	5.855902000	-1.582696000	0.178655000
H	4.480706000	-1.954220000	1.240581000
C	5.166409000	1.018523000	-0.474524000
H	5.696493000	1.561305000	0.323553000
H	4.576492000	1.741047000	-1.057103000
H	5.919478000	0.570532000	-1.139542000
Br	-2.198373000	1.364559000	3.709892000
O	0.232807000	3.136677000	-0.579556000
H	0.126200000	3.973984000	-1.051398000
C	2.266487000	4.109348000	0.250904000
H	2.830367000	3.528724000	-0.495182000
H	2.902674000	4.255307000	1.136560000
H	2.053524000	5.100996000	-0.180561000

43
cation-B-OH-Br-PBE0/Def2-TZVP

C	1.733129000	-0.386875000	1.364671000
C	0.705547000	0.066787000	2.397434000
C	2.043009000	1.047000000	0.527459000
C	3.301939000	0.622875000	0.950388000
H	1.210294000	0.337816000	3.327645000
H	0.030474000	-0.765787000	2.605891000
H	1.391620000	-1.038084000	0.564036000
H	2.550650000	-0.935068000	1.858366000
H	1.793563000	0.888251000	-0.516443000
H	3.646666000	0.952589000	1.929641000
C	-0.659492000	2.209135000	2.807249000
C	1.236380000	2.062775000	1.291116000
C	-1.061447000	3.529966000	2.178921000
H	0.029615000	2.379817000	3.636483000
C	0.969938000	3.398203000	0.607946000
H	1.811335000	2.315291000	2.189416000
C	0.155232000	4.242342000	1.594105000
H	-1.811623000	3.360236000	1.403720000
H	-1.522470000	4.168542000	2.934815000
H	0.820937000	4.546806000	2.408196000
H	-0.164365000	5.158180000	1.087451000
C	-0.000858000	1.282938000	1.797944000
C	-0.957836000	0.820093000	0.703851000
H	-0.432650000	0.276795000	-0.083144000
H	-1.475490000	1.648764000	0.228380000
H	-1.697432000	0.143329000	1.135759000

C	4.272982000	-0.092998000	0.090745000
H	3.730532000	-0.564763000	-0.733961000
C	5.096705000	-1.120429000	0.846294000
H	5.625026000	-0.656610000	1.683693000
H	5.839749000	-1.560463000	0.178628000
H	4.475005000	-1.929836000	1.236244000
C	5.162375000	1.017620000	-0.489700000
H	5.685955000	1.557044000	0.303668000
H	4.583122000	1.732461000	-1.076626000
H	5.909366000	0.558185000	-1.139888000
Br	-2.212316000	1.374384000	3.685554000
O	0.239640000	3.146546000	-0.585959000
H	0.155254000	3.984456000	-1.056411000
C	2.274929000	4.100056000	0.269434000
H	2.840526000	3.526934000	-0.469039000
H	2.895845000	4.234386000	1.158003000
H	2.068247000	5.088529000	-0.151710000

43

cation-B-OH-Br-B3LYP/Def2-TZVP

C	1.640795000	-0.469790000	1.459249000
C	0.574698000	0.022359000	2.444153000
C	2.017203000	0.936148000	0.595723000
C	3.350992000	0.656565000	0.922425000
H	1.039224000	0.276598000	3.397245000
H	-0.139209000	-0.783827000	2.616967000
H	1.307860000	-1.142517000	0.675852000
H	2.463732000	-0.966353000	1.980048000
H	1.741525000	0.768861000	-0.438540000
H	3.717077000	1.002421000	1.887127000
C	-0.717858000	2.229493000	2.795999000
C	1.225716000	1.997323000	1.334988000
C	-1.056812000	3.564669000	2.150623000
H	-0.058153000	2.376929000	3.648775000
C	1.032217000	3.346142000	0.640937000
H	1.784491000	2.228708000	2.245968000
C	0.207560000	4.231564000	1.592810000
H	-1.789913000	3.420889000	1.357265000
H	-1.509831000	4.224615000	2.890369000
H	0.853608000	4.525357000	2.424045000
H	-0.067476000	5.148263000	1.065509000
C	-0.063364000	1.266454000	1.811793000
C	-1.008208000	0.833617000	0.684298000
H	-0.482912000	0.256038000	-0.075162000
H	-1.468157000	1.678314000	0.182842000
H	-1.792402000	0.199210000	1.096759000
C	4.303076000	-0.046167000	0.052759000
H	3.758489000	-0.566896000	-0.736881000
C	5.219349000	-1.003848000	0.810339000
H	5.772079000	-0.477707000	1.591335000
H	5.938411000	-1.444767000	0.119443000
H	4.649485000	-1.810541000	1.273340000
C	5.115887000	1.103640000	-0.609728000
H	5.632362000	1.705348000	0.139241000
H	4.477980000	1.750809000	-1.209745000
H	5.861294000	0.642154000	-1.258234000
Br	-2.349628000	1.448277000	3.652930000
O	0.329951000	3.114251000	-0.589151000
H	0.270855000	3.957776000	-1.058061000
C	2.375745000	4.003732000	0.338980000
H	2.942450000	3.408915000	-0.378416000
H	2.972390000	4.119498000	1.245188000
H	2.217030000	4.995693000	-0.091140000

43

cation-B-OH-Br-MP2/Def2-TZVP

C	1.751552000	-0.398541000	1.357212000
C	0.750363000	0.085570000	2.400611000
C	2.071592000	1.118645000	0.505457000
C	3.283252000	0.610533000	0.964761000
H	1.280866000	0.370864000	3.314136000
H	0.075924000	-0.741794000	2.637840000
H	1.369961000	-0.964856000	0.509830000
H	2.542365000	-1.010939000	1.812305000
H	1.816341000	0.948350000	-0.537213000
H	3.623368000	0.924705000	1.952949000
C	-0.647227000	2.204607000	2.809227000
C	1.254303000	2.095294000	1.295380000
C	-1.068509000	3.524874000	2.181044000
H	0.040745000	2.387039000	3.641284000
C	0.956109000	3.423861000	0.611407000
H	1.832916000	2.352940000	2.193253000
C	0.145051000	4.258425000	1.606642000
H	-1.810466000	3.345025000	1.400207000
H	-1.544118000	4.149979000	2.940165000
H	0.811531000	4.556027000	2.423799000
H	-0.179863000	5.176494000	1.106038000
C	0.027680000	1.291959000	1.797148000
C	-0.922786000	0.808304000	0.700452000
H	-0.380512000	0.357769000	-0.132147000
H	-1.523906000	1.614564000	0.289153000
H	-1.587246000	0.048888000	1.116986000
C	4.254738000	-0.112884000	0.098868000
H	3.707835000	-0.573936000	-0.730160000
C	5.061379000	-1.156025000	0.859558000
H	5.580004000	-0.694609000	1.703354000
H	5.809532000	-1.595121000	0.198542000
H	4.433451000	-1.964998000	1.237940000
C	5.170172000	0.985477000	-0.466614000
H	5.689883000	1.506774000	0.340884000
H	4.604740000	1.714594000	-1.049137000
H	5.917659000	0.524031000	-1.113493000
Br	-2.179554000	1.339435000	3.652084000
O	0.200860000	3.140887000	-0.567425000
H	0.117427000	3.974179000	-1.057741000
C	2.248238000	4.137680000	0.241823000
H	2.809136000	3.556520000	-0.494094000
H	2.875673000	4.293394000	1.121972000
H	2.019147000	5.114232000	-0.194021000

3. Isolation of germacrene d

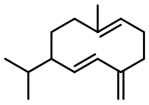

germacrene d

Gemacrene d was obtained from ylang ylang oil (Farfalla GmbH, ‘Ylang Ylang Complete organic Grand Cru, essential oil’, batch number #03.2026/032130) by the following protocol:

1.0 g of ylang ylang oil was purified by flash column chromatography over 5% $AgNO_3$-impregnated silica (40 g, CombiFlash), using a gradient of 0 – 50% Et_2O in pentane over 15 column volumes at a rate of 8 mL/min. Tubes were collected at a volume of 15 mL and analysed by GC-MS (Thermoscientific DSQ II, Optima® – 5 – accent – 0.25 mm, He); tubes that contained germacrene d with greater than 85% purity (Rt = 15.4 min) were pooled and concentrated by rotary evaporation. The resulting residue was 128 mg of germacrene d with a purity of 87%; with the major contaminant being carophyllene b.

Both enantiomeric forms of germacrene d are found in nature. Since our sample was not totally pure, it was not possible to judge the stereochemical composition of the mixture by comparison of the optical rotation to a known value.

4. Expression and purification of germacradien-4-ol synthase

The DNA sequence of N-terminal His_{10}-tagged Germacradien-4-ol synthase (GdolS, gene name: SC1) from *Streptomyces citricolor* was ordered in a pET-21 a (+) plasmid from BioCat GmbH (69120 Heidelberg, Germany) and transformed into *E.coli* strain Rosetta II using heat shock transformation. Briefly, 5 µg of plasmid were dissolved in 25 µL of double-distilled water (ddH_2O) and 5 µL of the plasmid solution was added to 100 µL of a solution of *E.coli* Rosetta II competent cells. After an incubation on ice for 10 minutes cells heat shocked at 42°C for 90 seconds before another incubation on ice for 5 minutes. After the addition of 1 mL of lysogeny broth (LB) medium the cells were allowed to recover at 37°C with gentle shaking (300 rpm) on a thermomixer before the solution was centrifuged for 2 minutes at 10 000 g. The cell pellet was resuspended in 100 µL of LB medium and the solution plated on a LB/agar plate treated with ampicillin (100 µg/mL) and chloramphenicol (30 µg/mL). The plate was incubated at 37°C overnight and a single colony was picked for the inoculation of 3 mL LB medium containing ampicillin (100 µg/mL) and chloramphenicol (30 µg/mL). After incubation overnight at 37°C and 170 rpm, the culture was treated with glycerol (final concentration 20%, v/v) and stored at -80°C as cell stock solution.

GdolS was expressed in ampicillin (100 µg/mL) and chloramphenicol (30 µg/mL) supplemented LB medium by inoculating the media to a starting OD of 0.2 using an overnight culture following an incubation at 37°C at 170 rpm. Induction was initiated at an OD of 0.7-0.8 using 0.5 mM isopropyl-*β*-D-thiogalactopyranosid (IPTG) following an incubation at 37°C, 170 rpm for 3h. Subsequently, cells were harvested at 10 000 g for 20 minutes at 4°C and cell pellets stored at -80°C before continuing with the cell workup. After thawing on ice, TBS buffer (50 mM Tris, 150 mM NaCl pH 7.5) was added to the cell

pellets (70 mL buffer for 1L LB media cell pellet) and the solution homogenized using an ultra turrax. The cell solution was submitted for lysis using a cell disruptor (Constant Systems, Northants, United Kingdom) at 1.9 kbar for three cycles before separating the cell debris from the soluble protein fraction via centrifugation at 50 000 g for 45 min at 4°C. The supernatant was loaded on TBS equilibrated 5mL Ni-sepharose column (Cytiva) using an ÄKTAprime plus system. The column was washed with 20 mM imidazole in TBS buffer and the protein eluted using a linear gradient from 20 to 500 mM imidazole in 10 column volumes. Fractions containing the target protein were pooled and dialysed into TBS, pH 8 at 4°C over-night. The protein solution was concentrated using Amicon Ultra-15 centrifugal filters (10 kDa molecular weight cut-off) and stored at -80°C upon the addition of 10 % glycerol (v/v).

Protein concentration was measured using a NanoDrop 2000c and the yield determined as 45 mg / liter cell culture. Final analysis of the protein was performed by Electrospray ionization mass spectrometry (ESI-MS) (Figure S4) and analytical reversed phase high pressure liquid chromatography (RP-HPLC) (Figure S5).

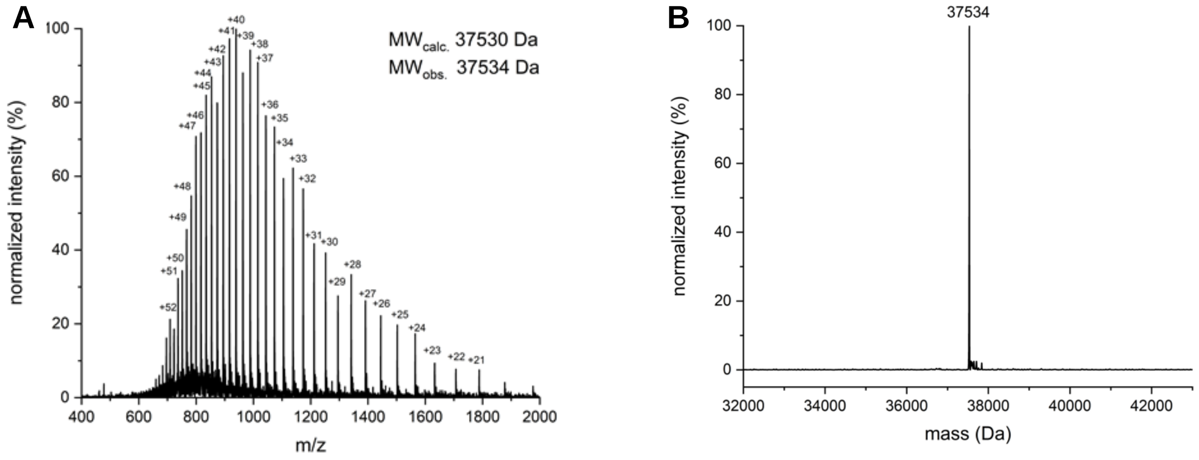

Figure S4. A) ESI-MS spectrum of GdolS after purification via immobilized metal affinity chromatography (Ni-NTA); B) Deconvoluted ESI-MS spectrum of GdolS.

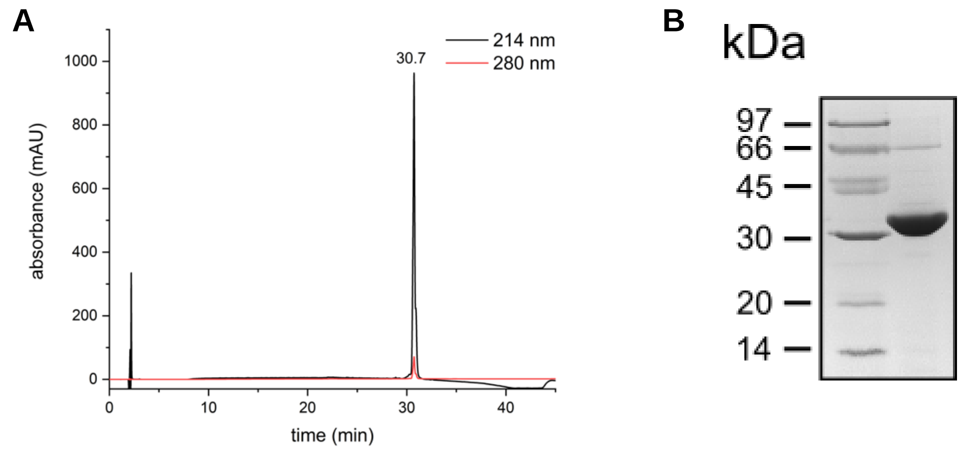

Figure S5. A) RP-HPLC characterization of recombinant GdolS using a linear gradient from 5 - 65% buffer B (MeCN + 0.08% TFA) in buffer A (ddH2O + 0.1 % TFA) on a C4 column (300-5-C4, 150 x 4.6 mm, 5 um particle size, Kromasil); B) SDS-PAGE analysis of purified *GdolS*

Amino acid sequence of His10-GdolS

MHHHHHHHHHHSDDTSLELP FTHRRNPHQT EAADRHLEWL QRHRELAAVV SGSTYTGWDI TELASLVYPE SSAEDLALAA DLMGFYFLFD DQFDSPLGRR PEQVALICER LSAIAHGTLT AVTSPSERAF ADLWRRITLG MTDRWRARAA CNWEYYFACH PAEAAGRTIG QPPDREGYLT LRRGTAAMES IFDMIERLGH FEVPQHVMHH PLFRQLRQLA ADIPSFTNDV RSFAQESERG DVANLVMIVR RDRCCSTEEA CAVVWDEAQR MADRFCDLRD QLPDACRSMS LDPAQRLAAE RYADGMALWL AGYLHWESHT RRYHHG

DNA Sequence of His10-GdolS

CACCACCACCACCACCACCACCACCACCACTCTGACGACACCTCTCTGGA
ACTGCCGTTCACCCACCGTCGTAACCCGCACCAGACCGAAGCTGCTGACC
GTCACCTGGAATGGCTGCAGCGTCACCGTGAACTGGCTGCTGTTGTTTCT
GGTTCTACCTACACCGGTTGGGACATCACCGAACTGGCTTCTCTGGTTTA
CCCGGAATCTTCTGCTGAAGACCTGGCTCTGGCTGCTGACCTGATGGGTT
TCTACTTCCTGTTCGACGACCAGTTCGACTCTCCGCTGGGTCGTCGTCCG
GAACAGGTTGCTCTGATCTGCGAACGTCTGTCTGCTATCGCTCACGGTAC
CCTGACCGCTGTTACCTCTCCGTCTGAACGTGCTTTCGCTGACCTGTGGC
GTCGTATCACCCTGGGTATGACCGACCGTTGGCGTGCTCGTGCTGCTTGC
AACTGGGAATACTACTTCGCTTGCCACCCGGCTGAAGCTGCTGGTCGTAC
CATCGGTCAGCCGCCGGACCGTGAAGGTTACCTGACCCTGCGTCGTGGTA
CCGCTGCTATGGAATCTATCTTCGACATGATCGAACGTCTGGGTCACTTC
GAAGTTCCGCAGCACGTTATGCACCACCCGCTGTTCCGTCAGCTGCGTCA
GCTGGCTGCTGACATCCCGTCTTTCACCAACGACGTTCGTTCTTTCGCTC
AGGAATCTGAACGTGGTGACGTTGCTAACCTGGTTATGATCGTTCGTCGT
GACCGTTGCTGCTCTACCGAAGAAGCTTGCGCTGTTGTTTGGGACGAAGC
TCAGCGTATGGCTGACCGTTTCTGCGACCTGCGTGACCAGCTGCCGGACG
CTTGCCGTTCTATGTCTCTGGACCCGGCTCAGCGTCTGGCTGCTGAACGT
TACGCTGACGGTATGGCTCTGTGGCTGGCTGGTTACCTGCACTGGGAATC
TCACACCCGTCGTTACCACCACGGT

5. Experimental Procedures

Bromide 6

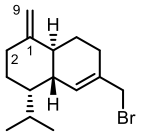

$\Delta^{1,9}$-**6**

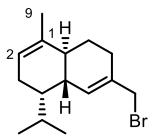

$\Delta^{1,2}$-**6**

To a solution of germacrene d (42.0 mg, 77% purity, 0.158 mmol) in DCM (6 mL) at –78 °C was added NBS (31.0 mg, 1.1 eq, 0.174 mmol) as a solution in DCM (0.5 mL). The resulting solution was stirred for 1 h at –78 °C, then allowed to warm to rt. The reaction mixture was then extracted with sat.aq. $Na_2S_2O_3$ (10 mL), and the organic phase was collected. The aqueous phase was extracted with DCM (2 × 5 mL), and the combined organic phases were dried over Na_2SO_4 and concentrated *in vacuo*. ^{1}H-NMR analysis of the crude oil showed $\Delta^{1,9}$-**6** and $\Delta^{1,2}$-**6** were formed in 22% and 8% yield, respectively (mesitylene internal standard). The crude oil was then purified by flash chromatography (pentane) to give $\Delta^{1,9}$-**6** and $\Delta^{1,2}$-**6** (3.2:1.0, 8.6 mg, 19%) as a clear oil mixture.

IR (film) ν_{max} 3001, 2958, 2864, 1712, 1456, 1222, 911 cm^{-1}; **^{1}H NMR** (600 MHz, $CDCl_3$) δ 6.07* (s, 0.3H), 6.02 (s, 1H), 5.41* (s, 0.3H), 4.69 (s, 1H), 4.57 (s, 1H), 4.02 – 3.94 (m, 2.6H), 2.43 – 2.36 (m, 1H), 2.19 (m, 3.6H), 2.08 – 1.98 (m, 2H), 1.89 (s, 1.3H), 1.79 (m, 2H), 1.68 (m, 2.3H), 1.53 – 1.48 (m, 1H), 1.32 – 1.23 (m, 1.6H), 1.13 (ddd, J = 25.5, 12.8, 4.1 Hz, 1H), 0.94 (d, J = 6.9 Hz, 3H), 0.91 (d, J = 6.9 Hz, 1H), 0.79* (d, J = 6.9 Hz, 1H), 0.75 (d, J = 6.9 Hz, 3H); **^{13}C NMR** (151 MHz, $CDCl_3$) δ 152.7†, 135.7*, 135.4*, 135.3†, 129.2*, 129.1†, 122.2*, 103.9†, 46.5†, 45.4†, 44.0†, 42.3*, 42.0*, 41.6*, 39.8*, 39.7†, 36.2†, 27.6*, 26.9†, 26.6†, 26.5*, 26.4†, 26.3*, 25.4†, 25.0*, 21.6†, 21.0*, 20.8*, 15.3†, 14.7*; **HRMS** (EI+) $[M]^+$ 282.0960 found, calculated 282.0983 for $C_{15}H_{23}Br^+$.

Signals arising exclusively from the minor isomer are indicated by an * and those arising solely from the major isomer are indicated by a †; undesignated signals arise from a mixture of both.

Farnesyl pyrophosphate (8)

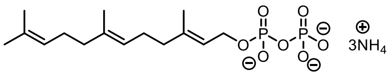

To a stirred solution of tris(tetra-n-butyl)ammonium hydrogen pyrophosphate (5.62 g, 6.23 mmol, 2.0 eq) in MeCN (22 mL) at rt was added geranyl chloride (750 mg, 3.11 mmol, 1.0 eq) in 3 mL of MeCN. The resulting solution was stirred at rt for 4 h, then concentrated *in vacuo*. The resulting residue was dissolved in 8.0 mL of 1:49 (v/v) iPrOH:25 mM NH_4HCO_3. The solution divided into two parts, and each portion was passed through a column containing 60 mL Dowex AG 50W-X8 cation exchange resin (ammonium form*, 60 mL) which had been equilibrated with two column volumes of 1:49 (v/v) iPrOH:25 mM NH_4HCO_3 buffer. The columns were eluted with 115 mL of the same buffer. The column eluents were pooled, adjusted to pH 8 by addition of aq. NaOH, then used directly in the following step.

* Dowex AG 50W-X8 H^+ form was converted to the ammonium form by treatment with aq. NH_3OH (~25%, two column volumes), followed by water (two column volumes).

This procotol was adapted from procedure of Polter et al.[20]

Germacradien-4-ol (7)

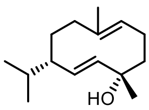

To a solution of aq. HEPES buffer (50 mM, pH 8, 3000 mL) containing $MgCl_2$ (2.5 mM) was added mercaptoethanol (1.31 mL, 5 mM resulting concentration); this solution was split into two large culture flasks. A solution of *GdolS* in HEPES buffer (10 mL *each*, c = 7 mg/mL, pH 8, 0.12 mol %) was added to both flasks, and the resulting solution was warmed to 37 °C in a water bath. The half of the solution of farnesyl pyrophosphate prepared in the previous step (≤1.35 g, ≤3.11 mmol, ~230 mL in H_2O, 8 pH) was added to each flask. The resulting solutions were covered by a layer of hexane (100 mL) and shaken in an incubator at 37 °C for 1 h. Upon cooling to rt, NaCl (100 g) was added and the solution was stirred until it completely dissolved. The organic phase was separated, and the aqueous phase was extracted with diethyl ether (2 x 500 mL). The combined organic were washed with brine and concentrated *in vacuo*. The crude material was purified by flash chromatography (0 – 20% Et_2O in pentane) to give germacradien-4-ol (**7**) (295 mg, 43%) as a clear oil.

$[\alpha]_D^{20}$ = +196.5 (*c* = 0.2, C_6D_6) ; **^{1}H NMR** (400 MHz, $CDCl_3$) δ 5.20 (dt, *J* = 15.7, 12.5 Hz, 1H), 4.95 (d, *J* = 11.4 Hz, 2H), 2.58 – 2.42 (m, 1H), 2.31 – 2.18 (m, 2H), 2.05 – 1.90 (m, 2H), 1.64 (dt, *J* = 14.0, 3.8 Hz, 1H), 1.57 (d, *J* = 3.8 Hz, 1H), 1.54 (s, 3H), 1.47 – 1.35 (m, 4H), 1.19 (s, 3H), 0.83 (d, *J* = 6.7 Hz, 3H), 0.79 (d, *J* = 6.8 Hz, 3H).

Based on the yield and loading of *GdolS*, the total number of turnovers under these conditions is 360 (assuming quantitative yield of the preceding diphosphorylation step).

The spectra matched those reported in the literature.[21]

Germacradien-4-methyloxy (17)

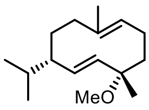

To NaH (25.2 mg, 60% dispersion in mineral oil, 0.63 mmol, 4.0 eq) in THF (1 mL) at rt was added germacradien-4-ol (**7**) (35.0 mg, 0.157 mmol, 1.0 eq) as a solution in THF (0.2 mL) dropwise. The resulting mixture was stirred for 20 min at rt, then methyl iodide (49 μL, 0.787 mmol, 5.0 eq) was added. The reaction mixture was then heated to 55 °C for 16 h, then allowed to cool to rt before addition slow addition of sat. aq. NH_4Cl (5 mL). The resulting suspension was extracted with DCM (3 × 5 mL), dried over Na_2SO_4, and concentrated *in vacuo.* The crude oil was purified by flash column chromatography (pentane) to give **17** (31.6 mg, 85%) as a clear oil.

$[\alpha]_D^{20}$ = +104 (*c* = 0.05, $CHCl_3$); **IR** (film) ν_{max} 2923, 2362, 1714, 1365, 1223, 736 cm^{-1}; **^{1}H NMR** (400 MHz, $CDCl_3$) δ 5.21 (dd, *J* = 15.8, 9.9 Hz, 1H), 4.94 (d, *J* = 15.4 Hz, 2H), 3.24 (s, 3H), 2.48 (dd, *J* = 24.1, 12.4 Hz, 1H), 2.26 – 2.19 (m, 2H), 2.01 – 1.91 (m, *J* = 14.3, 7.5 Hz, 1H), 1.91 – 1.75 (m, 2H), 1.53 (s, 3H), 1.48 – 1.34 (m, 4H), 1.12 (s, 3H), 0.84 (d, *J* = 6.7 Hz, 3H), 0.78 (d, *J* = 6.8 Hz, 3H); **^{13}C NMR** (101 MHz, $CDCl_3$) δ 136.1, 132.5, 129.7 (2C), 77.0, 52.9, 49.8, 41.3, 37.7, 33.3, 26.7, 24.8, 23.8, 20.8, 19.1, 16.7; **HRMS** (EI+) $[M]^+$ 236.2123 found, calculated 236.2140 for $C_{16}H_{28}O^+$.

6,8-cycloeudesmane 3

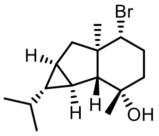

To a solution of NBS (48.0 mg, 1.1 eq, 0.27 mmol) in DCM (10 mL) at –78 °C was added germacradien-4-ol (50.0 mg, 0.225 mmol) as a solution in DCM (1 mL). The resulting solution was stirred for 1 h at –78 °C, then allowed to warm to rt. The reaction mixture was then extracted with sat. aq. $Na_2S_2O_3$ (10 mL), and the organic phase was collected. The aqueous phase was extracted with DCM (2 × 10 mL), and the combined organic phases were dried over Na_2SO_4 and concentrated *in vacuo*. The crude mixture was dissolved again in DCM (3 mL) and cooled to 0 °C. $NaHCO_3$ (37.8 mg, 0.45 mmol, 2.0 eq) and mCPBA (60.5 mg, 77% purity, 0.27 mmol, 1.2 eq) were then added, and the resulting mixture was stirred at 0 °C for 1 h. The mixture was then warmed to rt and sat. aq. $Na_2S_2O_3$ (10 mL) was added. The biphasic mixture was separated, and the aqueous phase was extracted with DCM (2 × 10 mL). The combined organic phases were washed with brine (20 mL), and dried over Na_2SO_4, then concentrated *in vacuo*. The resultant crude oil was purified by flash chromatography (15 – 40% Et_2O in heptane) to give **3** (23.7 mg, 35%) as a clear oil.

The absolute stereochemistry of **3** was not assigned in the isolation report, however, the optical rotation $[\alpha]_D^{20}$ = +10.0 (*c* = 0.13, MeOH) was given. We measured an optical rotation of $[\alpha]_D^{20}$ = –45 (*c* = 0.13, MeOH) indicating that the naturally occurring isomer can be assigned to the antipodal configuration as shown here.

Table S1. Comparison of ^{1}H-NMR data of natural and synthetic **3**[22]

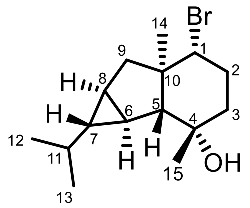

H	**natural** δ$_H$ (300 MHz, CDCl$_3$)	**synthetic** δ$_H$ (600 MHz, CDCl$_3$)	**Δδ$_H$**
1	3.86 (dd, *J* = 12.3, 4.1 Hz, 1H)	3.85 (dd, *J* = 12.4, 4.0 Hz, 1H)	-0.01
2a	1.95 (dddd, *J* = 13.9, 4.6, 4.1, 2.5 Hz, 1H)	1.95 (m, 1H)	0
2b	2.25 (dddd, *J* = 13.9, 13.9, 12.3, 4.1 Hz, 1H)	2.29 – 2.20 (m, 1H)	0
3a	1.37 (dt, J = 13.9, 4.6 Hz, 1H)	1.37 (app. td, J = 14.0, 4.9 Hz, 1H)	0
3b	1.62 (ddd, *J* = 13.9, 4.6, 2.5 Hz, 1H)	1.67 (ddd, *J* = 14.4, 4.5, 2.3 Hz, 1H)	+0.05
4	-	-	-
5	0.84 (br.d, *J* = 5.7 Hz, 1H)	0.85 (d, *J* = 5.6 Hz, 1H)	-0.01
6	1.24 (ddd, *J* = 8.5, 5.7, 3.2 Hz, 1H)	1.24 – 1.22 (m, 1H)	0
7	0.52 (td, *J* = 9.1, 3.2 Hz, 1H)	0.52 (dt, *J* = 8.3, 3.0 Hz, 1H)	0
8	1.14 (dddd, *J* = 11.2, 8.5, 6.7, 3.2 Hz)	1.14 (dddd, *J* = 11.3, 7.6, 4.0 Hz)	0
9a	0.91 (m, 1H, 1H)	0.91 – 0.90 (m, 1H)	0
9b	1.84 (dd, *J* = 12.8, 6.7 Hz, 1H)	1.81 (dd, *J* = 12.6, 6.9 Hz, 1H)	-0.03
10	-	-	-
11	0.94 (m, 1H)	0.93 (m, 1H)	+0.01
12	0.93 (s, 6H)	0.94 (m, 6H)	-0.01
13	“”	“”	0
14	1.29 (s, 3H)	1.29 (s, 3H)	0
15	1.32 (s, 3H)	1.31 (s, 3H)	-0.01
OH	1.16 (br.s, 1H, OH)	1.14 (br.s, 1H, OH)	-0.02

NB: The spectrum was calibrated to signal δ$_H$ = 0.52 ppm

Table S2. Comparison of ¹H-NMR data of natural and synthetic **3**[22]

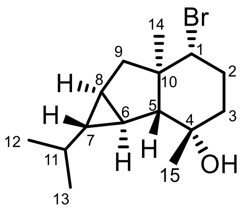

C	**natural**<br>δ_C (75 MHz, $CDCl_3$)	**synthetic**<br>δ_C (151 MHz, $CDCl_3$)	$\Delta\delta_C$
1	61.96	61.93	-0.03
2	31.16	31.17	+0.01
3	41.63	41.66	+0.03
4	71.18	71.18	0
5	62.18	62.22	+0.04
6	25.24	25.24	0
7	49.90	49.93	+0.03
8	23.11	23.11	0
9	45.95	45.98	+0.03
10	58.13	58.15	+0.02
11	32.42	32.41	-0.01
12	21.76 or 21.87	21.75 or 21.86	+0.01
13	“”	“”	-
14	17.76	17.65	-0.1
15	30.31	30.32	+0.01

NB: The spectrum was calibrated to signal δ_C = 71.18 ppm

6,8-cycloeudesmane 9

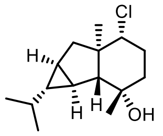

To a solution of NCS (3.2 mg, 0.024 mmol, 1.1 eq) in DCM (0.6 mL) at –78 °C was added germacradien-4-ol (5.0 mg, 0.023 mmol) as a solution in DCM (0.3 mL). The resulting solution was stirred for 1 h at –78 °C, then allowed to warm to rt. The reaction mixture was then extracted with sat. aq. $Na_2S_2O_3$ (10 mL), and the organic phase was collected. The aqueous phase was extracted with DCM (2 × 5 mL), and the combined organic phases were dried over Na_2SO_4 and concentrated *in vacuo*. ^{1}H-NMR analysis of the crude oil showed that **9** was formed in 33% (mesitylene internal standard). The crude mixture was dissolved again in DCM (1 mL) and cooled to 0 °C. $NaHCO_3$ (3.8 mg, 0.045 mmol, 2 eq) and mCPBA (6.1 mg, 77% purity, 0.027 mmol, 1.2 eq) were then added, and the resulting mixture was stirred at 0 °C for 1 h. The mixture was then warmed to rt and sat. aq. $Na_2S_2O_3$ (3 mL) was added. The biphasic mixture was separated, and the aqueous phase was extracted with DCM (2 × 5 mL). The combined organic phases were washed with brine (5 mL), and dried over Na_2SO_4, then concentrated *in vacuo*. The resultant crude oil was purified by flash chromatography (15 – 40% Et_2O in heptane) to give **9** (2.1 mg, 36%) as a clear oil.

$[\alpha]_D^{20}$ = –8 (*c* = 0.1, $CHCl_3$); **IR** (film) ν_{max} 3463, 3012, 2868, 1465, 1199, 1011, 949 cm^{-1}; **^{1}H NMR** (400 MHz, $CDCl_3$) δ 3.68 (dd, *J* = 12.1, 4.1 Hz, 1H), 2.10 (qd, *J* = 13.4, 4.6 Hz, 1H), 1.85 (td, *J* = 11.3, 4.7 Hz, 2H), 1.76 – 1.68 (m, 1H), 1.44 – 1.37 (m, 1H), 1.31 (s, 3H), 1.28 (s, 3H), 1.19 (m, 3H), 0.95 (d, *J* = 4.8 Hz, 6H), 0.93 – 0.88 (m, 2H), 0.80 (d, *J* = 5.3 Hz, 1H), 0.53 (dt, *J* = 6.9, 3.1 Hz, 1H); **^{13}C NMR** (101 MHz, $CDCl_3$) δ 71.2, 68.4, 62.3, 58.3, 50.1, 45.3, 41.0, 32.6, 30.5, 30.3, 24.9, 23.8, 22.0, 21.9, 16.6; **HRMS** (EI+) $[M – HCl]^+$ 220.1818 found, calculated 220.1822 for $C_{15}H_{24}O$.

6,8-cycloeudesmane 12

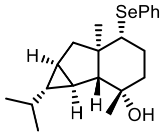

To a solution of *N*-(phenylseleno)phthalimide (61.2 mg, 0.202 mmol, 3 eq) in DCM (2.7 mL) at 0 °C was added germacradien-4-ol (15.0 mg, 0.068 mmol) as a solution in DCM (0.6 mL). The resulting solution was stirred for 1 h at 0 °C, then allowed to warm to rt, with continued stirring for 48 h. The reaction mixture was then concentrated *in vacuo*, ^{1}H-NMR analysis of the crude oil showed that **12** was formed in 15% yield (mesitylene internal standard). The crude mixture was then purified by flash chromatography (15 – 40% Et_2O in heptane) to give **12** (6.4 mg, 1.4:1 ratio between title compound and alkene byproducts, 15%) as a clear oil.

IR (film) ν_{max} 3461, 2955, 2867, 1713, 1197, 1109, 739 cm^{-1}; **^{1}H NMR** (700 MHz, $CDCl_3$) δ 7.55 – 7.51 (m, 2H), 7.24 (d, *J* = 1.7 Hz, 3H), 2.96 (dd, *J* = 12.6, 3.7 Hz, 1H), 2.10 (dd, *J* = 12.8, 4.4 Hz, 1H), 1.98 (s, 1H), 1.88 (dd, *J* = 12.6, 6.9 Hz, 1H), 1.85 – 1.81 (m, 1H), 1.66 (ddd, *J* = 14.2, 4.1, 2.3 Hz, 1H), 1.28 (m, 7H), 1.20 (dd, *J* = 5.6, 2.7 Hz, 1H), 1.17 – 1.13 (m, 1H), 1.03 (d, *J* = 6.3 Hz, 3H), 0.97 – 0.94 (m, 3H), 0.93 – 0.92 (m, 1H), 0.91 – 0.90 (m, 1H), 0.82 (d, *J* = 5.6 Hz, 1H), 0.51 (dt, *J* = 8.4, 2.9 Hz, 1H); **^{13}C NMR** (176 MHz, $CDCl_3$) δ 146.6, 134.7 (2C), 129.0 (2C), 127.3, 71.6, 63.3, 57.6, 55.1, 50.1, 46.4, 41.8, 32.6, 30.7, 29.9, 24.9, 23.7, 22.1, 21.90, 18.9; **HRMS** (EI+) $[M]^+$ 378.1449 found, calculated 378.1462 for $C_{21}H_{30}SeO^+$.

6,8-cycloeudesmane 14

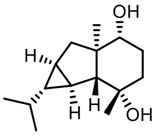

To a solution of germacradien-4-ol **7** (30.0 mg, 0.135 mmol, 1 eq) and $NaHCO_3$ (17.0 mg, 0.202 mmol, 1.5 eq) in DCM (2 mL) at 0 °C was added *m*CPBA (33.3 mg, 0.148 mmol, 1.1 eq). The resulting suspension was stirred at 0 °C for 20 min, then warmed to rt, and filtered through a syringe filter, washing with DCM until the filtrate diluted to 4 mL total volume. At this point, pivalic acid (41.3 mg, 0.405 mmol, 3 eq) was added. The resulting solution was stirred for rt for 48 h, then concentrated *in vacuo*. ^{1}H-NMR analysis of the crude oil showed that **14** was formed in 13% (mesitylene internal standard). [23] An analytical sample was purified by flash column chromatography.

$[\alpha]_D^{20}$ = –9 (c = 0.15, $CHCl_3$) measured, $[\alpha]_D^{20}$ = –4 (c = 0.25, $CHCl_3$) reported.[23]

Table S3. Comparison of ^{1}H-NMR data of natural and synthetic **14**[23]

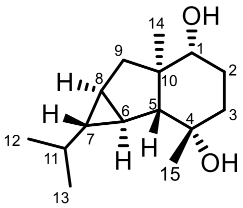

H	natural<br>δ_H (300 MHz, $CDCl_3$)	synthetic<br>δ_H (700 MHz, $CDCl_3$)	$\Delta\delta_H$
1	3.33 (dd, *J* = 10.9, 4.4 Hz, 1H)	3.33 (dd, *J* = 11.3, 4.4 Hz, 1H)	0
2a	1.72 (m, 1H)	1.73 (m, 1H)	+0.01
2b	1.61 (m, 1H)	1.61 (m, 1H)	0
3a	1.66 (m, 1H)	1.69 (m, 1H)	+0.03
3b	1.35 (m, 1H)	1.36 (td, *J* = 14.0, 4.9 Hz, 1H)	+0.01
4	-		-
5	0.71 (d, *J* = 4.6 Hz, 1H)	0.72 (d, *J* = 5.3 Hz, 1H)	+0.01
6	1.17 (m, 1H)	1.16 (m, 1H)	-0.01
7	0.52 (m, 1H)	0.51 (dt, *J* = 8.4, 3.0 Hz, 1H)	-0.01
8	1.18 (m, 1H)	1.19 (m, 1H)	+0.01
9a	0.91 (m, 1H)	0.91 (m, 1H)	0
9b	1.84 (m, 1H)	1.84 (dd, *J* = 12.1, 6.5 Hz, 1H)	0
10	-	-	-
11	0.98 (m, 1H)	0.98 (m, 1H)	0
12	0.93 (d, *J* = 6.6 Hz, 3H)	0.95 (d, *J* = 5.9 Hz, 3H)	+0.02
13	0.93 (d, *J* = 6.6 Hz, 3H)	0.95 (d, *J* = 5.9 Hz, 3H)	+0.02
14	1.17 (s, 3H)	1.18 (s, 3H)	+0.01
15	1.30 (s, 3H)	1.31	-0.01
OH	-	-	-
OH	-	-	-

Table S4. Comparison of ^{1}H-NMR data of natural and synthetic **14**[23]

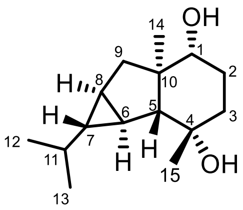

C	**natural** δC (75 MHz, $CDCl_3$)	**synthetic** δC (176 MHz, $CDCl_3$)	**Δδ$_C$**
1	78.04	78.10	+0.06
2	28.20	28.23	+0.03
3	39.57	39.59	+0.02
4	70.98	71.04	+0.06
5	60.61	60.61	0
6	23.97	24.00	+0.03
7	49.89	49.93	+0.04
8	24.37	24.42	+0.05
9	44.48	44.48	0
10	58.03	58.05	+0.02
11	32.48	32.52	+0.04
12	21.87	21.91	+0.04
13	21.73	21.78	+0.05
14	15.51	15.53	+0.02
15	30.28	30.32	+0.04

NB: The spectrum was calibrated to signal δ_C = 60.61 ppm

6,8-cycloeudesmane 19

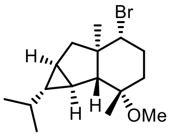

To a solution of NBS (16.6 mg, 1.1 eq, 0.093 mmol) in DCM (3.5 mL) at –78 °C was added germacradien-4-OMe **17** (20.0 mg, 0.085 mmol) as a solution in DCM (1 mL). The resulting solution was stirred for 1 h at –78 °C, then allowed to warm to rt. The reaction mixture was then extracted with sat. aq. $Na_2S_2O_3$ (5 mL), and the organic phase was collected. The aqueous phase was extracted with DCM (2 × 5 mL), and the combined organic phases were dried over Na_2SO_4 and concentrated *in vacuo*. The crude mixture was dissolved again in DCM (1 mL) and cooled to 0 °C. $NaHCO_3$ (14.2 mg, 0.17 mmol, 2 eq) and mCPBA (20.9 mg, 77% purity, 0.093 mmol, 1.2 eq) were then added, and the resulting mixture was stirred at 0 °C for 1 h. The mixture was then warmed to rt and sat. aq. $Na_2S_2O_3$ (10 mL) was added. The biphasic mixture was separated, and the aqueous phase was extracted with DCM (2 × 10 mL). The combined organic phases were washed with brine (20 mL), and dried over Na_2SO_4, then concentrated *in vacuo*. The resultant crude oil was purified by flash chromatography (0 – 5% Et_2O in heptane) to give **19** (9.1 mg, 34%) as a clear oil.

$[\alpha]_D^{20}$ = –20 (*c* = 0.1, $CHCl_3$); **IR** (film) ν_{max} 3012, 2955, 2866, 1715, 1222, 1080, 734 cm^{-1}; **^{1}H NMR** (400 MHz, $CDCl_3$) δ 3.87 (dd, *J* = 12.2, 4.0 Hz, 1H), 3.19 (s, 3H), 2.10 (qd, *J* = 13.1, 4.0 Hz, 1H), 2.03 – 1.96 (m, 1H), 1.94 – 1.86 (m, 1H), 1.80 (dd, *J* = 12.5, 7.0 Hz, 1H), 1.39 – 1.33 (m, 1H), 1.28 (s, 3H), 1.22 (s, 3H), 1.14 – 1.01 (m, 2H), 0.94 (d, *J* = 5.3 Hz, 6H), 0.92 – 0.86 (m, 2H), 0.83 (d, *J* = 5.4 Hz, 1H), 0.50 – 0.42 (m, 1H); **^{13}C NMR** (101 MHz, $CDCl_3$) δ 74.8, 63.7, 62.9, 58.4, 50.1, 49.2, 46.2, 35.5, 32.5, 31.4, 25.6, 23.6, 23.2, 22.1, 21.9, 17.9; **HRMS** (EI+) $[M – MeOH]^+$ 282.0960 found, calculated 282.0978 for $C_{15}H_{24}Br^+$.

6,8-cycloeudesmane 20

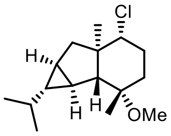

To a solution NCS (4.4 mg, 1.1 eq, 0.033 mmol) in DCM (1.2 mL) at –78 °C was added germacradien-4-OMe **17** (7.0 mg, 0.030 mmol) as a solution of in DCM (0.3 mL). The resulting solution was stirred for 1 h at –78 °C, then allowed to warm to rt. The reaction mixture was then extracted with sat. aq. $Na_2S_2O_3$ (2 mL), and the organic phase was collected. The aqueous phase was extracted with DCM (2 × 5 mL), and the combined organic phases were dried over Na_2SO_4 and concentrated *in vacuo*. The crude mixture was dissolved again in DCM (1 mL) and cooled to 0 °C. $NaHCO_3$ (5.0 mg, 0.059 mmol, 2 eq) and mCPBA (7.3 mg, 77% purity, 0.033 mmol, 1.2 eq) were then added, and the resulting mixture was stirred at 0 °C for 1 h. The mixture was then warmed to rt and sat. aq. $Na_2S_2O_3$ (5 mL) was added. The biphasic mixture was separated, and the aqueous phase was extracted with DCM (2 × 5 mL). The combined organic phases were washed with brine (5 mL), and dried over Na_2SO_4, then concentrated *in vacuo*. The resultant crude oil was purified by flash chromatography (0 – 5% Et_2O in heptane) to give **20** (2.6 mg, 32%) as a clear oil.

[α]$_D^{20}$ = +14 (*c* = 0.05, $CHCl_3$); **IR** (film) ν_{max} 3011, 2954, 2855, 1712, 1196, 1080, 911 cm^{-1}; **^{1}H NMR** (600 MHz, $CDCl_3$) δ 3.67 (dd, *J* = 12.1, 4.1 Hz, 1H), 3.19 (s, 3H), 2.04 (dt, *J* = 14.8, 3.5 Hz, 1H), 1.94 (ddd, *J* = 25.8, 13.5, 4.2 Hz, 1H), 1.84 (dd, *J* = 12.5, 7.0 Hz, 1H), 1.79 – 1.73 (m, 1H), 1.33 (ddd, *J* = 8.3, 5.6, 2.9 Hz, 1H), 1.24 – 1.22 (m, 6H), 1.14 (app.hept, *J* = 11.3, 7.5, 4.0 Hz, 1H), 1.08 – 1.02 (m, 1H), 0.96 – 0.92 (m, 6H), 0.87 (s, 2H), 0.77 (d, *J* = 5.5 Hz, 1H), 0.45 (dt, *J* = 8.4, 3.0 Hz, 1H); **^{13}C NMR** (151 MHz, $CDCl_3$) δ 74.6, 69.0 63.7, 58.3, 50.1, 49.2, 45.4, 34.6, 32.6, 30.4, 25.1, 23.7, 23.6, 22.1, 21.9, 16.7; **HRMS** (EI+) $[M – MeOH]^+$ 255.1504 found, calculated 255.1510 for $C_{15}H_{24}ClO^+$.

Aldehyde S1

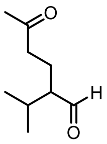

Aldehyde **S1** was prepared according to the protocol described by Baran et al.[24]

Sulfone S2

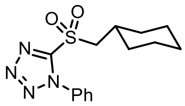

Sulfone **S2** was prepared according to the protocol described by Fuwa et al.[25]

Alkene S3

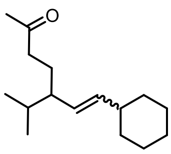

To a solution of sulfone **S2** (902 mg, 2.94 mmol, 1.15 eq) in THF (10 mL) at –78 °C was added LiHMDS (2.94 mL, 1 M solution in THF, 2.94 mmol, 1.15 eq) dropwise. After 25 min, aldehyde **S1** (400 mg, 2.56 mmol, 1.0 eq) as a solution in THF (2 mL), was added dropwise. The reaction mixture was stirred at –78 °C for 1.5 h and was allowed to warm to rt and stir for a further 16 h. Sat. aq. ammonium chloride (30 mL) was added, and the mixture was extracted with diethyl ether (3 × 50 mL). The combined organic phases were washed with brine, dried over $NaSO_4$ and concentrated *in vacuo*. The crude oil was purified by flash chromatography (5-30% EtOAc in heptane) to give alkene **S3** as a clear oil (343 mg, 57%, *E:Z* = 1.0.8).

^{1}H NMR (400 MHz, $CDCl_3$) δ 5.34 – 5.19 (m, 1.8H), 5.03 (dd, *J* = 15.4, 9.3 Hz, 0.8H), 4.91 (app. t, *J* = 10.8 Hz, 1H), 2.36 (ddd, *J* = 21.5, 9.6, 5.9 Hz, 3.6H), 2.11 (s, 7.3H), 1.96 – 1.88 (m, 0.8H), 1.81 – 1.47 (m, 14.3H), 1.16 (dd, *J* = 80.2, 12.0 Hz, 10H), 0.91 – 0.79 (m, 11H); **^{13}C NMR** (101 MHz, $CDCl_3$) δ 209.8, 209.6, 138.7, 137.7, 129.5, 128.9, 49.2, 43.3, 42.30, 42.28, 41.0, 36.8, 33.7, 33.58, 33.55, 33.3, 32.4, 32.3, 30.2, 30.1, 26.7, 26.5, 26.4, 26.21, 26.18, 26.1, 26.0, 20.83, 20.81, 19.4, 19.2; **HRMS** (EI+) $[M]^+$ 236.2132 found, calculated 236.2140 for $C_{16}H_{28}O^+$

Reported as a mixture of *E* and *Z* isomers.

Diene 21

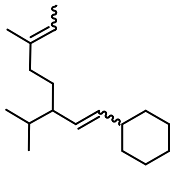

To ethyltriphenylphosphonium bromide (60 mg, 0.162 mmol, 1.6 eq) in THF at –78 °C was added *n*-BuLi (105 μL, 1.6 M in hexane, 1.6 eq, 0.168 mmol) dropwise. After 1 h, ketone **S3** (24 mg, 0.102 mmol, 1.0 eq) was added as a solution in THF (0.2 mL). After 2 h stirring at –78 °C, the reaction mixture was allowed to warm to rt and stir for 30 min, then it was heated to 50 °C for a further 48 h. Upon cooling to rt, the reaction was diluted in pentane (50 mL), washed with sat. aq. ammonium chloride (15 mL), and brine (5 mL), then concentrated *in vacuo*. The crude oil was purified by flash chromatography (pentane) to give diene **21** as a clear oil (16 mg, 63%, mixture of stereoisomers).

IR (film) ν_{max} 2955, 2923, 2851, 1448, 1358, 971, 890, 745 cm^{-1}; **^{1}H NMR** (400 MHz, $CDCl_3$) δ 5.35 – 4.92 (m, 3H), 2.27 – 1.75 (m, 4H), 1.74 – 1.42 (m, 14H), 1.13 (dd, *J* = 44.1, 13.1 Hz, 4H), 0.91 – 0.77 (m, 7H); **^{13}C NMR** (101 MHz, $CDCl_3$) δ 138.0, 137.9, 137.0, 136.9, 136.6, 130.4, 130.3, 129.5, 118.70, 49.3, 49.0, 43.5, 41.1, 37.9, 33.6, 33.5, 32.1, 31.0, 29.7, 26.4, 26.3, 26.2, 23.6, 20.9, 19.0, 15.9, 13.5; **HRMS** (EI+) $[M]^+$ 248.2487 found, calculated 248.2504 for $C_{18}H_{32}^+$

Alcohol 24

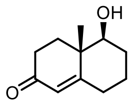

Prepared from enantiopure (*S*)-(+)-Wieland-Miescher according to the literature protocol described by Peng and co-workers.[26]

$[\alpha]_D^{20}$ = +113.6 (c = 1.0, $CHCl_3$).

Diol 25

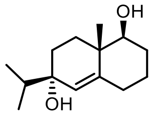

To a mixture of ketone **24** (1.00 g, 5.55 mmol, 1.0 eq) and anhydrous cerium chloride[27] (4.79 g, 19.4 mmol, 3.5 eq) in THF (35 mL) at –30 °C was added isopropyl magnesium chloride (9.47 mL, 2 M in THF, 19.4 mmol, 3.5 eq) dropwise. The reaction was left to stir overnight with slow warming to rt; then aq. NH_4Cl (50 mL) and DCM (50 mL) were added. The biphasic mixture was separated, and the aqueous phase was extracted with DCM (2 × 50 mL). The combined organic phases were washed with brine (50 mL), dried over Na_2SO_4 and concentrated *in vacuo*. The resultant crude oil was purified by flash column chromatography (10-100% EtOAc in heptane) to afford diol **25** as a clear gum (666 mg, 54% yield).

IR (film) ν_{max} 3358, 2940, 2866, 1466, 1415, 1142, 974, 625 cm^{-1}; **^{1}H NMR** (400 MHz, $CDCl_3$) *δ* 5.35 (s, 1H), 3.32 (d, *J* = 11.2 Hz, 1H), 2.16 (app. t, *J* = 13.2 Hz, 1H), 1.89 (d, *J* = 13.1 Hz, 1H), 1.75 (dd, *J* = 20.5, 11.1 Hz, 5H), 1.67 – 1.55 (m, 2H), 1.45 (d, *J* = 10.0 Hz, 1H), 1.20 (d, *J* = 13.4 Hz, 1H), 1.03 (s, 3H), 0.93 (d, *J* = 6.4 Hz, 3H), 0.88 (d, *J* = 6.6 Hz, 3H); **^{13}C NMR** (101 MHz, $CDCl_3$) *δ* 145.7, 127.5, 75.9, 72.5, 40.9, 37.0, 31.7, 30.8, 30.6, 28.2, 25.8, 18.2, 17.6, 16.8; **HRMS** (ESI+) $[M + Na]^+$ 247.1667 found, calculated 247.1669 for $C_{14}H_{24}O_2Na^+$; **$[\alpha]_D^{20}$** = +19 (c = 1.0, $CHCl_3$).

Alcohol S4

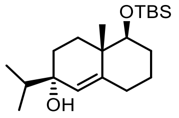

To a solution of alcohol **25** (310 mg, 1.38 mmol, 1.0 eq) and DIPEA (0.72 mL, 4.15 mmol, 3.0 eq) in DCM (5 mL) at 0 °C was added TBSOTf (0.39 mL, 1.66 mmol, 1.2 eq) as a solution in DCM (1 mL) over 10 min. The reaction was left to stir for 16 h at rt, then sat. aq. ammonium chloride (20 mL) was added. The biphasic mixture was separated, and the aqueous phase was extracted with DCM (2 x 30 mL). The combined organic phases were washed with brine (30 mL), dried over Na_2SO_4 and concentrated *in vacuo*. The resultant crude oil was purified by flash column chromatography (15% EtOAc in heptane) to afford alcohol **S4** as a clear oil (368 mg, 78% yield).

IR (film) ν_{max} 1470, 1250, 1083, 1029, 984, 831, 771, 668 cm^{-1}; **^{1}H NMR** (400 MHz, $CDCl_3$) δ 5.34 (s, 1H), 3.29 (dd, *J* = 10.5, 5.3 Hz, 1H), 2.25 – 2.11 (m, 1H), 1.88 (d, *J* = 13.2 Hz, 1H), 1.80 – 1.70 (m, 3H), 1.61 (ddd, *J* = 13.1, 9.1, 3.5 Hz, 4H), 1.47 – 1.38 (m, 1H), 1.02 (s, 3H), 0.96 (d, *J* = 6.8 Hz, 3H), 0.92 (d, *J* = 6.9 Hz, 3H), 0.89 (s, 11H), 0.03 (s, 6H); **^{13}C NMR** (101 MHz, $CDCl_3$) δ 146.3, 127.1, 76.4, 72.7, 41.5, 37.0, 31.9, 31.5, 31.1, 28.4, 26.0, 25.7, 18.7, 18.2, 17.6, 16.9, -3.8, -4.8; **HRMS** (ESI+) $[M + Na]^+$ 361.2530 found, calculated 361.2533 for $C_{20}H_{38}O_2NaSi^+$; **$[\alpha]_D^{20}$** = +26 (c = 0.1, $CHCl_3$).

Alkene 26

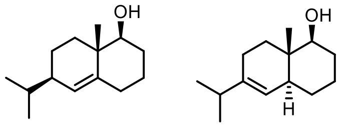

2 : 1

To a solution of alcohol **25** (365 mg, 1.08 mmol, 1.0 eq) in DCM (12 mL) at –78 °C was added triethylsilane (1.03 mL, 6.47 mmol, 6 eq) and BF_3•OEt_2 (0.16 mL, 1.29 mmol, 1.2 eq). The resultant mixture was stirred for 20 min at this temperature, then Et_3N (0.526 mL, 3.77 mmol, 3.5 eq) was added and the mixture was allowed to warm to rt. The reaction mixture was diluted in DCM (50 mL), washed with sat. aq. NH_4Cl (20 mL), then brine (10 mL), then the organic phase was dried over Na_2SO_4 and concentrated *in vacuo*. The crude oil was dissolved in THF (10 mL), then TBAF was added (4.31 mL, 1 M in THF, 4.31 mmol, 4.0 eq), and the resulting solution was heated at 50 °C for 40 h with stirring. The reaction mixture was then cooled to rt, diluted in Et_2O (50 mL), washed with H_2O (30 mL), then brine (20 mL). The organic phase was subsequently dried over Na_2SO_4, concentrated *in vacuo*, and purified by flash chromatography (15% EtOAc in heptane) to give alkene **26** as a 2:1 mixture of alkene regioisomers (183 mg, 82% yield).

IR (film) ν_{max} 3387, 2940, 2866, 1466, 1415, 1142, 974, 625 cm^{-1}; **^{1}H NMR** (400 MHz, $CDCl_3$) δ 5.29 (s, 1H)†, 5.04* (s, 0.5H), 3.28 (m, 1.5H), 2.14 (m, 1H)†, 1.92 (m, 3.5H), 1.78 – 1.72 (m, 3H)†, 1.63 – 1.55 (m, 2.5H), 1.38 – 1.28 (m, 4.5H), 1.02 – 0.98 (m, 7H), 0.92 – 0.84 (m, 8H), 0.74* (s, 1.5H); **^{13}C NMR** (176 MHz, $CDCl_3$) δ 142.3*, 142.2†, 125.9†, 121.7*, 80.3†, 78.5*, 43.0*, 42.7†, 40.7*, 36.4†, 34.8*, 33.7*, 32.3†, 32.0†, 30.9†, 30.6†, 29.6*, 27.0*, 25.0†, 24.7*, 23.4*, 21.9*, 21.7†, 21.5*, 19.8†, 19.3†, 17.2†, 9.5*; **HRMS** (EI+) $[M]^+$ 208.1817 found, calculated 208.1827 for $C_{14}H_{24}O^+$

Signals arising exclusively from the minor isomer are indicated by an * and those arising solely from the major isomer are indicated by a †; undesignated signals arise from a mixture of both.

Diene 23

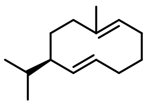

To alcohol **26** (1.05 g, 5.04 mmol, 1.0 eq) in pyridine (15 mL) at 0 °C was added MsCl (1.17 mL, 15.1 mmol, 3.0 eq) dropwise. The resulting solution was warmed to rt and stirred for 30 min. The mixture was then diluted in EtOAc (100 mL), washed with 1 N aq. HCl (2 × 10 mL), then brine (10 mL). The organic phase was then dried over Na_2SO_4 and concentrated *in vacuo*. Traces of pyridine were removed from the crude oil by concentration from toluene (3 × 5 mL), to give the crude mesylate used without further purification.

The crude mesylate was dissolved in THF (20 mL), cooled to 0 °C, then BH_3•THF (30.2 mL, 30.2 mmol, 6 eq) was added dropwise. After 5 min, the solution was then warmed to rt and stirred for 2 h. MeOH (1.2 mL) then NaOMe (4 mL, 2 M in MeOH, freshly prepared) were then added dropwise, and once the evolution of gas had subsided, the solution was brought to reflux for 30 min. The mixture was allowed to cool to rt, then sat. aq. NH_4Cl (50 mL) was then added carefully. The biphasic mixture was extracted with pentane (3 × 150 mL), then the organic phases were dried over Na_2SO_4 and concentrated *in vacuo*. The resultant crude oil was purified by flash chromatography (pentane) to afford cyclodecadiene **23** (486 mg, 50% yield) as a clear oil.

$[\alpha]_D^{20}$ = +58 (*c* = 0.2, $CHCl_3$); **IR** (film) ν_{max} 2954, 2921, 2851, 1464, 1367, 1038, 992 cm^{-1}; **^{1}H NMR** (400 MHz, $CDCl_3$) δ 5.25 – 5.14 (m, 1H), 5.11 – 4.98 (m, 2H), 2.36 – 2.22 (m, 4H), 2.13 – 2.04 (m, 1H), 2.04 – 1.92 (m, 2H), 1.63 (br.d, *J* = 14.0 Hz, 1H), 1.54 (s, 3H), 1.48 – 1.29 (m, 4H), 0.82 (d, *J* = 6.7 Hz, 3H), 0.77 (d, *J* = 6.8 Hz, 3H); **^{13}C NMR** (101 MHz, $CDCl_3$) δ 134.9, 132.0, 130.5, 130.5, 53.3, 41.3, 34.4, 33.2, 29.3, 27.0, 25.5, 20.8, 19.1, 17.0; **HRMS** (EI+) $[M]^+$ 192.1876 found, calculated 192.1878 for $C_{14}H_{24}^+$.

Bromide 28

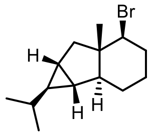

To a solution cyclodecadiene **23** (32.0 mg, 95% purity, 0.158 mmol) in DCM (22 mL) at –78 °C was added as a solution of NBS (30.9 mg, 1.1 eq, 0.174 mmol) in DCM (2 mL). The resulting solution was stirred for 1 h at –78 °C, then allowed to warm to rt. The reaction mixture was then extracted with sat. aq. $Na_2S_2O_3$ (5 mL), and the organic phase was collected. The aqueous phase was extracted with DCM (2 × 5 mL), and the combined organic phases were dried over Na_2SO_4 and concentrated *in vacuo*. ^{1}H-NMR analysis of the crude oil showed that **28** was formed in 36% (mesitylene internal standard). The crude mixture was dissolved again in DCM (2 mL) and cooled to 0 °C. $NaHCO_3$ (39.8 mg, 0.474 mmol, 3 eq) and mCPBA (39.0 mg, 77% purity, 0.174 mmol, 1.1 eq) were then added, and the resulting mixture was stirred at 0 °C for 1 h. The mixture was then warmed to rt and sat. aq. $Na_2S_2O_3$ (10 mL) was added. The biphasic mixture was separated, and the aqueous phase was extracted with DCM (2 × 10 mL). The combined organic phases were washed with brine (5 mL), and dried over Na_2SO_4, then concentrated *in vacuo*. The resultant crude oil was purified by flash chromatography (pentane) to give **28** (10.8 mg, 25% yield) as a clear oil.

IR (film) ν_{max} 1464, 1446, 1377, 1222, 1054, 924, 702 cm^{-1}; **^{1}H NMR** (600 MHz, $CDCl_3$) δ 3.92 (dd, *J* = 12.0, 4.8 Hz, 1H), 1.99 – 1.92 (m, 1H), 1.92 – 1.86 (m, 2H), 1.77 – 1.70 (m, 2H), 1.38 (qd, *J* = 12.9, 4.0 Hz, 1H), 1.30 – 1.23 (m, 1H), 1.09 (m, 4H), 1.01 – 0.81 (m, 10H), 0.52 (dt, *J* = 8.5, 3.0 Hz, 1H); **^{13}C NMR** (126 MHz, $CDCl_3$) δ 63.0, 58.2, 55.4, 49.5, 45.0, 33.2, 32.4, 29.5, 28.2, 24.1, 23.6, 22.1, 21.9, 15.6; **$[\alpha]_D^{20}$** = –10 (c = 0.1, $CHCl_3$); **HRMS** (EI+) $[M]^+$ 270.0970 found, calculated 270.0983 for $C_{14}H_{23}Br^+$

Alcohol 29

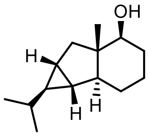

To a solution cyclodecadiene **23** (32.0 mg, 95% purity, 0.158 mmol) in DCM (2 mL) at 0 °C was added $NaHCO_3$ (39.8 mg, 0.474 mmol, 3 eq) and mCPBA (39.0 mg, 77% purity, 0.174 mmol, 1.1 eq) were then added, and the resulting mixture was stirred for 30 min. The mixture was then warmed to rt and filtered through a syringe filter. The filtrate was diluted to by addition of DCM (4.5 mL), then pivalic acid (48.4 mg, 0.474 mmol, 3 eq) was added. ^{1}H-NMR analysis of the crude oil showed that **29** was formed in 13% (mesitylene internal standard). The crude oil was then purified by flash chromatography (10 – 25% acetone in heptane) to give **29** (2.5 mg, 6%) as a clear oil.

$[\alpha]_D^{20}$ = +33 (*c* = 0.1, $CHCl_3$); **IR** (film) ν_{max} 2926, 2855, 2363, 2342, 1712, 912 cm^{-1}; **^{1}H NMR** (400 MHz, $CDCl_3$) δ 3.32 (dd, *J* = 10.8, 4.6 Hz, 1H), 1.90 (dd, *J* = 12.3, 6.8 Hz, 1H), 1.76 – 1.70 (m, 1H), 1.67 – 1.58 (m, 2H), 1.28 – 1.21 (m, 4H), 1.15 – 1.08 (m, 1H), 0.94 – 0.91 (m, 9H), 0.90 – 0.86 (m, 2H), 0.77 – 0.70 (m, 1H), 0.49 (dd, *J* = 8.0, 3.2 Hz, 1H); **^{13}C NMR** (151 MHz, $CDCl_3$) δ 78.0, 58.0, 53.6, 49.5 43.1, 32.5, 30.0, 28.1, 25.5, 25.0, 23.7, 22.1, 22.0, 13.6; **HRMS** (EI+) $[M]^+$ 208.1820 found, calculated 208.1827 for $C_{14}H_{24}O^+$.

Chloride 27

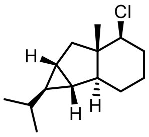

To a solution cyclodecadiene **23** (32.0 mg, 95% purity, 0.158 mmol) in DCM (5.2 mL) at –78 °C was added as a solution of NCS (23.2 mg, 1.1 eq, 0.174 mmol) in DCM (1 mL). The resulting solution was stirred for 1 h at –78 °C, then allowed to warm to rt. The reaction mixture was then extracted with sat. aq. $Na_2S_2O_3$ (5 mL), and the organic phase was collected. The aqueous phase was extracted with DCM (2 × 5 mL), and the combined organic phases were dried over Na_2SO_4 and concentrated *in vacuo*. ^{1}H-NMR analysis of the crude oil showed that **27** was formed in 36% (mesitylene internal standard).The crude mixture was dissolved again in DCM (2 mL) and cooled to 0 °C. $NaHCO_3$ (39.8 mg, 0.474 mmol, 3 eq) and mCPBA (39.0 mg, 77% purity, 0.174 mmol, 1.1 eq) were then added, and the resulting mixture was stirred at 0 °C for 1 h. The mixture was then warmed to rt and sat. aq. $Na_2S_2O_3$ (10 mL) was added. The biphasic mixture was separated, and the aqueous phase was extracted with DCM (2 × 10 mL). The combined organic phases were washed with brine (5 mL), and dried over Na_2SO_4, then concentrated *in vacuo*. The resultant crude oil was purified by flash chromatography (pentane) to give **27** (11.1 mg, 31%) as a clear oil.

$[\alpha]_D^{20}$ = –35 (*c* = 0.1, $CHCl_3$); **IR** (film) ν_{max} 2959, 1364, 2251, 2176, 1713, 1467, 993, 911, 736 cm^{-1}; **^{1}H NMR** (600 MHz, $CDCl_3$) δ 3.71 (dd, *J* = 11.8, 4.6 Hz, 1H), 1.93 (dd, *J* = 12.7, 6.9 Hz, 1H), 1.87 – 1.81 (m, 1H), 1.80 – 1.74 (m, 1H), 1.73 – 1.66 (m, 2H), 1.55 (s, 1H), 1.34 (ddd, *J* = 25.4, 12.7, 3.6 Hz, 1H), 1.29 – 1.22 (m, 1H), 1.09 (dq, *J* = 10.9, 3.7 Hz, 1H), 1.03 (s, 3H), 0.97 – 0.94 (m, 1H), 0.94 – 0.91 (m, 6H), 0.86 – 0.81 (m, 2H), 0.51 (dt, *J* = 8.4, 3.0 Hz, 1H); **^{13}C NMR** (151 MHz, $CDCl_3$) δ 68.7, 58.1, 55.3, 49.5, 44.1, 32.4, 32.2, 28.9, 27.1, 24.1, 23.8, 22.1, 21.9, 14.4; **$[\alpha]_D^{20}$** = – (c = 0.1, $CHCl_3$); **HRMS** (EI+) $[M]^+$ 226.1484 found, calculated 226.1488 for $C_{14}H_{23}Cl^+$

Selenyl ether 30

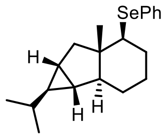

To a solution cyclodecadiene **23** (25.5 mg, 95% purity, 0.126 mmol) in DCM (5.2 mL) at 0 °C was added *N*-(phenylseleno)phthalimide (114 mg, 3 eq, 0.378 mmol). The resulting solution was stirred for 48 h at rt, then concentrated *in vacuo*. ^{1}H-NMR analysis of the crude oil showed that **30** was formed in 14% (mesitylene internal standard). The crude oil could not be satisfactorily purified by flash chromatography; characterisation was obtained from a semi-purified mixture after flash chromatography (50% toluene in heptane). Signals were assigned to the title compound with the aid of HSQC and HMBC experiments.

^{1}H NMR (600 MHz, $CDCl_3$) δ 7.58 – 7.51 (m, 2H), 7.25 – 7.21 (m, 3H), 3.05 – 3.27 (m, 1H), 2.25 – 2.18 (m, 1H), 2.04 – 1.98 (m, 2H), 1.93 (dd, *J* = 12.6, 6.8 Hz, 1H), 1.78 – 1.70 (m, 2H), 1.40 – 1.32 (m, 1H) 1.08 – 1.05 (m, 1H), 1.04 (s, 3H), 0.93 – 0.82 (m, 8H), 0.86 – 0.81 (m, 1H), 0.51 (dt, *J* = 8.4, 3.0 Hz, 1H); **^{13}C NMR** (151 MHz, $CDCl_3$) δ 134.7 (2C), 130.8, 129.0 (2C), 127.1, 60.2, 56.3, 55.3, 49.5, 45.2, 32.5, 32.3, 29.1, 28.2, 24.5, 24.1, 22.1, 22.0, 16.6; **HRMS** (EI+) $[M]^+$ (EI+) $[M]^+$ 348.1346 found, calculated 348.1356 for $C_{20}H_{28}Se^+$

6. NMR spectra

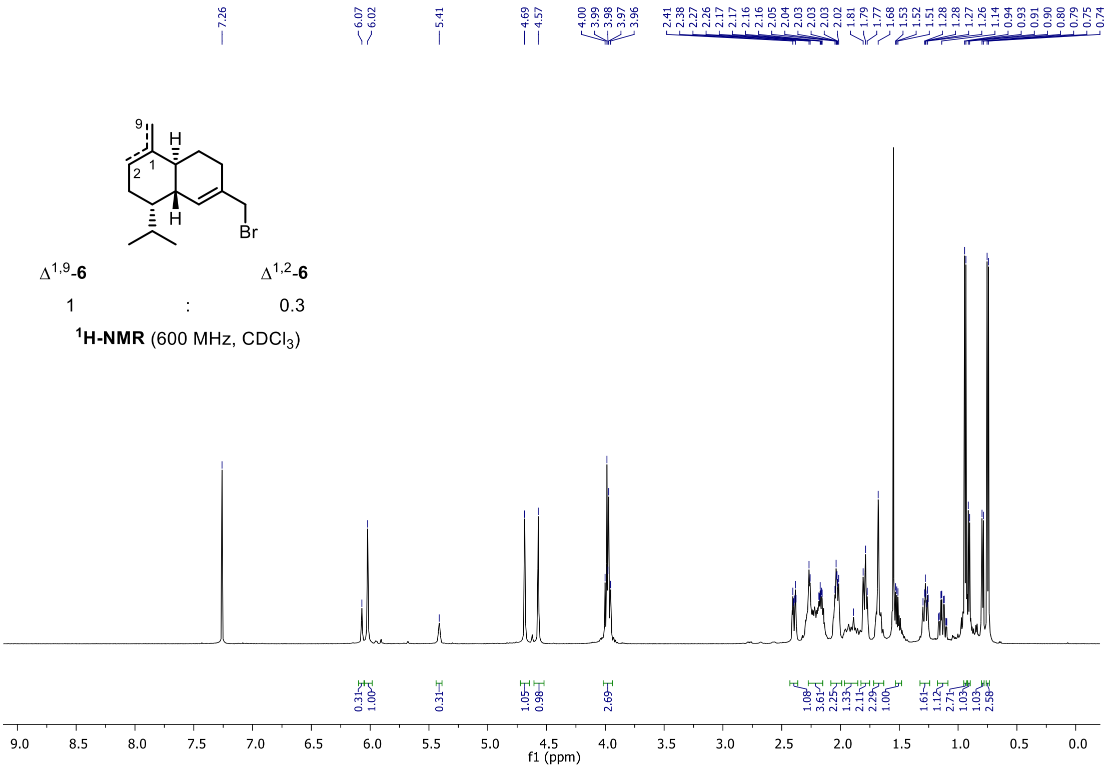

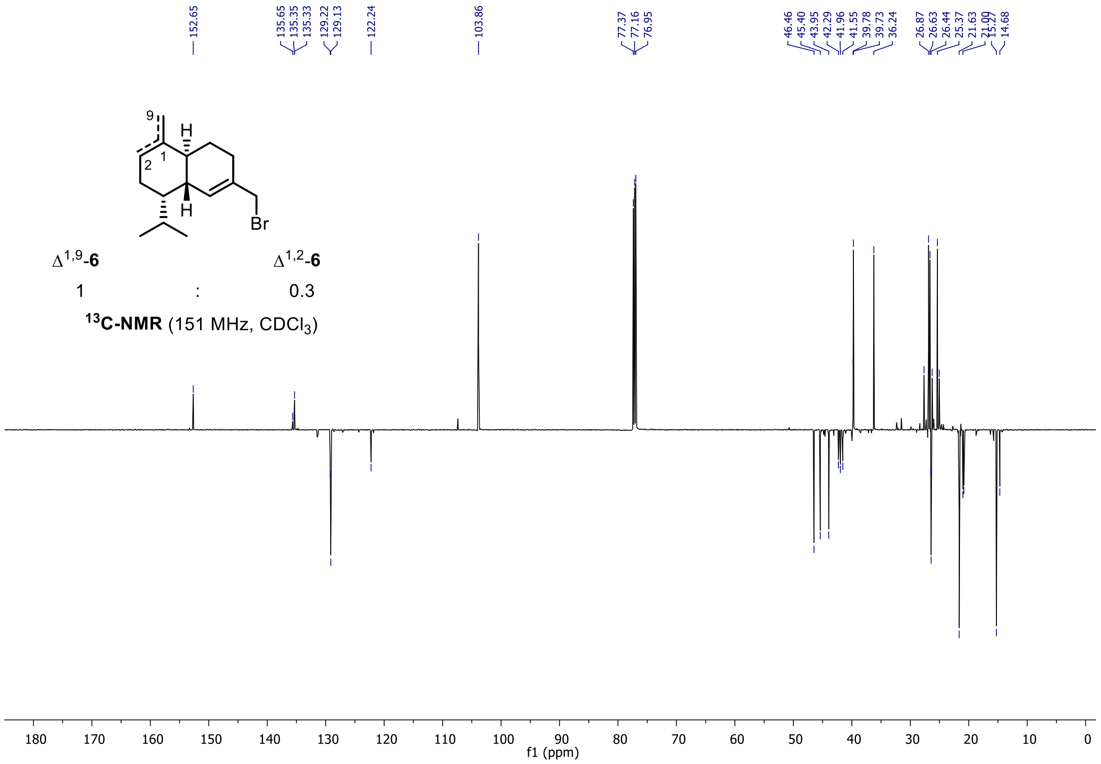

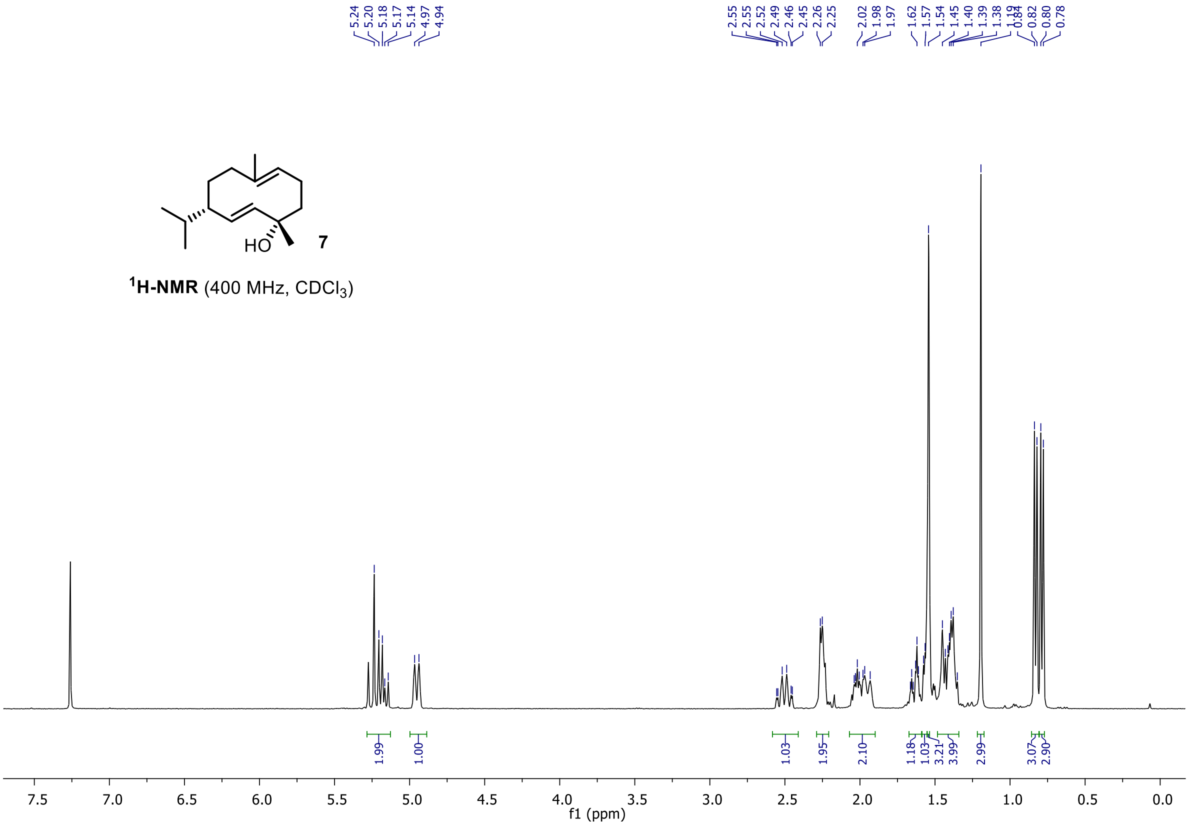

7

^{1}H-NMR (400 MHz, $CDCl_3$)

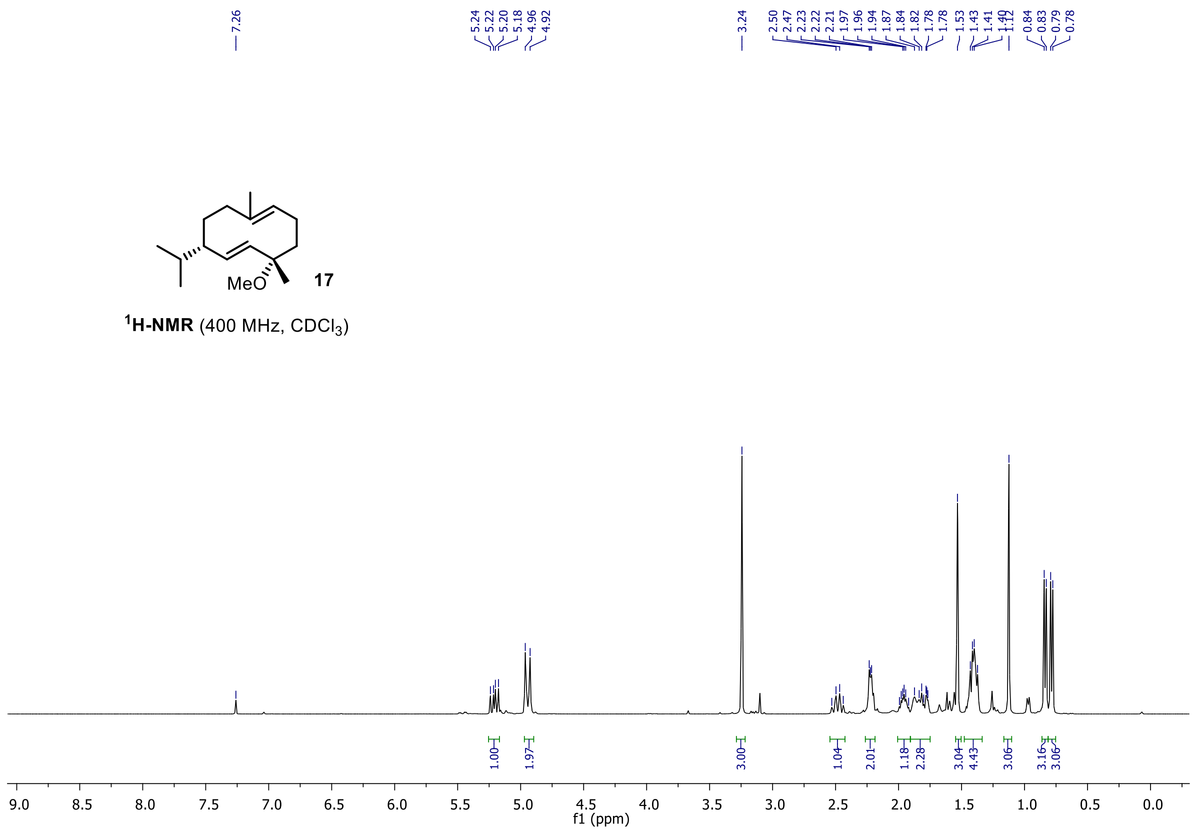

^{1}H-NMR (400 MHz, $CDCl_3$)

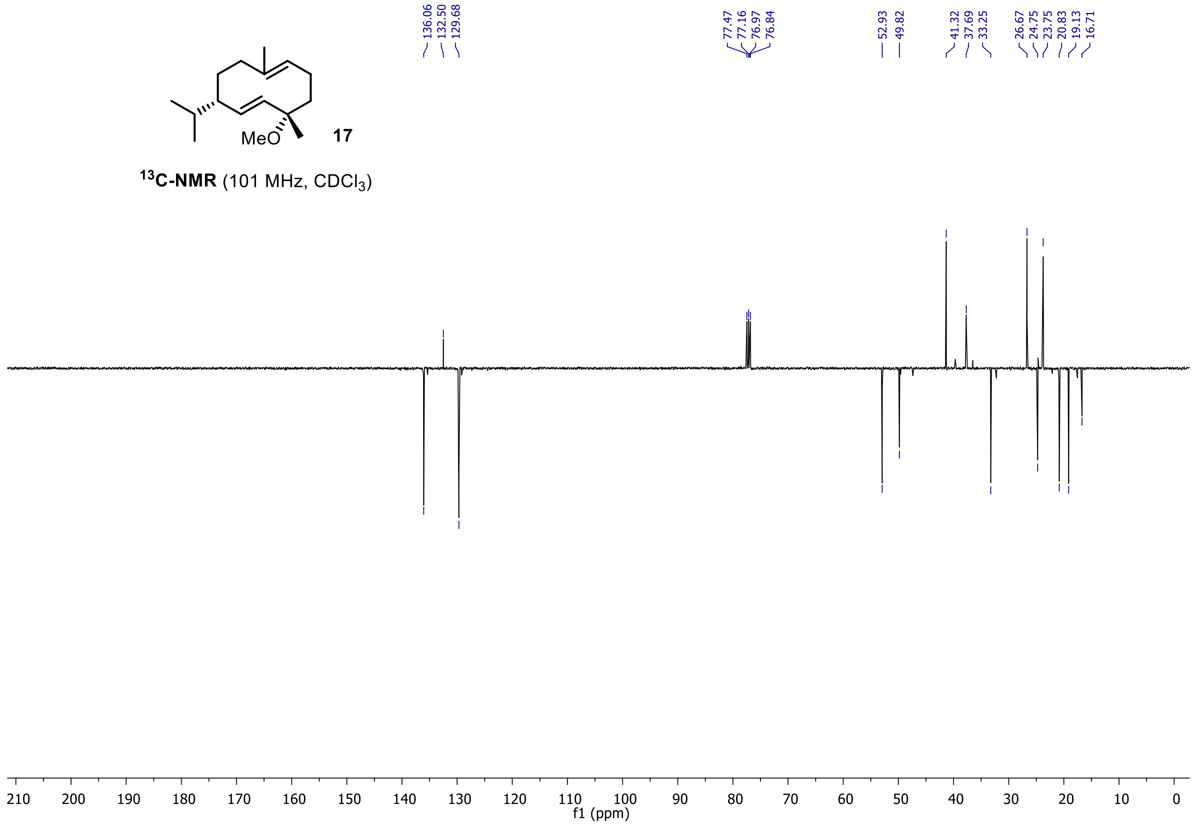

^{13}C-NMR (101 MHz, $CDCl_3$)

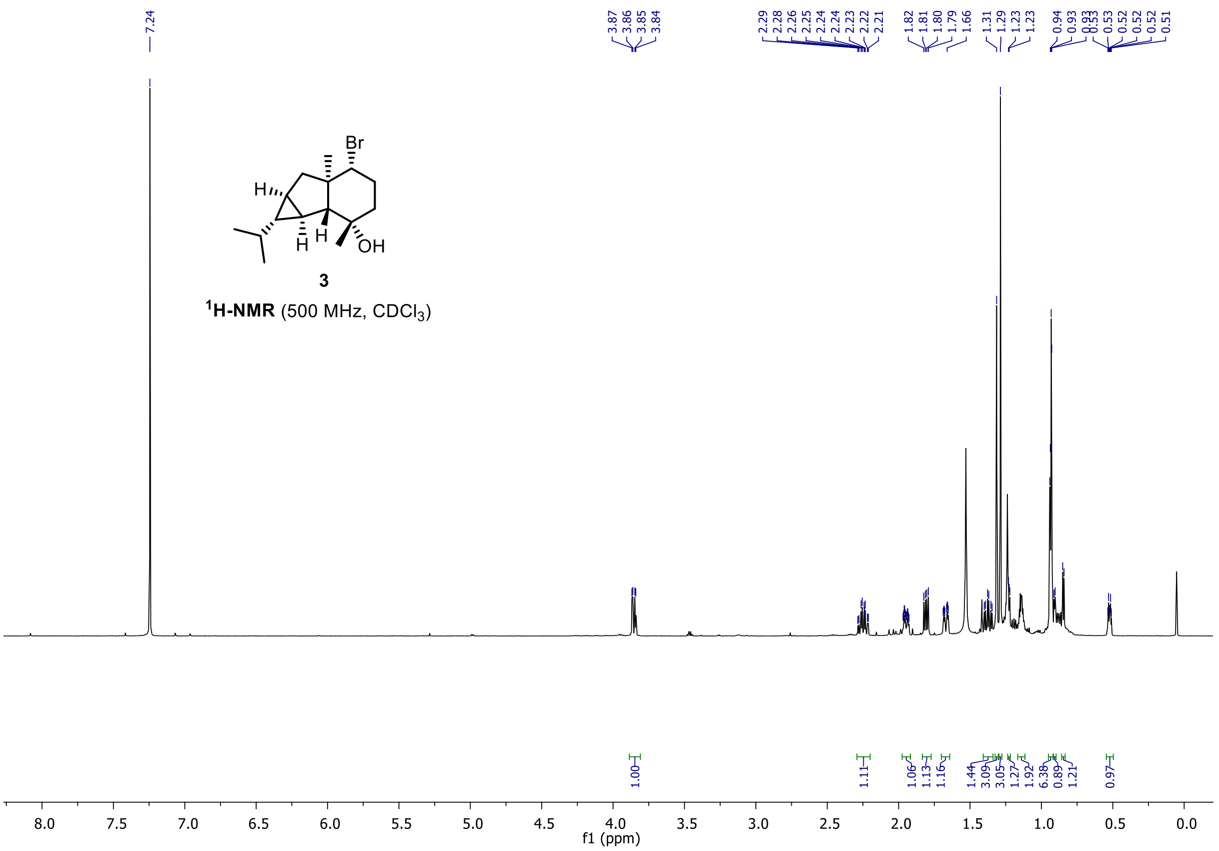
7.24
3.87 3.86 3.85 3.84
2.29 2.28 2.26 2.25 2.24 2.24 2.23 2.22 2.21
1.82 1.81 1.80 1.79 1.66
1.31 1.29 1.23 1.23
0.94 0.93 0.93 0.53 0.53 0.52 0.52 0.52 0.51
Br
H
H
H
OH
3
1H-NMR (500 MHz, CDCl3)
1.00 1.11 1.06 1.13 1.16 1.44 3.09 3.05 1.27 1.92 6.38 0.89 1.21 0.97
8.0 7.5 7.0 6.5 6.0 5.5 5.0 4.5 4.0 3.5 3.0 2.5 2.0 1.5 1.0 0.5 0.0
f1 (ppm)

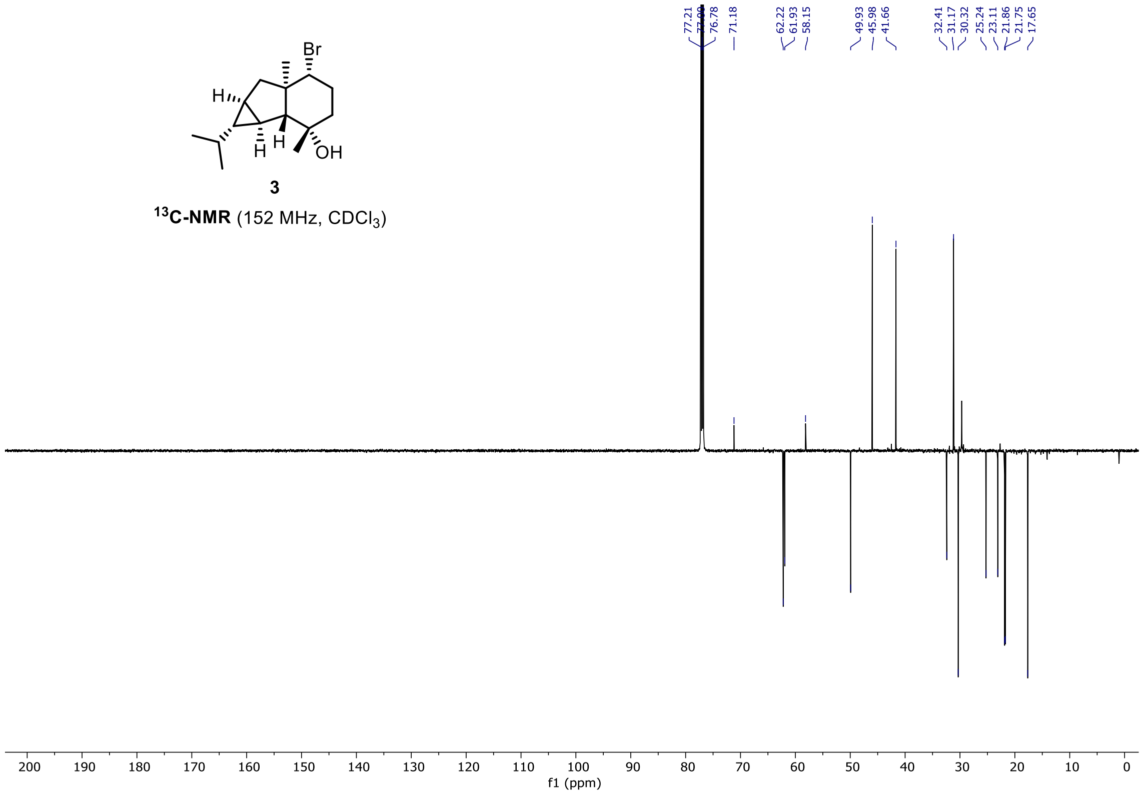
77.21 77.00 76.78 71.18
62.22 61.93 58.15
49.93 45.98 41.66
32.41 31.17 30.32 25.24 23.11 21.86 21.75 17.65
Br
H
H
H
OH
3
13C-NMR (152 MHz, CDCl3)
200 190 180 170 160 150 140 130 120 110 100 90 80 70 60 50 40 30 20 10 0
f1 (ppm)

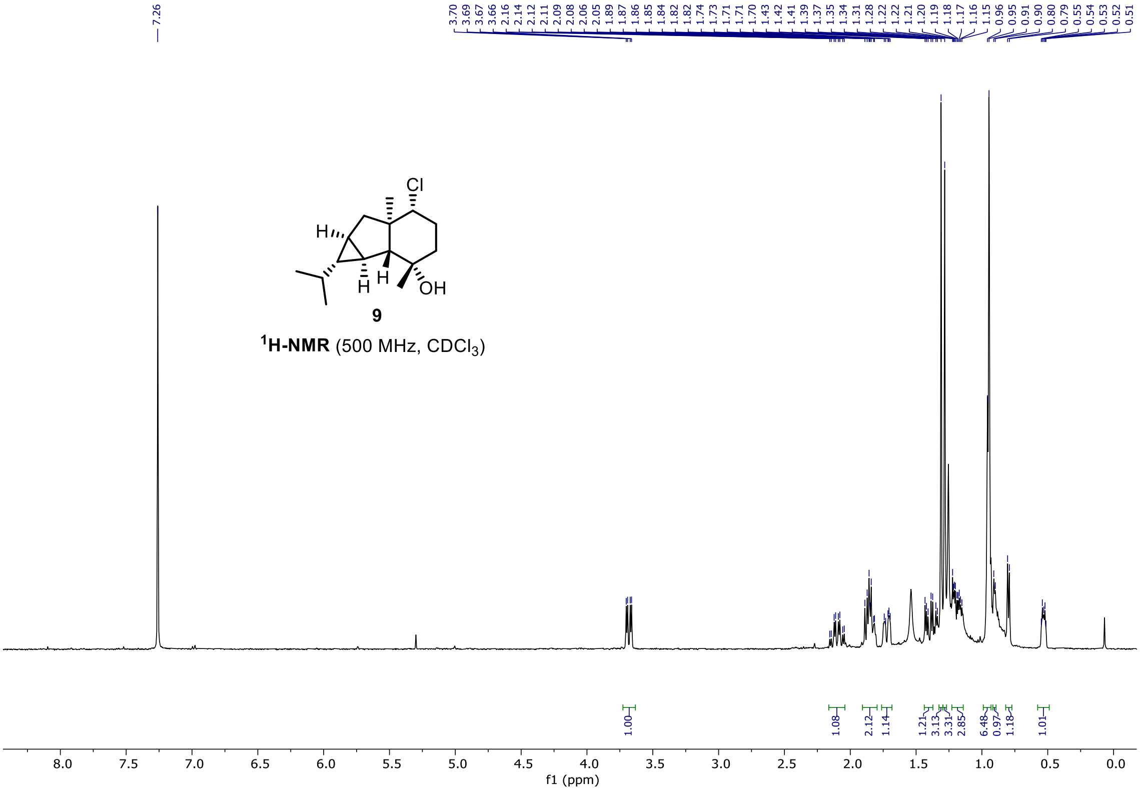
7.26
3.70 3.69 3.67 3.66 2.16 2.14 2.12 2.11 2.09 2.08 2.06 2.05 1.89 1.87 1.86 1.85 1.84 1.82 1.82 1.74 1.73 1.71 1.71 1.70 1.43 1.42 1.41 1.39 1.37 1.35 1.34 1.31 1.28 1.22 1.21 1.20 1.19 1.18 1.17 1.16 1.15 0.96 0.95 0.91 0.90 0.80 0.79 0.55 0.54 0.53 0.52 0.51
Cl
H
H
H
OH
9
1H-NMR (500 MHz, CDCl3)
1.00
1.08
2.12
1.14
1.21
3.13
3.31
2.85
6.48
0.97
1.18
1.01
f1 (ppm)

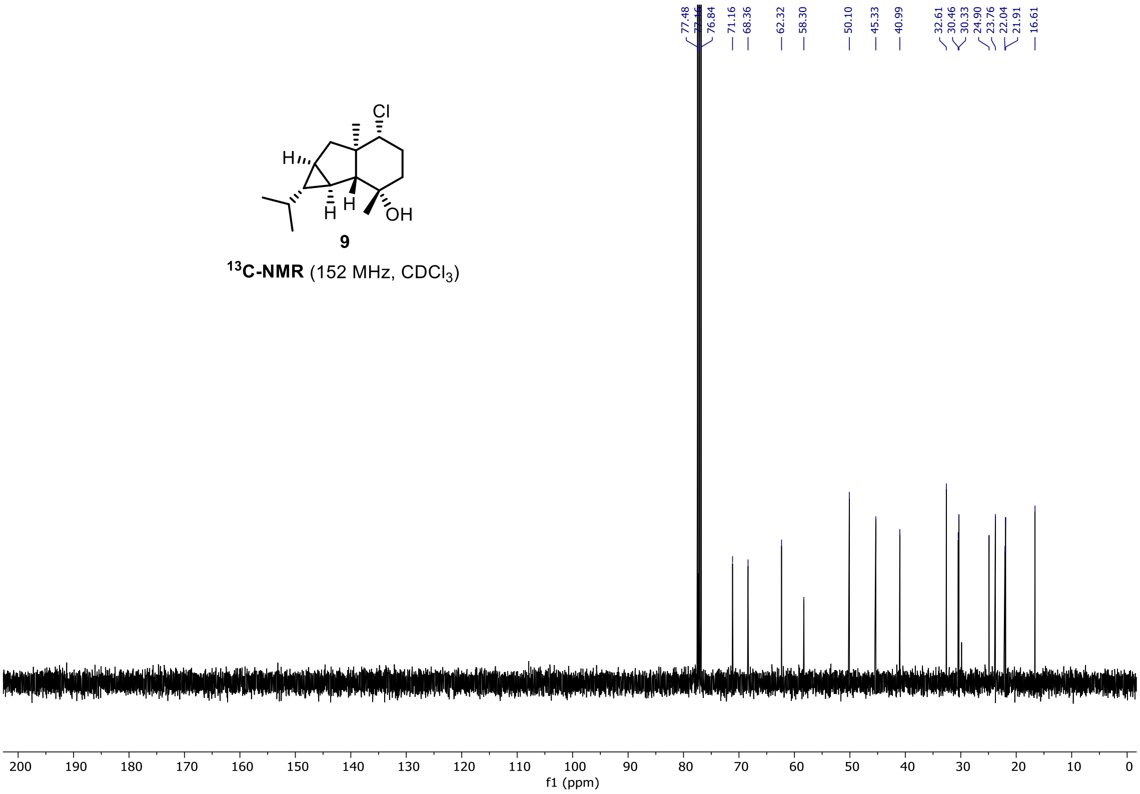
77.48 76.84 71.16 68.36 62.32 58.30 50.10 45.33 40.99 32.61 30.46 30.33 24.90 23.76 22.04 21.91 16.61
Cl
H
H
H
OH
9
13C-NMR (152 MHz, CDCl3)
f1 (ppm)

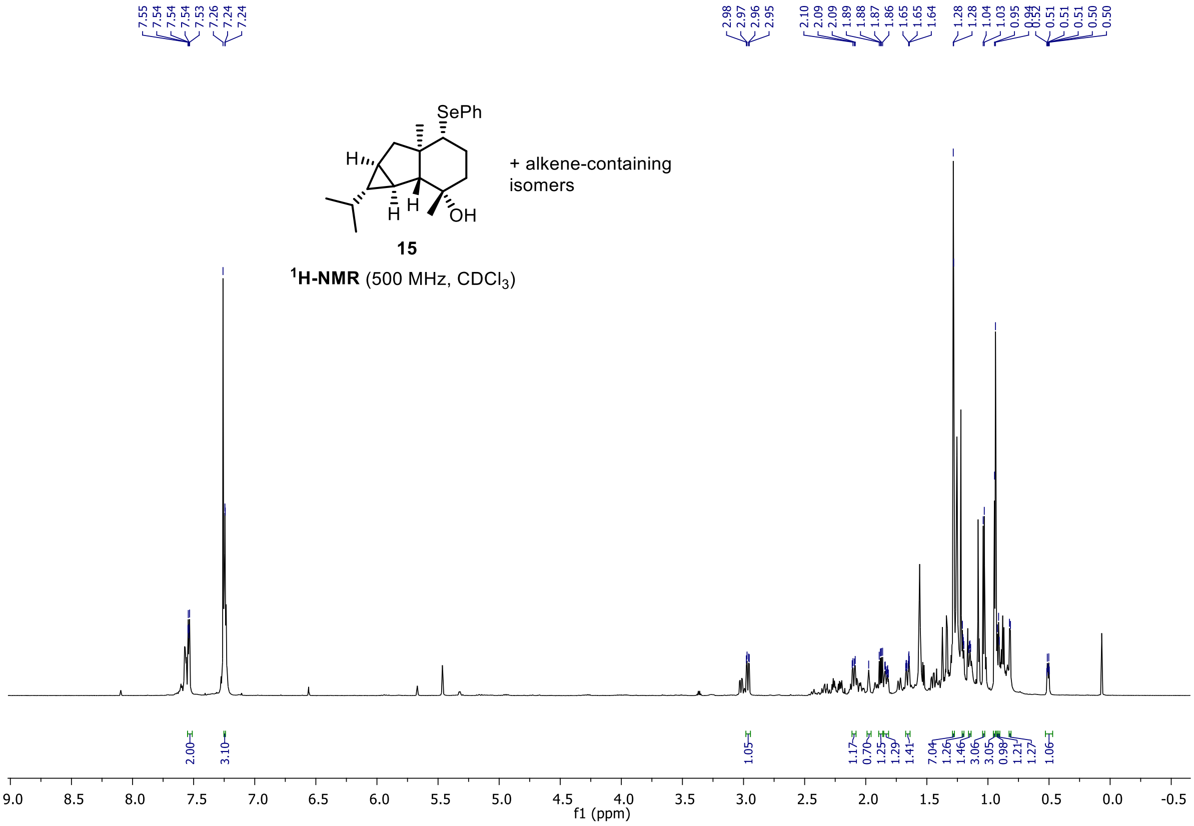
7.55 7.54 7.54 7.54 7.53 7.26 7.24 7.24
2.98 2.97 2.96 2.95
2.10 2.09 2.09 1.89 1.88 1.87 1.86 1.65 1.65 1.64 1.28 1.28 1.04 1.03 0.95 0.51 0.51 0.51 0.50 0.50
SePh
H
H
H
OH
+ alkene-containing isomers
15
1H-NMR (500 MHz, CDCl3)
2.00 3.10 1.05 1.17 0.70 1.25 1.29 1.41 7.04 1.26 1.46 3.06 3.05 0.98 1.21 1.27 1.06
f1 (ppm)

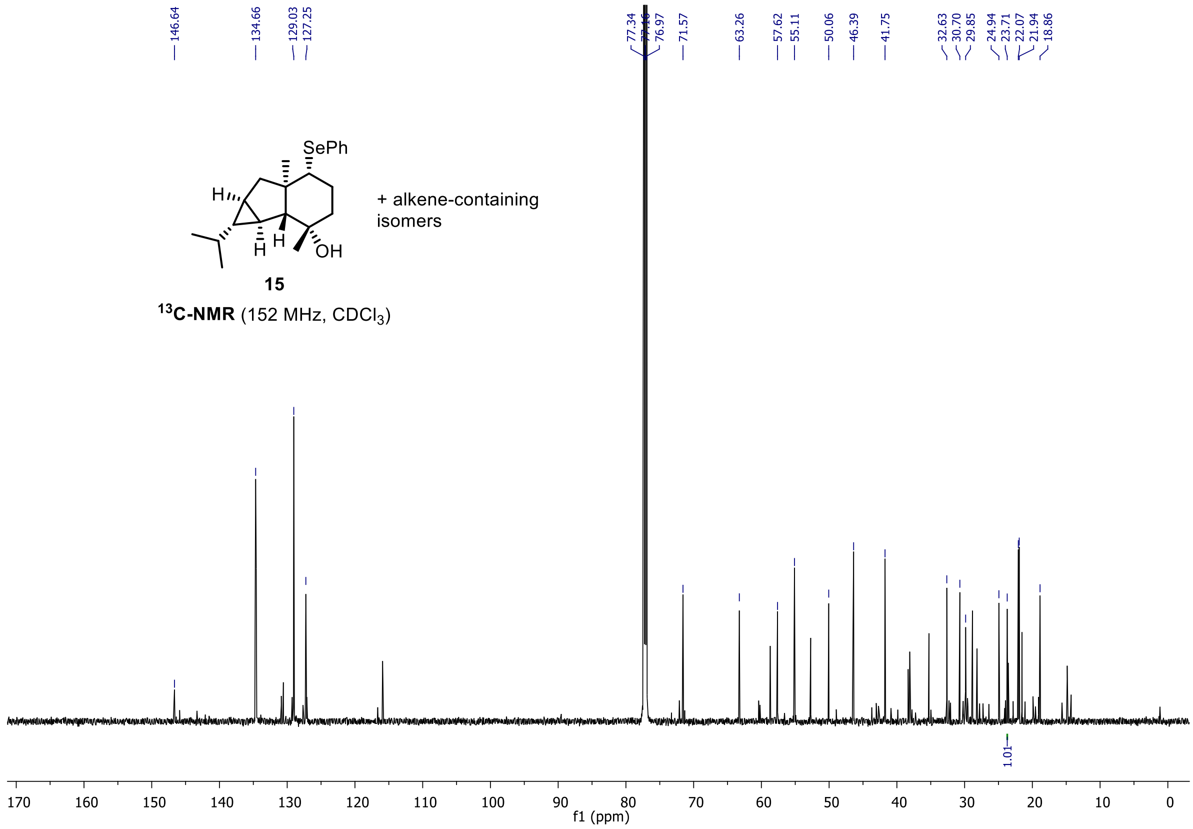
146.64 134.66 129.03 127.25 77.34 76.97 71.57 63.26 57.62 55.11 50.06 46.39 41.75 32.63 30.70 29.85 24.94 23.71 22.07 21.94 18.86
SePh
H
H
H
OH
+ alkene-containing isomers
15
13C-NMR (152 MHz, CDCl3)
1.01
f1 (ppm)

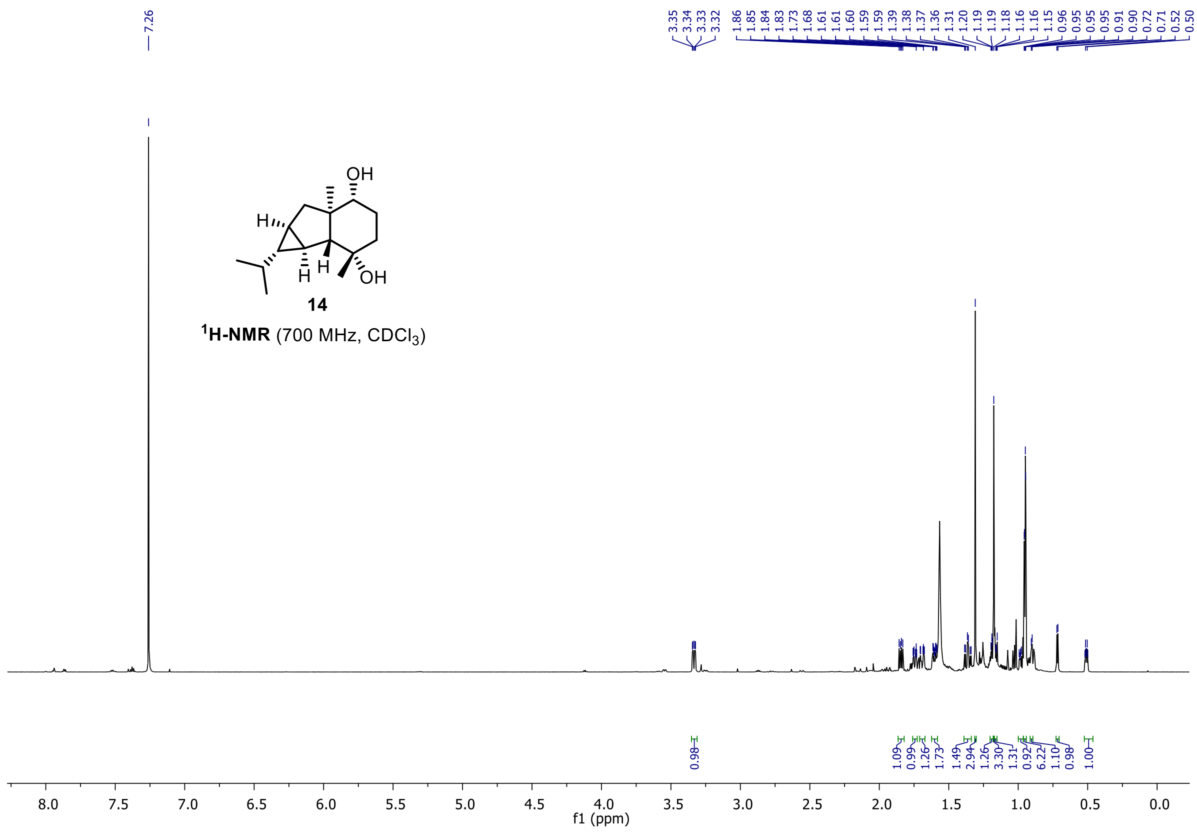
7.26
3.35 3.34 3.33 3.32 1.86 1.85 1.84 1.83 1.73 1.68 1.61 1.61 1.60 1.59 1.59 1.59 1.39 1.38 1.37 1.36 1.31 1.20 1.19 1.19 1.18 1.16 1.15 0.96 0.95 0.95 0.95 0.91 0.90 0.72 0.71 0.52 0.50
OH
H
H
H
OH
14
1H-NMR (700 MHz, CDCl3)
0.98
1.09 0.99 1.26 1.73 1.49 2.94 1.26 3.30 1.31 0.92 6.22 1.10 0.98 1.00
8.0 7.5 7.0 6.5 6.0 5.5 5.0 4.5 4.0 3.5 3.0 2.5 2.0 1.5 1.0 0.5 0.0
f1 (ppm)

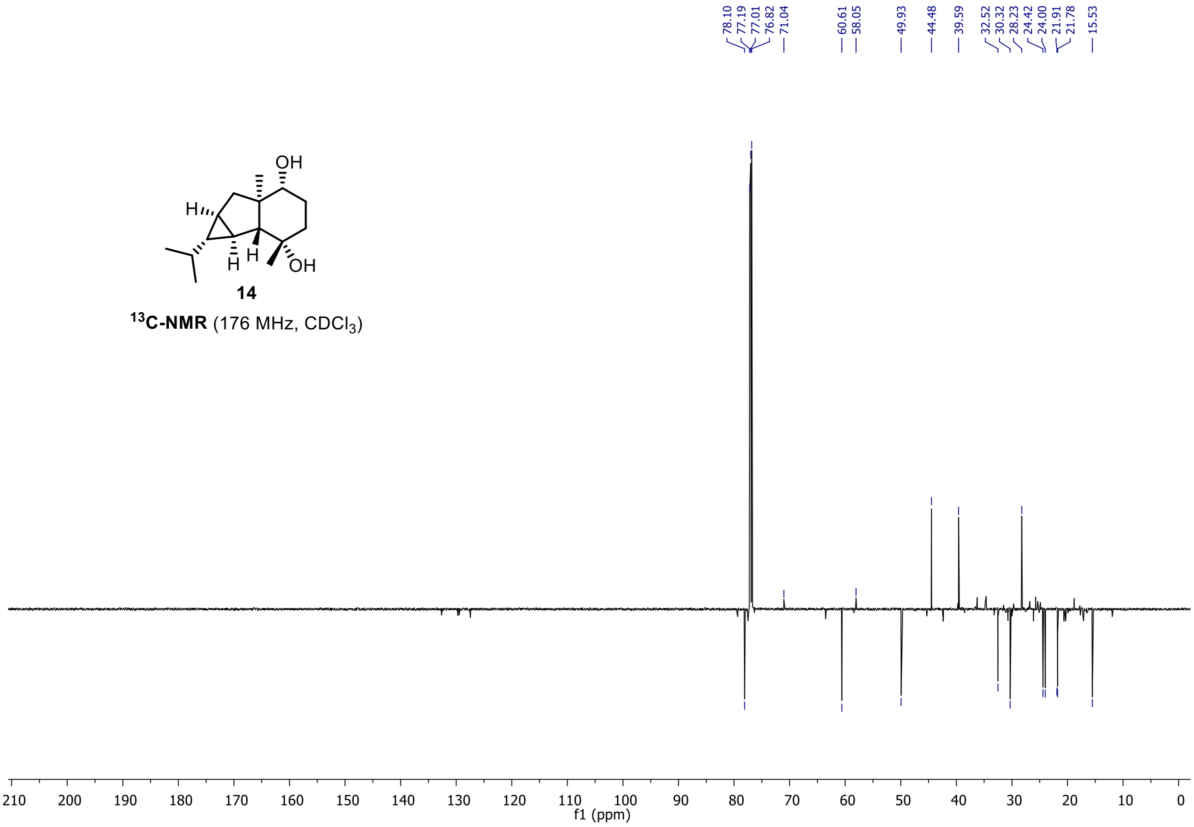
78.10 77.19 77.01 76.82 71.04 60.61 58.05 49.93 44.48 39.59 32.52 30.32 28.23 24.42 24.00 21.91 21.78 15.53
OH
H
H
H
OH
14
13C-NMR (176 MHz, CDCl3)
210 200 190 180 170 160 150 140 130 120 110 100 90 80 70 60 50 40 30 20 10 0
f1 (ppm)

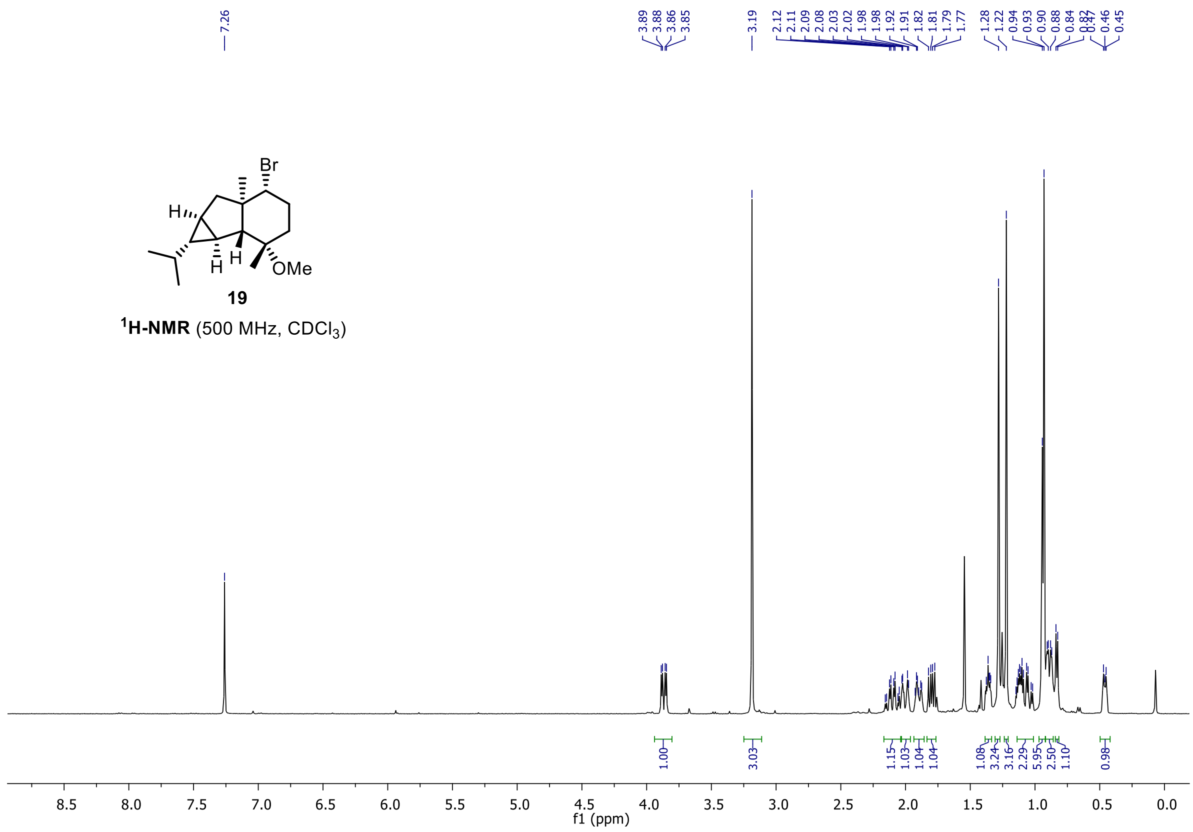

19

^{1}H-NMR (500 MHz, $CDCl_3$)

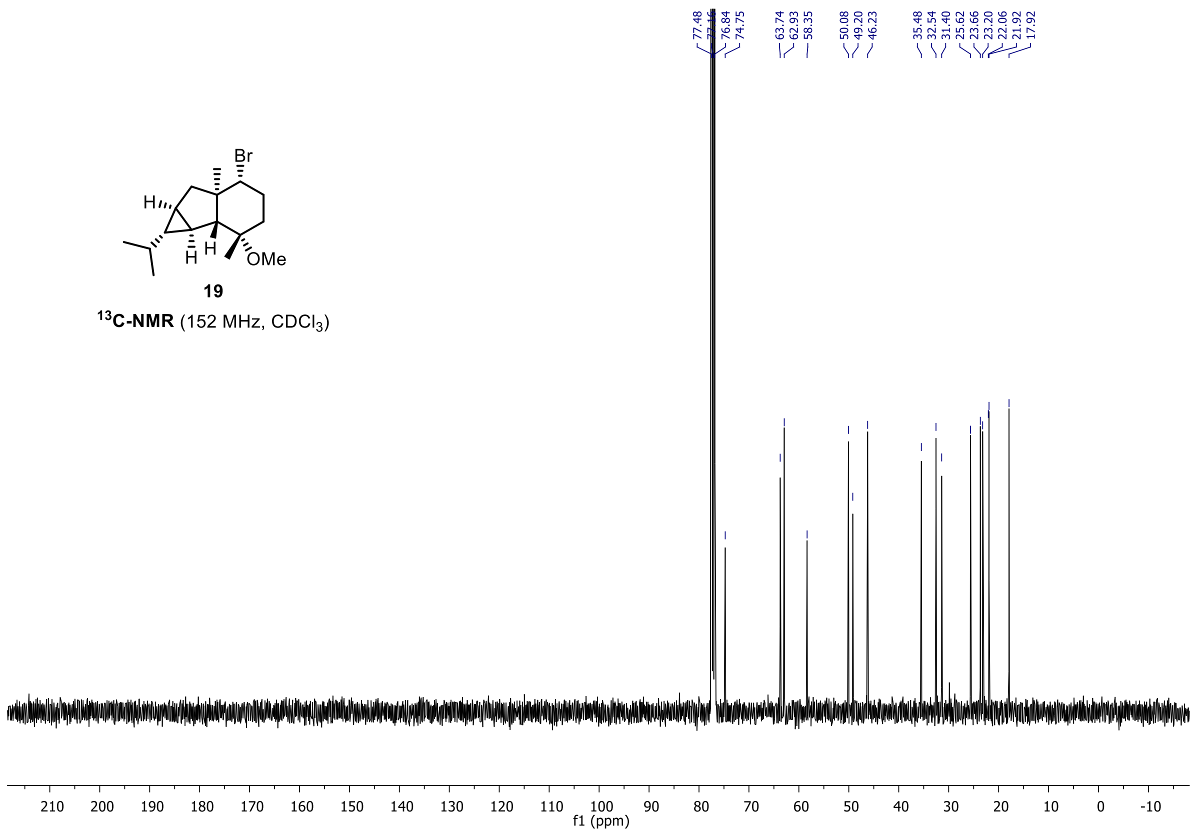

19

^{13}C-NMR (152 MHz, $CDCl_3$)

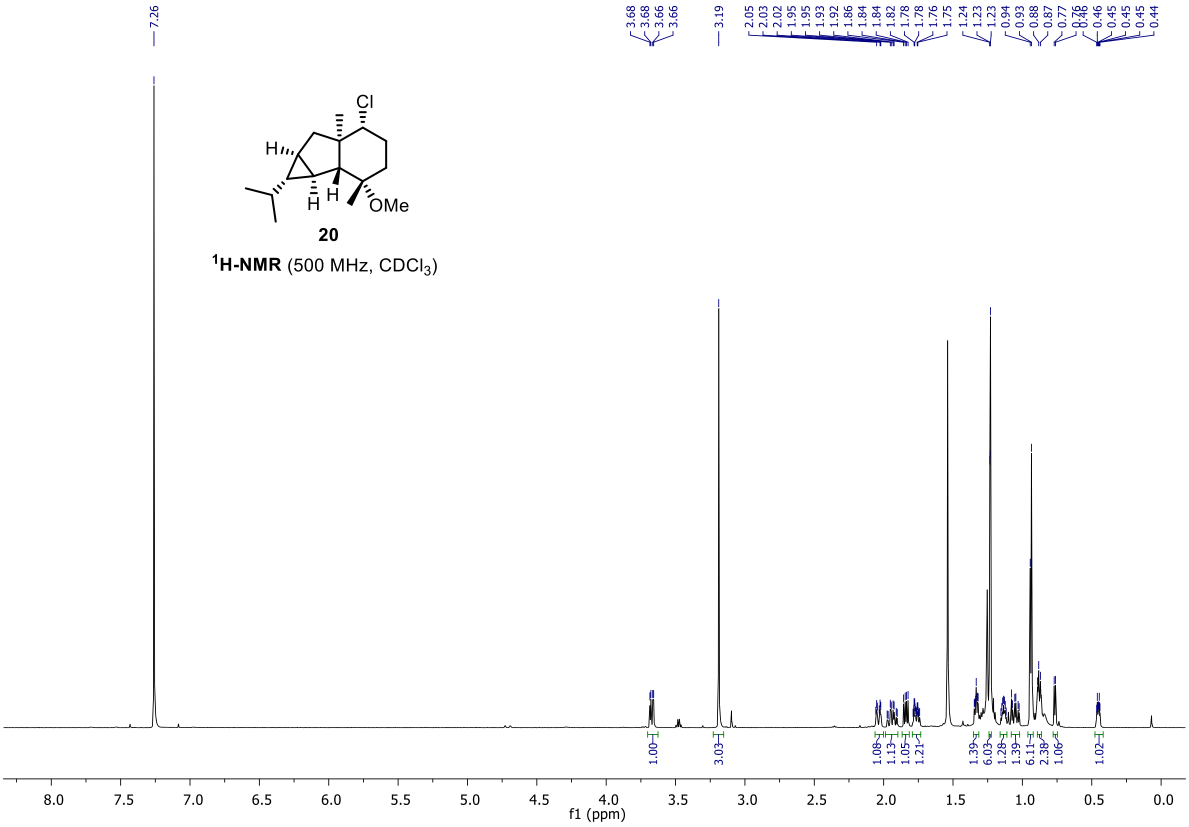

7.26
3.68
3.68
3.66
3.66
3.19
2.05
2.03
2.02
1.95
1.95
1.93
1.92
1.86
1.84
1.84
1.82
1.78
1.78
1.76
1.75
1.24
1.23
1.23
0.94
0.93
0.88
0.87
0.77
0.46
0.45
0.45
0.44
Cl
H
H
H
OMe
20
1H-NMR (500 MHz, CDCl3)
1.00
3.03
1.08
1.13
1.05
1.21
1.39
6.03
1.28
1.39
6.11
2.38
1.06
1.02
8.0 7.5 7.0 6.5 6.0 5.5 5.0 4.5 4.0 3.5 3.0 2.5 2.0 1.5 1.0 0.5 0.0
f1 (ppm)

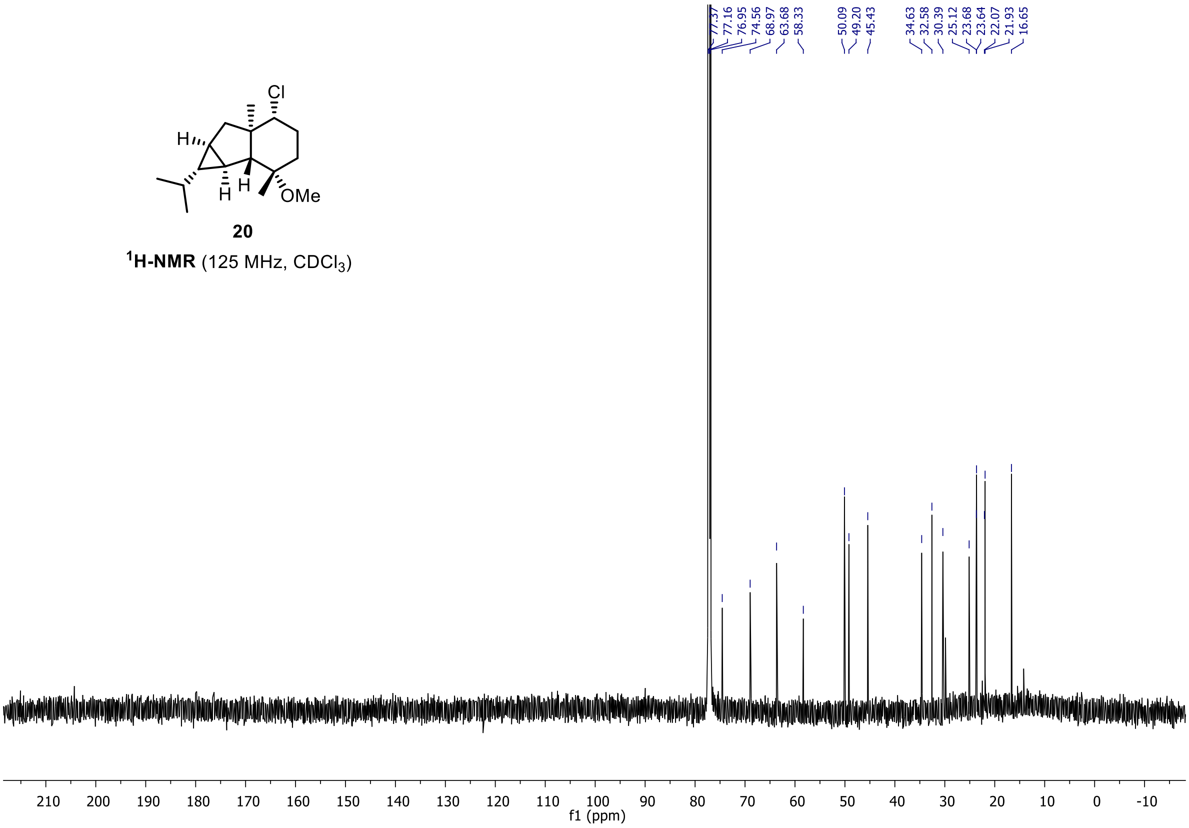

77.37
77.16
76.95
74.56
68.97
63.68
58.33
50.09
49.20
45.43
34.63
32.58
30.39
25.12
23.68
23.64
22.07
21.93
16.65
Cl
H
H
H
OMe
20
1H-NMR (125 MHz, CDCl3)
210 200 190 180 170 160 150 140 130 120 110 100 90 80 70 60 50 40 30 20 10 0 -10
f1 (ppm)

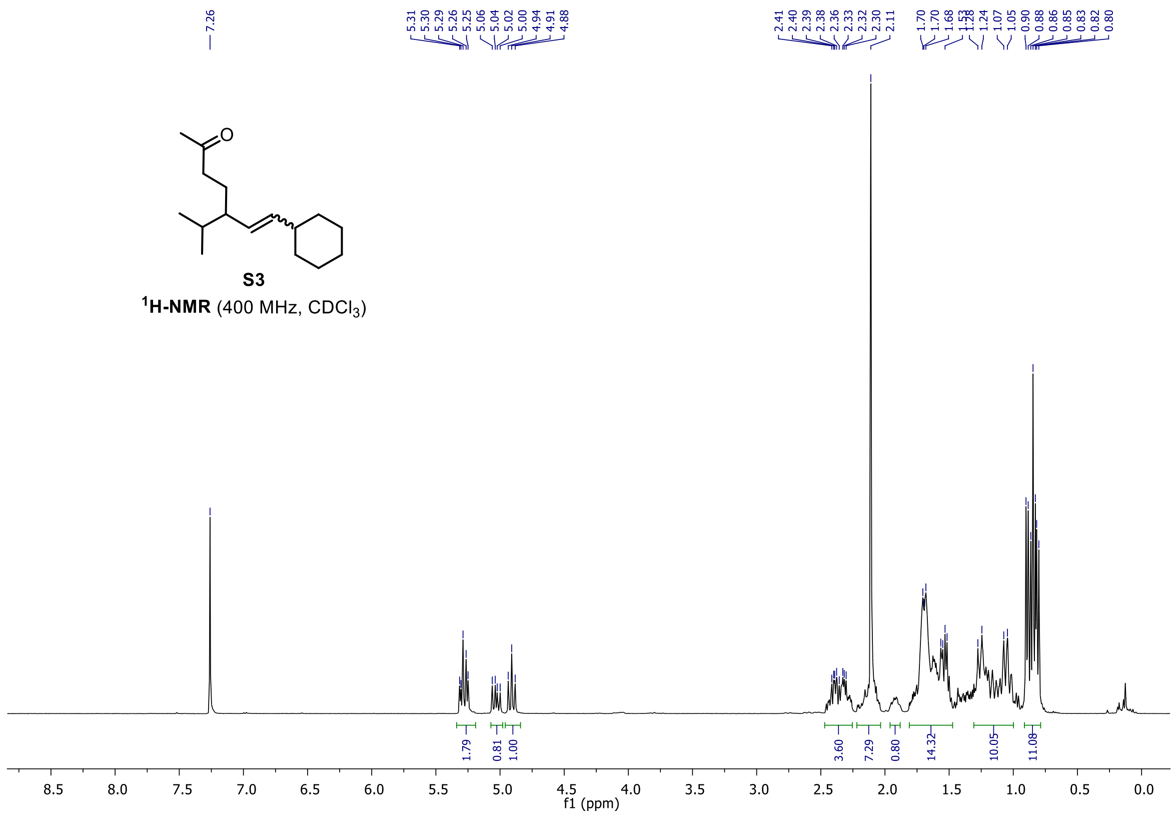

S3

^{1}H-NMR (400 MHz, $CDCl_3$)

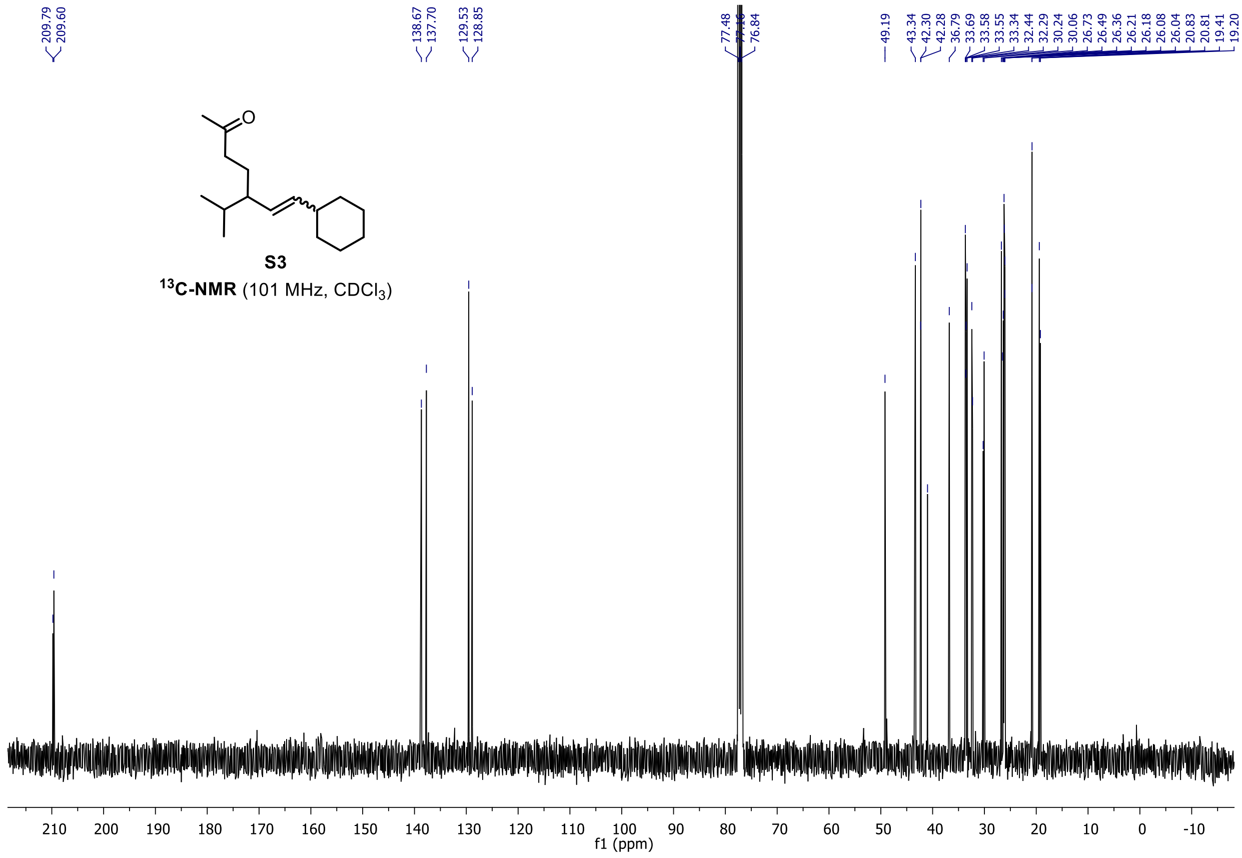

S3

^{13}C-NMR (101 MHz, $CDCl_3$)

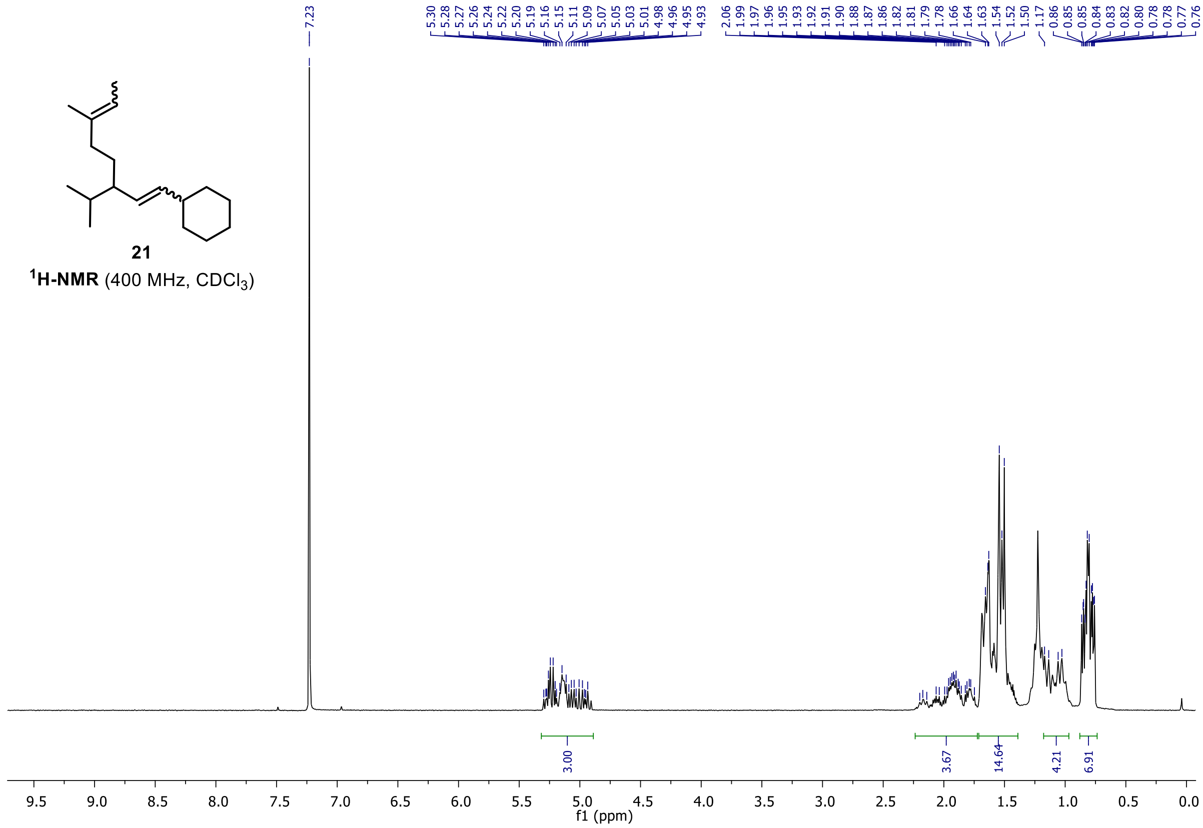

21
1H-NMR (400 MHz, CDCl3)
7.23
5.30 5.28 5.27 5.26 5.24 5.22 5.20 5.19 5.16 5.15 5.11 5.09 5.07 5.05 5.03 5.01 4.98 4.96 4.95 4.93
2.06 1.99 1.97 1.96 1.95 1.93 1.92 1.91 1.90 1.88 1.87 1.86 1.82 1.81 1.79 1.78 1.66 1.64 1.63 1.54 1.52 1.50 1.17 0.86 0.85 0.85 0.84 0.83 0.82 0.80 0.78 0.77 0.76
3.00
3.67
14.64
4.21
6.91
9.5 9.0 8.5 8.0 7.5 7.0 6.5 6.0 5.5 5.0 4.5 4.0 3.5 3.0 2.5 2.0 1.5 1.0 0.5 0.0
f1 (ppm)

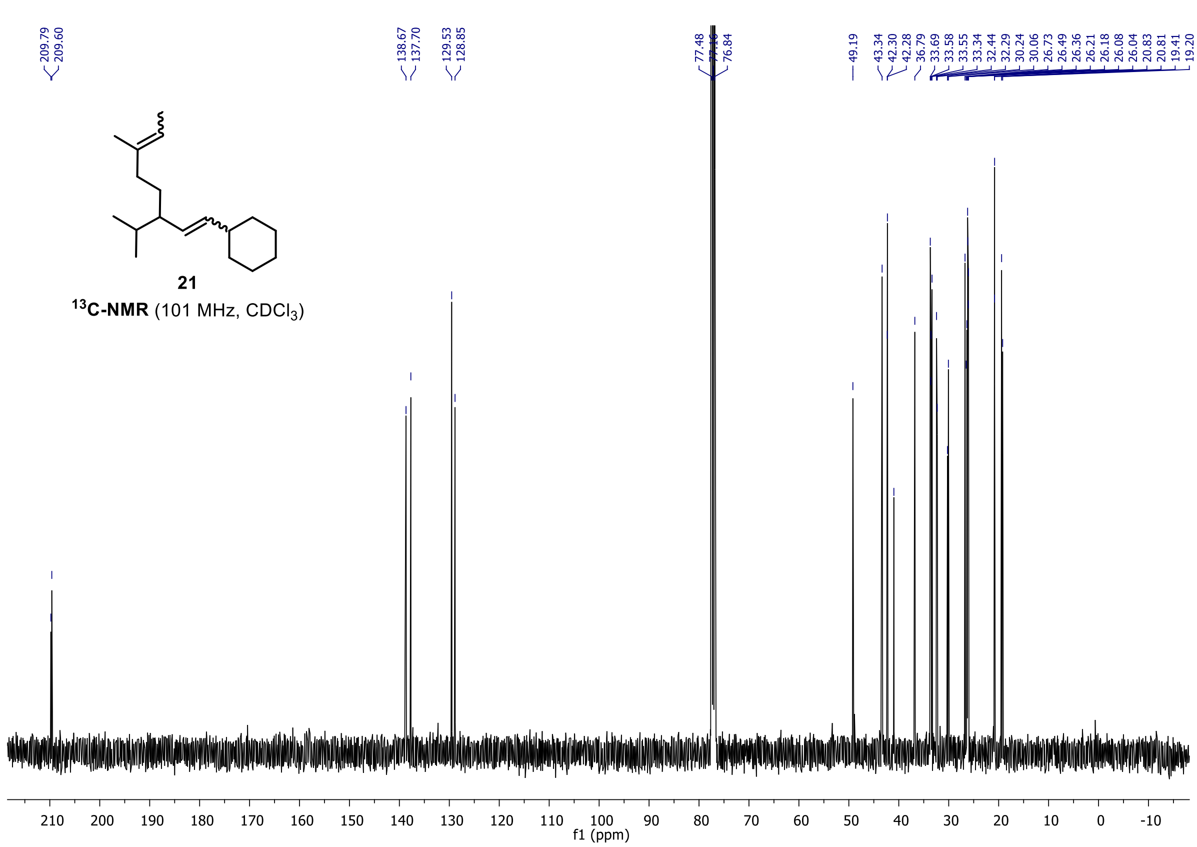

21
13C-NMR (101 MHz, CDCl3)
209.79 209.60
138.67 137.70
129.53 128.85
77.48 77.16 76.84
49.19
43.34 42.30 42.28 36.79 33.69 33.58 33.55 33.34 32.44 32.29 30.24 30.06 26.73 26.49 26.36 26.21 26.18 26.08 26.04 20.83 20.81 19.41 19.20
210 200 190 180 170 160 150 140 130 120 110 100 90 80 70 60 50 40 30 20 10 0 -10
f1 (ppm)

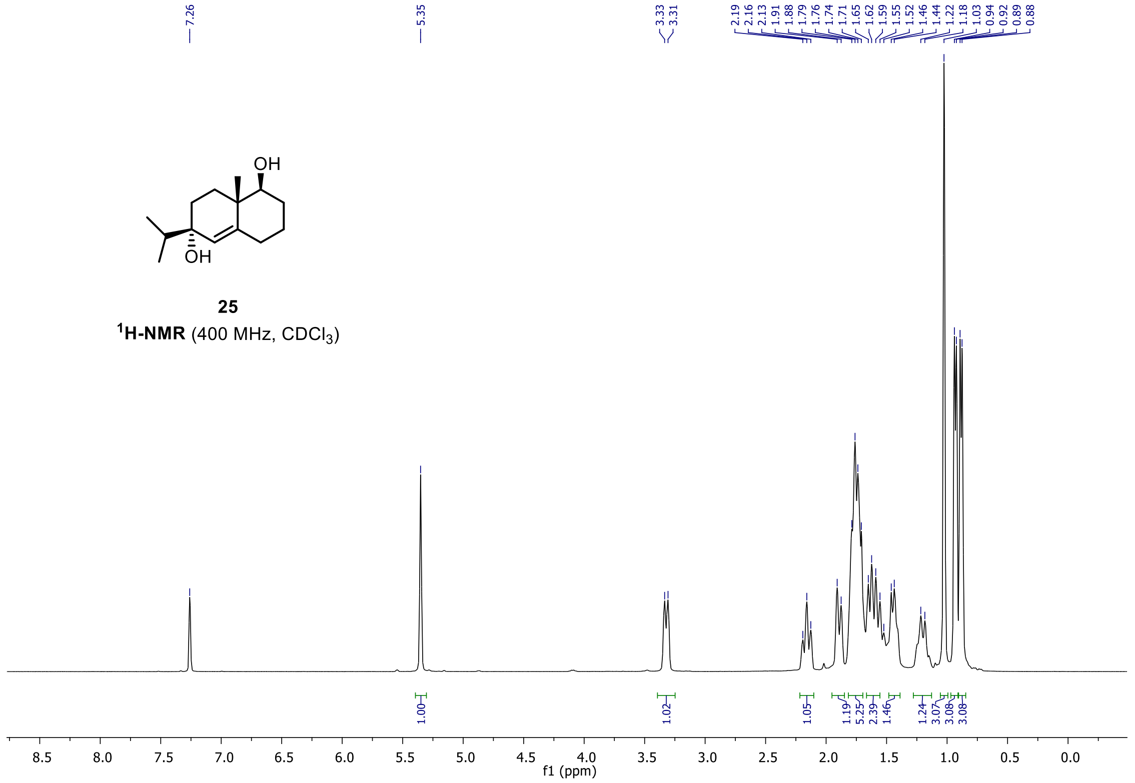
7.26
5.35
3.33
3.31
2.19 2.16 2.13 1.91 1.88 1.79 1.76 1.74 1.71 1.65 1.62 1.59 1.55 1.52 1.46 1.44 1.22 1.18 1.03 0.94 0.92 0.89 0.88
OH
OH
25
^{1}H-NMR (400 MHz, CDCl$_3$)
1.00
1.02
1.05
1.19 5.25 2.39 1.46
1.24 3.07 3.08 3.08
8.5 8.0 7.5 7.0 6.5 6.0 5.5 5.0 4.5 4.0 3.5 3.0 2.5 2.0 1.5 1.0 0.5 0.0
f1 (ppm)

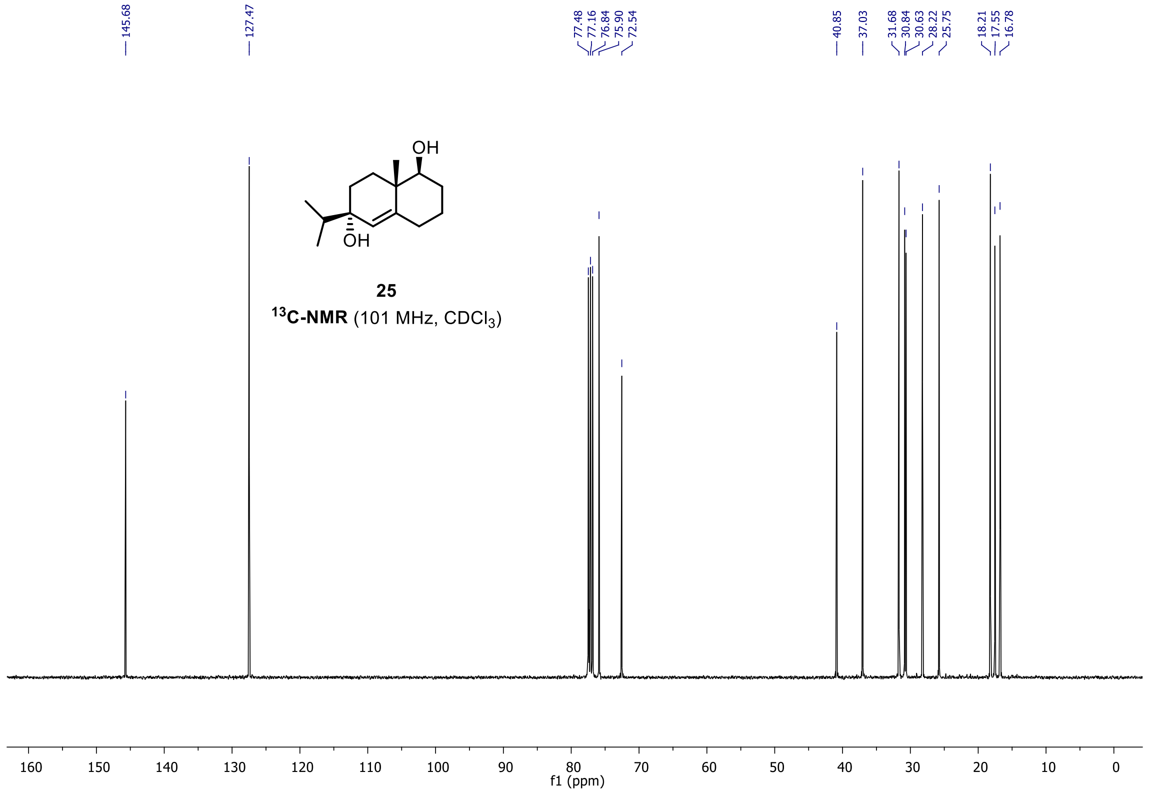
145.68
127.47
77.48 77.16 76.84 75.90 72.54
40.85
37.03
31.68 30.84 30.63 28.22 25.75
18.21 17.55 16.78
OH
OH
25
^{13}C-NMR (101 MHz, CDCl$_3$)
160 150 140 130 120 110 100 90 80 70 60 50 40 30 20 10 0
f1 (ppm)

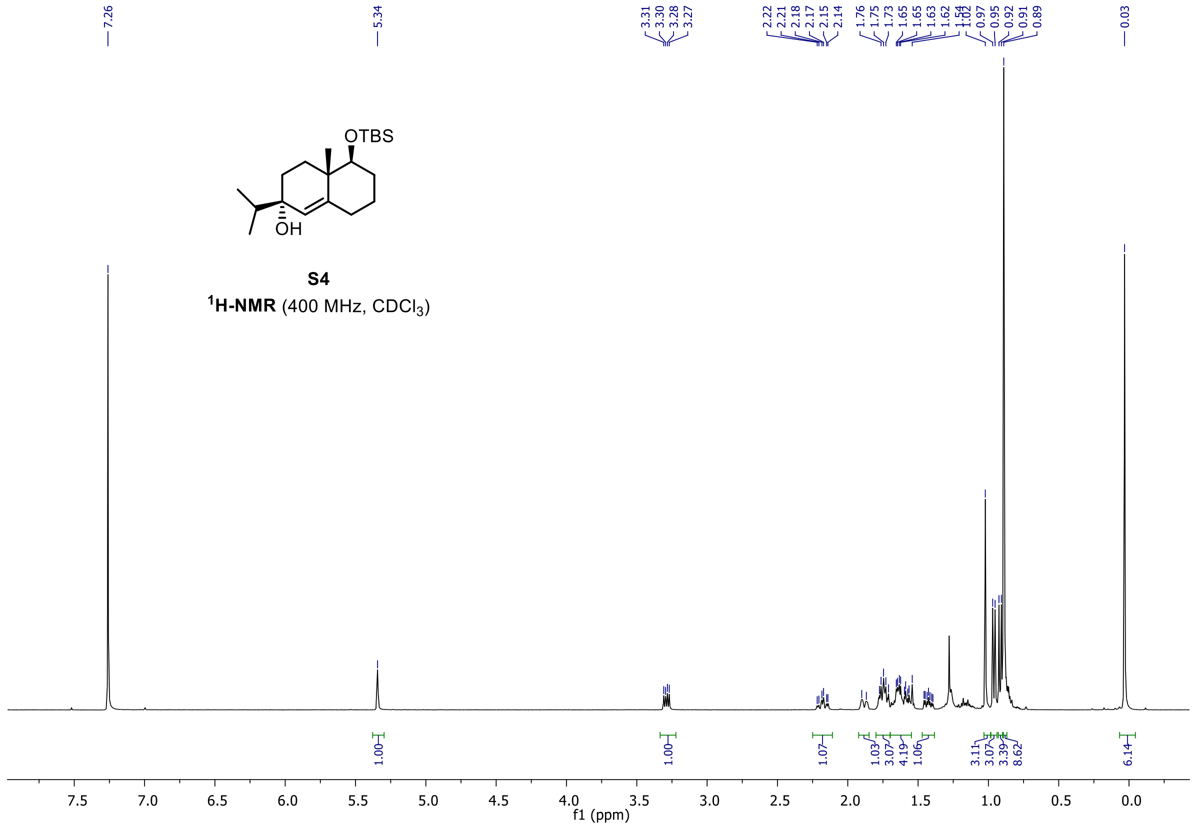

OTBS
OH
S4
1H-NMR (400 MHz, CDCl3)
f1 (ppm)

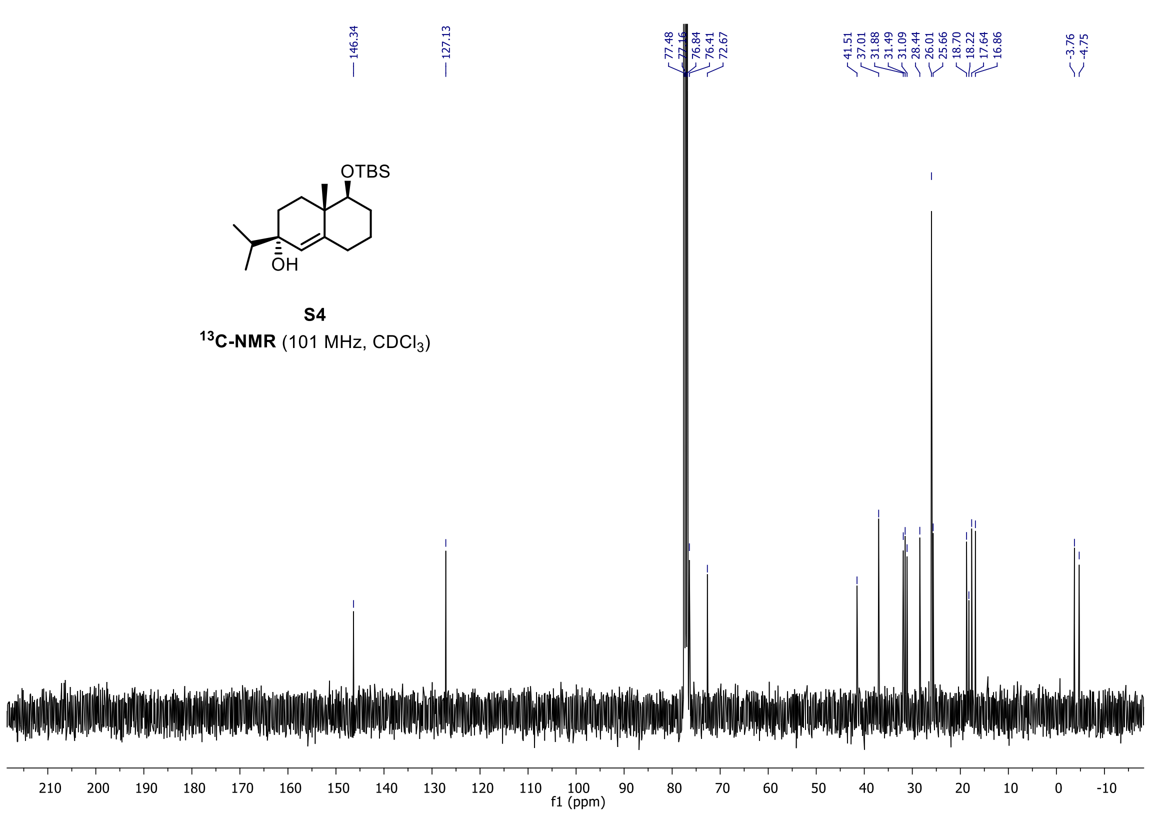

OTBS
OH
S4
13C-NMR (101 MHz, CDCl3)
f1 (ppm)

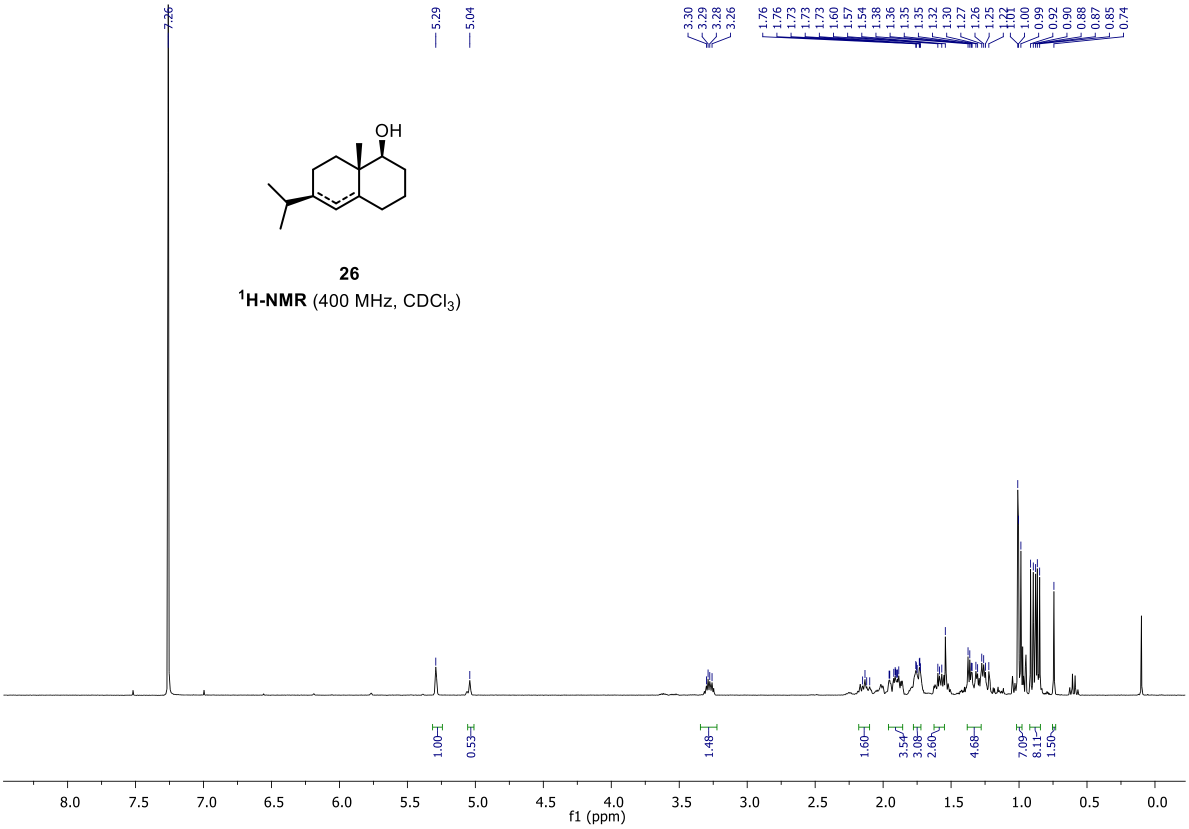
7.26
5.29
5.04
3.30 3.29 3.28 3.26
1.76 1.76 1.73 1.73 1.73 1.60 1.57 1.54 1.38 1.36 1.35 1.35 1.32 1.30 1.27 1.26 1.25 1.22 1.01 1.00 0.99 0.92 0.90 0.88 0.87 0.85 0.74
OH
26
^{1}H-NMR (400 MHz, CDCl$_3$)
1.00 0.53 1.48 1.60 3.54 3.08 2.60 4.68 7.09 8.11 1.50
f1 (ppm)

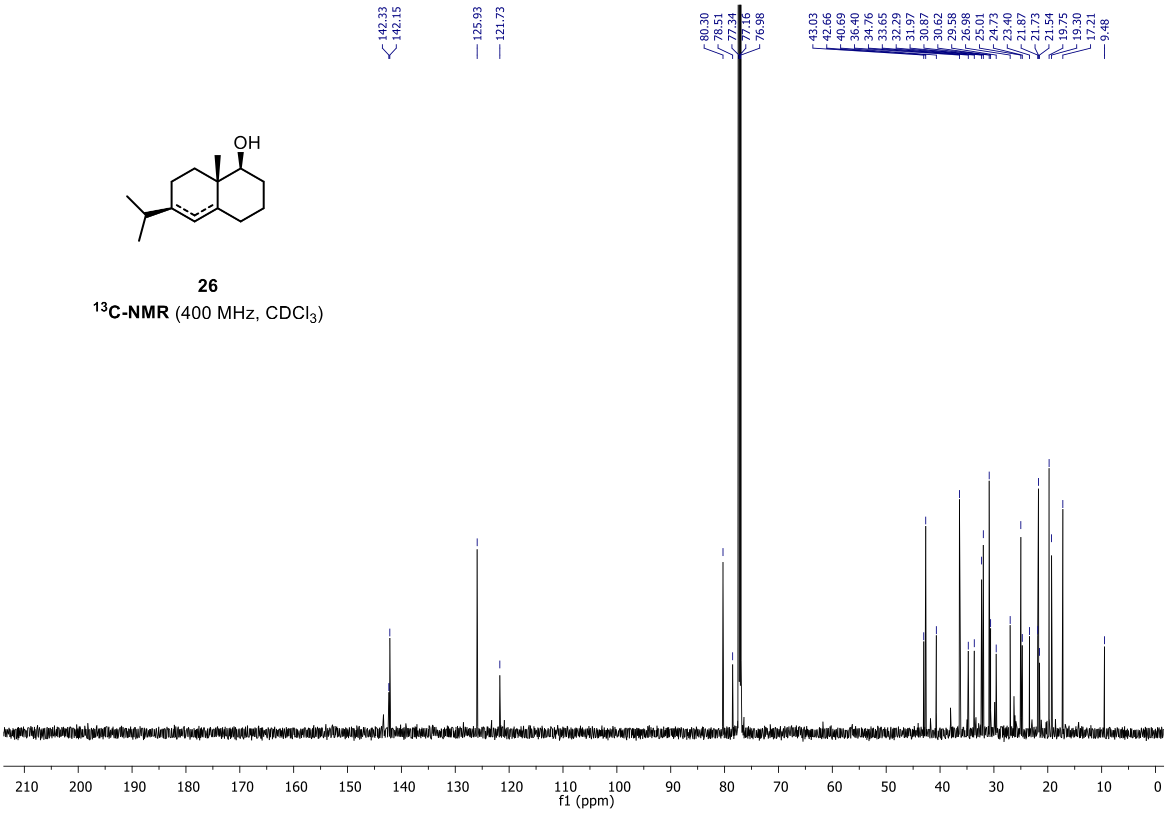
142.33 142.15
125.93
121.73
80.30 78.51 77.34 77.16 76.98
43.03 42.66 40.69 36.40 34.76 33.65 32.29 31.97 30.87 30.62 29.58 26.98 25.01 24.73 23.40 21.87 21.73 21.54 19.75 19.30 17.21 9.48
OH
26
^{13}C-NMR (400 MHz, CDCl$_3$)
f1 (ppm)

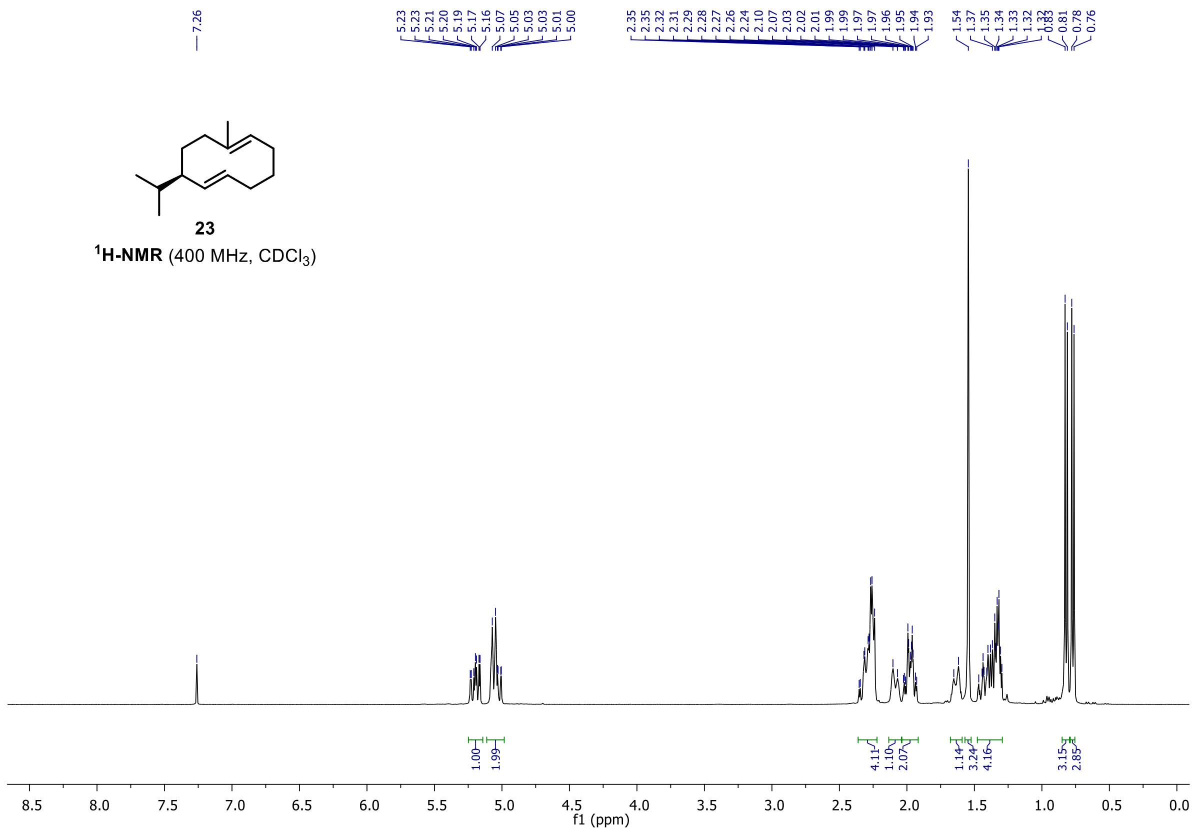
7.26
5.23 5.23 5.21 5.20 5.19 5.17 5.16 5.07 5.05 5.03 5.03 5.01 5.00
2.35 2.35 2.32 2.31 2.29 2.28 2.27 2.26 2.24 2.10 2.07 2.03 2.02 2.01 1.99 1.97 1.97 1.96 1.95 1.94 1.93
1.54 1.37 1.35 1.34 1.33 1.32 0.83 0.81 0.78 0.76
23
^{1}H-NMR (400 MHz, CDCl$_3$)
1.00 1.99 4.11 1.10 2.07 1.14 3.24 4.16 3.15 2.85
8.5 8.0 7.5 7.0 6.5 6.0 5.5 5.0 4.5 4.0 3.5 3.0 2.5 2.0 1.5 1.0 0.5 0.0
f1 (ppm)

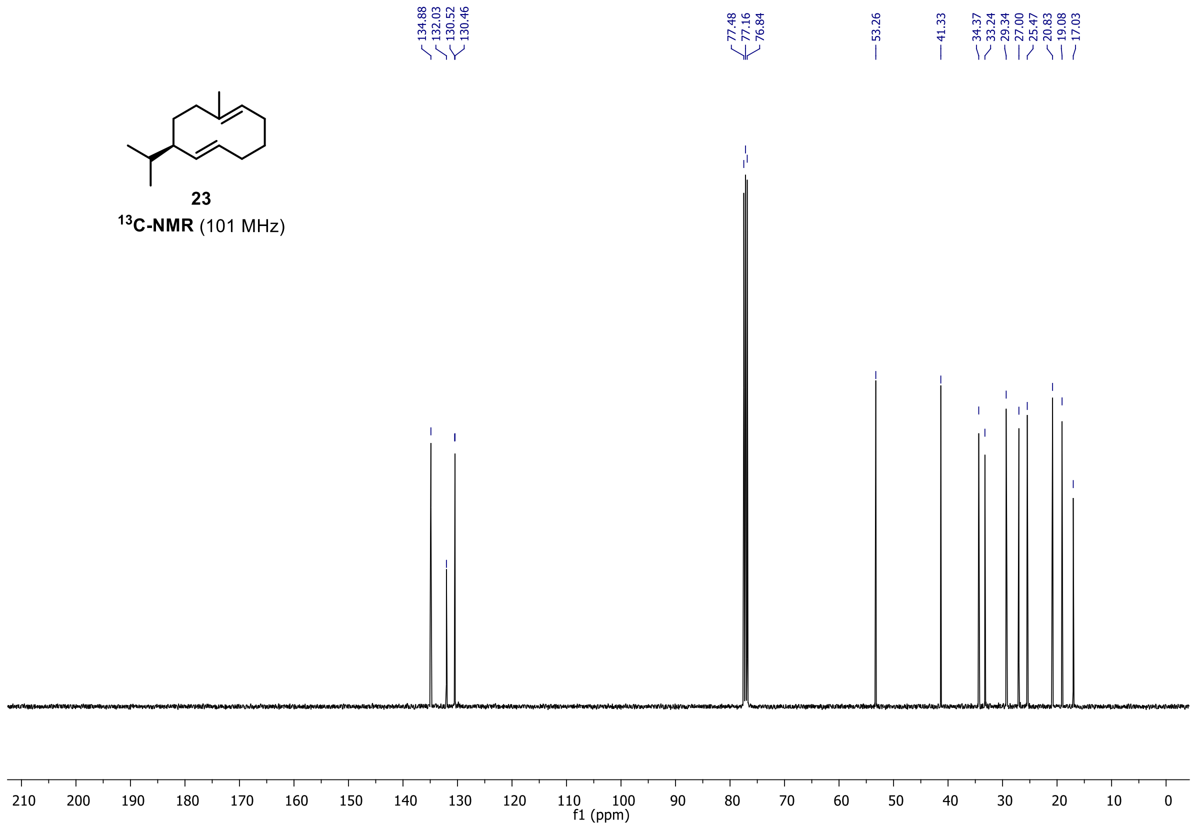
134.88 132.03 130.52 130.46
77.48 77.16 76.84
53.26
41.33
34.37 33.24 29.34 27.00 25.47 20.83 19.08 17.03
23
^{13}C-NMR (101 MHz)
210 200 190 180 170 160 150 140 130 120 110 100 90 80 70 60 50 40 30 20 10 0
f1 (ppm)

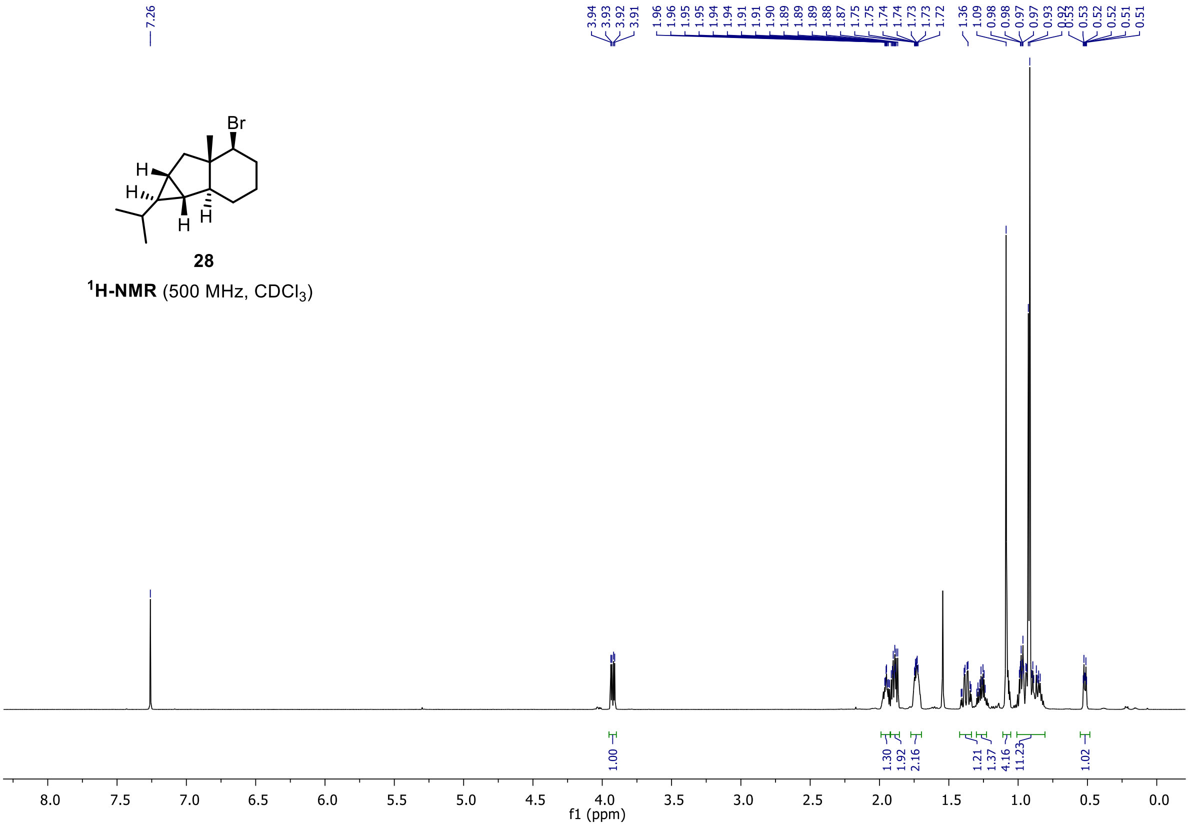

28

^{1}H-NMR (500 MHz, $CDCl_3$)

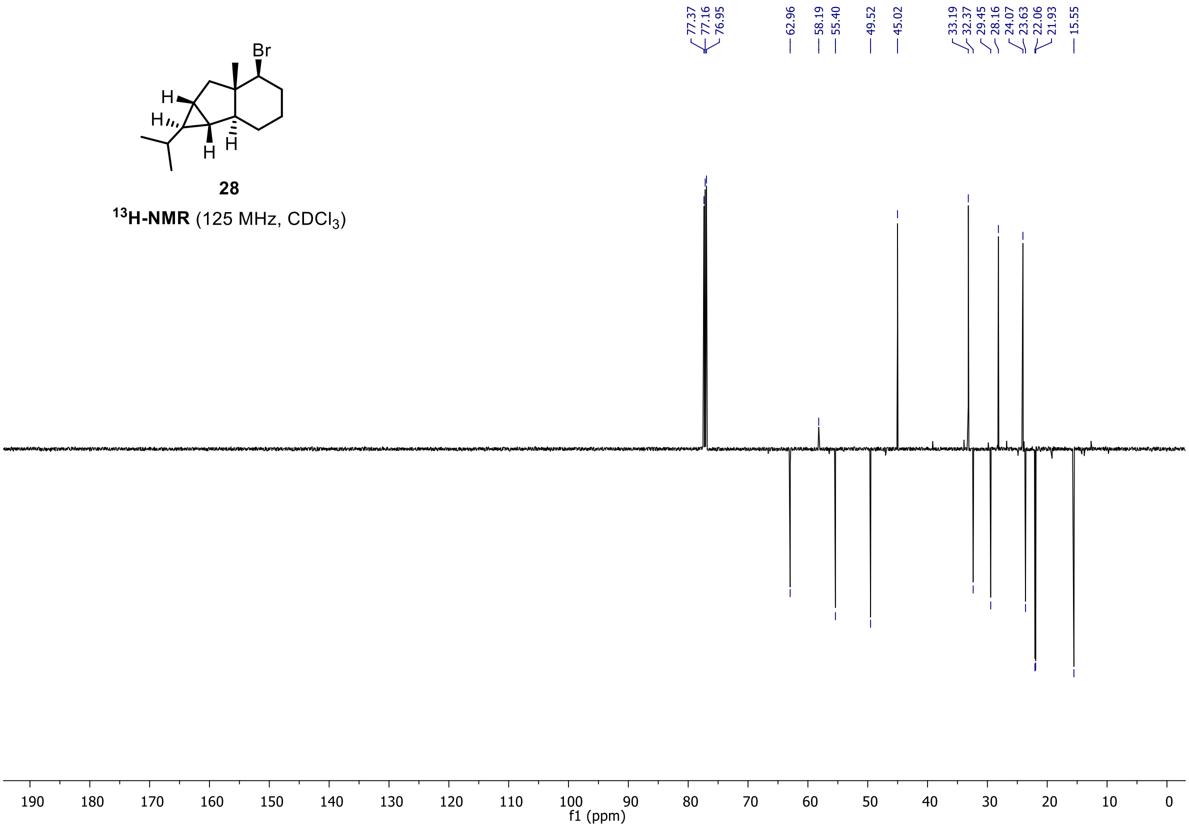

28

^{13}H-NMR (125 MHz, $CDCl_3$)

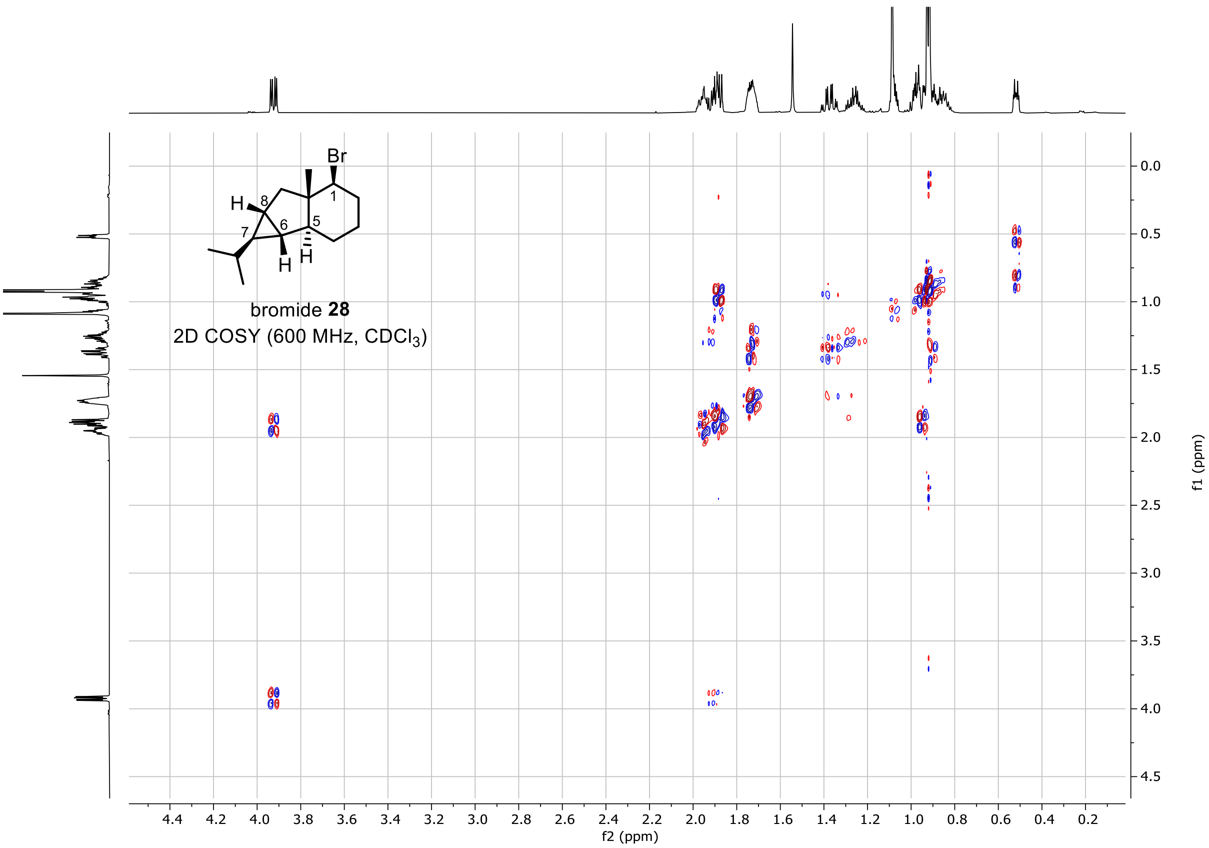

bromide **28**

2D COSY (600 MHz, $CDCl_3$)

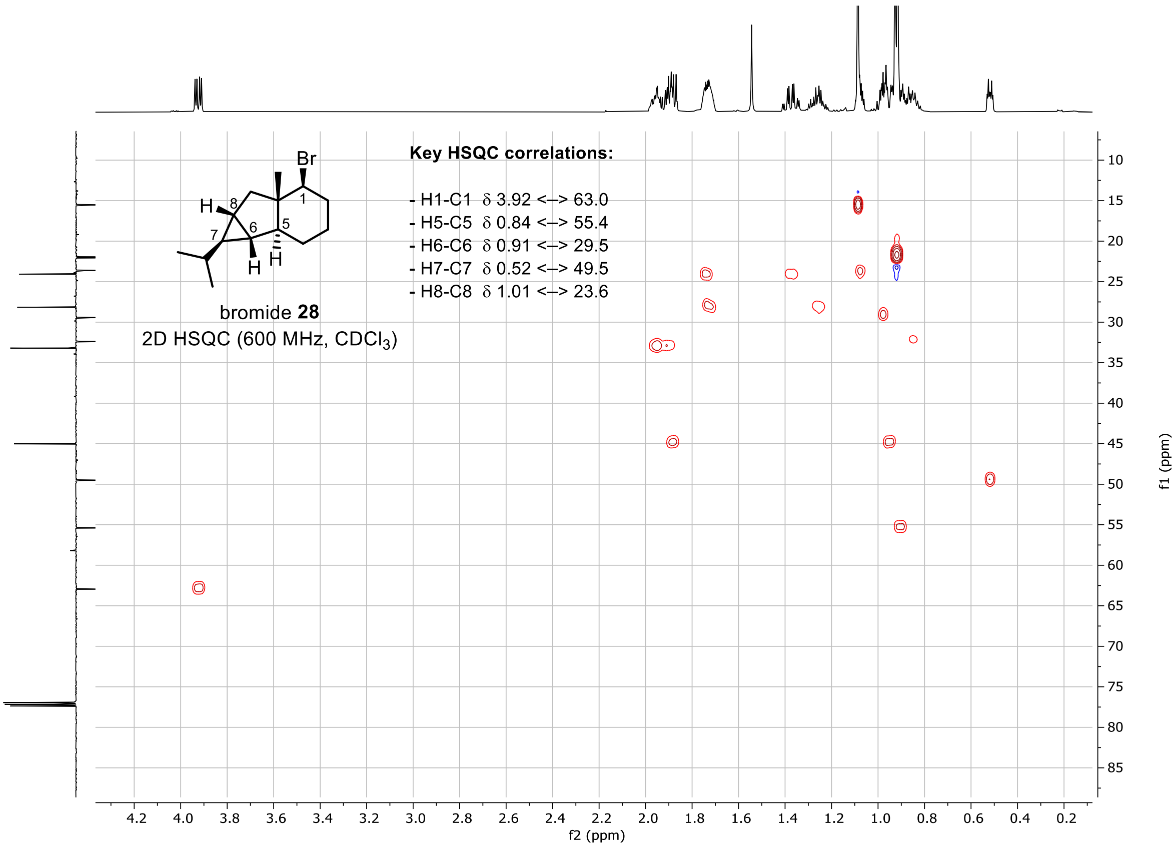

bromide **28**

2D HSQC (600 MHz, $CDCl_3$)

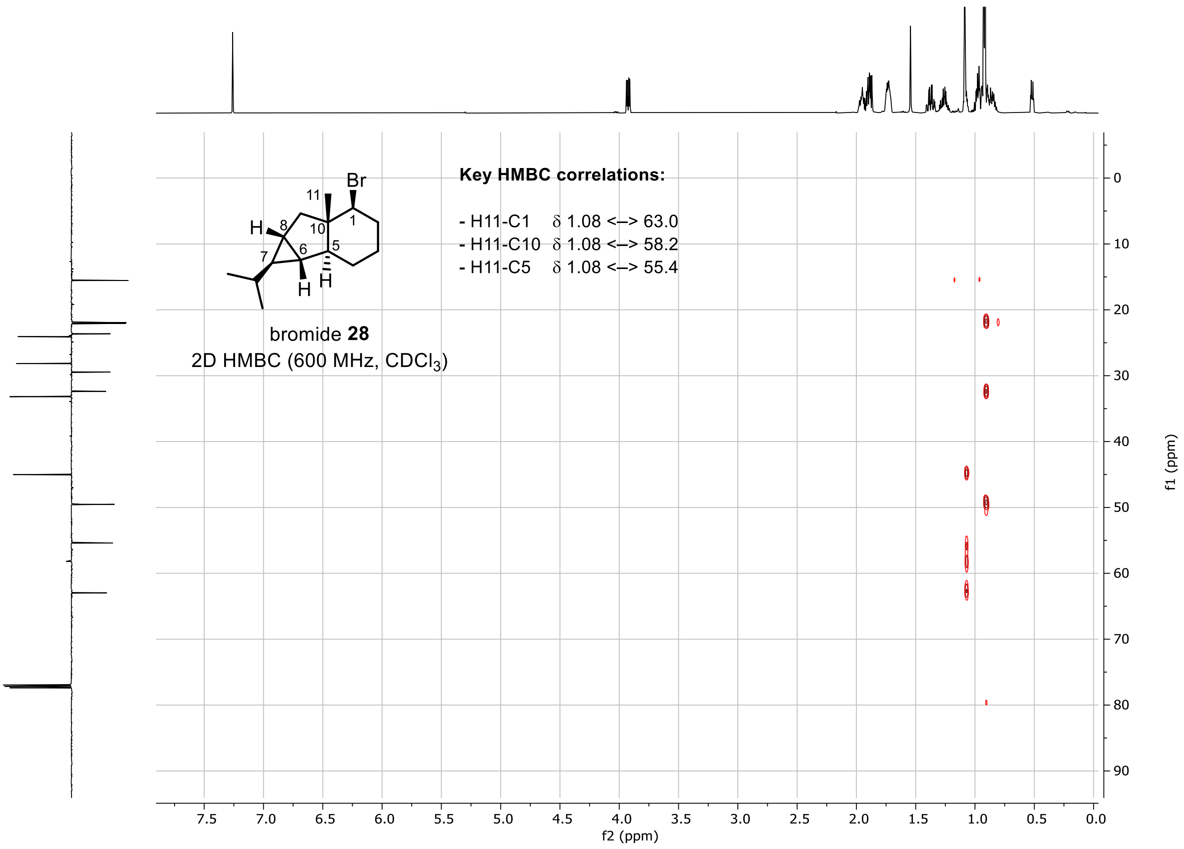
Br
11
H
8
10
1
7
6
5
H
H
bromide **28**
2D HMBC (600 MHz, $CDCl_3$)
Key HMBC correlations:
- H11-C1 δ 1.08 <–> 63.0
- H11-C10 δ 1.08 <–> 58.2
- H11-C5 δ 1.08 <–> 55.4
f2 (ppm)
f1 (ppm)

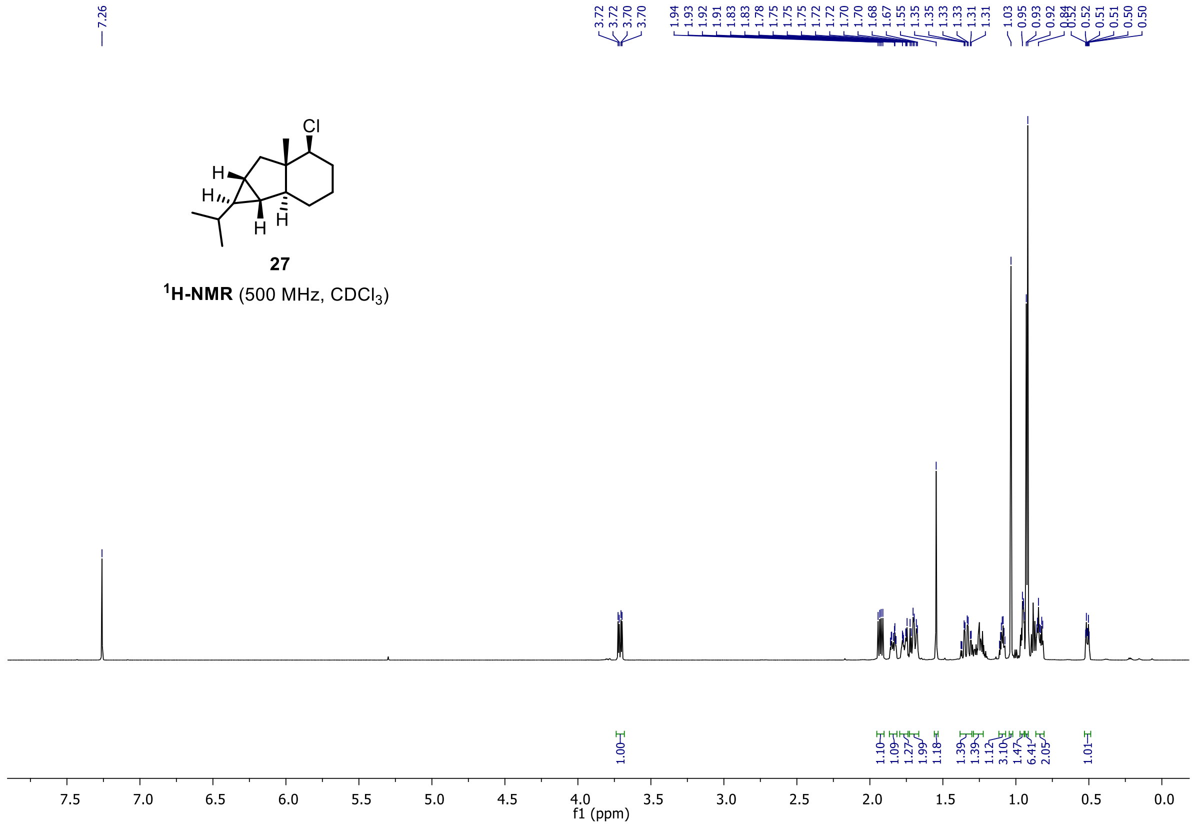

27

^{1}H-NMR (500 MHz, $CDCl_3$)

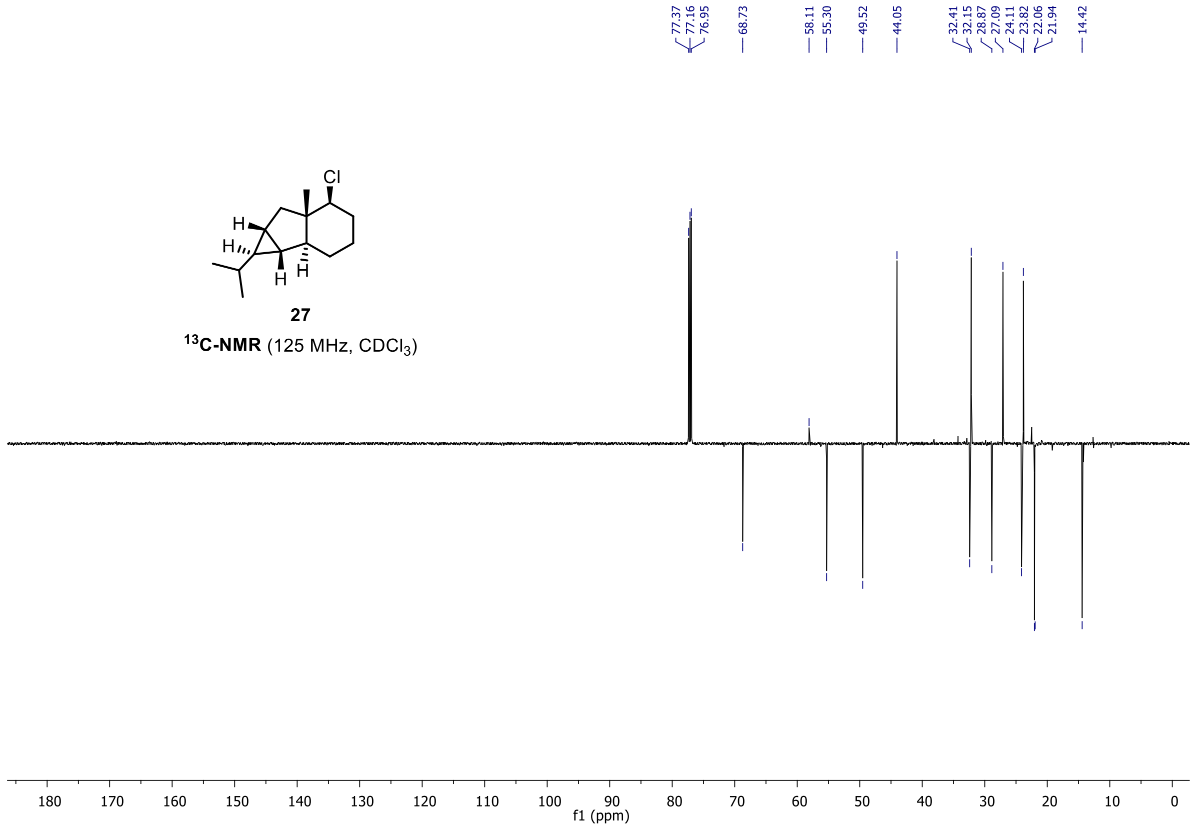

27

^{13}C-NMR (125 MHz, $CDCl_3$)

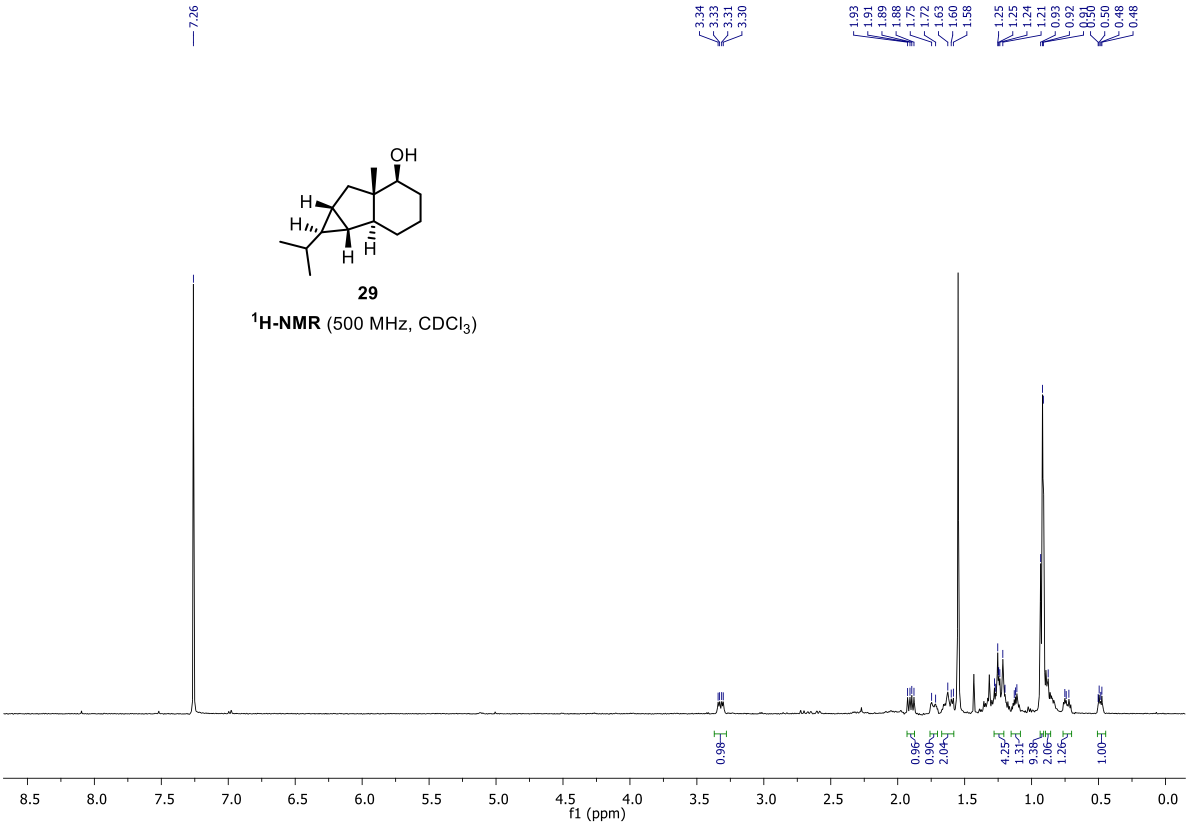
7.26
3.34
3.33
3.31
3.30
1.93
1.91
1.89
1.88
1.75
1.72
1.63
1.60
1.58
1.25
1.25
1.24
1.21
0.93
0.92
0.91
0.50
0.48
0.48
OH
H
H
H
H
29
1H-NMR (500 MHz, CDCl3)
0.98
0.96
0.90
2.04
4.25
1.31
9.38
2.06
1.26
1.00
f1 (ppm)

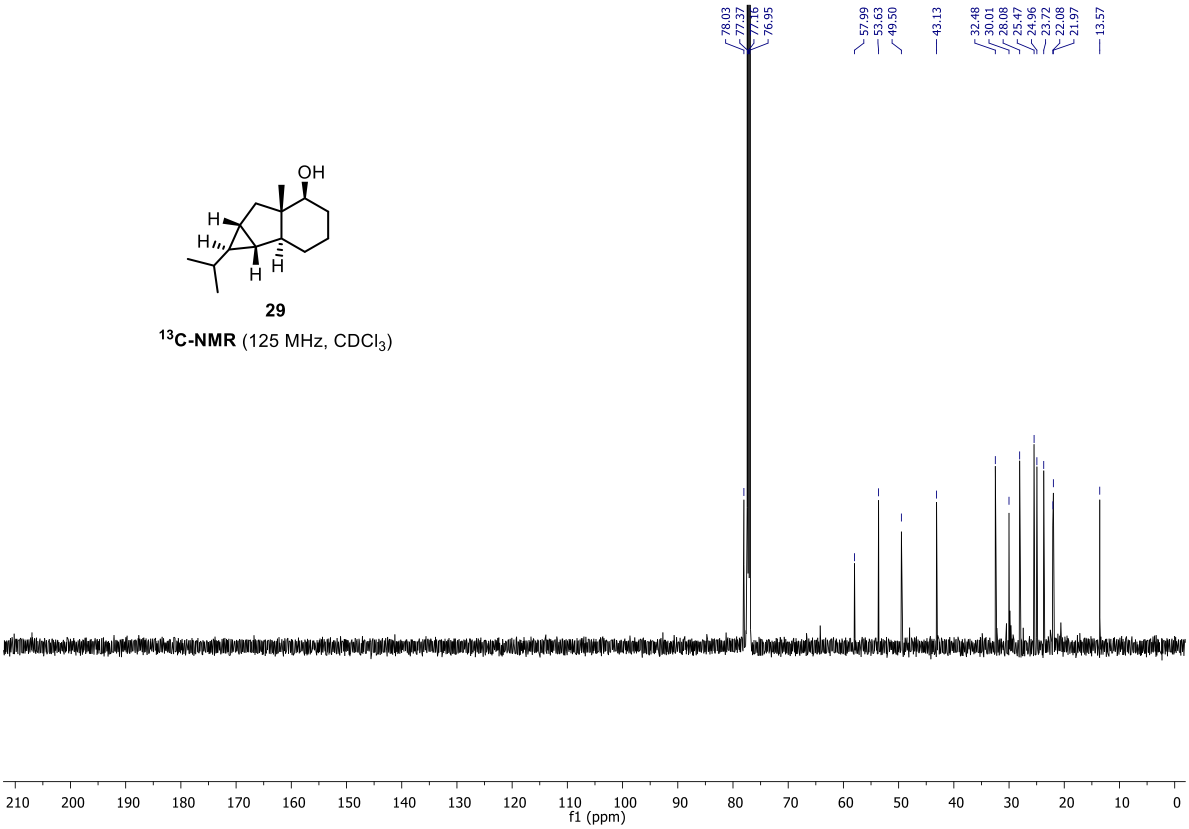
78.03
77.37
77.16
76.95
57.99
53.63
49.50
43.13
32.48
30.01
28.08
25.47
24.96
23.72
22.08
21.97
13.57
OH
H
H
H
H
29
13C-NMR (125 MHz, CDCl3)
f1 (ppm)

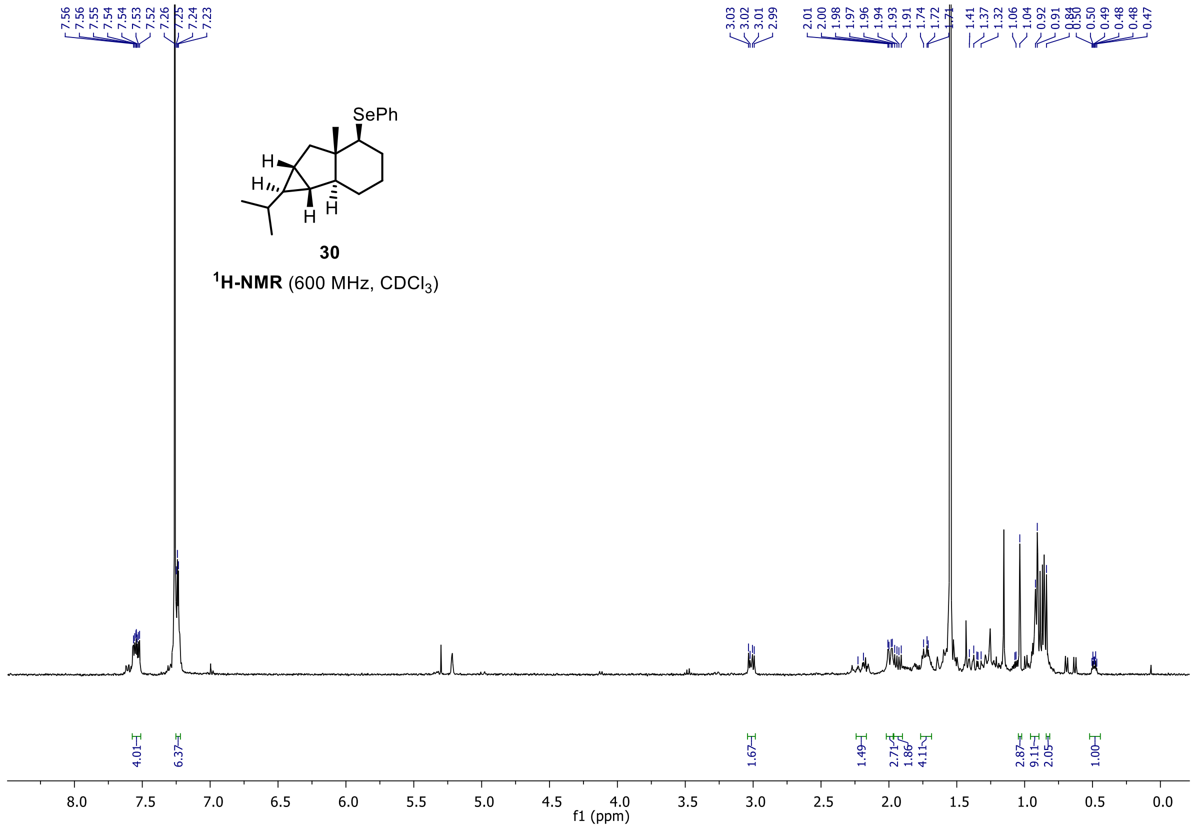
7.56 7.56 7.55 7.54 7.54 7.53 7.52 7.26 7.25 7.24 7.23
3.03 3.02 3.01 2.99
2.01 2.00 1.98 1.97 1.96 1.94 1.93 1.91 1.74 1.72 1.71
1.41 1.37 1.32 1.06 1.04 0.92 0.91 0.88 0.50 0.50 0.49 0.48 0.48 0.47
SePh
H
H
H
H
30
1H-NMR (600 MHz, CDCl3)
4.01 6.37 1.67 1.49 2.71 1.86 4.11 2.87 9.11 2.05 1.00
8.0 7.5 7.0 6.5 6.0 5.5 5.0 4.5 4.0 3.5 3.0 2.5 2.0 1.5 1.0 0.5 0.0
f1 (ppm)

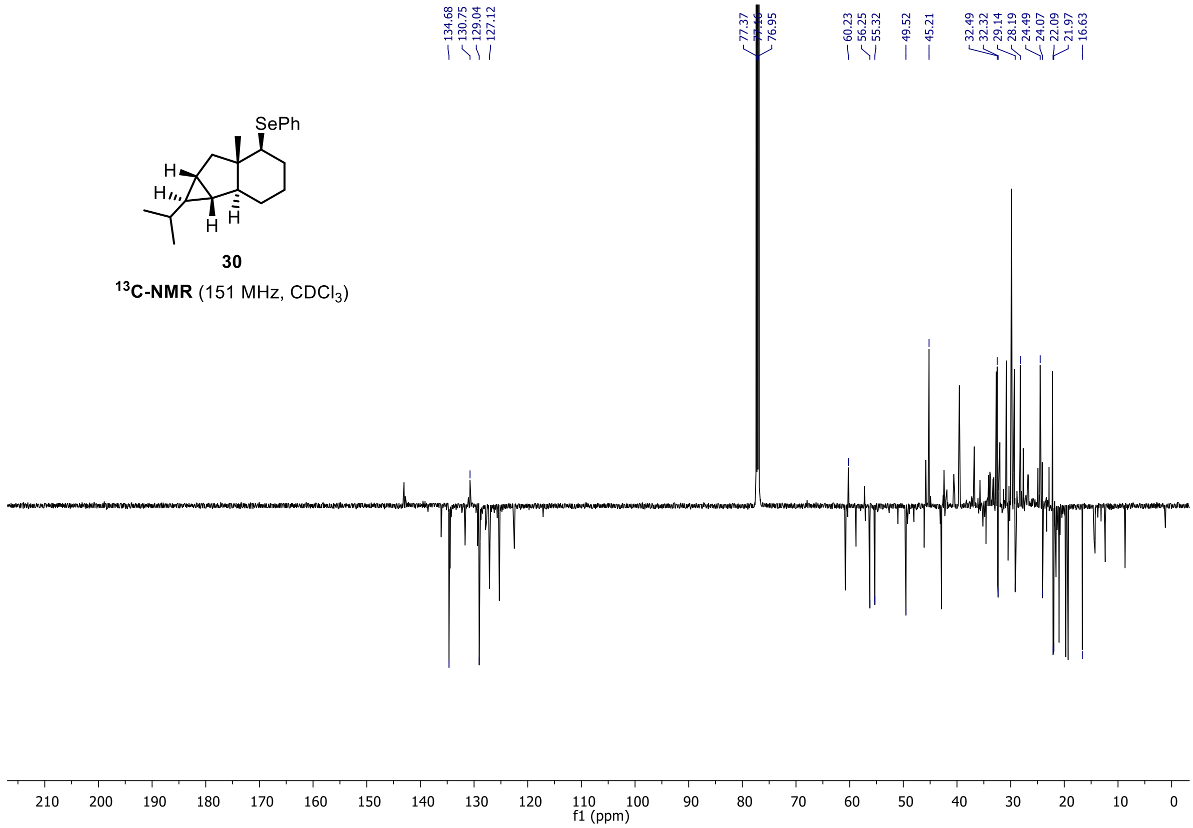
134.68 130.75 129.04 127.12
77.37 77.16 76.95
60.23 56.25 55.32 49.52 45.21
32.49 32.32 29.14 28.19 24.49 24.07 22.09 21.97 16.63
SePh
H
H
H
H
30
13C-NMR (151 MHz, CDCl3)
210 200 190 180 170 160 150 140 130 120 110 100 90 80 70 60 50 40 30 20 10 0
f1 (ppm)

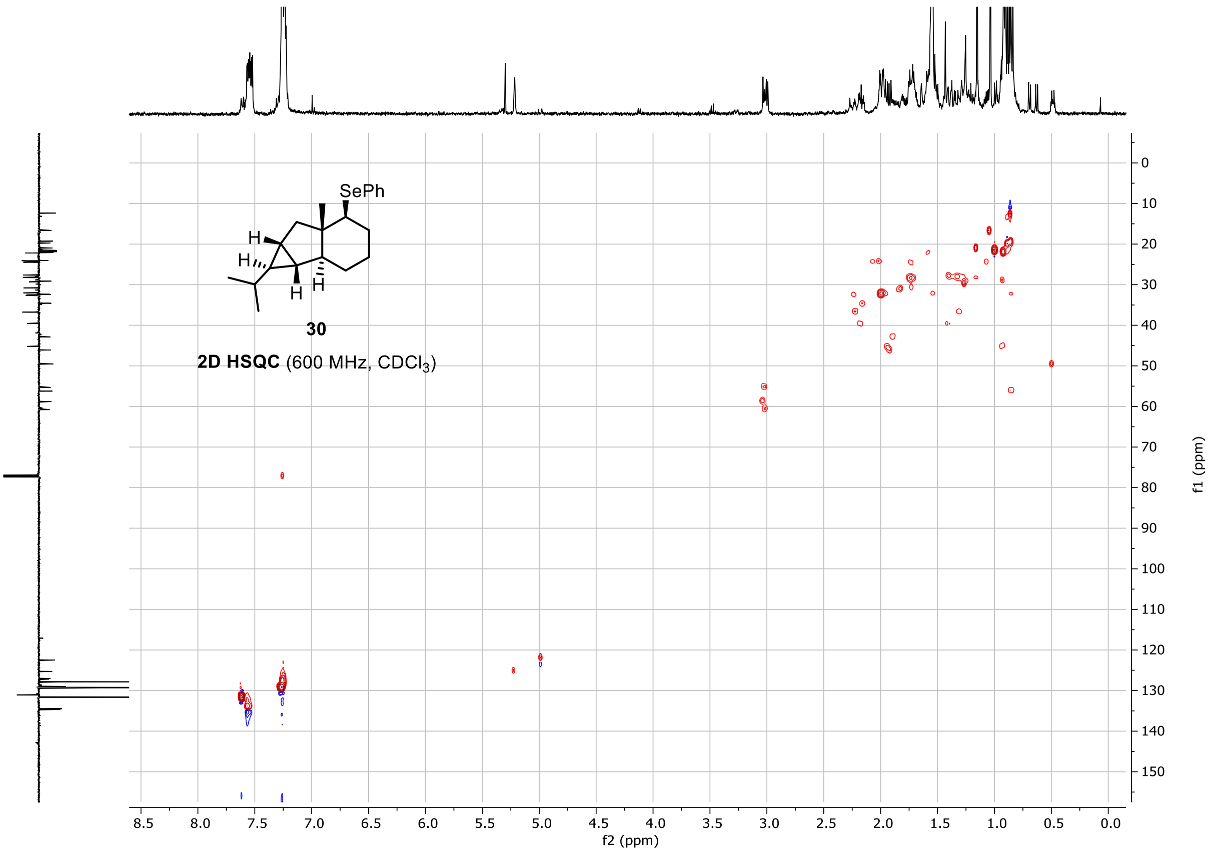
SePh
H
H
H
H
30
2D HSQC (600 MHz, CDCl3)
f2 (ppm)
f1 (ppm)

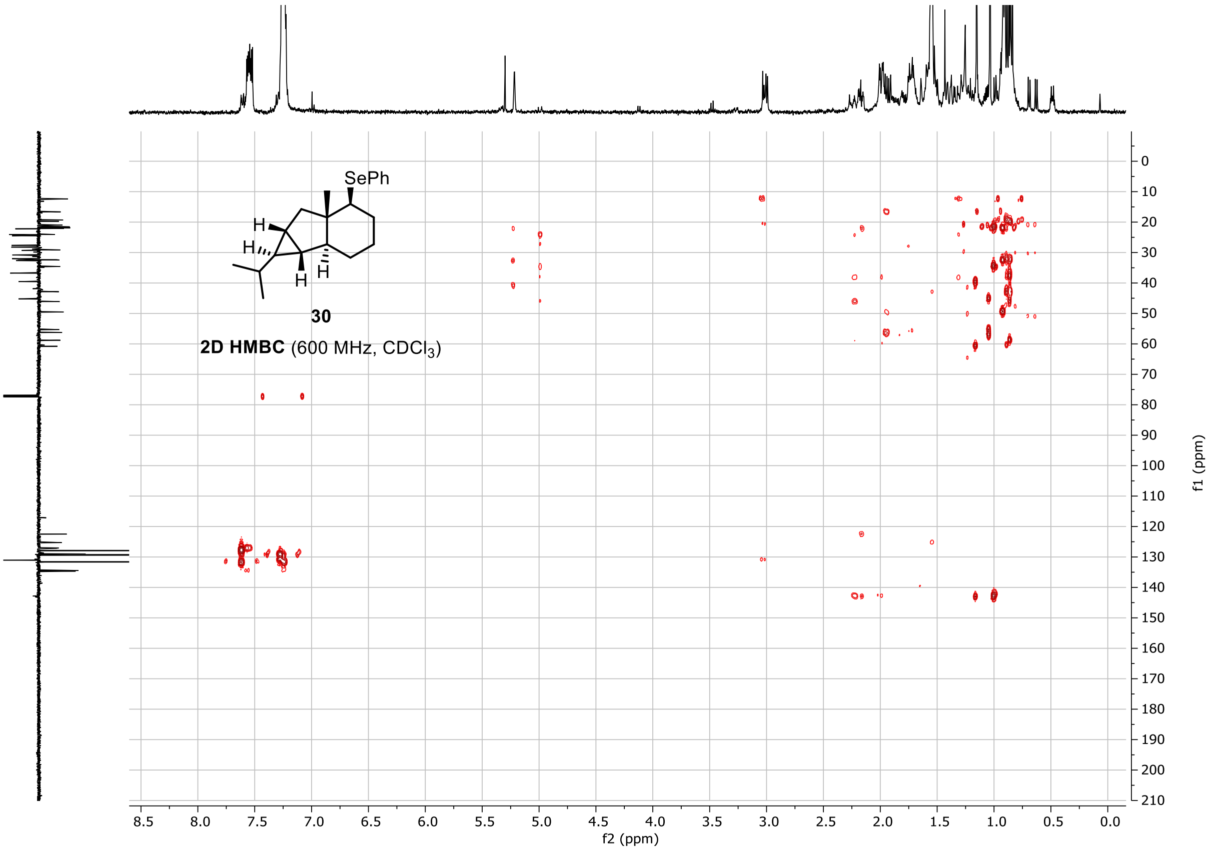
SePh
H
H
H
H
30
2D HMBC (600 MHz, CDCl3)
f2 (ppm)
f1 (ppm)

7. References

[1] P. Pracht, F. Bohle, S. Grimme, *Phys. Chem. Chem. Phys.* **2020**, *22*, 7169–7192.
[2] S. Grimme, *J. Chem. Theory Comput.* **2019**, *15*, 2847–2862.
[3] J. P. Perdew, K. Burke, M. Ernzerhof, *Phys. Rev. Lett.* **1996**, *77*, 3865–3868.
[4] J. P. Perdew, K. Burke, M. Ernzerhof, *Phys. Rev. Lett.* **1997**, *78*, 1396–1396.
[5] C. Adamo, V. Barone, *J. Chem. Phys.* **1999**, *110*, 6158–6170.
[6] S. Grimme, J. Antony, S. Ehrlich, H. Krieg, *J. Chem. Phys.* **2010**, *132*, 154104.
[7] S. Grimme, S. Ehrlich, L. Goerigk, *J. Comput. Chem.* **2011**, *32*, 1456–1465.
[8] F. Weigend, *Phys. Chem. Chem. Phys.* **2006**, *8*, 1057–1065.
[9] F. Weigend, R. Ahlrichs, *Phys. Chem. Chem. Phys.* **2005**, *7*, 3297–3305.
[10] Gaussian 16, Revision C.01, M. J. Frisch, G. W. Trucks, H. B. Schlegel, G. E. Scuseria, M. A. Robb, J. R. Cheeseman, G. Scalmani, V. Barone, G. A. Petersson, H. Nakatsuji, X. Li, M. Caricato, A. V. Marenich, J. Bloino, B. G. Janesko, R. Gomperts, B. Mennucci, H. P. Hratchian, J. V. Ortiz, A. F. Izmaylov, J. L. Sonnenberg, D. Williams-Young, F. Ding, F. Lipparini, F. Egidi, J. Goings, B. Peng, A. Petrone, T. Henderson, D. Ranasinghe, V. G. Zakrzewski, J. Gao, N. Rega, G. Zheng, W. Liang, M. Hada, M. Ehara, K. Toyota, R. Fukuda, J. Hasegawa, M. Ishida, T. Nakajima, Y. Honda, O. Kitao, H. Nakai, T. Vreven, K. Throssell, J. A. Montgomery, Jr., J. E. Peralta, F. Ogliaro, M. J. Bearpark, J. J. Heyd, E. N. Brothers, K. N. Kudin, V. N. Staroverov, T. A. Keith, R. Kobayashi, J. Normand, K. Raghavachari, A. P. Rendell, J. C. Burant, S. S. Iyengar, J. Tomasi, M. Cossi, J. M. Millam, M. Klene, C. Adamo, R. Cammi, J. W. Ochterski, R. L. Martin, K. Morokuma, O. Farkas, J. B. Foresman, and D. J. Fox, Gaussian, Inc., Wallin.
[11] E. Cancès, B. Mennucci, J. Tomasi, *J. Chem. Phys.* **1997**, *107*, 3032–3041.
[12] A. V. Marenich, C. J. Cramer, D. G. Truhlar, *J. Phys. Chem. B* **2009**, *113*, 6378–6396.
[13] A. D. Becke, *J. Chem. Phys.* **1993**, *98*, 5648–5652.
[14] F. Weigend, M. Häser, *Theor. Chem. Acc.* **1997**, *97*, 331–340.
[15] C. Møller, M. S. Plesset, *Phys. Rev.* **1934**, *46*, 618–622.
[16] F. Neese, F. Wennmohs, U. Becker, C. Riplinger, *J. Chem. Phys.* **2020**, *152*, 224108.
[17] M. T. de Oliveira, J. M. A. Alves, N. L. Vrech, A. A. C. Braga, C. A. Barboza, *Phys. Chem. Chem. Phys.* **2023**, *25*, 1903–1922.
[18] B. Maryasin, M. Olbrich, D. Trauner, C. Ochsenfeld, *J. Chem. Theory Comput.* **2015**, *11*, 1020–1026.
[19] S. K. Nistanaki, C. G. Williams, B. Wigman, J. J. Wong, B. C. Haas, S. Popov, J. Werth, M. S. Sigman, K. N. Houk, H. M. Nelson, *Science* **2022**, *378*, 1085–1091.
[20] V. Jo Davisson, A. B. Woodside, C. Dale Poulter, in *Methods Enzymol.*, Academic Press, **1985**, pp. 130–144.
[21] F. Bohlmann, C. Zdero, R. M. King, H. Robinson, *Phytochemistry* **1984**, *23*, 1798–1799.
[22] G. Guella, D. Skropeta, I. Mancini, F. Pietra, *Z. Für Naturforschung B* **2002**, *57*, 1147–1151.
[23] K. Y. Jung, D. S. Kim, S.-H. Park, I. S. Lee, S. R. Oh, J. J. Lee, E.-H. Kim, C. Cheong, H.-K. Lee, *Phytochemistry* **1998**, *48*, 1383–1386.
[24] K. Chen, Y. Ishihara, M. M. Galán, P. S. Baran, *Tetrahedron* **2010**, *66*, 4738–4744.
[25] M. Ohta, S. Kato, T. Sugai, H. Fuwa, *J. Org. Chem.* **2021**, *86*, 5584–5615.
[26] Y.-S. Lu, X.-S. Peng, *Org. Lett.* **2011**, *13*, 2940–2943.
[27] N. Takeda, T. Imamoto, *Org. Synth.* **1999**, *76*, 228.
